# Global map of oxytocin/vasopressin-like neuropeptide signalling in insects

**DOI:** 10.1038/srep39177

**Published:** 2016-12-13

**Authors:** Zita Liutkeviciute, Johannes Koehbach, Thomas Eder, Esther Gil-Mansilla, Christian W. Gruber

**Affiliations:** 1 Medical University of Vienna, Center for Physiology and Pharmacology, Vienna, 1090, Austria; 2 The University of Queensland, School of Biomedical Sciences, St Lucia, 4072, Australia; 3 University of Vienna, CUBE-Division of Computational Systems Biology, Department of Microbiology and Ecosystem Science, Vienna, 1090, Austria; 4 Ludwig Boltzmann Institute for Cancer Research, Medical University of Vienna, 1090 Vienna, Austria

## Abstract

Oxytocin and vasopressin mediate a range of physiological functions that are important for osmoregulation, reproduction, social behaviour, memory and learning. The origin of this signalling system is thought to date back ~600 million years. Oxytocin/vasopressin-like peptides have been identified in several invertebrate species and they appear to be functionally related across the entire animal kingdom. There is little information available about the biology of this peptide G protein-coupled receptor signalling system in insects. Recently over 200 insect genome/transcriptome datasets were released allowing investigation of the molecular structure and phylogenetic distribution of the insect oxytocin/vasopressin orthologue – inotocin peptides and their receptors. The signalling system is present in early arthropods and representatives of some early-diverging lineages. However, Trichoptera, Lepidoptera, Siphonaptera, Mecoptera and Diptera, lack the presence of inotocin genes, which suggests the peptide-receptor system was probably lost in their common ancestor ~280 million-years-ago. In addition we detected several losses of the inotocin signalling system in Hemiptera (white flies, scale insects and aphids), and the complete absence in spiders (Chelicerata). This unique insight into evolutionarily patterns and sequence diversity of neuroendocrine hormones will provide opportunities to elucidate the physiology of the inotocin signalling system in one of the largest group of animals.

Insects (phylum Arthropoda, subphylum Hexapoda) constitute the largest and most diverse group of organisms on earth, contributing to at least half of the global species diversity^1^. There are around one million species described to date, but experts suggest the total number to be between 3 and 80 million^1^. This large number of different insect species, their diverse life styles and various adaptation mechanisms allowed them to occupy and inhabit nearly all terrestrial ecosystems and thus they have become extremely useful and interesting research objects. With the rise of new technologies such as next generation sequencing and functional genomics, the ‘world of insects’ and other arthropods (mites, scorpions, spiders, centipedes, shrimps, etc.) received a lot of attention from the scientific community. Recently a research consortium used next generation sequencing and data analysis of several insect orders to construct a species tree of insects^2^ making it possible to compare and study insect phylogeny at a genetic level.

Here we took advantage of this opportunity to investigate the repertoire of oxytocin/vasopressin-like peptides, namely inotocin peptide hormone precursors, and their cognate family of receptors. Although this peptide G protein-coupled receptor (GPCR) signalling system is thought to date back more than 600 million years^3^, little is known about the phylogenetic distribution and evolution of this peptidergic signalling system within arthropods. In humans the nonapeptides oxytocin and vasopressin are thought to have originated from an ancestral hormone vasotocin^4^. All oxytocin/vasopressin-like peptides share a similar structure with a six residue N-terminal ring formed by a disulfide bond between the conserved cysteines in positions 1 and 6, and an amidated three residue C-terminal tail^5^. They are products arising from larger precursors, which are generally conserved across species. All described precursor molecules have a short signal peptide upstream of the nonapeptide domain, which is followed by a canonical amidation sequence. The large C-terminal domain encodes for a protein called neurophysin and contains 14 conserved cysteines. In humans and other mammals the two nonapeptides oxytocin (CYIQNCPLG) and vasopressin (CYFQNCPRG) act via four cognate GPCRs, i.e. the oxytocin and three vasopressin V_1a_-, V_1b_- and V_2_-receptors. Oxytocin and vasopressin signalling is involved in a variety of vital functions both in the periphery and the central nervous system. Oxytocin plays crucial roles in reproduction including uterine contraction or milk ejection, and functions as neurotransmitter in complex social behaviour such as bonding, stress or anxiety^6^,^7^. Vasopressin regulates fluid balance and blood pressure and is implicated in memory, learning and aggressive behaviour^6^,^8^. Homologous peptides have been identified in a variety of species including both vertebrates and invertebrates^5^ and are known under different names such as mesotocin (CYIQNCPIG)^3^, isotocin (CYISNCPLG)^3^, inotocin (CLITNCPRG)^9^,^10^,^11^ and many others. Notably the physiological functions of oxytocin/vasopressin-like peptides across evolutionarily distant species are conserved^9^. Their involvement in this repertoire of physiological functions and pathological conditions has further resulted in great interest in exploring the potential of these peptides in drug development and to assess their role for therapeutic applications^12^.

In the present study we analysed over 260 invertebrate species of insects and other arthropods for the presence of the inotocin peptide GPCR signalling system. By sequence mining of publicly available genome and transcriptome datasets it was possible to identify and annotate peptide precursor and receptor coding sequences. Furthermore we carried out sequence analyses for conserved motifs on the sequences that were found and performed a phylogenetic analysis of the inotocin receptors. This revealed a unique insight into the phylogenetic distribution and aspects of the evolution of the oxytocin/vasopressin-like peptide GPCR signalling system in arthropods.

## Results

### Identification of inotocin precursors and peptides

We analysed publicly available genome and transcriptome datasets of 269 species for the presence of the inotocin signalling system. Human oxytocin and vasopressin and published invertebrate oxytocin/vasopressin-like peptide precursors were used for BLAST searches against a custom built insect database as well as NCBI databases. All hits were manually refined and evidence for inotocin and inotocin-like peptide precursors was obtained for a total of 144 species, including full-length and partial sequences (Supplementary Data S1). We identified 121 complete sequences that contain the mature peptide domain (Supplementary Figure S1, Supplementary Data S1), and 23 partial sequences [*in 12 out of these 23 species that contain partial sequences we found putative inotocin receptor sequences as well; in the 11 remaining species we cannot exclude the possibility that these inotocin precursor sequences may have been derived as artefacts or sequencing errors*] that shared similarity to the neurophysin domain of oxytocin/vasopressin-like precursors (Supplementary Figure S1, Supplementary Data S1). In the majority of sampled species we identified one single gene or transcript. In addition, we found two inotocin precursors in *Scolopendra subspinipes* (centipede), *Atelura formicaria* (silverfish) and *Leptinotarsa decemlineata* (beetle) and three different precursor genes/transcripts in *Calanus finmarchicus* (crustacean) (Supplementary Figure S2). The two *Scolopendra subspinipes* inotocin precursors encode two different inotocin-like peptides, CFITNCPPG, which has been found in many other species, and CYIINCIDND, a putative decapeptide that was identified only once. The two *Leptinotarsa decemlineata* precursors also encode two different inotocin-like peptides, CLITNCP**K**G and CLITNCP**I**G. The different inotocin precursor transcripts in *Atelura formicaria* and *Calanus finmarchicus* are encoding the same peptide in each species.

**Figure 1:**
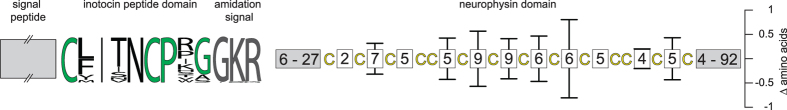
Inotocin precursor structure in arthropods and sequence logo of mature peptides. Inotocin prepropeptides consist of a signal peptide (in dark grey), the inotocin peptide domain, an amidation signal and the neurophysin domain. The mature peptide domain and the processing signal of arthropod precursors have been illustrated by a frequency plot of various inotocin sequences identified in this study (Table 1). Conserved residues within the nonapeptide domain, i.e. the two cysteine residues that form a disulfide bond as well as the C-terminal proline and glycine residues are highlighted in green. The dibasic amidation signal GKR is highlighted in grey. Conserved cysteines within the neurophysin domain are highlighted in yellow and the mean length of inter-cysteine sequences are shown as number of residues. Error bars indicate variable and conserved regions and represent the standard deviation of inter-cysteine sequences (n = 71–103). Regions of the precursor sequence outside the conserved cysteines of the neurophysin domain are indicated in light grey and numbers indicate as the minimal and maximal number of residues in each region, respectively.

Sequence analysis of all putative inotocin precursors revealed that there is some degree of conservation within parts of the cysteine-rich C-terminal neurophysin domain (Fig. 1). However, none of these C-terminal domains exhibited any obvious similarity to the copeptin domain of vertebrate vasopressin-like precursors (Supplementary Figure S1). Sequence analysis of the mature inotocin domain revealed 21 different peptide sequences with the consensus C*X*_4_CP*X*G (Table 1) whereof most have not been reported previously. The nonapeptide ligands show the highest sequence variation in position 2 within the cyclic N-terminal ring as well as in position 8. This is in line with previous studies across a range of species^13^. Of the 110 analysed peptide precursors, 103 sequences contain the conserved amidated C-terminal glycine, and 109 were found to have the conserved dibasic amidation motif (GKR was met most often, infrequently we also observed GRK and GKK) (Supplementary Figure S1). Notably, inotocin-like peptides from *Dermatophagoides farinae, Sarcoptes scabiei* and *Calanus finmarchicus* (2 peptides) contain an alanine or serine residue instead of glycine in position 9; in addition we identified three sequences of putative decapeptides that contain a 4-residue tail before the processing site, i.e. CYIINCIDND in *Scolopendra subspinipes*, CFITNCPVGG within *Lepeophtheirus salmonus* and CFITNCPVGS within *Caligus rogercresseyi*. One unusual sequence had neither an amidated glycine nor a typical amidation processing site (*Speleonectes cf. tulumensis* - CFILDCPLM-IRN) and hence it remains doubtful whether this precursor will actually be processed to a mature inotocin-like peptide.

### Identification of inotocin receptor sequences

Similar to the presence of inotocin peptide sequences we identified 153 putative inotocin receptor sequences within 269 analysed species (Supplementary Figure S3, Supplementary Data S2). Several species among the early-diverging branches (subphyla Chelicarata and Myriapoda) were found to have multiple copies of inotocin receptors, i.e. *Limulus polyphemus* (3 subtypes), *Ixodes scapularis* (3 subtypes), *Metaseiulus occidentalis* (2 subtypes), *Varroa destructor* (2 subtypes), *Achipteria coleoptrata* (2 subtypes), *Platynothrus peltifer* (5 subtypes) and *Strigamia maritima* (2 subtypes). Additionally we found 4 different subtypes in crustaceans, e.g. *Daphnia magna* (Supplementary Figure S2).

To distinguish inotocin receptors from closely related crustacean cardioactive peptide (CCAP) receptors, which share a high degree of sequence similarity and evolved by gene duplication before Protostomia and Deutorostomia have separated, we aimed to identify sequence motifs exclusively present in the inotocin receptors. Firstly we prepared an alignment of 68 CCAP receptors of insects available at NCBI and 68 invertebrate and vertebrate oxytocin/vasopressin-like receptors that have been previously analysed^13^. At the end of transmembrane domain 2 and at the beginning of extracellular loop 1 we were able to utilize a highly conserved motif consisting of 12 residues with the consensus motif *X***PQ***X*_2_**W***X*_5-6_**F**^14^. In all of the analysed arthropod receptors, at least 3 of the 4 residues (underlined and highlighted in bold) are identical. On the contrary, for CCAP receptors this motif is considerably different (*X*_2_**D***X*_8_**W**; Supplementary Figure S4). For partial receptor sequences, which lack the N-terminal domain, we utilized the common NP motif located in transmembrane domain 7 of GPCRs to gain confidence in receptor assignments^15^. In oxytocin/vasopressin-like receptors the first (cysteine) and last (tryptophan) residue (**C***X*NP**W),** appear to be conserved, which allows distinguishing them from CCAP receptors that contain alanine and valine/leucine/phenylalanine, respectively (**A***X*NP**V/L/F**) in these two positions (Supplementary Figure S4). To confirm receptor assignments, we performed phylogenetic tree analysis of all oxytocin/vasopressin-like receptors. The newly annotated inotocin receptors grouped together with previously known oxytocin/vasopressin-like receptors, and form a separate branch with high confidence (>95%) distinct from CCAP receptors (Supplementary Figure S5).

### Presence and absence of the inotocin peptide GPCR signalling system

**Figure 2:**
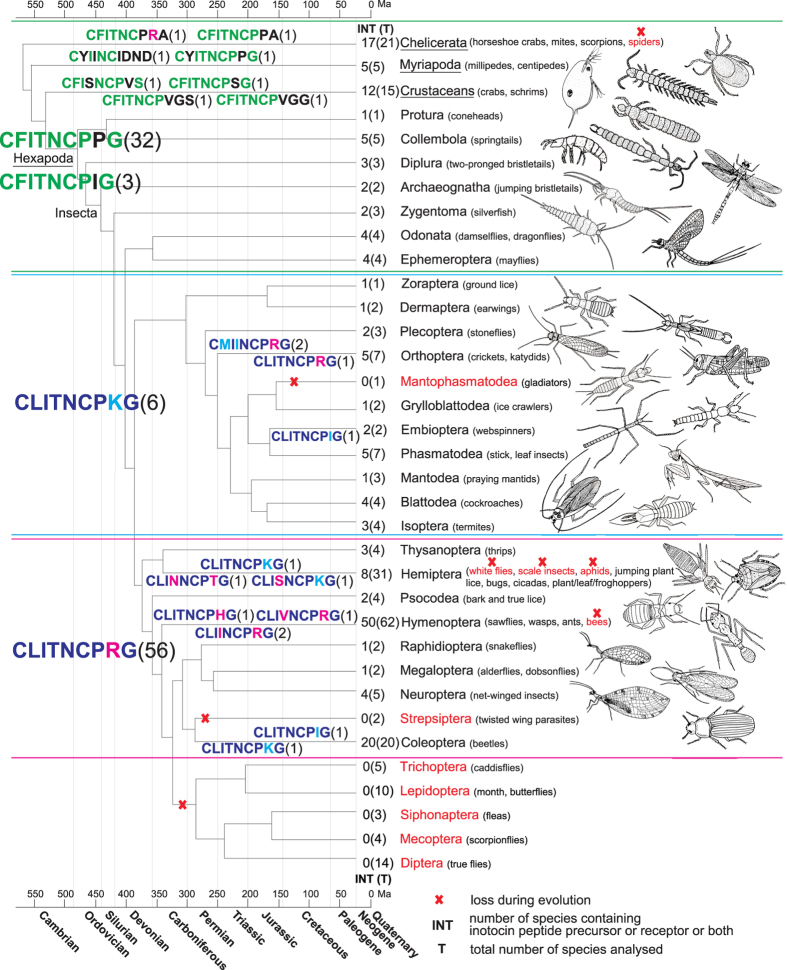
Map of inotocin peptide GPCR signalling in arthropods. A phylogenetic map based on the recently established insect phylogeny^2^ is shown. The absence of the inotocin signalling system is highlighted in red. Different putative peptide sequences are shown in different colours to indicate the diversity and distribution throughout the phylogeny. The numbers in brackets next to the peptide sequences indicate the frequencies of occurrence (from a total of 121 analysed precursor sequences that contained a mature peptide domain). Number of species where the inotocin signalling system (receptor and/or precursor) is present (INT) as well as the total number of sampled species (T) has been indicated next to the tree branches. The inotocin signalling system is confined to specific groups of arthropods. For clarity of this phylogenetic illustration, the upper three groups of Arthropoda (Chelicerata, Myriapoda and Crustaceans; underlined) represent subphyla. All other groups denote orders of the subphylum Hexapoda; the class of Insecta comprises the orders Archaeognatha to Diptera.

Having identified both inotocin peptides and receptors it was of interest to map their presence across all sampled arthropod species. In 105 species we were able to identify both an inotocin-like precursor as well as the cognate receptor, and in another 64 species we could establish evidence for the presence of the inotocin peptide GPCR signalling system by identification of either peptide or receptor. In addition there are 100 species that showed no evidence for the presence of inotocin precursor sequences or receptors (Supplementary Table S1). Bearing in mind that *in silico* mining strongly relies on raw sequence data quality, completeness of genomes and sampling size, some orders (for instance Mantophasmatodea and Strepsiptera) only contained a few sampled species and hence the apparent absence of the inotocin signalling system in these orders should be treated carefully. Furthermore we identified inotocin precursor and receptor sequences in one species (*Arachnocampa luminosa*) of the order Diptera (flies), which most likely was due to a contamination of the dataset (Supplementary Figure S6). The taxonomic representation of inotocin peptide sequences within arthropods suggests that certain putative inotocin-like peptides are exclusively expressed by specific insect orders and/or subphyla of arthropods (Fig. 2). For example, the putative peptide C**F**ITNCP*X*G (with some exceptions) is distributed among Chelicerata, Myriapoda, Crustaceans and early-diverging lineages of Hexapoda; the putative peptide CLITNCPKG is almost exclusively present in the monophyletic insects group Polyneoptera (inclusive orders of Zoraptera to Isoptera), and the putative peptide CLITNCPRG is prevalent amongst the latest diverged lines of insects (orders Thysanoptera to Coleoptera).

There are some heterogeneous groups of which some species do and others don’t contain components of the inotocin signalling system; for instance in Hymenoptera and Hemiptera there appear to be losses of inotocin precursor and receptor genes (Supplementary Table S1). In Hemiptera there are four monophyletic suborders, Sternorryncha (jumping plant lice, aphids, whiteflies, scale insects), Coleorrhyncha (moss bugs), Heteroptera (bugs) and Auchenorrhyncha (cicadas and plant/leaf/froghoppers)^16^,^17^,^18^. We sampled 31 Hemipteran species of all four suborders and have identified heterogeneous results within three of them. Within Sternorryncha, the inotocin signalling system appears to be lost in white flies (2 species sampled), scale insects (7 species) and aphids (4 species), but it is present in jumping plant lice (3 species). Within 10 species of Heteroptera, the signalling system has been identified only in the shield bug *Halyomorha halys* (both inotocin precursor and receptor) and the large milkweed bug *Oncopelcus fasciatus* (inotocin precursor only); in Auchenorrhyncha it was found in the plant/leaf/froghoppers, but not in cicadas. In a total of 62 analysed species of Hymenoptera, all sawflies, wasps (30 species) and ants (20 species) were identified to contain components of the inotocin signalling system. In agreement with the scientific literature^10^,^11^ we did not find neither inotocin precursor nor receptor genes in bees (superfamily Apoidea: 12 species sampled) (Supplementary Table S1).

## Discussion

Oxytocin and vasopressin peptide ligands and their cognate receptors comprise one of the oldest and best-studied peptide GPCR signalling systems. Being present across a range of distantly-related animal species and dating back more than 600 million years in evolution, it has been an intriguing model system for comparative endocrinology and neurophysiology^19^,^20^. Furthermore oxytocin/vasopressin-like signalling has been extensively studied for its biological function, which appears to be conserved across vertebrates and invertebrates^9^,^19^. Due to a lack of knowledge about the phylogenetic distribution of this peptide GPCR signalling system, only a few isolated functional studies with the insect oxytocin/vasopressin orthologue inotocin have been carried out to date; including for instance the red flour beetle *Tribolium castaneum*^11^,^21^ and the migratory locust *Locusta migratoria*^22^,^23^. Thus the biology of inotocin neuropeptide signalling in insects and arthropods is still puzzling and requires further studies.

In this project, we analysed the presence or absence of ligands and receptors of the inotocin signalling system in Arthropoda and compared these data to the recently established phylogeny^2^. We have found evidence for the presence of components of this signalling system in all subphyla of Arthropoda and in 25 out of 32 orders of the subphylum Hexapoda. Coleoptera (beetles), being the biggest order of insects and accounting for about 40% of all insect species^1^, is very homogeneous: all 20 analysed species exhibited evidence for the presence of the inotocin signalling system. In contrast, there is a certain degree of heterogeneity within some groups, for example spiders (Chelicerata), white flies, scale insects, aphids (Hemiptera) and bees (Hymenoptera), which appear to lack inotocin signalling components, whereas other species within those groups, for example mites, scorpions (Chelicerata), jumping plant lice, plant/leaf/froghoppers (Hemiptera), sawflies, wasps and ants (Hymenoptera) appear to contain the inotocin signalling system. On the other hand, several insect orders (7 in total) completely lack peptide or receptor sequences indicating that the inotocin signalling system is confined to distinct phylogenetic groups. Having analysed many more species we confirmed previous findings that inotocin signalling system was lost several times during insect evolution, namely in bees (Hymenoptera), flies (Diptera) and butterflies (Lepidoptera)^10^,^11^. Additionally, here we show for the first time that the inotocin signalling system was lost in spiders at least ~240 million years ago at the basal line of Araneomorphae^24^ which cover 90% of spider species diversity^25^ (all 4 spider genomes analysed here belong to this group). Moreover, several losses were observed in the order Hymenoptera where the inotocin peptide GPCR signalling system is not present in white flies, scale insects, aphids, most bugs and cicadas (more details in Results). The inotocin signalling system appears to exists in early-diverging insect orders, whereas it is missing in the late-diverging insects orders and presumably has been lost ~280 million years ago in the common ancestor of Trichoptera, Lepidoptera, Siphonaptera, Mecoptera and Diptera (Fig. 2). Similarly, the loss was also reported in several species of nematodes, although most of them, including *C. elegans*, possess oxytocin/vasopressin-like precursor and receptors^19^.

After defining a global map for the phylogenetic distribution of inotocin peptide GPCR signalling system in insects and other arthropods, we aimed to analyse the molecular structure of inotocin peptides and receptors. The vast majority of all sampled species contain one gene encoding for the inotocin receptor and one for the precursor (referred to as prepropeptide). All of the identified prepropeptides have a similar structure containing a signal peptide, a mature inotocin domain, an amidation/processing signal and a neurophysin domain (Fig. 1). Only within *Speleonectes cf. tulumensis* we did not find the dibasic amidation signal between the peptide and neurophysin domain. There are a few exceptions, for instance the Colorado potato beetle (*Leptinotarsa decemlineata*) which has two copies of inotocin prepropeptides that encode two different inotocin-like peptides (CLITNCP**K**G and CLITNCP**I**G) while all other analysed beetle datasets showed evidence only for one copy of the prepropeptide encoding for the putative mature inotocin peptide CLITNCP**R**G. Interestingly, six of the identified multi-copy receptor species were found within the subphylum Chelicerata and one in Myriapoda, but not a single species within the Hexapoda. We also analysed four different *Daphnia magna* transcripts of inotocin receptors, which are probably the result of alternative splicing since these sequences are almost identical except for minor deletions/insertions in intracellular loop 3 as well transmembrane domains 3 and 5. The presence of multiple copies in some of the species could be explained by whole genome or large scale duplication events, which have been reported for *Limulus polyphemus*^26^,^27^, *Ixodes scapularis*^28^, *Calanus finmarchicus*^29^ and *Strigamia maritima*^30^. Local and whole genome duplication and deletion events during vertebrate evolution led to different number of gene copies (two or more) of oxytocin/vasopressin receptors and precursors within distinct vertebrate species^31^,^32^. Likewise among invertebrates more than one copy of receptor and/or precursor is known for nematodes^19^ or molluscs^33^,^34^,^35^,^36^. In some molluscs the expression pattern of three oxytocin/vasopressin-like (conopressin) receptors and two precursors in different organs is indicative for different biological functions^34^,^36^. Thus, these Chelicerata species mentioned above could become interesting model systems to study the biological functions of multiple and different copies of inotocin-like peptides and receptors in arthropods.

Besides the advantages of obtaining deeper insight into the phylogenetic distribution and evolution of the inotocin signalling system as discussed above, the identification of novel inotocin peptides also provides opportunities with regard to GPCR pharmacology. Novel peptide sequences may prove useful in the development of receptor specific ligands for the human oxytocin/vasopressin receptor family^5^. A plethora of chemicals have been synthesized over the last decades^12^, yet there is still a great demand for selective ligands that target specific receptors or specific receptor-mediated signalling pathways. Therefore the identification of naturally-occurring oxytocin/vasopressin-like peptides provides valuable peptides for the design and development of novel leads that selectively target one receptor subtype, and hence they are considered as invaluable tools for pharmacology. In fact, the discovery and characterization of Conopressin-T, an oxytocin/vasopressin-like peptide isolated from cone snails, provided new impetus for the development of antagonists for the human receptors^37^. The availability of genome and transcriptome datasets constitutes a useful resource for the discovery of novel peptide ligands using *in silico* mining approaches^10^,^38^. In this current study we discovered 21 different putative oxytocin/vasopressin-like peptides, whereof most are previously unknown sequences. Notably the inotocin-like sequences identified in *Scolopendra subspinipes, Lepeophtheirus salmonis* and *Caligus rogercresseyi* are decapeptides. Such unusual ‘longer’ oxytocin/vasopressin-like analogues have previously been identified in *C. elegans*^39^,^40^, as well as in *Ciona intestinalis*^41^ and *Styela plicata*^42^. Using detailed consensus analysis of the sequences identified in this study, we found that insect inotocin-like peptides are highly conserved; four residues in the 6-residue cyclic ring are identical (C*X*I*X*NC) and are similar to the ancestral vasotocin sequence (Table 1).

Similarly, the C-terminal tripeptide of oxytocin/vasopressin-like molecules is of great importance for receptor binding and activation. For example, peptides lacking the tail sequence are inactive at the human oxytocin receptor^43^. As opposed to a loss of activity, the C-terminal deamination leads to more hydrophobic and more potent analogues^12^,^44^. Besides the C-terminal residue, positions 4 and 8 are known to display the highest sequence variability^5^ in oxytocin/vasopressin-like peptides. In this study we have identified novel sequence variations of position 8: the inotocin-like peptide of *Dinoponera quadriceps* contains a histidine, and the inotocin-like peptides of *Tigriopus californicus* as well as *Diaphorina citri* were found to have more hydrophilic residues, i.e. a serine and threonine respectively. Histidine is a basic amino acid similar to lysine and arginine, whereas serine and threonine are hydrophilic amino acids and thus may represent interesting starting points for bioactivity testing. Interestingly two inotocin-like peptide sequences, i.e. from *Dermatophagoides farinae* and *Sarcoptes scabiei* were found to have a C-terminal alanine, both followed by an amidation domain. Given the promising pharmacology of certain invertebrate oxytocin/vasopressin-like peptides as pointed out above^37^, there is potential that this natural library of neuropeptides from insects may advance the design of novel ligands and probes for the human oxytocin and vasopressin GPCRs^5^.

## Conclusion

Peptides and their cognate receptors are essential components of neuronal communication and physiology in animals^45^. Many peptidergic systems have been characterized in vertebrates, but their phylogenetic distribution in arthropods, and in particular in insects, is little understood. We interrogated genomic and transcriptomic sequence databases and used phylogenetic reconstruction tools to report a first map of the inotocin peptide GPCR signalling system in insects. Our analysis provides a comprehensive view of neuropeptide phylogeny that will pave the way for comparative and molecular studies leading to a better understanding of insect physiology. Taken together our results provide a unique insight into the phylogenetic distribution and molecular structure of a particular neuropeptide GPCR signalling system – inotocin – in insects and other arthropods. We believe that our approach will be useful to analyse other neuropeptide systems in the largest group of animals. At the very least, our discoveries will provide starting points for fundamental studies about the physiology and function of oxytocin/vasopressin-related signalling in arthropods, and some of these newly-derived inotocin sequences may yield useful peptides and tools in a translational approach to study the pharmacology of human oxytocin/vasopressin peptides and their receptors.

## Methods

### Preparation of datasets

The insect transcript assemblies were retrieved from public databases described by Misof *et al*.^2^. This included 103 transcript assemblies from the BioProject PRJNA183205: “The 1KITE project: evolution of insects” and 40 from diverse other resources^2^. A list of all species and their respective accession numbers is given in Supplementary Table S2. Additional sequences were derived from the NCBI transcriptome shotgun assembly, whole-genome shotgun, and non-redundant databases (Supplementary Table S1).

### In silico mining using tBLASTn similarity searches

Selected published oxytocin/vasopressin-like and inotocin precursor amino acid sequences (Accessions: *Tribolium castaneum* NP_001078831.1, *Acromyrmex echinatior* XP_011065328.1, *Nasonia vitripennis* XP_001606547.1 and *Daphnia pulex* EFX71881.1) were used to perform off-line database searches using tblastn and blastp (version 2.2.31) with an E-value cut-off of 10 e^−4^ and default parameters (scoring matrix: BLOSUM62; gap opening penalty: 11; gap extension penalty: 1)^46^. Bedtools^47^ (version 2.25.0) was used to retrieve the respective sequences from the published oxytocin/vasopressin-like and inotocin sequences. A custom shell script was applied to align the species list with hits of precursors and receptors, respectively. Additionally we queried the NCBI databases via tBLASTn online. Hits with high probability scores (E-value > 10^−4^) were discarded and other hits were manually annotated and verified.

### Annotation of peptide precursor and receptor sequences

Annotation of peptide precursors and receptors was achieved manually. In genomes the exon/intron boundaries were annotated manually using the transcriptome sequence of evolutionarily nearest species as a reference. Sequence hits of inotocin-like precursors derived from automated similarity searches were assigned and confirmed by homology to related oxytocin/vasopressin-like precursors, and the presence of cysteines in position 1 and 6 of the mature peptide domain followed by an amidation domain and a cysteine-rich neurophysin domain. Of all hits, the 110 annotated sequences (Supplementary Data S1) were used for further analyses. tBLASTn receptor hits with homology to known oxytocin/vasopressin-like receptors (probability scores, i.e. E-values < 10^−4^) were assigned and confirmed based on two unique sequence criteria: (i) presence of a conserved motif at the end of transmembrane domain 2 and at the beginning of extracellular loop 1 (*X***PQ***X*_2_**W***X*_5-6_**F**), and (ii) presence of a **C***X*NP**W** motif in transmembrane domain 7 (Supplementary Figure S4). Sequences of putative receptors with identity in at least three residues (shown in bold above) of the first motif (i), and/or identity in at least one residue of the second motif (ii) were considered as positive hits (Supplementary Figure S4). Similar motifs were identified in a previous study comparing neuropeptide S and oxytocin/vasopressin-like receptors^15^.

### Sequence alignment analysis

ClustalO (http://www.ebi.ac.uk/Tools/msa/clustalo/) was used to perform alignments analysis of peptides, precursor proteins and receptor sequences. Signal peptides, mature peptide domain, amidation signal and neurophysin domain were assigned based on homology to query sequences. For the preparation of sequence logos the free WebLogo tool (http://weblogo.berkeley.edu/) was used.

### Phylogenetic analysis

For the generation of phylogenetic trees a maximum likelihood phylogenic reconstruction was done via RaxML^48^,^49^ (version 8.2.2) using the PROTGAMMABLOSUM62 model and 100 bootstrap replicates for both multiple sequence alignments. Dendroscope^50^ was used for midpoint rooting and visualizing the phylogenetic analysis.

### Assembly completeness check

To elucidate if inotocin peptide and receptor found in *A. luminosa* are native or a contamination of *B. koreanus*, the two transcriptomes were checked for completeness by appling BUSCO^51^ (version 1.1b1) with default parameters for transcriptomes against Arthropoda and Metazoa datasets.

## Additional Information

**How to cite this article**: Liutkeviciute, Z. *et al*. Global map of oxytocin/vasopressin-like neuropeptide signalling in insects. *Sci. Rep.* **6**, 39177; doi: 10.1038/srep39177 (2016).

**Publisher's note:** Springer Nature remains neutral with regard to jurisdictional claims in published maps and institutional affiliations.

## Supplementary Material

Supplementary Information

## Figures and Tables

**Table 1:** Oxytocin, vasopressin and inotocin peptide sequences.

Sequence^a^ (Frequency^b^)	Name	Group of animals or Species	Reference
**C**YIQN**C**PLG*	oxytocin	mammals	—
**C**YFQN**C**PRG*	vasopressin	mammals	—
**C**YIQN**C**PRG*	vasotocin	non-mammalian vertebrates	—
**C**LITN**C**PRG* (57)	inotocin	insects	ref. 11
**C**FITN**C**PPG* (32)	inotocin-like	arthropods	ref. 11
**C**LITN**C**PKG* (8)	inotocin-like	insects	this study
**C**FITN**C**PIG* (3)		*Symphylella vulgaris, Metaseiulus occidentalis, Varroa destructor*	ref. 9
**C**LIIN**C**PRG* (2)		*Athalia rosae Neodiprion lecontei*	this study
**C**LITN**C**PIG* (2)		*Haploembia palaui Leptinotarsa decemlineata*	this study
**C**FISN**C**PVS* (2)		*Calanus finmarchicus*	this study
**C**MIIN**C**PRG* (2)		*Gryllotalpa sp. Teleogryllus commodus*	this study
**C**FITN**C**PRA* (1)		*Dermatophagoides farinae*	this study
**C**FITN**C**PPA* (1)		*Sarcoptes scabiei*	this study
**C**FITN**C**PSG* (1)		*Tigriopus californicus*	this study
**C**LIVN**C**PRG* (1)		*Camponotus floridanus*	ref. 10
**C**LITN**C**PHG* (1)		*Dinoponera quadriceps*	this study
**C**YITN**C**PWG*^c^ (1)		*Arachnocampa luminosa*	this study
**C**LISN**C**PKG* (1)		*Pachypsylla venusta*	this study
**C**LINN**C**PTG* (1)		*Diaphorina citri*	this study
**C**YITN**C**PPG* (1)		*Strigamia maritima*	refs 9,30
**C**FILD**C**PLM^d^ (1)		*Speleonectes cf. tulumensis*	this study
**C**YIIN**C**IDND* (1)		*Scolopendra subspinipes*	this study
**C**FITN**C**PVGG* (1)		*Lepeophtheirus salmonis*	this study
**C**FITN**C**PVGS* (1)		*Caligus rogercresseyi*	this study

^a^Conserved cysteine (positions 1 and 6) residues are highlighted in bold and presence of canonical sequence for amidation at the C-terminus is indicated by an asterisk;

^b^Frequency of occurrence is shown in total numbers (see Fig. 2) based on the analysis of this study;

^c^The peptide identified in *A. luminosa* is considered an artefact (Supplementary Figure S6);

^d^Typical amidation processing site is missing.
