# Supplementary material for: Global map of oxytocin/vasopressin-like neuropeptide signalling in insects
Source: Sci Rep. 2016 Dec 13;6:39177. doi: 10.1038/srep39177 (PMC5153645; doi:10.1038/srep39177)
Supplement: Supplementary Information [file srep39177-s1.pdf]

Supplementary Information

Global map of oxytocin/vasopressin-like neuropeptide signalling in insects

Zita Liutkeviciute[1,2], Johannes Koehbach[2], Thomas Eder[3,4], Esther Gil-Mansilla[1] and Christian W. Gruber[1,2]

[1]*Medical University of Vienna, Center for Physiology and Pharmacology, Vienna, 1090, Austria*

[2]*The University of Queensland, School of Biomedical Sciences, St Lucia, 4072, Australia*

[3]*University of Vienna, CUBE-Division of Computational Systems Biology, Department of Microbiology and Ecosystem Science, Vienna, 1090, Austria*

[4]*Ludwig Boltzmann Institute for Cancer Research, Medical University of Vienna, 1090 Vienna, Austria*

Table of Contents

Supplementary Figure S1. Alignment of inotocin precursors S2

Supplementary Figure S2. Alignment of multiple copies of inotocin precursors and receptors. S13

Supplementary Figure S3........ S18

Supplementary Figure S4. Discrimination between oxytocin/vasopressin-like and crustacean cardioactive peptide receptors........ S43

Supplementary Figure S5. Phylogenetic tree of inotocin receptors........ S46

Supplementary Figure S6. Alignment of selected inotocin-like receptors and precursors........ S48

Supplementary Data S1. Putative inotocin precursors........ S50

Supplementary Data S2. Putative inotocin receptors........ S59

Supplementary Table S1. List of analysed arthropod species S82

Supplementary Table S2. List of species and accession numbers used to prepare dataset S90

Supplementary References........ S94

Supplementary Figure S1. Alignment of inotocin precursors. Putative mature inotocin peptide sequences are highlighted in green; amidation sequences are shown in grey; conserved vicinal cysteine pairs of the neurophysin domain are highlighted in magenta; additional C-terminal residues of decapeptides as well as ambiguous C-terminal peptide sequences are highlighted in red. (A) Alignment of 110 inotocin precursor sequences encoding for putative inotocin and inotocin-like peptides. (B) Alignment of 23 inotocin precursors containing neurophysin domains but lacking inotocin peptide domains.

(A)

```
                                   1                                                50
Calanus_finmarchicus1              MITSWKILDPRIRRSWSFLIILLAQTMSASHGCFISNCPVSGKKRSVGEE
Calanus_finmarchicus3              MITSLKILDPSIRRSWRFLMILLAHTIPDSHGCFISNCPVSGKKRSVGEE
Daphnia_pulex                      -------MAGLWTFCLIALSMTEMIIPLTAKPCFITNCPPGGKRSSQLVE
Daphnia_magna                      -------MAALWTLCLIAFSILEMTMPSAAKPCFITNCPPGGKRSGHVSE
Lepeophtheirus_salmonis            -------------MAFGYPLITLLLIFQIANACFITNCPVGGGKKGSGST
Caligus_rogercresseyi              ---------------------MALCLLQVTESCFITNCPVGSGRKRADPG
Dermatophagoides_farina            ---MFRRESFSMMKIILVLCLIWICLFNDVRGCFITNCPRAGKRSIMTES
Sarcoptes_scabiei                  ---MLFQQTNYLFRSISILVMIVLIVEN-VRGCFITNCPPAGKRSMLAAI
Symphylella_vulgaris               ---------MSSGKSDITNAFVLLTFVSTVSACFITNCPIGGKRS-----
Tigriopus_californicus             ------MQITFDRRCFIVLGLLSLLKAVSVNSCFITNCPSGGKKR-----
Metaseiulus_occidentalis           ---------------MKLVALYFLSVSSLSWACFITNCPIGGKRS-----
Varroa_destructor                  ---------------MKLHVLVLANIVGLSLTCFITNCPIGGKRSD----
Gryllotalpa_sp                     --------------MKVYYLLVFISLIVLNTACMIINCPRGGKR-AMKIN
Teleogryllus_commodus              ----------MAQFKLYSNIIVLMMILTFSCACMIINCPRGGKRSAMENN
Haploembia_palaui                  -----------MINFRFARFIILITLAAVCTACLITNCPIGGKRSLLHG-
Aposthonia_japonica                -----------MVNFRFARFIILITLAVVCTGCLITNCPKGGKRSLAHG-
Zootermopsis_nevadensis            ------------MKMQLGTATLLAVFISLCTACLITNCPKGGKRAGTHSQ
Blattella_germanica                ----------------MQLALVFVLFCAVCRACLITNCPKGGKRAGVQSQ
Galloisiana_yuasai                 ----------MRLKQCLRMLALLVAVVGISSACLITNCPKGGKRALNHS-
Locusta_migratoria_manilensis      --------------MKPQLAVLLALVVSLAAACLITNCPRGGKR------
Nilaparvata_lugens                 ------------MKNTTSIGIMLFYAVGLAAGCLITNCPRGGKRGLHSYM
Halyomorpha_halys                  ----------------MYRALLFLTALCLVQSCLITNCPRGGKRSLRPGL
Homalodisca_vitripennis            -----------------MRGAVVLTVCSLATACLITNCPKGGKRSQSTID
Gynaikothrips_ficorum              -------------MICVKIPSIFLLLLAMTSACLITNCPRGGKRNGARPP
Cotesia_vestalis                   ---------------MMHKFLLVFFFITSSFACLITNCPRGGKRGES---
Microplitis_demolitor              ---------MNLPGTMIRKIFAVVFLLSTSFACLITNCPRGGKRGES---
Fopius_arisanus                    ---------------MSKIILIMSALICMSSACLITNCPRGGKRGEN---
Diachasma_alloeum                  ---------------MSKIILIISALVCVSCACLITNCPRGGKRGEN---
Telenomus_podisi                   ---------------MIAWVIVLVSIFSLSSACLITNCPRGGKRADQ---
Nasonia_giraulti                   ---------------MSKVIIVLTTLVSLSYGCLITNCPRGGKRGD----
Nasonia_vitripenis                 ---------------MSKVIIVLTTLVALSYGCLITNCPRGGKRGD----
Copidosoma_floridanum              ---------------MLKVSLVLLALVSVAYSCLITNCPRGGKRGD----
Trichogramma_pretiosum             --------------MLKVIVFVFVMLFSLSHCCLITNCPRGGKRGD----
Brachycistis_timberlakei           ---------------MLKKFILIAILISLSYGCLITNCPRGGKRGE----
Crioscolia_alcione                 ------------------------------YGCLITNCPRGGKRGE----
Chrysis_viridula                   -----------MQYKMLTKLILFASMFAVVYGCLITNCPRGGKRGD----
Trachymyrmex_septentrionalis       ---------------MLKELIVFASLIFLSYACLITNCPRGGKRSD----
Trachymyrmex_cornetzi              ---------------MLKELIVFASLIFLSYACLITNCPRGGKRSD----
Atta_cephalotes                    ---------------MLKELIVFASLIFLSYACLITNCPRGGKRSD----
Trachymyrmex_zeteki                ---------------MLKELIVFASLIFLSYACLITNCPRGGKRSD----
Acromyrmex_echinatior              ---------------MLKELIVFASLIFLSYACLITNCPRGGKRSD----
Vollenhovia_emeryi                 ---------------MLKEFVIFASLIFLSYACLITNCPRGGKRGD----
Solenopsis_invicta                 ---------------MLKELVVLASLIFLSYACLITNCPRGGKRGD----
Monomorium_sp                      ---------------MLKEFVVFASLIFFSYGCLITNCPRGGKRGD----
Wasmannia_auropunctata             ---------------MLKELVVLMSLIFLSYACLITNCPRGGKRNN----
Harpegnathos_saltator              ---------------MLRELVVFASLIFLSYACLITNCPRGGKRGD----
Dinoponera_quadriceps              ---------------MLRELVVLASLIFLSYACLITNCPHGGKRGD----
Cerapachys_biroi                   ---------------MLKKLVVFATLIFLSYACLITNCPRGGKRGD----
Linepithema_humile                 ---------------MLKELIVFANLILLSYACLITNCPRGGKRGD----
Pogonomyrmex_barbatus              ---------------MLKKIVVFASLIFLSYACLITNCPRGGKRSD----
Camponotus_floridanus              ---------------MLKQLVICASLIFLSHACLIVNCPRGGKRSD----
Pseudomasaris_vespoides            ---------------MAKELIVLIAIVVLASACLITNCPRGGKRG-----
Chyphotes_mellipes                 ---------------MAKELFVFAALLVVAYGCLITNCPRGGKRGGGKHG
Athalia_rosae                      ---------------MFQKTAIILLLVSSALGCLIINCPRGGKRNGA---
Neodiprion_lecontei                ---------------MFRKIVVILFLVSSTLGCLIINCPRGGKRDGG---
Cephus_cinctus                     ---------------MLKLCTVIMVFISISFACLITNCPRGGKRSGA---
Polistes_canadensis                ---------------MTKKILIFTSFIFISSACLITNCPRGGKRGD----
Polistes _metricus                 ---------------MTKKILIFTSFIFISSACLITNCPRGGKRGD----
Polistes_dominula                  ---------------MTKKILIFTSFIFITSACLITNCPRGGKRGD----
Mischocyttarus_flavitarsis         --------------MTKQILVFITSFILISSACLITNCPRGGKRVDNKV-
Corydalus_cornutus                 -----------MYPVVKYFVLVIVLVSVMIDSCLITNCPRGGKRNGSDRI
Onthophagus_taurus                 -----------MVKLIIFTLILCIIQDQLSHGCLITNCPRGGKRNGR-IN
Aleochara_curtula                  ---------MFSSKCCLLIVGLACVVLQAVDGCLITNCPRGGKRSGKYLP
```

```
Nicrophorus_vespilloides           --MHSPPSTMFILKSSALLLVLAVVFVGLCDSCLITNCPRGGKR---AMQ
Oryctes_borbonicus                 ------------MNSLLLIGVFCIAGDQLIEGCLITNCPRGGKRSGKMSH
Anoplophora_glabripennis           ----------MFKNILLLVSLLVVFGEIFVNCCLITNCPRGGKRSGKFGA
Leptinotarsa_decemlineata1         -----------MVRSVPLTIIYLFIFVMYVESCLITNCPKGGKRGGEFST
Leptinotarsa_decemlineata2         -----------MSKTVSLFTLFLSVFVIFVESCLITNCPIGGKRSGKFSL
Priacma_serrata                    --------------------AFFVLNSFLADSCLITNCPRGGKRS--RYN
Gyrinus_marinus                    ------------------------ALLDICDSCLITNCPRGGKRSGRSQH
Dendroctonus_ponderosae            ---MTSRSSSKIAKMISQILMFILLSNILVSGCLITNCPRGGKRSGKYGL
Meloe_violaceus                    ----------ATNKFLLVLIVLTIFTNYFINGCLITNCPRGGKRGNTRYL
Tribolium_castaneum                ------------MSTIITSIILLVLSESLVSGCLITNCPRGGKR--SKFA
Hypothenemus_hampei                -MVSKRNQLICTAGSNSTKLSIFMACLITVSGCLITNCPRGGKRSGKFLE
Agrilus_planipennis                ------------MYTNVCLVFVIVLCYSLVFGCLITNCPRGGRKRNGKME
Arachnocampa_luminosa              MNENFLMMNLNDKKNLKILFILVVINLNVINACYITNCPWGGKRSQPFLD
Tetranychus_urticae                -------------MLASFILLTFILNFHSSQACFITNCPPGGKRS--HGD
Scolopendra_subspinipes1           ------------------HWLLFFYVIGMTSSCFITNCPPGGKRSLGE--
Scolopendra_subspinipes2           ------------MRSSTCHWILYLSLVGLVSACYIINCIDNDGRKRSIDH
Strigamia_maritima                 ----------MKSTHFVNIFIYSVFIFIMADGCYITNCPPGGKRSGNEK-
Limulus_polyphemus                 ------------MHWTSFIMILGFCLAVTIKGCFITNCPPGGKRSIGTF-
Litopenaeus_vannamei               --------------MQISAVVAMALLLGSGTACFITNCPPGGKRSSPSAQ
Homarus_americanus                 --------------MQLGVVVVVMTVVVGSTTACFITNCPPGGKRSGPTAQ
Occasjapyx_japonicus               ---------MSSPVRIHPRLLALACVFCISSACFITNCPPGGKRSQQAHY
Catajapyx_aquilonaris              ----------MSPFRVHQTLLAVACIFCITSACFITNCPPGGKRSQ-SHY
Lithobius_forficatus               --------MASHAAQSSVFLVVLLCVFHITLACFITNCPPGGKRSLGDQK
Meinertellus_cundinamarcensis      ---------MKSSMSTPAALFALLFVVSITSSCFITNCPPGGKRAMGAPG
Machilis_hrabei                    -----MSADMTNSASSTFALFTLLLFVGCVTSCFITNCPPGGKRGLGSPG
Tricholepidion_gertschi            ---------MVARKNPQTVVICLMAAMSAATACFITNCPPGGKRSMGQVG
Atelura_formicaria1                -------------------------------ACFITNCPPGGKRSMGQMG
Atelura_formicaria2                ----------SLSKGILSVTIFFLAVISVTTACFITNCPPGGKRSMGQMG
Speleonectes_cf_tumulensis         --------MHLRPMDVLLSALCVLHVAGISTACFILDCPLMIRNRTVRTL
Anurida_maritima                   ----------MRKYHTSIICLVWLISVSAAFGCFITNCPPGGKR---SGP
Pogonognathellus_sp                ---------MIQFVTYNLWLGVLLVSFVAASGCFITNCPPGGKR---SGT
Folsomia_candida                   -----------MKTSLLFAFGIVMVESLLVSGCFITNCPPGGKR---SG-
Sminthurus_viridis                 ------------MINLNCGICLFLITLSVVTGCFITNCPPGGKR---SG-
Ephemera_danica                    ------MHRKYEMATKVYLTLTALIIVQVTTACFITNCPPGGKRSSAVVS
Triops_cancriformis                ---------------------------IHSAPCFITNCPPGGKRS-----
Eurylophella_sp                    ------------MVHRRGATLSAMLIVHVVSACFITNCPPGGKRS-----
Ixodes_scapularis                  ---------------MALSHMLLLAVVGGTSACFITNCPPGGKRSSEPS-
Ixodes_ricinus                     ---------------MALSHMLLLAVVGGTSACFITNCPPGGKRSSEPS-
Ladona_fulva                       ---MYSRQGMSMDQRQWLLVAVIFALLGLTSACFITNCPPGGKRSDVAFG
Menopon_gallinae                   ---------MYNIFQFQLRLGLLVATFGLVVSCLITNCPRGGKRGDNSLD
Pseudomallada_prasinus             ----------MNNNSLKATIVTFVIFTTLVHSCLITNCPRGGKRS-NLLD
Liposcelis_bostrychophila          --------------MNTFQFVVLYIFVNSVNSCLITNCPRGGKRDGSENN
Diaphorina_citri                   --------MYAMISRLLVVCLVGWYFFGSTWCCLINNCPTGGKRSALMAL
Apachyus_sp                        ------------MSFSLRFFAIFTLLLCLGTGCLITNCPKGGKR------
Pachypsylla_venusta                ------------MIPKIFFFLIFLCFLNFSFSCLISNCPKGGKR------
Cryptocercus_wrighti               --------------QLGTLATLAILLIGVCTACLITNCPKGGKR------
Orussus_abietinus                  --------------MKELLALYVLISAGYC--CLITNCPRGGKRSHHSES
                                                                   * * :*
                                   51                                             100
Calanus_finmarchicus1              QMVSFQAR----------EEVLACPS-NPAGLCYSPGLCC-VQGGCYADK
Calanus_finmarchicus3              QLVSFQAR----------EEVLACPS-NPAGLCYSPGLCC-IQGACYADM
Daphnia_pulex                      --------------PSSYLECAPCGP-AGKGTCLGANLCCGSHFGCFFKT
Daphnia_magna                      DPSSFQGVA--EERPLLSHQCASCGP-GGKGTCFGASLCCGSEFGCFFKT
Lepeophtheirus_salmonis            NLGFLNSSF-------QYKQCSSCGP-NNTGRCFGPRLCCSSEFGCFVYS
Caligus_rogercresseyi              -LFSPKNRR-------QLDECSPCGP-SNEGRCYGPNLCCGSTFGCFVYA
Dermatophagoides_farina            NLGSNHKKK-------IKIKCTRCGP-SLSGRCYGPNICCSPLTGCNVGG
Sarcoptes_scabiei                  GSSTQTRR-----------ECIRCGP-GLSGRCYGPSICCSPLFGCNVGG
Symphylella_vulgaris               --------GHSLSSSVALRQCSPCGP-GGAGRCFGKSACCVPGMGCFLGP
Tigriopus_californicus             --------SELLT--VASRKCPPCGP-LGMGQCFGPSMCCGQYIGCHLNT
Metaseiulus_occidentalis           ------AAFGLQLYSNEYRRCPPCGP-GSSGQCFGPGICCTPDY-CVLDP
Varroa_destructor                  ------TGFGLVQFSSDFRKCPPCGP-GSTGQCFGPNICCNSES-CLIDA
Gryllotalpa_sp                     E-------------ISSIRSCARCGL-NNAGHCYGPDICCAPELGCLIAT
Teleogryllus_commodus              E-------------ILPIRNCARCGP-NNTGHCYGPSTCCAEELGCLMGG
Haploembia_palaui                  --------------TESVRQCPRCGP-AKLGHCFGPTICCGHEIGCLINT
Aposthonia_japonica                --------------SESLRQCPRCGP-AKLGHCFGPTICCGHEIGCLINT
Zootermopsis_nevadensis            EL------------HT-IRQCARCGP-AKLGHCYGPAICCGPQIGCLVAT
Blattella_germanica                DS------------TVSIRQCARCGP-AKLGHCYGPAICCGPQIGCLIAT
Galloisiana_yuasai                 --------------KDSVRQCPRCGP-AKLGHCYGPAICCATQFGCLVAT
Locusta_migratoria_manilensis      --------------ASLQHRCAACGP-GGQGVCLGPHICCGPRMGCRLAS
Nilaparvata_lugens                 KKLAEEKMA----LAQVPLQCARCGP-EGEGYCMGPSICCGPQLGCLLAS
Halyomorpha_halys                  RDP----------------DCRRCGP-GLEGRCVGKTVCCGPRIGCLIGT
Homalodisca_vitripennis            NTLGTE--------IVSLVACLRCGP-GLQGRCLGPAVCCGPRLGCLVAT
Gynaikothrips_ficorum              TKG----------SIEELPFCTLCGPPELGGLCFGPTMCCSPTGGCELGE
Cotesia_vestalis                   ---PLLS------MPSLIKECSSCGP-GDQGQCFGPHICCGASIGCYIRS
Microplitis_demolitor              ---PLLS------MPSLIKECSSCGP-NDQGQCFGPHICCGATIGCYIKG
Fopius_arisanus                    ---PLLS------LHSLAAECPSCGP-GHQGQCFGPNICCGTTIGCFIGT
Diachasma_alloeum                  ---PLLS------LHSLAAECPSCGP-GHQGQCFGPNICCGTAIGCFIGT
```

```
Telenomus_podisi                    ---SLML------METSIRECTSCGR-DGSGRCFGPYICCGSTIGCFIGT
Nasonia_giraulti                    ---PTFL------LENIARECPACGR-EEQGRCFGPHICCSPSMGCLIGT
Nasonia_vitripenis                  ---PTFL------LENIARECPACGR-EEQGRCFGPHICCSPSMGCLIGT
Copidosoma_floridanum               ---PTFL------LENIARECPACGH-EEQGRCFGPHICCGPSMGCHIGT
Trichogramma_pretiosum              ---PTFF------LENIARECPVCGH-EEQGHCFGPHICCSPSMGCLIGT
Brachycistis_timberlakei            ---AVVS------LETIARECPSCGP-NRAGQCFGPHICCGPTIGCFIGT
Crioscolia_alcione                  ---IAPL------LESVARECPSCGP-NHSGQCFGPHICCGPNIGCFIRT
Chrysis_viridula                    ---SAVT------LDHLARECPSCGP-NRLGQCFGPHICCGPSIGCFIGT
Trachymyrmex_septentrionalis        ---VASS------LKTVIRECPSCGP-NHLGQCFGPYICCGPSIGCFIGT
Trachymyrmex_cornetzi               ---IASS------FKTVIRECPSCGP-NHLGQCFGPYICCGPSIGCFIGT
Atta_cephalotes                     ---VASS------LRTVIRECPSCGP-NHLGQCFGPYICCGPSIGCFIGT
Trachymyrmex_zeteki                 ---IASS------LRTVIRECPSCGP-NHLGQCFGPYICCGPSIGCFIGT
Acromyrmex_echinatior               ---IAS-------LKTVIRECPSCGP-NHLGQCFGPYICCGPSIGCFIGT
Vollenhovia_emeryi                  ---IAPS------LGTVVRECPSCGP-NRLGQCFGPYICCGPSIGCFIGT
Solenopsis_invicta                  ---IAPS------LGTVERECPSCGP-NRLGQCFGPHICCGPSIGCFIGT
Monomorium_sp                       ---IVPS------LGTVARECSSCGP-NRLGQCFGPHICCGPSIGCFIGT
Wasmannia_auropunctata              ---IASS------LETVARECPSCGP-NHQGQCFGPYICCGPSIGCFIGT
Harpegnathos_saltator               ---IIPS------LGTVTRECPPCGP-NHLGQCFGPHICCGPTIGCFIGT
Dinoponera_quadriceps               ---IAPS------LGVVTRECPLCGP-NHLGQCFGPHICCGPSIGCFIGT
Cerapachys_biroi                    ---IAPS------LGTVARECPSCGP-NHLGQCFGPHICCGPSIGCFIGT
Linepithema_humile                  ---IAP-------LETIARECPSCGP-NHLGQCFGPHICCGPSIGCFIGT
Pogonomyrmex_barbatus               ---LAP-------LGNVVRECPSCGP-NHLGQCFGPHICCGPNIGCFIGT
Camponotus_floridanus               ---IASF------LKTVTRECPTCGP-NHMGQCFGPRICCGPNIGCFIGT
Pseudomasaris_vespoides             ---DIAA------LESVVRECPACGP-NRSGQCFGPHICCGRTIGCYIGT
Chyphotes_mellipes                  EVVPAFE------DVRVTRECPSCGP-NRLGRCFGPRICCGPTIGCFIGT
Athalia_rosae                       ----LLP------LKNPIRECPACGP-EMQGQCFGPKICCGPSIGCFFGT
Neodiprion_lecontei                 ----FNP------FKYQVRECSTCGP-DRQGQCFGPKICCGPSIGCFFGT
Cephus_cinctus                      ----ITS------LENIVRECPSCGA-DKQGQCFGPRICCGPSLGCLIGT
Polistes_canadensis                 ----IEV------PESPIRECLPCGP-SQLGQCFGPNICCGPTFGCHIGT
Polistes_metricus                   ----IEV------PESPIRECLPCGP-SQLGQCFGPNICCGPTFGCHIGT
Polistes_dominula                   ----IEV------PES-IRECLPCGP-NQLGQCFGPKICCGPTFGCHIGT
Mischocyttarus_flavitarsis          --PLVTT------TEYPVKDCISCGP-NGQGQCFGSNICCGPTFGCHMGT
Corydalus_cornutus                  SNEQFIK------------QCISCGP-QRSGQCFGPSICCGS-FGCLVGT
Onthophagus_taurus                  SLDTNIK------------QCISCGP-ARSGQCFGPGICCGP-FGCLIGT
Aleochara_curtula                   DNNIKP--------------CIACGP-GHSGQCFGPDICCGP-FGCLLGT
Nicrophorus_vespilloides            DNTQIQ--------------CITCGP-GRSGQCFGPGICCGP-FGCLLGT
Oryctes_borbonicus                  LAAAN--------------QCVSCGP-GHTGQCFGPNICCGP-FGCLVGT
Anoplophora_glabripennis            LEANIK------------FQCVSCGP-GHSGQCFGPSICCGP-FGCLMGT
Leptinotarsa_decemlineata1          SESNIKTVSINLNNYKHILQCISCGP-GHTGKCFGPNICCGP-FGCLLGS
Leptinotarsa_decemlineata2          TESNIK-------------QCISCGP-GHTGQCFGPNICCGP-FGCLMGS
Priacma_serrata                     EEN--------------IKQCISCGP-AHSGQCFGPNICCGL-FGCLIGT
Gyrinus_marinus                     ENN--------------IKQCISCGP-GRSGQCFGSNICCGP-FGCLMGT
Dendroctonus_ponderosae             STSNNIKQVIQFSLI--QCICIPCGP-GGAGQCFGPSICCGP-FGCLLGT
Meloe_violaceus                     QKDTQIK------------QCINCGP-GNNGQCFGPQICCGP-FGCLFNT
Tribolium_castaneum                 ISENAVK------------PCVSCGP-GQSGQCFGPSICCGP-FGCLVGT
Hypothenemus_hampei                 AVVLFK--------------CIPCGP-GGTGQCFGPKICCGP-FGCLLDT
Agrilus_planipennis                 GDIQP-------------VCTSCGP-GLTGQCFGPNICCGQ-FGCLIGT
Arachnocampa_luminosa               SE--------------NAHQCRKCAS-G-LGMCFGPRICCGPDMGCLIDT
Tetranychus_urticae                 VD--------------HMRECLRCGP-DNRGHCVSPNICCGTEFGCLINN
Scolopendra_subspinipes1            HKRTLR-------------QCTACGP-GSQGRCFGPDLCCGEFFGCYLKT
Scolopendra_subspinipes2            QKKAIR-------------QCMSCGP-GMKGRCFGPDLCCIESFGCFTQT
Strigamia_maritima                  SGRGVR-------------QCTPCGP-GGIGRCYGPDICCGANVGCFVGT
Limulus_polyphemus                  ATHYTR-------------ECGQCGP-GGMGQCQGPDICCSDIFGCFIKT
Litopenaeus_vannamei                LG--------------SVRTCAQCGP-GLQGRCMGPDICCGPEIGCYMGT
Homarus_americanus                  LG--------------RTRTCTACGP-GLQGRCLGPEICCVLGIGCFLGT
Occasjapyx_japonicus                KPQAS-------------RQCAVCGP-NGQGRCFGPELCCGPEIGCYMRT
Catajapyx_aquilonaris               KPQAIRQ--------VSSAQCAVCGP-NGQGRCFGPELCCGPEIGCYLRT
Lithobius_forficatus                G---------------TTRQCTACGP-GGLGHCFGPNLCCGPQIGCFLHT
Meinertellus_cundinamarcensis       PRGVQR-------------QCPSCGP-HRSGRCFGPDLCCG-EFGCHLGG
Machilis_hrabei                     PRGEPR-------------QCPSCGP-RHSGRCFGPDLCCG-DFGCRLGA
Tricholepidion_gertschi             -THATR-------------ECTSCGP-AKLGRCYGPAICCGPQIGCLVGT
Atelura_formicaria1                 -LHVNK-------------QCTSCGP-ARTGRCYGPAICCGPRFGCLVGT
Atelura_formicaria2                 -LHANK-------------QCTSCGP-ARMGRCYGPAICCGPRFGCLVGT
Speleonectes_cf_tumulensis          KSSTSR-------------QCTSCGPDDLG-RCYSPNICCGPES------
Anurida_maritima                    LSTQAR-------------QCESCGPEGSGGRCYGPNICCSPGSGCLIGT
Pogonognathellus_sp                 TALTVR-------------QCERCGPPGMMGRCFGPQICCSPEFGCFFST
Folsomia_candida                    ADTKIR-------------QCEQCGPPGMNGLCYGPHICCSSELGCLFGT
Sminthurus_viridis                  IERKTR-------------QCEQCGPPGAGGRCYGPSICCSPELGCLIAT
Ephemera_danica                     ETQPKSVIRQFPITCSSYFVQCARCGPGLTGRCFGPRICCESSRGCLLGS
Triops_cancriformis                 ----------FPMSSSQHFL------------------------------
Eurylophella_sp                     -----------SSITTVNARQCSRCGPGLSGRCFGARICCDSRRGCMLAS
Ixodes_scapularis                   --------PARLCPRCGPAGRGVCYSADVCCAGSMCILNDALATLSCRAE
Ixodes_ricinus                      --------PARLVS-----------------------SQFRSSLTVSWC
Ladona_fulva                        R------IPLRQVMVVSSVLSFKCSRCGFGGRCFGPAICCSLKTGCILAE
Menopon_gallinae                    KTSS-YP---------DTNYCIPCGP-DDQGVCLGKNVCC-SREGCTTRS
Pseudomallada_prasinus              S-------------------------------------------------
Liposcelis_bostrychophila           LIEAPRS---------DIKTCTPCGP-NNQGVCIGRDICCGKHLGCLISS
```

```
Diaphorina_citri                    EERRQSHQKR--AKLWNDRNIISCNAWGAEGVCVGPLCCDPSRSEGDPGS
Apachyus_sp                         ---------------------------TSITVSFILKF------------
Pachypsylla_venusta                 ---------------------------FDKKFPQNKKEIFDKSKVSTLYI
Cryptocercus_wrighti                ------------------------AGSHSQDSHPIRQVFLGYLTTLYQLL
Orussus_abietinus                   PETTIEK--------------FPLAGLYDQSRYPSLHVYNDQFIDSYARS

                                    101                                            150
Calanus_finmarchicus1               GCLPQMEESRENRVEPRLNKRDL---------RFLYGWAPAHEKMFGQTG
Calanus_finmarchicus3               --------------------------------------------------
Daphnia_pulex                       ----------------------------------------------EETN
Daphnia_magna                       ----------------------------------------------NETN
Lepeophtheirus_salmonis             NDIAKN-PCQTEAYDPVPCQ-----------------NNVKSCSSVLKNG
Caligus_rogercresseyi               KSLQHQNPCLTEARLPLPCR-----------------NKGGSCSGILENG
Dermatophagoides_farina             ---FTAARCSLEAYHPILCT-----------------NPGTVCGPNGK-G
Sarcoptes_scabiei                   ---FASSRCALEAFNPMLCS-----------------NPGSACGPNGK-G
Symphylella_vulgaris                AHT---ATCRLEHSYP------------------VPCDNGTPACGENGNG
Tigriopus_californicus              PDT---QVCKTENSNP------------------IPCDNDVPRCQSVRRG
Metaseiulus_occidentalis            VG---LSTCKSEALKM------------------TPCNINRPRCGAENKG
Varroa_destructor                   GDSPHLRSCKREALKL------------------KPCTNTGMRCGSENKG
Gryllotalpa_sp                      PETVPCQEESQSPD------------------PCIDTDLGPACHNGK--G
Teleogryllus_commodus               PEAKPCEAEALSPD------------------PCVEPALGPRCFGGQ--G
Haploembia_palaui                   PDTLVCLKEHLSPD------------------PCVGPTATNCGNRK---G
Aposthonia_japonica                 PDTLVCLKEHLSPD------------------PCVGPSASNCGNRK---G
Zootermopsis_nevadensis             PDTARCLSEAASPV------------------PCTAPTGAQCGEGKFA-G
Blattella_germanica                 PETTRCLSEAASPS------------------PCTTPSGAPCGSGKNA-G
Galloisiana_yuasai                  ADTAICQGESLYPE------------------PCAN-TMPASSCRGEA-G
Locusta_migratoria_manilensis       -ADADSAACRAAPP------------------CPLDSPEMRCAAGRGR--
Nilaparvata_lugens                  PATLNQCAGSVLPP------------------QSPNSPAPTCYMPDGY-G
Halyomorpha_halys                   PAVLSQCAAEPMQL------------------RGGQR--------CSI-G
Homalodisca_vitripennis             PGIVRQCAGAAEGP------------------SPHGKP---CITPSGL-G
Gynaikothrips_ficorum               PHVASCLGVHTVPPLCRLPPQSMPLNGPYSPSAPSQTRATSLVTTCGS-G
Cotesia_vestalis                    PETYKCRKE-SLHSH--------------------PCVSGFAMCRDNT-A
Microplitis_demolitor               PETYKCRKE-SLYSH--------------------PCVSGFAMCRDNT-A
Fopius_arisanus                     PETYKCRTE-SLYSR--------------------PCIAGFAMCRDNT-G
Diachasma_alloeum                   PETYKCRTE-SLYSR--------------------PCIAGFAMCRDNT-G
Telenomus_podisi                    PETYKCRKE-SLYSK--------------------PCIAGFAMCRGNK-G
Nasonia_giraulti                    PETLRCRKE-SLYSR--------------------PCVAGFAMCQGNS-G
Nasonia_vitripenis                  PETLRCRKE-SLYSR--------------------PCVAGFAMCQGNS-G
Copidosoma_floridanum               LETLRCRKE-SLYSR--------------------PCVAGFAMCQGNS-G
Trichogramma_pretiosum              SETLSCRKE-SLYSR--------------------PCVAGFAMCQNNN-G
Brachycistis_timberlakei            PETYRCRKE-SLFSR--------------------PCIAGYAMCRGNT-A
Crioscolia_alcione                  PETYRCRKE-SLFS------------------------------------
Chrysis_viridula                    PETYKCRKE-SLFSR--------------------PCVAGYAMCRGNS-A
Trachymyrmex_septentrionalis        PETFQCRKE-SLYTR--------------------PCIAGYAMCRGNT-A
Trachymyrmex_cornetzi               PETFRCRKE-SLYTR--------------------PCIAGYAMCRGNT-A
Atta_cephalotes                     PETFRCRKE-SLYTR--------------------PCIAGYAMCRGNT-A
Trachymyrmex_zeteki                 PETSRCRKE-SLYTR--------------------PCIAGYAMCRGNT-A
Acromyrmex_echinatior               PETFRCRKE-SLYTR--------------------PCIAGYAMCRGKT-A
Vollenhovia_emeryi                  PETYRCRKE-SLYTR--------------------PCVAGYAMCRGNT-A
Solenopsis_invicta                  PETYRCRKE-SLYTR--------------------PCIAGYAMCHGNT-A
Monomorium_sp                       PETYRCRKE-SLYTR--------------------PCIAGYAMCRGNT-A
Wasmannia_auropunctata              PETYRCRKE-SLYTR--------------------PCIAGYAMCRGNT-A
Harpegnathos_saltator               PETYRCRKE-SPYAR--------------------PCIAGYAMCRGNT-A
Dinoponera_quadriceps               PETYRCRKE-SLYAR--------------------PCIAGYAMCRGNT-A
Cerapachys_biroi                    SETYRCRKE-SLYTR--------------------PCVAGYAMCRGNT-A
Linepithema_humile                  PETYRCRKE-SLYTR--------------------PCIAGYAMCRGKT-A
Pogonomyrmex_barbatus               SETYRCRKE-SLYIR--------------------PCIAGYAMCRGNT-A
Camponotus_floridanus               PETYRCRKE-SLYTK--------------------PCIAGYAMCRGNT-A
Pseudomasaris_vespoides             PETYRCRKE-SLYSR--------------------PCIAGYAMCRGNT-A
Chyphotes_mellipes                  PETYGCRGEGSPFSK--------------------PCVAGYAMCRRNT-A
Athalia_rosae                       AETHNCRKE-SLYSR--------------------PCIAGFAMCRGNT-G
Neodiprion_lecontei                 AETHKCRKE-SLYSR--------------------PCTAGFAMCRGNT-G
Cephus_cinctus                      PETHKCRKE-SLFSR--------------------PCIAGYAMCRGNT-G
Polistes_canadensis                 SQTQKCRKE-QFDSS--------------------PCMAGFAMCNGNK-G
Polistes_metricus                   SQTQKCRKE-QFDSS--------------------PCMAGFAMCNGNK-G
Polistes_dominula                   SQTQKCRKE-QFDTS--------------------PCLAGFSMCNGNK-G
Mischocyttarus_flavitarsis          SYTKKCRKE-FSDQI--------------------SCLSGFAMCSGNV-G
Corydalus_cornutus                  PETVRCARDGGFHEPE-------------------PCIAGQASCRGDS-G
Onthophagus_taurus                  PDTLKCLKEGQFHENE-------------------PCIAGNSSCRRNS-G
Aleochara_curtula                   HETARCRRDGNFLEPE-------------------PCIAGNASCRRNS-G
Nicrophorus_vespilloides            QETVKCQREGFFHGRE-------------------PCIAGSAPCRKNT-G
Oryctes_borbonicus                  AETLRCQKDGFFHEAE-------------------PCIAGNSPCRRNT-G
Anoplophora_glabripennis            PETLRCQREGFFHERE-------------------PCIAGGASCRKNT-G
Leptinotarsa_decemlineata1          PETERCHKEGLFQETE-------------------PCIAGFSTCRKNT-G
Leptinotarsa_decemlineata2          HETFGCQRG-GFHESE-------------------PCIAGFSSCRKNT-G
Priacma_serrata                     SETIRCQREGFFHERE-------------------PCIAGNAFCRKKH-R
Gyrinus_marinus                     SDTIRCHREGYFQDSE-------------------PC-------------
```

```
Dendroctonus_ponderosae           PETIRCQREGMFHESE-------------------PCIAGNSN-------
Meloe_violaceus                   IDTVRCQKDGNFHQHE-------------------PCIAGFMNCHHYN-G
Tribolium_castaneum               PETLRCQREGFFHERE-------------------PCIAGSAPCRKNT-G
Hypothenemus_hampei               PDSIHCQNEGMFYDLE-------------------PCTAGTMNCRKNT-G
Agrilus_planipennis               SDSLKCKYEGLFNGPE-------------------PCIAGNSSCRWKH-G
Arachnocampa_luminosa             KETSVCQLEDLKSNVPCQP-------------------YGKICDKVEF-G
Tetranychus_urticae               HYSSPCRAENLIP-TPCKI-------------------PGKLCSSGE--G
Scolopendra_subspinipes1          RESAICRFENLSPIPCDND----------------VLSCGP------DGA
Scolopendra_subspinipes2          PESAVCHLENLSPVPCDND----------------TPSCG-------LGA
Strigamia_maritima                RESAICRLENLYSLPCQNE----------------GRACG-------TDG
Limulus_polyphemus                RESVICRYENLQVIPCNKK----------------GKICETV-----PHG
Litopenaeus_vannamei              REAFLCRSENLVPVTCSNDD---------------LKACGRQ-----REG
Homarus_americanus                REARMCHAENLVPVTCANRD---------------LKSCGRM-----QEG
Occasjapyx_japonicus              RESSSCRAENLFPVQCENR----------------ARPCGSE-----RSG
Catajapyx_aquilonaris             REASACRAENLFPVQCENK----------------ARPCGSE-----RSG
Lithobius_forficatus              R------------------------------------------------G
Meinertellus_cundinamarcensis     RD-PVCRGEMSAPGLCTNP-----------------GRPCG-------VG
Machilis_hrabei                   RD-ASCRREMSAPGLCINQ-----------------GRPCG-------VG
Tricholepidion_gertschi           REAAVCQTENFYPVPCANR-----------------GGQCGG-----ESG
Atelura_formicaria1               RDTAVCQTENFTPVPCGNR-----------------GPSCG------ESG
Atelura_formicaria2               RDTAVCQ-------------------------------------------
Speleonectes_cf_tumulensis        --------------------------------------------------
Anurida_maritima                  PETVACRSEARFS-TPCLNP---------------GISCSG-SGSLSIKG
Pogonognathellus_sp               AETFVCQLENRFP-TPCHNP---------------GSSCSGNGDTGLING
Folsomia_candida                  SETLTCFQENLMYNTPCQNP---------------GESCSGNDATNPING
Sminthurus_viridis                PETYSCQLENRHT-TLCQNP---------------GATCAGPNGD-ISGG
Ephemera_danica                   PLEVFMLPCLVESLQPTP------------------CRNQASNCGLTGDE
Triops_cancriformis               --------------------------------------------------
Eurylophella_sp                   STEPSLLPCLAESMQTTP------------------CRNRAASCGQSGAG
Ixodes_scapularis                 ALHGVACHVPGKRCGTDGR-----------------CAIRGYCCGPDGCT
Ixodes_ricinus                    SLLGVDFQRMSFYRMPDY--------------------------------
Ladona_fulva                      KSNNLPLLRPCAIEAGLPG-----------------ACISGSKRCGNSGG
Menopon_gallinae                  RYTDPCRSIQNTRIS---------------------CLSEVNVQTCGDKG
Pseudomallada_prasinus            --------------------------------------------------
Liposcelis_bostrychophila         PSTKICRRVREFPES---------------------CLSSFN--PCGNGG
Diaphorina_citri                  KCGVDYKTCGSSRE-----------------------VMEEYALCLSKGL
Apachyus_sp                       --------------------------------------------------
Pachypsylla_venusta               GD------------------------------------------------
Cryptocercus_wrighti              SL------------------------------------------------
Orussus_abietinus                 ADTTKSATEACWENKEFL------------------NDNGNNQRKKMKLT

                                  151                                            200
Calanus_finmarchicus1             FSIPCIGPYCNGNEKMIMRKEKQSQEKKSDDKVLDGSSNYDSLVYNENLS
Calanus_finmarchicus3             --------------------------------------------------
Daphnia_pulex                     VCLLTNLKSTQICNQHFWKTDLKSASCSLNGDKIDGICVADLLCCSLGNL
Daphnia_magna                     ICLLTNLKSSRSCDERFWQIYFKSAPCSLNGDKLDGICVADRLCCSLGQC
Lepeophtheirus_salmonis           QCVFDNYCCNSSGTCRFVEEELICSVSDDKKNYEKEKELVDDLQKNMIYI
Caligus_rogercresseyi             QCVFENYCCSPSGTCRFVDESICSNSTVPQLGDS---DFMEDFRRRLFLA
Dermatophagoides_farina           VCALNATCCTNG---KYFICFFYCKKN-----------------------
Sarcoptes_scabiei                 ICAINSTCCTDS---KI---------------------------------
Symphylella_vulgaris              RCAVEGLCCTGDG-CFYEDTCRDS--------SLLTSSLHHSGSTLEDET
Tigriopus_californicus            FCATNGFCCNDQGECTPEEKCLVENLSDFPSPFILRRHLSSSASLASRPT
Metaseiulus_occidentalis          YCALNRLCCTSDS-CTLDESCSSAKDRDILRESLMLN-------------
Varroa_destructor                 HCALNRFCCTS---------------------------------------
Gryllotalpa_sp                    HCGANNICCTQDS-CYSDQTCQFGFPVE----------------------
Teleogryllus_commodus             FCATSTLCCTQVS-CHTDPSCQFAVAGHKSVGATDLALPPHRSYVYSLLN
Haploembia_palaui                 KCAADGVCCTPES-CEID--------------------------------
Aposthonia_japonica               KCAADSICCTPDS-CEIDASCRFGS--PQHFRNVGIYPQYVE--------
Zootermopsis_nevadensis           RCTANGVCCTHES-CHIDITCQLTT--SDAPELIDVSADQTNPLYSLYS-
Blattella_germanica               RCAANGVCCTHES-CHIDVSCHPSA--TDIAIGNTDQLNSFYNLYNVISS
Galloisiana_yuasai                KCAANGICCTSES-CHVDMSCRLTSRGEDDVIQFPDLSSDAKGVFSLFSN
Locusta_migratoria_manilensis     -CSAPGVCCSQDS-CHIDPSCVADTTEPADRYALDIFAVNDGDRDDMRI-
Nilaparvata_lugens                VCSTDGVCCNSES-CRIDPTCEVDPDHNVCNVWTEPKNMAL---------
Halyomorpha_halys                 VCLADGVCCSPDG-CKMDMSCEVEQSVDVCSLAEQQVY------------
Homalodisca_vitripennis           VCATDGVCCNSGG-LYFPS----PQIRHIYRNFEISQY------------
Gynaikothrips_ficorum             ICAANGLCCTSDW-CRADDRCLSSASQSNLVIPDHNLPANTRQEPQQAEF
Cotesia_vestalis                  RCAANGICCSQES-CFLDSSCKIGDEFL------SDRKIDQEFSRLIGEN
Microplitis_demolitor             RCAANGICCSQES-CHVDPSCRVGDEYL------SERKISQEFSRLIADN
Fopius_arisanus                   RCAANGICCSQEN-CHADSSCRVGDEFS-------DRKIVQEYSRLSPEN
Diachasma_alloeum                 RCAANGICCSQEN-CHVDSSCRISDEFN-------DRKIGQEYSRLSPEN
Telenomus_podisi                  RCAANGICCSQEA-CYAD--------------------------------
Nasonia_giraulti                  RCAANGICCSQES-CFIDSACKLVDETG------NDRKIGAEFGAFLLEN
Nasonia_vitripenis                RCAANGICCSQES-CFIDSACKLVDETG------NDRKIGAEFGAFLLEN
Copidosoma_floridanum             RCAANGICCSQES-CFTDSACKLVDESG------NDRKIGAEFGAFLLEN
Trichogramma_pretiosum            RCAANGICCSQES-CFIDPACKLVDEN-------NDRKIGAEFGAFLLEN
Brachycistis_timberlakei          RCAANGICCSQES-CSMDAMCKIPDALR------HDRKFD-SDVSSILSE
Crioscolia_alcione                --------------------------------------------------
```

```
Chrysis_viridula                    RCAANGICCSQET-CHMDSTCRISDVDT------RDRKEEGVDVAAVLSD
Trachymyrmex_septentrionalis        RCASNGICCSQAS-CHMDTSCKISDVG-------NDRKLD-DNVNVILPG
Trachymyrmex_cornetzi               RCASNGICCSQAS-CHMDTSCKISDVG-------NDRKLD-DNVNVILPG
Atta_cephalotes                     RCASNGICCSQAF-CHMDTSCKISDVD-------NDRKLD-DNVNVILPG
Trachymyrmex_zeteki                 RCASNGICCSQAS-CHMDTSCKISDVG-------NDRKLD-DNVNVILPG
Acromyrmex_echinatior               RCASDGICCSQAS-CHMDTSCKISDVG-------NDRNLD-DNVNVILPG
Vollenhovia_emeryi                  RCASSGICCSQES-CHMDTSCRIPDVVG------NDKKLD-ANLNVILPG
Solenopsis_invicta                  RCASNGICCSQES-CHMDTSCRISDVVG------NDRKLD-ANLNVIFPD
Monomorium_sp                       RCALNGICCSQES-CHMDTSCKISNVVG------NNQKLD-DNLNVILSG
Wasmannia_auropunctata              RCASNGICCSQES-CNMDTSCKISDVG-------NDRKLD-ANLNVILSG
Harpegnathos_saltator               RCATNGICCSQDS-CHMDTSCRISDVVS------NDRKMD-ADLSAILSS
Dinoponera_quadriceps               RCATNGICCSQES-CHMDTSCRISDVVG------NDRKMD-TDLNVILSS
Cerapachys_biroi                    RCATNGICCSQES-CHMDTSCRISDVG-------NDRKID-ADLNVMFPG
Linepithema_humile                  RCATNGICCSQES-CHMDTSCRISNIVD------NNRKID-TDLNMIIPG
Pogonomyrmex_barbatus               RCASNGICCSQES-CYIDSSCRISNAVG------SDRKLD-ADLNVILPG
Camponotus_floridanus               RCAANGICCSQ---------------------------------------
Pseudomasaris_vespoides             RCAANGICCSQES-CFIDNTCRIPDIMA------KDQKID-SDINMIFGE
Chyphotes_mellipes                  RCAASGICCSQES-CHIDASCRLSETSDGGGGNGHERRFDSDLSAILSEN
Athalia_rosae                       RCAAGGICCSQES-CHADSNCKVTDDFNAN-NQGISFDLNTLFSENTSLN
Neodiprion_lecontei                 RCAANGICCSQES-CYVDPNCKVRSDIIQMSKQVTSFDMDKIYSESNSLN
Cephus_cinctus                      RCAANGICCTQES-CYVDDTCRITDDVNNDHRIIS--ELNGFLNEGNVLA
Polistes_canadensis                 RCGAGGICCSQDS-CFIDPSCRFSTVDSS-FRRSIDSDLKAIASEIEILK
Polistes_metricus                   RCGAGGICCSQDS-CFIDPSCRFSTVDSP-FRRSIDSDLKAIASEIEILK
Polistes_dominula                   RCGAAGICCSQNS-CFIDPSCRFPTFDDWPGRQKIDSDLKTIASEILKEE
Mischocyttarus_flavitarsis          RCAAIGICCSQES-CFLDPICRLSDNLPVGRKFNT---------------
Corydalus_cornutus                  RCAADGICCTQES-CHTDSSCAFDEERHDRSHNGRPAIPIESIFSVLSPY
Onthophagus_taurus                  RCASEGICCTQGN-F-----------------------------------
Aleochara_curtula                   RCAAEGICCSQ---------------------------------------
Nicrophorus_vespilloides            RCAAEGLCCSQGI-IIQLLLLLHLRLTHRLHHPMMI--------------
Oryctes_borbonicus                  RCATDGVCCSQGI-RYIDS-------------------------------
Anoplophora_glabripennis            RCAIDGICCSQGN-NSGFLKKLKNNLFLSLQILVMSTNNARWKTKAGFFP
Leptinotarsa_decemlineata1          RCAAEKICCTQGI-WTFFLVSN----------------------------
Leptinotarsa_decemlineata2          RCATENICCTQGI-FKIRSYT-----------------------------
Priacma_serrata                     TMCQRGHMLHARL-LPHRQNYAHLTTNKQN--------------------
Gyrinus_marinus                     --------------------------------------------------
Dendroctonus_ponderosae             --------------------------------------------------
Meloe_violaceus                     RCATDGICCNQDS-CHIDKTCSITTTIGFIENEKTRNFLETSPADLYNFI
Tribolium_castaneum                 RCAFDGICCSQDS-CHADKSCASD---------------DKSPIDLYTLI
Hypothenemus_hampei                 RCATNGICCSQGK-RTVLVNSSSVYN------------------------
Agrilus_planipennis                 RCATQGVCCNQGK-CFTSFYLLLNVLDISPYQGIIRRKVLLNKNLTNVGT
Arachnocampa_luminosa               RCATSNLCCNPEH-CLEDSTCVSEENDYSEDYKEIDTKLLKALKRLISKK
Tetranychus_urticae                 VCTSNGICCNSDG-CFNDSTCENDK--------------LSNMQRIFTSD
Scolopendra_subspinipes1            KCAADGLCCSSDQ-CKMEERCR----------------------------
Scolopendra_subspinipes2            NCAVDGLCCNTSQ-CKRDERC-----------------------------
Strigamia_maritima                  TCSADGFCCSTDQ-CKADESCRGKVHHTN---------------NLQRVL
Limulus_polyphemus                  YCAAPGICCSAIQ-CAVDDECPTGVLDKG---------------VAYKYD
Litopenaeus_vannamei                RCASSGVCCTEVK-CEFDINCVIEG-------------------VDRHRY
Homarus_americanus                  RCAAAGLCCTEMK-CEFDSSCTVEGREERVGKQ----------RAERQHL
Occasjapyx_japonicus                KCAAVGICCTEDS--CTSDQTCRASPGKEAVAAAVLPRGPSRPQLPSDLV
Catajapyx_aquilonaris               KCAAMGLCCTEAD-SCTSDSACRGAPSKEVVAASRGQR---PQQLPSDLV
Lithobius_forficatus                KCHLPF--------------------------------------------
Meinertellus_cundinamarcensis       KCAAQGVCCSETA-CALDSMCLDSWPS-------RNVVQ-----MNSGLA
Machilis_hrabei                     VCAAQGVCCSETS-CALDASCMDSWSP-------RQLVP-----VNNGLS
Tricholepidion_gertschi             RCAATGVCCTEES-CSIDPSCHVTPQETILTPVSRQMYP-----INSAMN
Atelura_formicaria1                 RCAANGVCCTEET-CTIDPSCRISNED---ISSSRQMFP-----ITNSVS
Atelura_formicaria2                 --------------------------------------------------
Speleonectes_cf_tumulensis          --------------------------------------------AATSAP
Anurida_maritima                    QCTGDGICCSSET-CTHDENCWERSMT---------------SGTGDSQE
Pogonognathellus_sp                 QCG-----------------------------------------------
Folsomia_candida                    QCATLGVCCSSDT-CTIDDNCHKKDEQQNVSGHSRSTVAMDGSHTKEHAT
Sminthurus_viridis                  QCGAEGICCNSET-CTMDETCHDEASVTRES------------VAKLSGL
Ephemera_danica                     LCAAPGICCSQGK-------------------------------------
Triops_cancriformis                 --------------------------------------------------
Eurylophella_sp                     QCASQGICCLQDSCHADVKCHENYD-------------------------
Ixodes_scapularis                   KDSSCSGGVPTDQFGSAVDILEYGMSER----------------------
Ixodes_ricinus                      --------------------------------------------------
Ladona_fulva                        RCASDGICCNDGKRN-----------------------------------
Menopon_gallinae                    RCKAKFICCSRDQCVMDLSCKSMPPAKYRDVNE-----------------
Pseudomallada_prasinus              ----------------KISTDSIKP-------------------------
Liposcelis_bostrychophila           LCVARNVCCFKNLCRIDDTCKTIPYVDLGYRLGTSDILNRLSSYSVDYPL
Diaphorina_citri                    ACDLNTLKCCNMYAIKVPTEVKLQLHQ-----------------------
Apachyus_sp                         --------------------------------------------------
Pachypsylla_venusta                 --------------------------------------------------
Cryptocercus_wrighti                --------------------------------------------------
Orussus_abietinus                   ELFKRRRCKSKFINDTSIPSPNILI-------------------------
```

```
                                   201                                            250
Calanus_finmarchicus1              NQRCNWMDLYLAR-------------------------------------
Calanus_finmarchicus3              --------------------------------------------------
Daphnia_pulex                      PQDDL---------------------------------------------
Daphnia_magna                      KQNFAC--------------------------------------------
Lepeophtheirus_salmonis            RRQAYLGNGVPRLQQLNINDLEQ---------------------------
Caligus_rogercresseyi              RELHSL--------------------------------------------
Dermatophagoides_farina            --------------------------------------------------
Sarcoptes_scabiei                  --------------------------------------------------
Symphylella_vulgaris               TQ------------------------------------------------
Tigriopus_californicus             VEKAMITIPQPNAKAYQEALNLMQIIPKSNSALNKYLSRHGDVK------
Metaseiulus_occidentalis           --------------------------------------------------
Varroa_destructor                  --------------------------------------------------
Gryllotalpa_sp                     --------------------------------------------------
Teleogryllus_commodus              ALAKNQDHNPQNNVE-----------------------------------
Haploembia_palaui                  --------------------------------------------------
Aposthonia_japonica                --------------------------------------------------
Zootermopsis_nevadensis            SYQQENPGLGLSE-------------------------------------
Blattella_germanica                AYQQENP-------------------------------------------
Galloisiana_yuasai                 LGSYEPDHQAYRLPSNVERD------------------------------
Locusta_migratoria_manilensis      --------------------------------------------------
Nilaparvata_lugens                 --------------------------------------------------
Halyomorpha_halys                  --------------------------------------------------
Homalodisca_vitripennis            --------------------------------------------------
Gynaikothrips_ficorum              NDWHS---------------------------------------------
Cotesia_vestalis                   DQMIN---------------------------------------------
Microplitis_demolitor              DQLIN---------------------------------------------
Fopius_arisanus                    E-------------------------------------------------
Diachasma_alloeum                  EQ------------------------------------------------
Telenomus_podisi                   --------------------------------------------------
Nasonia_giraulti                   AGTNEHIL------------------------------------------
Nasonia_vitripenis                 AGTNEHIL------------------------------------------
Copidosoma_floridanum              AGANERILR-----------------------------------------
Trichogramma_pretiosum             SGRNEHIL------------------------------------------
Brachycistis_timberlakei           SEISNEII------------------------------------------
Crioscolia_alcione                 --------------------------------------------------
Chrysis_viridula                   NELSADSVQ-----------------------------------------
Trachymyrmex_septentrionalis       NEVSNEILQ-----------------------------------------
Trachymyrmex_cornetzi              NEVSNEILQ-----------------------------------------
Atta_cephalotes                    -EVSNEILQ-----------------------------------------
Trachymyrmex_zeteki                NEVSNEILQ-----------------------------------------
Acromyrmex_echinatior              NEVSNEILQ-----------------------------------------
Vollenhovia_emeryi                 SEVSSEILQ-----------------------------------------
Solenopsis_invicta                 NEVSSDILQ-----------------------------------------
Monomorium_sp                      NEISSEIPQ-----------------------------------------
Wasmannia_auropunctata             NEVSSDLF------------------------------------------
Harpegnathos_saltator              NEASHEIIQ-----------------------------------------
Dinoponera_quadriceps              NEVSHEILQ-----------------------------------------
Cerapachys_biroi                   NEASSETFP-----------------------------------------
Linepithema_humile                 NEVSSEILQ-----------------------------------------
Pogonomyrmex_barbatus              NEVSNEMFQ-----------------------------------------
Camponotus_floridanus              --------------------------------------------------
Pseudomasaris_vespoides            NEISNELTQ-----------------------------------------
Chyphotes_mellipes                 VGTSVEMVR-----------------------------------------
Athalia_rosae                      DQ------------------------------------------------
Neodiprion_lecontei                DE------------------------------------------------
Cephus_cinctus                     DHNQ----------------------------------------------
Polistes_canadensis                EEAK----------------------------------------------
Polistes_metricus                  EEAK----------------------------------------------
Polistes_dominula                  AM------------------------------------------------
Mischocyttarus_flavitarsis         --------------------------------------------------
Corydalus_cornutus                 RS------------------------------------------------
Onthophagus_taurus                 --------------------------------------------------
Aleochara_curtula                  --------------------------------------------------
Nicrophorus_vespilloides           --------------------------------------------------
Oryctes_borbonicus                 --------------------------------------------------
Anoplophora_glabripennis           ITLWEWICII----------------------------------------
Leptinotarsa_decemlineata1         --------------------------------------------------
Leptinotarsa_decemlineata2         --------------------------------------------------
Priacma_serrata                    --------------------------------------------------
Gyrinus_marinus                    --------------------------------------------------
Dendroctonus_ponderosae            --------------------------------------------------
Meloe_violaceus                    NYQSELNSE-----------------------------------------
Tribolium_castaneum                NYQAELAGDK----------------------------------------
Hypothenemus_hampei                --------------------------------------------------
Agrilus_planipennis                LNIE----------------------------------------------
Arachnocampa_luminosa              RQKNENYKSHNIDNYQS---------------------------------
```

```
Tetranychus_urticae                 --------------------------------------------------
Scolopendra_subspinipes1            --------------------------------------------------
Scolopendra_subspinipes2            --------------------------------------------------
Strigamia_maritima                  DGEIDLNDVMGPQR------------------------------------
Limulus_polyphemus                  HLRFVLLPWNMNGKLPD---------------------------------
Litopenaeus_vannamei                SLLDTLTEAQWRI-------------------------------------
Homarus_americanus                  TFLSSLPEDQWNL-------------------------------------
Occasjapyx_japonicus                SFLEEVVDNMAPASGSEV--------------------------------
Catajapyx_aquilonaris               SFLEEVVENIAPASAPEV--------------------------------
Lithobius_forficatus                --------------------------------------------------
Meinertellus_cundinamarcensis       SVLENMLEEVPSENGDDKK-------------------------------
Machilis_hrabei                     SVLENMLEDIPMESGDDRR-------------------------------
Tricholepidion_gertschi             NYLEEAVDALPSHVILENRR------------------------------
Atelura_formicaria1                 MYLEELNDDLPARVGSDRR-------------------------------
Atelura_formicaria2                 --------------------------------------------------
Speleonectes_cf_tumulensis          KNRRLPVRE---LLPCSL--------------------------------
Anurida_maritima                    FNQDLPLREDRFLIPLPATHEKFRSFPISQIIRVGRRKNQFEDLRNHLLH
Pogonognathellus_sp                 --------------------------------------------------
Folsomia_candida                    TMFQNPLDRWIPRRFGLGRPTGQRKHVRIGVKPDAQILAPENSLMDRLVI
Sminthurus_viridis                  YHHRYPLDVVFQRFVDGQHSQHHRLPARLPLPTQSSSSSNDY--------
Ephemera_danica                     --------------------------------------------------
Triops_cancriformis                 --------------------------------------------------
Eurylophella_sp                     --------------------------------------------------
Ixodes_scapularis                   --------------------------------------------------
Ixodes_ricinus                      --------------------------------------------------
Ladona_fulva                        --------------------------------------------------
Menopon_gallinae                    --------------------------------------------------
Pseudomallada_prasinus              --------------------------------------------------
Liposcelis_bostrychophila           ELPSNMPIGSTSLDEVWGSKEDSIVPNYNFKIEAS---------------
Diaphorina_citri                    --------------------------------------------------
Apachyus_sp                         --------------------------------------------------
Pachypsylla_venusta                 --------------------------------------------------
Cryptocercus_wrighti                --------------------------------------------------
Orussus_abietinus                   --------------------------------------------------

                                    251
Calanus_finmarchicus1               -------------------
Calanus_finmarchicus3               -------------------
Daphnia_pulex                       -------------------
Daphnia_magna                       -------------------
Lepeophtheirus_salmonis             -------------------
Caligus_rogercresseyi               -------------------
Dermatophagoides_farina             -------------------
Sarcoptes_scabiei                   -------------------
Symphylella_vulgaris                -------------------
Tigriopus_californicus              -------------------
Metaseiulus_occidentalis            -------------------
Varroa_destructor                   -------------------
Gryllotalpa_sp                      -------------------
Teleogryllus_commodus               -------------------
Haploembia_palaui                   -------------------
Aposthonia_japonica                 -------------------
Zootermopsis_nevadensis             -------------------
Blattella_germanica                 -------------------
Galloisiana_yuasai                  -------------------
Locusta_migratoria_manilensis       -------------------
Nilaparvata_lugens                  -------------------
Halyomorpha_halys                   -------------------
Homalodisca_vitripennis             -------------------
Gynaikothrips_ficorum               -------------------
Cotesia_vestalis                    -------------------
Microplitis_demolitor               -------------------
Fopius_arisanus                     -------------------
Diachasma_alloeum                   -------------------
Telenomus_podisi                    -------------------
Nasonia_giraulti                    -------------------
Nasonia_vitripenis                  -------------------
Copidosoma_floridanum               -------------------
Trichogramma_pretiosum              -------------------
Brachycistis_timberlakei            -------------------
Crioscolia_alcione                  -------------------
Chrysis_viridula                    -------------------
Trachymyrmex_septentrionalis        -------------------
Trachymyrmex_cornetzi               -------------------
Atta_cephalotes                     -------------------
Trachymyrmex_zeteki                 -------------------
Acromyrmex_echinatior               -------------------
```

```
Vollenhovia_emeryi                 -------------------
Solenopsis_invicta                 -------------------
Monomorium_sp                      -------------------
Wasmannia_auropunctata             -------------------
Harpegnathos_saltator              -------------------
Dinoponera_quadriceps              -------------------
Cerapachys_biroi                   -------------------
Linepithema_humile                 -------------------
Pogonomyrmex_barbatus              -------------------
Camponotus_floridanus              -------------------
Pseudomasaris_vespoides            -------------------
Chyphotes_mellipes                 -------------------
Athalia_rosae                      -------------------
Neodiprion_lecontei                -------------------
Cephus_cinctus                     -------------------
Polistes-c_canadensis              -------------------
Polistes-m_metricus                -------------------
Polistes_dominula                  -------------------
Mischocyttarus_flavitarsis         -------------------
Corydalus_cornutus                 -------------------
Onthophagus_taurus                 -------------------
Aleochara_curtula                  -------------------
Nicrophorus_vespilloides           -------------------
Oryctes_borbonicus                 -------------------
Anoplophora_glabripennis           -------------------
Leptinotarsa_decemlineata1         -------------------
Leptinotarsa_decemlineata2         -------------------
Priacma_serrata                    -------------------
Gyrinus_marinus                    -------------------
Dendroctonus_ponderosae            -------------------
Meloe_violaceus                    -------------------
Tribolium_castaneum                -------------------
Hypothenemus_hampei                -------------------
Agrilus_planipennis                -------------------
Arachnocampa_luminosa              -------------------
Tetranychus_urticae                -------------------
Scolopendra_subspinipes1           -------------------
Scolopendra_subspinipes2           -------------------
Strigamia_maritima                 -------------------
Limulus_polyphemus                 -------------------
Litopenaeus_vannamei               -------------------
Homarus_americanus                 -------------------
Occasjapyx_japonicus               -------------------
Catajapyx_aquilonaris              -------------------
Lithobius_forficatus               -------------------
Meinertellus_cundinamarcensis      -------------------
Machilis_hrabei                    -------------------
Tricholepidion_gertschi            -------------------
Atelura_formicaria1                -------------------
Atelura_formicaria2                -------------------
Speleonectes_cf_tumulensis         -------------------
Anurida_maritima                   RPQMDFQPPISSTESLYP-
Pogonognathellus_sp                -------------------
Folsomia_candida                   FPDTSGRISSDEY------
Sminthurus_viridis                 -------------------
Ephemera_danica                    -------------------
Triops_cancriformis                -------------------
Eurylophella_sp                    -------------------
Ixodes_scapularis                  -------------------
Ixodes_ricinus                     -------------------
Ladona_fulva                       -------------------
Menopon_gallinae                   -------------------
Pseudomallada_prasinus             -------------------
Liposcelis_bostrychophila          -------------------
Diaphorina_citri                   -------------------
Apachyus_sp                        -------------------
Pachypsylla_venusta                -------------------
Cryptocercus_wrighti               -------------------
Orussus_abietinus                  -------------------
```

(B)

```
                                  1                                                50
Tenthredo_koehleri                ------------------------CPACGPD-RQGQCFGPKICCGPS---
Tetramorium_bicarinatum           ------------GDIAPLGTIVRECPSCGPN-HLGQCFGPHICCGPS---
Ceratosolen_solmsi_marchali       MTNYPRGGKR----------AMFANPTFLLD-KIAREFGPHICCGPT---
Lepicerus_sp                      -VNCPRGGKRGELPFLSIQGLVKECQSCGPG-HQGQCFGPNICCGTN---
Inocellia_crassicornis            --------------------------------------------------
Osmylus_fulvicephalus             -----------SVYFFGKSMYTLQCISCGPQ-RSGQCFGPAICCGP----
Aethina_tumida                    ------------------------CVSCGPG-HTGQCFGPSICCGP----
Pogonus_chalceus                  ------------------------CIACGPG-HSGQCFGPNICCGP----
Dastarcus_helophoroides           -----------------------QCIPCGPA-RTGQCFGPNICCGP----
Diabrotica_virgifera_virgifera    ----------------------------------GQCFGPNICCGP----
Prorhinotermes_simplex            -----------------------QCARCGPA-KLEHCYGPAICCGPQ---
Periplaneta_americana             --------------------------------------------------
Ceuthophilus_sp                   ------------------------CARCGPA-KLGHCYGPAICCAPQ---
Blaberus_atropos                  ---IFFLCNVDIKIMHLKYLDTKQCARCGPA-KLGHCYGPAVCCGPQ---
Medauroidea_extradentata          ----------VTHFNSSPGFKHKKCPRCGPA-KLGHCYGDNICCGVE---
Oncopeltus_fasciatus              ---------FLLLFKCKIYFFQQAFPRCEPD-REGRCLGRAICCGPK---
Scylla_paramamosain               ----------------------------------GRCVGPDICCGAR---
Campodea_augens                   ----------------------------------GRCFSPDLCCGPE---
Baetis_sp                         --------------------------------------------------
Isonychia_bicolor                 --------------------ACLQCARCGFG-LAGRCFGPRICCDTR---
Tetrodontophora_bielanensis       ------------IPEQHNTNKVRECASCGPVGQGGRCYGPETCCSPN---
Frankliniella_occidentalis        -----------------------QCTPCGPG-GEGLCVGPGICCSPV---
Calanus_finmarchicus2             ------PVSGKKRSLHVGEEQVRDGELTCPSNPAGLCYSPGLCCVQGGCY

                                  51                                              100
Tenthredo_koehleri                --IGCFID-----------------TAETHKCRKESLYS--RPCTAGFA-
Tetramorium_bicarinatum           --IGCFIG-----------------TSETYRCRKESLYT--RPCVAGYA-
Ceratosolen_solmsi_marchali       --MGCLLG-----------------TPEAHHCQKESLYS--QPYVAGFA-
Lepicerus_sp                      --IGCFIG-----------------TPETYKCKMESLYS--RPCIAGFS-
Inocellia_crassicornis            --FGCLVG-----------------TPETIRCERDGAFQEREPCIAGRS-
Osmylus_fulvicephalus             --FGCLIA-----------------TAETTRCERDGAFHEREPCIAGKS-
Aethina_tumida                    --FGCLMG-----------------TSETIRCQREGFFQEREPCIAGSS-
Pogonus_chalceus                  --FGCLIG-----------------TSETLKCQREGFFQEPEPCIAGNG-
Dastarcus_helophoroides           --FGCLVG-----------------TPDTIKCQREGFFHERDPCIAGNS-
Diabrotica_virgifera_virgifera    --FGCLLG-----------------SPETVRCQREGSFHEREPCIAGSG-
Prorhinotermes_simplex            --IGCLVA-----------------TPETARCLTEAASP--VPCIAPTGV
Periplaneta_americana             ---GCLIA-----------------TPDTARCLSEAASP--VPCIAPTGA
Ceuthophilus_sp                   --FGCLIG-----------------TPDTDPCQREDQSP--IPCIGTG--
Blaberus_atropos                  --IGCLIA-----------------TPDTARCLNEAASP--IPCTA----
Medauroidea_extradentata          --IGCLLA-----------------TPDTQVCQRESMTP--DPCVGPIG-
Oncopeltus_fasciatus              --IGCLVG-----------------TPAILSQCSEQPPE--LPYSKPYS-
Scylla_paramamosain               --IGCFLG-----------------SRETRMCRTENMVPITCYNSDLKPC
Campodea_augens                   --IGCYFR-----------------TKESSVCKSENLFPVPCEN-PAKSC
Baetis_sp                         ------VAPAATA-----------SNLALLPCVAESLFPEPCSNGPTVRC
Isonychia_bicolor                 --LGCMVGSAMEP-----------LQAELLPCLAESIFPAACHN-HAATC
Tetrodontophora_bielanensis       --FGCMVG-----------------TPETHTCLIEDRIN-----------
Frankliniella_occidentalis        --FGCVLTSRGGCGRGAVFAPRCSPSSAALAAASAAAVPLDAPCGADLTP
Calanus_finmarchicus2             ADGGCLPG-----------------LTERGEEQIKTRESVSKRGLRFLYG

                                  101                                             150
Tenthredo_koehleri                --MCRGNTGRCAADGICCSQESCQVDPNCKITDDSITS------------
Tetramorium_bicarinatum           --MCRGNTARCASNGICCSQDSCYVDASCKNSDGVRNDQKFDANLNEVLP
Ceratosolen_solmsi_marchali       --MCRGNSGRCAANGIMNNIPKNHVSLIHHAN------------------
Lepicerus_sp                      --MCRDNNGRC---------------------------------------
Inocellia_crassicornis            --NCRGDTGRCAADGICCTQGNYE--------------------------
Osmylus_fulvicephalus             --SCRGDTGRCAFDGICCTQGMCTVQLKLGLKSLPNAIRLI---------
Aethina_tumida                    --FCRKNTGRCAIDGICCNQESCHVDKHCSLDEKKSSENLMGMEL-----
Pogonus_chalceus                  --YCRKNTGRCAADGVCCTQ------------------------------
Dastarcus_helophoroides           --SCRKNTGRCAADGICCS-------------------------------
Diabrotica_virgifera_virgifera    --FCRKNTGRCATDNICCDQDSCYTDRQCSSDEKLKAEALSGLDIYNFLN
Prorhinotermes_simplex            QCGEGKLAGRCTANGVCCTHGKQSSTMYCLYVFNQAQVQL----------
Periplaneta_americana             QCGEGKSAGRCTANGVCCTHGEASQNIYNLQYSFITSTTFLLPYLLEILK
Ceuthophilus_sp                   SGPRCGEQGHCTANEVCCTQESCHIDPTCRLTLPAEGGLPDGPDATTDLS
Blaberus_atropos                  --------------------------------------------------
Medauroidea_extradentata          -GAPCGNSGKCAAPGVCCTPDSCTIDPTCHQTLSQEMASLYLYNAGNARG
Oncopeltus_fasciatus              -------IGVCLSDGGLLFIG-----------------------------
Scylla_paramamosain               G---RMQEGRCGAPGICCTENKCEMNDDCVAEDTQGEE------------
Campodea_augens                   G---TDRNGKCAGQGLCCTEDSCTVDSSCHVILKDLPPPPPAAVAPPSPW
Baetis_sp                         G---RGAKGVCAAPGVCCFHNSCETDASCAKMRR----------------
Isonychia_bicolor                 G---RMATGQCAATGLCCYQDSCHPDMTCRGRSE----------------
Tetrodontophora_bielanensis       --------SVCHNPGIPCTVNVDNGNINGFCATG----------------
Frankliniella_occidentalis        D---GSAPGRCAAQGVCCTH------------------------------
Calanus_finmarchicus2             MAPSQMNLFGQEGMAIPCIGPYCNGKGARSHGKQKQRKRKQSQEHVNRDQ
```

```
                                    151
Tenthredo_koehleri                  ---------------------------------
Tetramorium_bicarinatum             GNEFSREIM------------------------
Ceratosolen_solmsi_marchali         ---------------------------------
Lepicerus_sp                        ---------------------------------
Inocellia_crassicornis              ---------------------------------
Osmylus_fulvicephalus               ---------------------------------
Aethina_tumida                      ---------------------------------
Pogonus_chalceus                    ---------------------------------
Dastarcus_helophoroides             ---------------------------------
Diabrotica_virgifera_virgifera      TY-------------------------------
Prorhinotermes_simplex              ---------------------------------
Periplaneta_americana               YISQ-----------------------------
Ceuthophilus_sp                     DRNLVYSLFNAISTYQ-----------------
Blaberus_atropos                    ---------------------------------
Medauroidea_extradentata            VSQISDVDDK-----------------------
Oncopeltus_fasciatus                ---------------------------------
Scylla_paramamosain                 ------VEESQRGGRPRLDLLAAARDRWEEQ--
Campodea_augens                     LKYPSVFRVLQQQQQPNTDLSSATGALWDDMLE
Baetis_sp                           ------VNAIARESNAFA---------------
Isonychia_bicolor                   ---------------------------------
Tetrodontophora_bielanensis         ---------------------------------
Frankliniella_occidentalis          ---------------------------------
Calanus_finmarchicus2               ALLGSSNYDSAVSDDQFLKHNCDWMDLYLAR--
```

Supplementary Figure S2. Alignment of multiple copies of inotocin precursors and receptors. Relevant parts of the sequences (inotocin peptides and transmembrane domain; TM) are highlighted in yellow. Transmembrane domains were assigned according to the human neuropeptide S receptor (GeneBank: NP_997055)[1] after alignment with the receptors. (A) Inotocin precursors and (B) inotocin receptors. Multiple copies of precursors were only found in 4 species; multiple copies of receptors were only found in eight species.

(A)

Alignment of 2 inotocin precursor sequences of *Scolopendra subspinipes*:

```
                                                        mature peptide
Scolopendra_subspinipes1      ------HWLLFFYVIGMTSSCFITNCPP--GGKRSLGEHKRTLRQCTACGPGSQGRCFGP
Scolopendra_subspinipes2      MRSSTCHWILYLSLVGLVSACYIINCIDNDGRKRSIDHQKKAIRQCMSCGPGMKGRCFGP
                                    **:*:: ::*:.*:*:* **    * ***: .:*:::*** :**** :******

Scolopendra_subspinipes1      DLCCGEFFGCYLKTRESAICRFENLSPIPCDNDVLSCGPDGAKCAADGLCCSSDQCKMEE
Scolopendra_subspinipes2      DLCCIESFGCFTQTPESAVCHLENLSPVPCDNDTPSCGL-GANCAVDGLCCNTSQCKRDE
                              **** * ***: :* ***:*::*****:*****. ***  **:**.*****.:.*** :*

Scolopendra_subspinipes1      RCR
Scolopendra_subspinipes2      RC-
                              **
```

Alignment of 3 inotocin precursor sequences of *Calanus finmarchicus*:

```
                                                                mature peptide
Calanus_finmarchicus2      --------------------------------------PVSGKKRSLHVG---EEQVRDG
Calanus_finmarchicus1      MITSWKILDPRIRRSWSFLIILLAQTMSASHGCFISNCPVSGKKRSVGEEQMVSFQAREE
Calanus_finmarchicus3      MITSLKILDPSIRRSWRFLMILLAHTIPDSHGCFISNCPVSGKKRSVGEEQLVSFQAREE
                                                                 ********:      . *.*:

Calanus_finmarchicus2      ELTCPSNPAGLCYSPGLCCVQGGCYADGGCLPGLTERGEEQIKTRESVSKRGLRFLYGMA
Calanus_finmarchicus1      VLACPSNPAGLCYSPGLCCVQGGCYADKGCLPQMEESRENRVEP--RLNKRDLRFLYGWA
Calanus_finmarchicus3      VLACPSNPAGLCYSPGLCCIQGACYADM--------------------------------
                            *:****************:**.****

Calanus_finmarchicus2      PSQMNLFGQEGMAIPCIGPYCNGKGARSHGKQKQRKRKQSQEHVNRDQALLGSSNYDSAV
Calanus_finmarchicus1      PAHEKMFGQTGFSIPCIGPYCNGNEKMI-----MRKEKQSQEKKSDDKVLDGSSNYDSLV
Calanus_finmarchicus3      ------------------------------------------------------------

Calanus_finmarchicus2      SDDQFLKHNCDWMDLYLAR
Calanus_finmarchicus1      YNENLSNQRCNWMDLYLAR
Calanus_finmarchicus3      -------------------
```

Alignment of 2 inotocin precursor sequences of *Atelura formicaria*:

```
                                                  mature peptide
Atelura_formicaria1      ---------------------ACFITNCPPGGKRSMGQMGLHVNKQCTSCGPARTGRCYG
Atelura_formicaria2      SLSKGILSVTIFFLAVISVTTACFITNCPPGGKRSMGQMGLHANKQCTSCGPARMGRCYG
                                              *********************.*********** *****

Atelura_formicaria1      PAICCGPRFGCLVGTRDTAVCQTENFTPVPCGNRGPSCGESGRCAANGVCCTEETCTIDP
Atelura_formicaria2      PAICCGPRFGCLVGTRDTAVCQ--------------------------------------
                         **********************

Atelura_formicaria1      SCRISNEDISSSRQMFPITNSVSMYLEELNDDLPARVGSDRR
Atelura_formicaria2      ------------------------------------------
```

Alignment of 2 inotocin precursor sequences of *Leptinotarsa decemlineata*:

```
                                                          mature peptide
Leptinotarsa_decemlineata1      MVRSVPLTIIYLFIFVMYVESCLITNCPKGGKRGGEFSTSESNIKTVSINLNNYKHILQC
Leptinotarsa_decemlineata2      MSKTVSLFTLFLSVFVIFVESCLITNCPIGGKRSGKFSLTESNI-------------KQC
                                * ::* *  ::* :**::********** ****.*:** :****              **

Leptinotarsa_decemlineata1      ISCGPGHTGKCFGPNICCGPFGCLLGSPETERCHKEGLFQETEPCIAGFSTCRKNTGRCA
Leptinotarsa_decemlineata2      ISCGPGHTGQCFGPNICCGPFGCLMGSHETFGCQRGG-FHESEPCIAGFSSCRKNTGRCA
                                *********:**************:** **  *:: * *:*:********:*********

Leptinotarsa_decemlineata1      AEKICCTQGIWTFFLVSN
Leptinotarsa_decemlineata2      TENICCTQGIFKIRSYT-
                                :*:*******:.:   :
```

(B)

Alignment of 3 inotocin receptor sequences of *Limulus polyphemus*:

```
                                                                          TM1
Limulus_polyphemus3        MSSLKEEQTTELTNASFVNFSPIENITV-GDTQRDETIARVEVGVLAIIFILTIIGNTCV
Limulus_polyphemus1        MSTLDFGETPEILNVSFMNSSTIINNTKDKGIQRDETIARIEVGVLASIFTLTILGNMCV
Limulus_polyphemus2        MSTLAKGETTGFLNLSFVNLSTITNNTKADVVQRDETVARIEVAVLATIFTLTVIGNTIV
                           **:*   :*  : * **:* * * * *     *****:**:**.*** ** **::**  *

                                                  TM2                            TM3
Limulus_polyphemus3        LVALAVRRTNMTRMYYFLLHLCISDLITAFFHVLPQLAWDAAHRFYGGNVLCKVVKYLQI
Limulus_polyphemus1        LVALAVRRIKMARMYYFLLHLCISDLITAFFNILPQLAWDVTYRFYGGNILCKVIKYLQI
Limulus_polyphemus2        LVALVVRRIKMTRMYYFLQHLCISDLITAFFHVLPQLAWDVTYRFYGGNVLCKIVKYMQI
                           ****.*** :*:****** ************.:*******.::******:***::**:**

                                    TM3                                TM4
Limulus_polyphemus3        LGPYLSSYVLMVTAIDRYQAICFPLTRCTWTPRRSKLMIVGAWIISILCCSPQAFIFSYQ
Limulus_polyphemus1        LGPYLSSYVLMVTAIDRYHAICFPLRNCVWTPRKSKLMIIAAWTISMLCCVPQVFIFSYQ
Limulus_polyphemus2        LGPYLSSYVLLVTAIDRYQAICFPLTSCVWTPRKSKIMIANAWIISILCCFPQIFIFSYQ
                           **********:*******:******  *.****:**:**  ** **:*** ** ******

                                                         TM5
Limulus_polyphemus3        QVSSDPLVFDCWGTFIQPWGEKVYVLWYTISQFFIPLLVITFTYVNVTKTVWNNYYLRKK
Limulus_polyphemus1        KVLIEPITFDCWGVFIQPWGEKVYVLWYALSQFFIPLIVITFTCVRICKNVWYNFHLRGK
Limulus_polyphemus2        KVSEDPVTFDCWGIFIQPWGEKVYVLWYAVSQFFIPLVVIAFTYIRICKSVWYNLQMRRQ
                           :*  :*:.***** **************::*******:**:** :.: *.** *  :* :

                               ICL3                               TM6
Limulus_polyphemus3        NF-------IHCRSVPDLSRAKVKTIRITIVVIACYIICSTPFLAVQLWAYWSPYAQNSP
Limulus_polyphemus1        NS-------SRSHSMRGLSRAKVKTVRITVVVIACYIICSTPFIVVQLWAYWSPYAQTSP
Limulus_polyphemus2        ASNCETFTVPRSHCMRGLSRSKVKTFKITVVVITCYIICSTPFIIVQLWAYWSPNAKNSS
                                     :.:.:  ***:****.:**:***:*********: ********* *:.*

                                     TM7
Limulus_polyphemus3        IWKGPTVAILMLLASLNSCVNPWIYLAFNHNLITALKQLCCRSVLQDYMAQSTEATSNNN
Limulus_polyphemus1        IWKGPTVAILMLLASLNSCVNPWIYLAFNHNLVTALKHICCHERSQGYSPQVPVENIN-N
Limulus_polyphemus2        IWKGPTVAILMLLASLNSCVNPWIYLAFNYNLVTALREVCCQTSSKSFSSQVPENTDN-N
                           *****************************:**:***:.:**:   : :  *    . * *

Limulus_polyphemus3        NANRSYPTENEVNSSYPLSFFIAQEEPVMLRNRLKKKQ----------------------
Limulus_polyphemus1        TTSRSSQTDNEICTSYPLSLRCGVHYTSSFRLKKTKITGCRPCPNIQDLH---DEAETFS
Limulus_polyphemus2        TFSR-----SERCSSYPLSVRTGSRHKFP--DGSTTRTRKRLCRELGSKNESTDVGENRG
                           . .*     .*  :*****.  . .         ..

Limulus_polyphemus3        ------------------------
Limulus_polyphemus1        A-----------------------
Limulus_polyphemus2        SVTQTETTSEPGKFVTRDKDKTLC
```

Alignment of 3 inotocin receptor sequences of *Ixodes scapularis*:

```
                                                                          TM1
Ixodes_scapularis2         ---------------------------------------QVEVAVLTFIFGFTVLGNTCV
Ixodes_scapularis1         MDV-PSNQKPEFAASVPTVLNFASTNLTASTETRDESLALVELAVLTLIFAFTVMGNSTV
Ixodes_scapularis3         MDLVANQDQPAFSTSLPDSLDDVPVNETFWTGTRDESLAQIELGVLTAIFTFTVIGNTTV
                                                                   :*:.*** ** ***:**: *

                                                    TM2                         TM3
Ixodes_scapularis2         LVALAARRSKMTRMYYFLIHLCVSDLTTAFLSVLPQLGWDATHRFRGGNLVCKAVKFGQL
Ixodes_scapularis1         LLALATRRSKMTRMYYFLLHLCVSDLITAFLTVLPQLGWDATYRFQGGNLACKAVKFGQL
Ixodes_scapularis3         LAALAARRSKMSRMYYFLLHLCVSDLITAFFTVLPQLGWDATYRFRGGNLACKTVKFGQL
                           * ***:*****:******:******* ***::**********:**:****.**:******

                                   TM3                              TM4
Ixodes_scapularis2         LGPYLSSYVLVVTAVDRYQAICFPLSNCSWSPTKCKLLIAGAWVAALVCCVPQVFIFSYQ
Ixodes_scapularis1         LGPYLSSYVLVVTAVDRYQAICFPLSNCSWTPTKSKLLICAAWVAALLCCVPQVFIFSYQ
Ixodes_scapularis3         LGPYLSSYVLVVTAADRYQAICFPLSNCSWTPTKSKLLISAAWVASLLCCVPQVFIFSYQ
                           **************.***************:***.****..****:*:************

                                                           TM5
Ixodes_scapularis2         EVAPGVFDCWGTFAQPWGLRAYVTWYSVSVFFVPLAVLSFTYVCICRSIWRNLYLKRKSS
Ixodes_scapularis1         EVSPEVFDCWGTYVESWGLRAYVTWYGMSVFFVPLMVLTFTYVCICRSIWRNLYLKRKSS
Ixodes_scapularis3         EISPKVFDCWGTYVEPWGLRAYVTWYGVSVFFVPLVVLSFTYVCICRSIWRNLYLKRKSS
                           *::* *******:.: **********.:******* **:*********************

                                      ICL3                                TM6
Ixodes_scapularis2         DA-----------------------------DHSVRGMSRAKVKTVKITVVVIALYIVCS
Ixodes_scapularis1         DAESWKGERAYRFRGLAHNGTKQDSSFVGPRSHSVRGLSRAKIKTVKITVVVIALYVVCS
Ixodes_scapularis3         DAESWKGSRAYRFKGTAQNGTKQDSSF-----------------TVKITVVVIALYVVCS
                           **                                          ************:***

                              TM6                             TM7
Ixodes_scapularis2         SPFICVQMWMYWSPDVDLADVWINATVTILMLLNS-L-----NSCVNPWVYLFFNRSLVH
Ixodes_scapularis1         SPFICVQMWMYWSPHVDMANPWISE-----------------------------------
Ixodes_scapularis3         SPFICVQMWMYWSPDAD-------------------------------------------
                           **************..*

Ixodes_scapularis2         TLRHQICRCPDAKNVSSGIAGTGSCPSAVILADLTTQGTEAQSVLSYKSSPTDTSARLAT
Ixodes_scapularis1         ------------------------------------------------------------
Ixodes_scapularis3         ------------------------------------------------------------
```

```
Ixodes_scapularis2      AVVGKRGATDVEEDLQCSLEKTS
Ixodes_scapularis1      -----------------------
Ixodes_scapularis3      -----------------------
```

Alignment of 2 inotocin receptor sequences of *Metaseiulus occidentalis*:

```
                                                       TM1                        TM2
Metaseiulus_occidentalis1     --MNSSAGNFGDARDETIAHVRITVLCFCLLFTLGGNLFVFLLVTRNRPKSVHQLHIYHF
Metaseiulus_occidentalis2     MEYNITDTDFVPQPENWVLSVKCATLVIIFFLTLSSNLFVLHAVLLRNRQNRPLSRVQLF
                                 *  :   :*     :: :   *: :.* : :::**..****:  *   .. :.    ::  *
                                           TM2                                  TM3
Metaseiulus_occidentalis1     MVHLSLADMLVGVFNILPQIVWEIYFRFRWGNIACKFVKFMQIFVLYLSTYTLVGLSLNS
Metaseiulus_occidentalis2     MLHLTVADILVALLNILPQLAWDLTIQFRGGAVLCKLVKFAQVYVLYLSTYILTGMSLDR
                              *:**::**:**.::*****:.*::  ::** *  :  **:***  *::******* *.*:**:
                                                    ICL2                          TM4
Metaseiulus_occidentalis1     SLVVRSGSSST-------------------S---------------SRLWVILGAGWFLS
Metaseiulus_occidentalis2     LITMRAIESQWQTASLRRSDHDQSSDKSPQSHKIKFNNHRCRVGYRKFAKKLIVFAWFLA
                               :.:*:  .*.                  *                .     ::   .***:
                                                 ECL2                           TM5
Metaseiulus_occidentalis1     ALLASPQVYIFSFKQLSN--GVYDCWGTFDPP-LTSVRYVVYFIAMALIVPALIMGACYS
Metaseiulus_occidentalis2     AALSIPQLFLFSYTRIHDHGSWYDCKANFSWHRFGQRIYTTYFVVLILGVPVVMMLFCYI
                              * *: **:::**:.:: :   . ***  ..*.     : .    *..**:.: * **.::*   **
                                                  ICL3                           TM6
Metaseiulus_occidentalis1     YLCRAS---------------------------SKRLMSDAKLKTIRMTMVMVLVFVLCW
Metaseiulus_occidentalis2     QICLIIVRIQRHHMTKTTSAPANFGVNTAGFFESNRRITKAKVRMVKMTFTVVLCFIVCW
                               :*                               *:*  ::.**::  ::**:.:** *::**
                                                                    TM7
Metaseiulus_occidentalis1     TPFCCAQLYL---VFGGEEASTFVTMCLMVPNLNSCANPWVYLSFSTDLRRRLVNF-CSL
Metaseiulus_occidentalis2     SPYSIAELLLAYKIAGGEHVSPPFMVFLLLASLNSAVNPWIYTAFTNSFACKSLRTLKPE
                              :*:.  *:* *    : ***..*    .  :  *::  .***..***:*  :*:..:    :  :.

Metaseiulus_occidentalis1     IHLRGNRYGEESRKPCTRANAIVCEKPARQLRGYRIS
Metaseiulus_occidentalis2     VHLITV-------------------------------
                              :**
```

Alignment of 2 inotocin receptor sequences of *Varroa destructor*:

```
                            TM1                            TM2
Varroa_destructor1      MAGNIVVLVLVLQSKPKSAHLSRIYYFLLHLSIADILVGIFNISPQLVWDIYFRFPLGNF
Varroa_destructor2      -------------EARRSSSLSRVQLFMMHLSIADILVALLNILPQLAWDITARFYGGAL
                                     .  :*: ***:  *::*********.::** ***.***   **   * :
                                     TM3                               TM4
Varroa_destructor1      ACKIVKFLQVFVLYLSTYVLAGMAVDRYLAIRSGINRPIVVVRTILSVSWLVAAVLASPQ
Varroa_destructor2      LCKFVKYAQVLVLYLSTYILTGMSLDRLVSM-----------------------------
                         **:**: **:*******:*:**::** :::
                                                            TM5
Varroa_destructor1      LYIFSFQKLPNGAHDCWATFEPPITSFRYVLFFITAVLFIPVTLMALCYTYLSWIISKRS
Varroa_destructor2      ------------------------------------------------------------

                                              TM6                         TM7
Varroa_destructor1      LSHTKLNPIRMTMVMVIVFVLCWTPFCCAQLYLESTGQVPSIFITLFLLVPNLNSCANPW
Varroa_destructor2      ------------------------------------------------------------

Varroa_destructor1      VCLTFSTTLRRKLTDALSFFGFCEHLSVYRSKNAITISILQYRPPNRSKRQPRAYRGA
Varroa_destructor2      ----------------------------------------------------------
```

Alignment of 2 inotocin receptor sequences of *Achipteria coleoptrata*:

```
                                                                           TM1
Achipteria_coleoptrata2     MSSDSSDMSVENISQTIIFQIINDDNMTANTSLAVKRNEKLVLLEIGILLLIFLLIVFGN
Achipteria_coleoptrata1     ----------EN----------ITEELKNNTTVSDTRDEHLAIFEIATLSLLFVLILFGN
                                      **              :::. **::: .*:*:*.::**. * *:*:**:***
                                TM1                    TM2                      TM3
Achipteria_coleoptrata2     SCVLIALQINKLKMNRMYYFLQHLSISDMITAFFNVLPQLAWEITHRFYGGNILCKTIKY
Achipteria_coleoptrata1     SCVLLALLMRKIKMNRMYYFLLHLIIADYLVAFFNVLPQLIWDITYRFYGGNLLCKVIKY
                            ****:** :.*:********* ** *:* :.********* *:**:******:***.***
                                      TM3                             TM4
Achipteria_coleoptrata2     LQIFGPYLSSYVLVMTAIDRYQAICNPLSNCKWTPGRSKLMITIAWIVTSFCCSPQIFIF
Achipteria_coleoptrata1     LQILGPYLSSYVLVMTSIDRYQAICYPLTNCQWTPTRSQLMITLAWIIALLCCIPQVLIF
                            ***:***********:******** **:**:*** **:****:***::  :** **::**
                                                       TM5
Achipteria_coleoptrata2     SYQQIPGTDAIYDCWGTFQ-KPWGERIYVTWYAVSVFIIPFIIIVVTHYHICREIWLNLH
Achipteria_coleoptrata1     SYQQIPGTD-IYDCWGTFPLQPYGERIYVTWYAISFFFIPFILLTATHVCICREIW----
                            ********* ********   :*:**********:*.*:****::..**  ******
                                                                                     TM6
Achipteria_coleoptrata2     KKRKSFKLNKNRVGKSYRFKSRGKLEVSVDKDFKEDFSPRTHSIHGLSRAKIKTVKITVV
Achipteria_coleoptrata1     ------------------------------------------------------------

                                  TM6                        TM7
Achipteria_coleoptrata2     IILCYIMCSTPFICVQLWAYWVPTAQKSHIWNVKYTYNIYLMFFSIIDVTIAFKILYSE
Achipteria_coleoptrata1     -----------------------------------------------------------
```

Alignment of 5 inotocin receptor sequences of *Platynothrus peltifer*:

```
                                                                            TM1
Platynothrus_peltifer5      METVSDLITFSREQEFDFELLNDSNNVNKSSFEIRDSRNESLAIIEISLLTFMFISILIG
Platynothrus_peltifer3      -------------------M--------TGNGSLTVKRDEKWVLLEIGILSLIFILIVFG
Platynothrus_peltifer1      MEEIGTTVTLEELEDKLPET--------IIANVTKDSRDEHLAVFEIATLGLIFLLIVFG
Platynothrus_peltifer2      ME-VSTAIS-EETDGIPFET--------SSIKPNITGRNENLALIEVGTLGTIFVLIVFG
Platynothrus_peltifer4      MSNVIMSI--NNTTSVLYN------------ISGSDKRDENLAIMEVSILALIFILIIIG
                                                                 *:*  .::*:. *  :*: *::*
                               TM1                        TM2                      TM3
Platynothrus_peltifer5      NIFVLTALIVRDSKMNRMYYFLFHLCIADLITAFFNVLPQLIWDITYRFYGGNVLCKLIK
Platynothrus_peltifer3      NSCVLIALQVRKLKMNRMYYFLLHLCIADLITAFFNVIPQLAWEITHRFYGGNFLCKTIK
Platynothrus_peltifer1      NSCVLIALIMRKLKMNRMYYFLLHLCIADLITAFFNVLPQLAWDITYRFYGGNILCKTIK
Platynothrus_peltifer2      NCCVLLALALRRLKMTRMYYYLLHLCISDLMTGFFTVLPQLAWDITYRFNGGNILCKAVK
Platynothrus_peltifer4      NLCVLIALAVKRFKMTRMYYFLLHLCISDIITGCFTVMPQLAWDITHRFNGGNILCKLVK
                            *  ** ** ::  **.****:*:****:*::*. *.*:*** *:**:** ***.*** :*
                                      TM3                                 TM4
Platynothrus_peltifer5      YLQILGPYLSSYILVITAIDRYKAICFPLSNCSWTPKRSQLMISIAWVLSLIFCAPQLFI
Platynothrus_peltifer3      YLQILGPYLSSYVLVMTAIDRYQAICYPLSNCAWTPSRSKLMILMAWIVSLLCCAPQIII
Platynothrus_peltifer1      YLQILGPYLSSYVLVMTSIDRYQAICYPLSNCAWTPKRSQLMISFAWIIALLCCIPQVII
Platynothrus_peltifer2      SMQILGPYLSSYVLVVTAIDRYQAICFPLSNCSWTPRRAKLMISCAWIISIVCSLPQAFI
Platynothrus_peltifer4      CLQLFGPYLSSYVLVVTAIDRYQAICFPLTNCHWTSRKSKLMITIAWIISILCSAPQAFI
                             :*::*******:**:*:****:***:**:** **  :::***  **::::: . ** :*
                                                                TM5
Platynothrus_peltifer5      FSYQQIPGTLGNVYDCWGIFPQPYGERIYVTWYAITVFVIPCIIILFAHVCICREICNNL
Platynothrus_peltifer3      FSYQEI-GS--QIFDCWGTFSQPYGERIYVTWYAVSVFIIPFIILTFTHVHICREIWLNL
Platynothrus_peltifer1      FSYQQIPGY--DIIDCWGTFPQPFGERIYVSWYAISFFFVPFILLTFTHVCICREIWRNV
Platynothrus_peltifer2      FSYQEVPETN-HIYDCWGTFPQPWGERIYVTWYAISVFFIPLVILTFTHIYICREIWVNL
Platynothrus_peltifer4      FSYQEIPRTN-GVRDCWGTFPQPYGERIYVTWYAITVFFIPLIIITYTYVYICREVWANV
                            ****::      : **** * **:******:***::.*.:* ::: :::: ****:  *:
                                               ICL3
Platynothrus_peltifer5      HRKRESIEEIKNCIHYSFKRNKEFS-TSSLKSTSLTSFSGPIKSPI------TYSIRPSK
Platynothrus_peltifer3      HKKRKSFKINRNKSSSSCNNRNDKLDCDSVNSPETSE--HRSGTRLLVTVGKSYRFKGKG
Platynothrus_peltifer1      HQKRKSVKMEQNKIIQNDDS-DRVSSCSS----TNDS--IARSTNLLVTVGRSYRFKGRG
Platynothrus_peltifer2      NLKRQTGRRETQTINQNIDNSNSY---------------SRTRKCLLIALNRVYRLGRRQ
Platynothrus_peltifer4      RRKRQTLKPEISLIEVRKNW-------------------G--------------KLSKSS
                            . **:: .   .      .                                    :
                                        ICL3                                    TM6
Platynothrus_peltifer5      RLRRIHSQIDLKKDLRNSLTNLSVKVNDLNSTPRSHSLRFVSRAKIKTVKITIVIMLCYV
Platynothrus_peltifer3      QVEVSVEK-DC--------------GKEDYNSPRTHSIHGLTRAKIKTVKITIVIIMCYI
Platynothrus_peltifer1      RVEVSVDG-PK--------------SRGTY-NPRSHSLNGLSRAKIKTVKITVVVILCYI
Platynothrus_peltifer2      DTETAEIQINCDNQEVRPLHTVVTFQESVSSSPRVNTFNRLSRAKIKTVKITVVVILCYV
Platynothrus_peltifer4      DDKSRLQSDRC--------------LANNLLNPRSHSIYRISKAKIKTVKVTVVVVICYI
                     .                                     .** .::  :::*******:*:*:::**:
                                   TM6
Platynothrus_peltifer5      ICSMPFISVQLWVYWFPSAQSFFQTSE----LSNIY-------
Platynothrus_peltifer3      FCSTPFICVQLWAYWWPNAQQSPIWNVKYTYLSNNFA------
Platynothrus_peltifer1      ICSSPFICVQLWAYWWPNAQNSTFWSGKCSENSFIIQFRYSFQ
Platynothrus_peltifer2      ICSSPFICVQLWAYWWPNASTTTFWNGMFSLFII---------
Platynothrus_peltifer4      VCSSPFICVQVWAYWYPSAQETSFWTGKH--------------
                            .** ***.**:*.**:*.*.     .
```

Alignment of 2 inotocin receptor sequences of *Strigamia maritima*:

```
Strigamia_maritima1      MSNESHWEDDLTMDNATSNAPPPQS----------------S---AIADAKL-----TVL
Strigamia_maritima2      MA--THAE----GDSYTRTNPPPTKTIPMDDNNPNYNNKTTDGIMDIRDENLASIEIAVQ
                         *:  :* *     *. * . *** .                .    * * :*     :*
                                   TM1                                 TM2
Strigamia_maritima1      ALMFATIMLGNTIVITSLL--IRRKKLTRMYFFILHLCIADLIVAFLHVLPQLCWDVTHR
Strigamia_maritima2      SIILVVAIISNSFVFAALCCQRRFRPTSRMYLFMLHLSVADLLVAFLSLLPQLIWDVTFR
                         ::::.. ::.*::*:::*    *  :  :***:*:***.:***:**** :**** ****.*
                                            TM3                              TM4
Strigamia_maritima1      FQGNDFLCKVIKFGQLLGPYLSSYILVGMAIDRYLAICFPLN-SWAATRS-KGMVCVAWV
Strigamia_maritima2      FQGSDILCRVVKYLQVMVLYLSTYILVMMAVDRCRAVCWSITGHWNSLRAAKLMIVGAWV
                         ***.*:**:*:*: *::  ***:**** **:**  *:*: :.  * : *: * *:  ***
                             TM4                                           TM5
Strigamia_maritima1      VSVVFSSPQMFLFSYKYVSVLKYWECWVDFTLE-EERAYVTWYFVANTFVPLLVLVYTYS
Strigamia_maritima2      LAFLAAIPQAIIFGKMEI-RPGVHDCWAHFEQPWGEKAYVTWFVLSIFIIPLIVIAASYG
                         ::.: : ** ::*.   :      :**..*     *:*****:.::  ::**:*:. :*.
                                                         ICL3
Strigamia_maritima1      AICWAVWRNFRHKKNSPKGDFDSAASSSPTTATHLSVPQNVNHIQRNGSCRFKASESVNP
Strigamia_maritima2      FICYTLWIYDEEHGR--SGDVIALRAVGNGTG---------GGDTVTDSCRLYSRRVA--
                          **:::*   ..: .  .**. :  : .  *.              . ***: : . .
                                                  TM6                            TM7
Strigamia_maritima1      RSHGLQRISRAKIKTVKLTVVVITCYILCSMPYTCAMMWVAYDKRAQETAFYKGGLFPVL
Strigamia_maritima2      GVSNPNPISEAKVKTLKLTFLVVVCFFVCWSPFCITQLVLTFNPPSPDA--QIGSVEVIL
                              : **.**:**:***.:*:.*:::*  *:  : : ::::  : ::    *.:  :*
                              TM7
Strigamia_maritima1      VLVASLNSCVNPWVYLLFNKNLVHTLRHYLCCSKENEMKSHRTLATTYSRTSFSESVGPR
Strigamia_maritima2      LLLASLNSCTNPWIYVAFSGSLLNQLRMCLGLGLTRPK----DIASIGDEEPQPQQQQP-
```

```
                         :*:******.***:*: *. .*:. **  *  .  .       :*:  ..    :.  *
Strigamia_maritima1      VALQSLPPSSNNFLRVNDNKLNVRVAAVSDSALYSCEKRHSSPNLNVKSAASTLVLCSQQ
Strigamia_maritima2      --TNKLPSTKSEMV----------------------------------------------
                            :.** :..:::
Strigamia_maritima1      HHHDTQNHLKDGVTMETQCA
Strigamia_maritima2      --------------------
```

Alignment of 4 different splicing variants of inotocin receptor sequences of *Daphnia magna*:

```
                                                      TM1
Daphnia_magna4      MASSSLLSNVTNVTTELASSSRDEKLASIEIGTLSFILLLAVTSNLTMLIAIWRQRRNRP
Daphnia_magna3      MASSSLLSNVTNVTTELASSSRDEKLASIEIGTLSFILLLAVTSNLTMLIAIWRQRRNRP
Daphnia_magna1      MASSSLLSNVTNVTTELASSSRDEKLASIEIGTLSFILLLAVTSNLTMLIAIWRQRRNRP
Daphnia_magna2      MASSSLLSNVTNVTTELASSSRDEKLASIEIGTLSFILLLAVTSNLTMLIAIWRQRRNRP
                    ************************************************************

                                   TM2                              TM3
Daphnia_magna4      LSRMYFFMMHLSLADLLVALFNILPQLAWDITYRFHGGDVLCRFVKYTQIMTLYLSTYIL
Daphnia_magna3      LSRMYFFMMHLSLADLLVALFNILPQLAWDITYRFHGGDVLCRFVKY-------------
Daphnia_magna1      LSRMYFFMMHLSLADLLVALFNILPQLAWDITYRFHGGDVLCRFVKYTQIMTLYLSTYIL
Daphnia_magna2      LSRMYFFMMHLSLADLLVALFNILPQLAWDITYRFHGGDVLCRFVKYTQIMTLYLSTYIL
                    ***********************************************

                                               TM4
Daphnia_magna4      MFMAVDRYRAVCCRNLHWNSLKVAKCFVAASWVMSMLFAIPQAVIFHEEEISVGVTDCWV
Daphnia_magna3      ----------------TWNSRKVAKCFVAASWVMSMLFAIPQAVIFHEEEISVGVTDCWV
Daphnia_magna1      MFMAVDRYRAVCCRNLHWNSLKVAKCFVAASWVMSMLFAIPQAVIFHEEEISVGVTDCWV
Daphnia_magna2      MFMAVDRYRAVCCRNLHWNSLKVAKCFVAASWVMSMLFAIPQAVIFHEEEISVGVTDCWV
                                     *** ***************************************

Daphnia_magna4      QFAEPWGAKAYVTWFV------------------------------------------VS
Daphnia_magna3      QFAEPWGAKAYVTWFV------------------------------------------VS
Daphnia_magna1      QFAEPWGAKAYVTWFV------------------------------------------VS
Daphnia_magna2      QFAEPWGAKAYVTWFVVSIFGAPLLVVAVCYXXXXXXXXXWVQFAEPWGAKAYVTWFVVS
                    ****************                                          **

                         TM5                              ICL3
Daphnia_magna4      IFGAPLLVVAVCYGVICRQIWIYSQSAQPSLLPSDNSAIPSHSSITETGSTLSVMRRWFL
Daphnia_magna3      IFGAPLLVVAVCYGVICRQIWIYSQSAQPSLLPSDNSAIPSHSSITETGSTLSVMRRWFL
Daphnia_magna1      IFGAPLLVVAVCYGVICRQIWIYSQSAQPSLLPSDNSAIPSHSSITETGSTLSVMRRWFL
Daphnia_magna2      IFGAPLLVVAVCYGVICRQIWIYSQSAQPSLLPSDNSAIPSHSSITETGSTLSVMRRWFL
                    ************************************************************

                               ICL3
Daphnia_magna4      RAGMRWQKSRNSSNAIKNNSATASQLSDTIPMRSLATQQASNPLTTPDTIPMRSLATQQA
Daphnia_magna3      RAGMRWQKSRNSSNAIKNNSATASQLS-------------------ADPIPMRSLATQQA
Daphnia_magna1      RAGMRWQKSRNSSNAIKNNSATASQLS--------------------DTIP---------
Daphnia_magna2      RAGMRWQKSRNSSNAIKNNSATTSQLS--------------------DTIPMRSLATQQA
                    **********************:****                    * **

                               ICL3                                    TM6
Daphnia_magna4      SNPLTTENKISPMLPQPPPAAPQRCQQMSLRRSNSNQNRITKAKMKTIKLTLAVVLCFVA
Daphnia_magna3      SNPLTTENKISPMLPQPPPAAPQRCQQMSLRRSNSNQNRITKAKMKTIKLTLAVVLCFVA
Daphnia_magna1      -------NKISPMLPQPPPAAPQRCQQMSLRRSNSNQNRITKAKMKTIKLTLAVVLCFVA
Daphnia_magna2      SNPLSTENKISPMLPQPPPAAPQRCQQMSLRRSNSNQNRITKAKMKTIKLTLAVVLCFVA
                           *****************************************************

                          TM6                         TM7
Daphnia_magna4      CWAPFCITQLIMVYCPPTSQADVSPVAVIILLLASLNSCSNPWIYLAFSGSLLNQMRVCL
Daphnia_magna3      CWAPFCITQLIMVYCPPTSQADVSPVAVIILLLASLNSCSNPWIYLAFSGSLLNQMRVCL
Daphnia_magna1      CWAPFCITQLIMVYCPPTSQADVSPVAVIILLLASLNSCSNPWIYLAFSGSLLNQMRVCL
Daphnia_magna2      CWAPFCITQLIMVYCPPTSQADVSPVAVIILLLASLNSCSNPWIYLAFSGSLLNQMRVCL
                    ************************************************************


Daphnia_magna4      GLRWLRGQDKDSIGEDDRRGAAGPAGGQPADTNHRFGRGPRRAQTRDQHRQV--------
Daphnia_magna3      GLRWLRGQDKDSIGEDDRRGAAGPAGGQPADTNHRFGRGPRRAQTRDQHRQVMAEQQLLP
Daphnia_magna1      GLRWLRGQDKDSIGEDDRRGAAGPAGGQPADTNHRFGRGPRRAQTRDQHRQVMAEQQLLP
Daphnia_magna2      GLRWLRGQDKDSIGEDDRRGAAGPAGGQPADTNHRFGRGPRRAQTRDQHRQVMAEQQLLP
                    ****************************************************

Daphnia_magna4      -----------------------------------------
Daphnia_magna3      AGEQHPAMLATKCTRADIVTHLGNTKKLQFQQNHQHVEKIG
Daphnia_magna1      AGEQHPAMLATKCTRADIVTHLGNTKKLQFQQNHQHVEKIG
Daphnia_magna2      AGEQHPAMLATKCTRADIVTHLGNTKKLQFQQNHQHVEKIG
```

Supplementary Figure S3. Alignment of inotocin receptors. Alignment of 125 putative inotocin receptor sequences of arthropods. Conserved sequence motifs XPQX_2W$X_{5\text{-}6}$F and CXNPW are highlighted green and grey, respectively. Residues that diverge from the consensus of motif conservation are highlighted in red.

```
                                   1                                                          60
Varroa_destructor1                 ------------------------------------------------------------
Metaseiulus_occidentalis1          ------------------------------------------------------------
Metaseiulus_occidentalis2          ------------------------------------------------------------
Tigriopus_californicus             ----------------------------MATTLLSSDIEAKMDKEPQDNPYNSIP---DS
Anurida_maritima                   ------------------------------------------------------------
Meinertellus_cundinamarcensis      ------------------------------------------------------------
Pogonognathellus_sp                ----------------------------MATN---------------SNTTNEI-----S
Triops_cancriformis                ------------------------------------------------------------
Strigamia_maritima2                ----------------------------MATH-----AEGDSYTRTNPPPTKTIP---MD
Catajapyx_aquilonaris              ------------------------------------------------------------
Daphnia_pulex                      ------------------------------------------------------------
Daphnia_magna3                     -----------------------------------------------------MA---SS
Daphnia_magna2                     -----------------------------------------------------MA---SS
Daphnia_magna1                     -----------------------------------------------------MA---SS
Daphnia_magna4                     -----------------------------------------------------MA---SS
Arachnocampa_luminosa              ---------------------------------------MTSYQENILP-N----YSEQD
Calanus_finmarchicus               ------------------------------------------------------------
Epiophlebia_superstes              ------------------------------------------------------------
Ladona_fulva                       ------------------------------------------------------------
Cordulegaster_boltonii             ------------------------------------------------------------
Diaphorina_citri                   ------------------------------------------------------------
Pachypsylla_venusta                ------------------------------------------------------------
Acanthocasuarina_muellerianae      ------------------------------------------------------------
Glomeris_pustulata                 ------------------------------------------------------------
Frankliniella_cephalica            ------------------------------------------------------------
Machilis_hrabei                    ------------------------------------------------------------
Carabus_granulatus                 ------------------------------------------------------------
Dendroctonus_frontalis             ------------------------------------------------------------
Lepeophtheirus_salmonis            ------------------------------------------------------------
Caligus_rogercresseyi              ------------------------------------------------------------
Parhyale_hawaiensis                ----------------------------------MLDEPLPTMSEELVL-NVSVNA--SE
Hyalella_azteca                    ------------------------------------------------------------
Strigamia_maritima1                ---------------------------------------MSNE----------SHW----
Conwentzia_psociformis             ---------IPYKMNDFNATDQ--------------NYGFEYF-------LNSTA---SD
Liposcelis_bostrychophila          ---------MNTKAREFGNKGKRKT-STRSQKHLSHNEKMDEEARQSSIPDINLT-----
Tetranychus_urticae                -----------MDLSSFGSLEF----STL--SVFSTNETSSTLPSLVLPFDVNLTSSRYL
Osmylus_fulvicephalus              ---------------------R----TTM--ALMNNSVGMSGVPTPSLELVGVLAAGGGG
Ornithodoros_turicata              ------------------------------------------------------------
Acerentomon_sp                     ----------------------------M--ANQSLN-----VGDPWI--NETQVAEHVL
Ixodes_ricinus                     ---------------------------------------MDLV----A--NQDQPAF-ST
Folsomia_candida                   ----------------------------------------------------PLHED-NF
Limulus_polyphemus1                ---------------------------------------MSTL----DF-GETPEIL-NV
Limulus_polyphemus3                ---------------------------------------MSSL----KE-EQTTELT-NA
Platynothrus_peltifer5             ---------------------------------------METVSDLITF-SREQEF--DF
Platynothrus_peltifer1             ---------------------------------------MEEIGTTVTL-EE--------
Platynothrus_peltifer3             ------------------------------------------------------------
Achipteria_coleoptrata2            ----------------------------M--SSDSSDMSVENISQTIIF-QI--------
Platynothrus_peltifer2             ---------------------------------------ME-VSTAIS--EE--------
Platynothrus_peltifer4             ---------------------------------------MSNVIMSI---NN--------
Ixodes_scapularis2                 ------------------------------------------------------------
Ixodes_scapularis1                 -------------------MDV-P---SNQKPEFAASVP---------------------
Ixodes_scapularis3                 -------------------MDLVA---NQDQPAFSTSLP---------------------
Cotesia_vestalis                   ------------------------------------------------------------
Microplitis_demolitor              ------------------------------------------------------------
Zorotypus_caudelli                 ------------------------------------------------------------
Nasonia_giraulti                   ------------------------------------------------------------
Nasonia_vitripennis                ------------------------------------------------------------
Varroa_destructor2                 ------------------------------------------------------------
Leuctra_sp                         ------------------------------------------------------------
Tricholepidion_gertschi            ------------------------------------------------------------
Perla_marginata                    ------------------------------------------------------------
Crioscolia_alcione                 ---------------------------------------------------ME-------
Mischocyttarus-_flavitarsis        ------------------------------------------------------------
Polistes_metricus                  ------------------------------------------------------------
Polistes_canadensis                ------------------------------------------------------------
Chrysis_viridula                   ------------------------------------------------------------
Argochrysis_armilla                ------------------------------------------------------------
Chyphotes_mellipes                 ------------------------------------------------------------
```

```
Brachycistis_timberlakei            ---------------------MKNFCETLRESHRGPSCPRE-QTGLHD--NPG-------
Sphaeropthalma_orestes              ------------------------------------------------------------
Homalodisca_vitripennis             ------------------------------------------------------------
Cercopis_vulnerata                  ------------------------------------------------------------
Nilaparvata_lugens                  --------------------------------------------MIEA--NYT-------
Achipteria_coleoptrata1             ------------------------------------------------------------
Ephemera_danica                     ------------------------------------------------------------
Leptopilina_clavipes                ------------------------------------------------------------
Ganaspis_sp                         ------------------------------------------------------------
Leptopilina_boulardi                ------------------------------------------------------------
Mantis_religiosa                    ------------------------------------------------------------
Blattella_germanica                 ------------------------------------------------------------
Zootermopsis_nevadensis             ------------------------------------------------------------
Stigmatomma_oregonense              -------------------------MSYDP---INSSSPS--PSP---------------
Dinoponera_quadriceps               ---------------------------------------M--SDDLN-------------
Harpegnathos_saltator               ---------------------------------------M--SYDLN-------------
Cerapachys_biroi                    -------------------------MSYDL---SSSSLPL--SLSLP-------------
Monomorium_pharaonis                ---------------------------------------M--SRDLN-------------
Tetramorium_bicarinatum             ---------------------------------------M--LHDFN-------------
Solenopsis_invicta                  ---------------------------------------M--SHDLN-------------
Atta_cephalotes                     ---------------------------------------M--SHDLN-------------
Acromyrmex_echinatior               ---------------------------------------M--SHDLN-------------
Pogonomyrmex_barbatus               ---------------------------------------M--SHDWN-------------
Wasmannia_auropunctata              ---------------------------------------M--SHDLN-------------
Vollenhovia_emeryi                  ---------------------------------------M--SRDLN-------------
Orussus_abietinus                   ---------------------------------------------------MI-----H-
Blaberus_atropos                    ------------------------------------------------------------
Limulus_polyphemus2                 -------------------------MSTLAKGETTGFLNL--------------------
Isonychia_bicolor                   ------------------------------------------------------------
Occasjapyx_japonicus                ----------------------------------------------------------LE
Baetis_sp                           ------------------------------------------------------------
Panonychus_ulmi                     ------------------------------------------------------------
Frankliniella_occidentalis          ------------------------------------------------------------
Eurylophella_sp                     ------------------------------------------------------------
Telenomus_podisi                    ------------------------------------------------------------
Aposthonia_japonica                 ------------------------------------------------------------
Haploembia_palaui                   ------------------------------------------------------------
Halyomorpha_halys                   MFLYFRSLYTETRMVAFSSMGDASYSDYADKDNITSTVPS---RVLC-------------
Stenobothrus_lineatus               ------------------------------------------------------------
Inocellia_crassicornis              ------------------------------------------------------------
Tenthredo_koehleri                  --------------------------------------------MSNS--TAE-----NR
Corydalus_cornutus                  ------------------------------------------------------------
Chrysopa_pallens                    ------------------------------------------------------------
Meloe_violaceus                     ---------------------------------------MHFNQDYQR--NMN-----I-
Aethina_tumida                      --------------------------------------------MYGL--KMN-----V-
Gyrinus_marinus                     ------------------------------------------------------------
Tribolium_castaneum                 --------------------------------------------MYTP--KLS-----QM
Nicrophorus_vespilloides            ----------------------------------------------------------HH
Aleochara_curtula                   ------------------------------------------------------------
Pogonus_chalceus                    ------------------------------------------------------------
Sipyloidea_sipylus                  -------------------MGFNRNFSLPDKSENMVQSPLHVLEMFAA--NNY-------
Extatosoma_tiaratum                 -------------------MCVSRNCT----SEDSDMPPLQVLDVFVG--NYS-------
Aretaon_asperrimus                  -------------------MDVSRNCSSAA-PEEGDQHSLQILDEIVG--NCS-------
Medauroidea_extradentata            -------------------MGDGRNCSLAT-SEDWCQLPN----MLVA--NHS-------
Ramulus_artemis                     -------------------MGASSNCSYGA-TEDWRQPSLD---MLAA--NFS-------
Galloisiana_yuasai                  ------------------------------------------------------------
Athalia_rosae                       --------------------------------------------MLCG--SGM-------

                                    61                                                       120
Varroa_destructor1                  ----------------------------------------MAGNIVVLVLVLQSKPKS--
Metaseiulus_occidentalis1           ---------MNSSAGNFGDARDETIAHVRITVLCFCLLFTLGGNLFVFLLVTRNRPKS--
Metaseiulus_occidentalis2           ------MEY-NITDTDFVPQPENWVLSVKCATLVIIFFLTLSSNLFVLHAVLLRNRQN--
Tigriopus_californicus              NETKGSLDLNFTTDGGPDFSRDQDLATAEVVVQAVIFVLALVGNGLVLVA-LIRQLRR--
Anurida_maritima                    ------------------------------------------------------------
Meinertellus_cundinamarcensis       ------------------------LAIVEVTVLAVVLFVALAGNTMALFS-LRLLRRL--
Pogonognathellus_sp                 EF-----PENKSAAAVPDSGRNESLANIEIAVLSVILFFAVVGNGLMLLA-LRRQLQF--
Triops_cancriformis                 ------------------SARDEKLAIAEIATLAVIFLLALSSNVFMLVA-IWRQRQY--
Strigamia_maritima2                 DN---NPNYNNKTTDGIMDIRDENLASIEIAVQSIILVVAIISNSFVFAA-LCCQRRF--
Catajapyx_aquilonaris               -------LRDNVSGLVEQDSRDETLAAVEIATLAIILLVAILGNMFMVVG-IWRQLQF--
Daphnia_pulex                       -M---STNMSNATEEPSSSLRDEKLASVEISTLSVILVLAVTSNLTMLIA-IWRQRRN--
Daphnia_magna3                      SL---LSNVTNVTTELASSSRDEKLASIEIGTLSFILLLAVTSNLTMLIA-IWRQRRN--
Daphnia_magna2                      SL---LSNVTNVTTELASSSRDEKLASIEIGTLSFILLLAVTSNLTMLIA-IWRQRRN--
Daphnia_magna1                      SL---LSNVTNVTTELASSSRDEKLASIEIGTLSFILLLAVTSNLTMLIA-IWRQRRN--
Daphnia_magna4                      SL---LSNVTNVTTELASSSRDEKLASIEIGTLSFILLLAVTSNLTMLIA-IWRQRRN--
Arachnocampa_luminosa               KFENSNVSLNNSNSVQQFQLNSSLLTKIEVFLLSSLFLLTVIGNLIVILI-ILLYRNMSP
Calanus_finmarchicus                ------------------------------------------------------------
```

```
Epiophlebia_superstes               ------------------GGRDEELAKAEIATLAAMLAVSVIGNGAVLVA-LWVRPRPSP
Ladona_fulva                        -----------------------------MATLAAMLVISIVGNGAVMVA-LWARPRSSP
Cordulegaster_boltonii              -----------------------------MATLAAMLVVSVVGNGAVLIA-LWARPRPSP
Diaphorina_citri                    ------------------------------------------------------------
Pachypsylla_venusta                 --MDESNGTLVNYNDTDSDERDEDLARVEILTLLIILVITIVGNIMVLFA-LYSRRHFQK
Acanthocasuarina_muellerianae       ------------------------------------------------------------
Glomeris_pustulata                  ------------------------------------------------------------
Frankliniella_cephalica             ------------------------------------------------------------
Machilis_hrabei                     ------------------------------------------------------------
Carabus_granulatus                  ------------------------------------------------------------
Dendroctonus_frontalis              ------------------------------------------------------------
Lepeophtheirus_salmonis             ------VSYNTSLNTTIPSSSPNSLLHTKLWVLGIIIVLTILGNSIVILA-IATRF----
Caligus_rogercresseyi               ---------------------EGNSLHQTKLWVLGIIIVLTILGNSIVILA-IATRH----
Parhyale_hawaiensis                 DAINFD--ILSVQNNTSFGSRDEVLAAFEVLTLASIFVVTIVGNCIVIFK-HFMRR----
Hyalella_azteca                     ------------------------------------------------------------
Strigamia_maritima1                 --EDDL--TMDNATSNAPPPQSSAIADAKLTVLALMFATIMLGNTIVITS-LLIRR----
Conwentzia_psociformis              AVIEAG--TDLAAASDNGDERNEELAKIEILILIVIFNVTLIGNLVVLLA-LSARKRYSG
Liposcelis_bostrychophila           --ADPTMELVRKNDSTDDFGRNEDLAQIEIGTLGVILIVTILGNGLVLLA-LYAKKCKAG
Tetranychus_urticae                 KAVSTGSPSASSLSSSSAHSRDENLAILEIITLSAIFVMIVFGNTSVLLA-LILSR----
Osmylus_fulvicephalus               GLLLPVDPTSANSTAMHDYDRDEALAQVEIGTLALIFGVTLLGNGAVLIA-LYIRRRYSG
Ornithodoros_turicata               ------------------------------------------------------------
Acerentomon_sp                      NVGDPWTNETQEAEDGFKYERNEDLARIEIAILAAIFCATVIGNVLVLVA-LFNTK----
Ixodes_ricinus                      SLPDPL-DDVPVNDTSWTGTRDESLAQIELGVLTAIFTFTVIGNTTVLAA-LAARR----
Folsomia_candida                    SLTPNL-SL-VNTTLTPASPRDEFLAKIEQSVLAIIFILTIVGNFLVLMG-IYFRK----
Limulus_polyphemus1                 SFMNSS-TI-INNTKDKGIQRDETIARIEVGVLASIFTLTILGNMCVLVA-LAVRR----
Limulus_polyphemus3                 SFVNFS-PI-E-NITVGDTQRDETIARVEVGVLAIIFILTIIGNTCVLVA-LAVRR----
Platynothrus_peltifer5              ELLNDS-NNVNKSSFEIRDSRNESLAIIEISLLTFMFISILIGNIFVLTA-LIVRD----
Platynothrus_peltifer1              --LEDK-LPETIIANVTKDSRDEHLAVFEIATLGLIFLLIVFGNSCVLIA-LIMRK----
Platynothrus_peltifer3              --------M--TGNGSLTVKRDEKWVLLEIGILSLIFILIVFGNSCVLIA-LQVRK----
Achipteria_coleoptrata2             --INDD-NM--TANTSLAVKRNEKLVLLEIGILLLIFLLIVFGNSCVLIA-LQINK----
Platynothrus_peltifer2              --TDGI-PFETSSIKPNITGRNENLALIEVGTLGTIFVLIVFGNCCVLLA-LALRR----
Platynothrus_peltifer4              --TTSV-LYN----ISGSDKRDENLAIMEVSILALIFILIIIGNLCVLIA-LAVKR----
Ixodes_scapularis2                  --------------------------QVEVAVLTFIFGFTVLGNTCVLVA-LAAR-----
Ixodes_scapularis1                  ----TVLNFASTNLTASTETRDESLALVELAVLTLIFAFTVMGNSTVLLA-LATR-----
Ixodes_scapularis3                  ----DSLDDVPVNETFWTGTRDESLAQIELGVLTAIFTFTVIGNTTVLAA-LAAR-----
Cotesia_vestalis                    ------------DNSTSLDTRDENLAFWEIIVLVINLIITLIGNCFIFFA-LYLRRYH--
Microplitis_demolitor               ----------MMGNSTQPDTRDENLAIWEITVLAINLIITTIGNCLIFFA-LYLRRYH--
Zorotypus_caudelli                  -------------------------------LLVIILLVTLVGNGIVLFA-IYVRRCR--
Nasonia_giraulti                    ----MEESATSMIAVPAEDWRDESLAVWEVIVLVLILTTTLMGNVLVLFA-IYLKRCR--
Nasonia_vitripennis                 ----MEESATSMIAAPAEDWRDESLAVWEVIVLALILTTTFMGNVLVLFA-IYLKRCR--
Varroa_destructor2                  -----------------------------------------------------------E
Leuctra_sp                          ------------------------------------LVTAVVANLLVLTA-LILRS----
Tricholepidion_gertschi             ------------------------------------------------------------
Perla_marginata                     ----------------------------EVTSLAAIFLTALAGNVCVLLA-LYGHR----
Crioscolia_alcione                  G------MPEDQNSSEEIDGRDEYLAKWEITVLTSILLLTLLGNTLVLVS-LYIRKRY--
Mischocyttarus-_flavitarsis         ------------------------------------------------------------
Polistes_metricus                   --MNETTNSSFTAANEEEDIRDENLAKWEIVLLANILIFTIFGNSLVLLS-LYLRRYR--
Polistes_canadensis                 --MNETTNSSFTVANEEKDTRDENLAKWEIVLLANILIFTIFGNSLVLLS-LYLRKYR--
Chrysis_viridula                    -MSV---EQNLNVTETEEDVRDEYLAKWEIIVLATILYMTLVGNSLLLFA-LYLKRHR--
Argochrysis_armilla                 ------------------------------------------------------------
Chyphotes_mellipes                  -MYRDRNATARGCTAGERDERDEYLARWEIAVLVTILVVTILGNGLVLLS-LHVRRHR--
Brachycistis_timberlakei            DMFADRNSSEVQTSDELDDERDEELAKWELVVLASILVITLVGNGLVLLA-LYARRYH--
Sphaeropthalma_orestes              -MYNDRNNT-TTLGEAKDDTRDEELAKWEIGVLTSIFIITILGNSLVLFA-LYIRRRR--
Homalodisca_vitripennis             ---MAND-LVFNSSLASDLERDEELARAEIAVLAVIFVVTVTGNTAVLVA-LYARRFC--
Cercopis_vulnerata                  ----------------------EELARAEIAVLAAIFVITVTGNTVVLVA-LYGRRFC--
Nilaparvata_lugens                  ---FSTERVNESVVAVSNGGRDEDLARVEVATLAAILAITLAGNSTVLVA-LYMRRTW--
Achipteria_coleoptrata1             ----ENITEELKNNTTVSDTRDEHLAIFEIATLSLLFVLILFGNSCVLLA-LLMR-----
Ephemera_danica                     -----------------------------IATLAIIFGVTVLGNSCVLAA-ISFR-----
Leptopilina_clavipes                ------------------------------------------------------------
Ganaspis_sp                         ----------MENGTLINDTRDEELAKWEIGILACFFVIIIAGNSLVLFS-IYLKRYY--
Leptopilina_boulardi                ----------MENGTLINDTRDEELAKWEIGILACLFVIIIIGNSLVLFS-IYLKRHY--
Mantis_religiosa                    ------------------------------AILSTLFVVIVLGNSSVLLA-LSSR-----
Blattella_germanica                 --MENASQAVNGSTTAEDLDRDEVLARAEIATLSVMFTVTVVGNFAVLLV-LSAR-----
Zootermopsis_nevadensis             --EVSEACNLTTANSTSGLDRDEALAQAEIATLATMLTVTVLGNFAVLLA-LSAR-----
Stigmatomma_oregonense              ---LSLSLPSSSSERTSNDARDEYLARWEIAVLTSIFLITLIGNALVLFA-LYVRRRH--
Dinoponera_quadriceps               --STS--SPPSASEQTPVDGRNEDLAKWEIALLTSIFLITLIGNTLVLLA-LYARKRR--
Harpegnathos_saltator               --SSSLSLP-PSEQKTSVDARDEYLAKWEIAVLTSIFLITLIGNALVLFA-LYVRRRY--
Cerapachys_biroi                    --SSPSSSSSPSSLEVSDDLRDEYLARWEIAVLTSIFLITIIGNGLVLFA-LYARRCY--
Monomorium_pharaonis                --S-----SSSPSEWPLDDARDEYLARWEIAVLTSIFLLTVIGNILVLLA-LYVRRRY--
Tetramorium_bicarinatum             --SSSLTSPSPPSPLPPDDARDEYLARWEIAVLTSIFLVTLIGNSLILLA-LYARRRY--
Solenopsis_invicta                  --SSS--SPPSSSEWPPDDARDEYLARWEIAVLTSIFLVTLIGNSLVLLA-LYARRRY--
Atta_cephalotes                     --SSS--SSEL---PLDDDARDEYLARWEIAVLTSIFLITIIGNVLVLLA-LYARRRY--
Acromyrmex_echinatior               --SSS--SSEL---PLDDDARDEYLARWEIAVLTSIFLITIIGNVLILLA-LYARRRY--
Pogonomyrmex_barbatus               --S-----S-LSSEWPPDDVRDEYLARWEIAVLTSIFLTTLIGNSLVLLA-LYTRRRY--
Wasmannia_auropunctata              --SSSSSPP-LSSDRPQDDARDEDLARWEIAVLTSIFLVTLIGNALILLA-LYVRRRY--
Vollenhovia_emeryi                  --SSSSTSP-SLSEWPSNDARDEYLAKWEIAVLTSIFLVTLIGNALVLLA-LYIRRRY--
Orussus_abietinus                   -EDEMDAEDPPTTTNPVEDFRDSDLARWEIFTLAAILLAILLENSLVLLA-LYLR-----
```

```
Blaberus_atropos                  ------------------------------------------------------------
Limulus_polyphemus2               SF--VNLSTITNNTKADVVQRDETVARIEVAVLATIFTLTVIGNTIVLVA-LVVR-----
Isonychia_bicolor                 -----------------------------------------VGNTCVLIA-IRHR-----
Occasjapyx_japonicus              SPRMNASSNASATGGMNGYERNEDLAKVEIAILSTIFVLTVIGNSMVLVA-IFLK-----
Baetis_sp                         --MDSSVDNSTTTTVDAVPTRDEQLARVEIATLAFIFAVTVIGNSLILSA-ILFRR----
Panonychus_ulmi                   --------------------RDENLAIIEIITLSAIFVMIVFGNTSVLLA-LILS-----
Frankliniella_occidentalis        ------------------------APQVEVAVLGVLLVLTVLGNAVVLLA-LYARRRC--
Eurylophella_sp                   ------------------------------------FVVTTIGNSCVLVA-LSLR-----
Telenomus_podisi                  -----MPVESQTVKNVTEDFRDESLAQWEVGVLAAIFVITIIGNSLVLLA-IYLRRYR--
Aposthonia_japonica               ---------MNSSAEKDSDGRDEDLARVEVTVLAIIFAITLVGNLLVLVA-LLVL-----
Haploembia_palaui                 -MVHRKRQNDVMNSSVASDGRDEDLARVEVTVLAIIFAITLVGNLLVLLA-LLVL-----
Halyomorpha_halys                 --------PNDSINCSAVHERDEDLAKIEMATLAVMFVMTVVGNGAVLAA-LFARRQS--
Stenobothrus_lineatus             ------------------------------------------------------------
Inocellia_crassicornis            ---MNGTELIKSAPETNGNERDEVLAQAEIITSAVIFVVTVIGNGTVLLA-LFARQKY--
Tenthredo_koehleri                SF--VESECNLTISSEIDDSRDEYLAYWEVATLALIFVVAIAGNSLVLTA-FYFRRYD--
Corydalus_cornutus                ------------------------------------------------------------
Chrysopa_pallens                  -----------------------------ILTLAIIFVVTLLGNGAVLLA-LYNRQKC--
Meloe_violaceus                   -SIENTTKSLRDDTEESPTDRDESLARIEIATLAVIFLITVIGNSTVLIA-LWARRRF--
Aethina_tumida                    ----TSTGETIEELERAAKERDESLAKIEVAVLAVIFIVTVIGNSTVILA-LWARRRC--
Gyrinus_marinus                   -------MNTTANATDQTFDRDENLAQFEIATLAVIFVVTVIGNGTVLLA-LWTRRRC--
Tribolium_castaneum               DISENSTYLFDKHEDRNNTDRDENLARVEVATLAIIFLVTVIGNSTVLLA-LWTRRRY--
Nicrophorus_vespilloides          SL--RGSPSSEREERGSSSHRDCN----------------FSHHLHSHHX-LWTRRRY--
Aleochara_curtula                 --------------------------------------------------------RY--
Pogonus_chalceus                  ------------------------------------------------------------
Sipyloidea_sipylus                SVDNGNGNSNGSNSTSSGYDRDEDLARAEIATLAVMLLVTVIGNSCILYA-LCARR----
Extatosoma_tiaratum               ------AE--NSTNSSSGYDRDEDLARGEIATLAIMFIVTVVGNSCILFA-LCARR----
Aretaon_asperrimus                ------GSSANITNSTGGYDRDENLARAEIATLAVMFIITVIGNSCILYA-LCLRK----
Medauroidea_extradentata          ------VK--NSTNSTDGYDRDEDLAHAEIATLAVMFIITMVGNSCILYA-LCARM----
Ramulus_artemis                   ------AENSSSANSTYGYDRDENLAHAEIATLATMFAITLVGNFCILYA-LCARM----
Galloisiana_yuasai                ------------------------------------------------------------
Athalia_rosae                     CA--TANEEALKVDDGNRDTRDEDIANWEVATLAAMFVVAVFGNSLVVTA-LYLRRYGKT
```

121 **$XPQX_2WX_{5\text{-}6}F$ motif** **180**

```
Varroa_destructor1                ---------AHLSRIYYFLLHLSIADILVGIFNISPQLVWDIY-FRFPLG-NFACKIVKF
Metaseiulus_occidentalis1         ---------VHQLHIYHFMVHLSLADMLVGVFNILPQIVWEIY-FRFRWG-NIACKFVKF
Metaseiulus_occidentalis2         ---------RPLSRVQLFMLHLTVADILVALLNILPQLAWDLT-IQFRGG-AVLCKLVKF
Tigriopus_californicus            ---------KPSSRMYRLMYHLSIADLLVAVLNVLPQLIWDIT-HRFHGP-DILCRLVKF
Anurida_maritima                  -------------------------------------------------S-DFLCRLIKF
Meinertellus_cundinamarcensis     ---------RPLSRMYFFMQQLSIADLMVAVFNVLPQLIWDIT-YRFKGG-DVLCRLVKF
Pogonognathellus_sp               ---------RPMSRMYFFMLNLSVADLMVAFGNILPQLAWDIT-YRFKGN-DLLCRVVKF
Triops_cancriformis               ---------RPLSRMYFFMLHLSLADVLVAVFNILPQLLWDIT-YRFLGG-DLLCRFVKY
Strigamia_maritima2               ---------RPTSRMYLFMLHLSVADLLVAFLSLLPQLIWDVT-FRFQGS-DILCRVVKY
Catajapyx_aquilonaris             ---------RPMSRMYFFMLHLSVADLLVAVFNILPQLLWDIT-YRFQGS-DALCRTVKY
Daphnia_pulex                     ---------RPLSRMYFFMMHLSLADLLVALFNILPQLAWDIT-YRFQGG-DVLCRFVKY
Daphnia_magna3                    ---------RPLSRMYFFMMHLSLADLLVALFNILPQLAWDIT-YRFHGG-DVLCRFVKY
Daphnia_magna2                    ---------RPLSRMYFFMMHLSLADLLVALFNILPQLAWDIT-YRFHGG-DVLCRFVKY
Daphnia_magna1                    ---------RPLSRMYFFMMHLSLADLLVALFNILPQLAWDIT-YRFHGG-DVLCRFVKY
Daphnia_magna4                    ---------RPLSRMYFFMMHLSLADLLVALFNILPQLAWDIT-YRFHGG-DVLCRFVKY
Arachnocampa_luminosa             KRARWFKLNKNISRMSFYIIHLSIADFNVAFMSILPQLIWRQS-VVFNHS-HFLCKFVAF
Calanus_finmarchicus              ------------------------ADILTAIFTLIPEIAWTLTLPAFWGG-DAVCKIIKF
Epiophlebia_superstes             --------RRRLSRMHLLVLYLCTADLITAAFTVLPQMAWEAT-YRFAPQAAPLCKPLKV
Ladona_fulva                      --------RRRLSRMHLLVLHLCTADLITAAFTVLPQMAWEVT-YRFAPQAAPLCKPLKV
Cordulegaster_boltonii            --------RRRLSRMHLLVLHLCTADLITAAFTVLPQMAWEAT-YRFAPEAAPLCKPLKV
Diaphorina_citri                  ----------------------------------------------------------MV
Pachypsylla_venusta               --------QIKMPRMYYFILHLSIADLITAFFNVLPQLWWDIN-YRFPGSSNWLCKLVKF
Acanthocasuarina_muellerianae     ------------------------------------------------------------
Glomeris_pustulata                ------------------------------------------------------------
Frankliniella_cephalica           ------------------------------------------------------------
Machilis_hrabei                   ------------------------------------------------------------
Carabus_granulatus                ------------------------------------------------------------
Dendroctonus_frontalis            ------------------------------------------------------------
Lepeophtheirus_salmonis           ---------IKMTRMYFFILHLSIADVLTAFLTLLPELIWTYTSPNFYGG-AFLCKAVKF
Caligus_rogercresseyi             ---------IKMTRMYFFILHLSIADVLTAFLTLLPELIWTYTSPNFYGG-GALCKIVKF
Parhyale_hawaiensis               ---------KKFRRMNYFIFALSLSDLVTAMFNVLPQLIWDVT-FRFKGG-NVLCKCVKF
Hyalella_azteca                   ------------------------SDLVTAAFNVFPQLMWDIT-FRFRGG-DFLCRSVKF
Strigamia_maritima1               ---------KKLTRMYFFILHLCIADLIVAFLHVLPQLCWDVT-HRFQGN-DFLCKVIKF
Conwentzia_psociformis            --------HRKLTRMFFFILHLSLADLISAFLNVLPQMIWK-------------------
Liposcelis_bostrychophila         --------KKTLSRMYFYIFHLCVADLITAFLNVLPQLFWDIT-YRFKGN-QFLCKSVKY
Tetranychus_urticae               ---------IKLRRMYFFLLHLSIADLITAFANVLPQLIWEIT-ERFYGG-NVLCKGIKY
Osmylus_fulvicephalus             --------RKKLSRMYFFILHLSIADLVTAFFNVLPQLAWEVT-YRFRGG-GVLCKAVKF
Ornithodoros_turicata             ------------------------------------------------------------
Acerentomon_sp                    ---------KKMTRMYYFMMHLSIADLFTAFFNVLTQLIWEIT-YRFAGG-NYLCKFVKY
Ixodes_ricinus                    ---------SKMSRMYYFLLHLCVSDLITAFFTVLPQLGWDAT-YRFRGG-NLACKTVKF
Folsomia_candida                  ---------QKMTRMYYFIAHLAIADLTTAFFQVLTQLAWEVT-YRFQGG----------
Limulus_polyphemus1               ---------IKMARMYYFLLHLCISDLITAFFNILPQLAWDVT-YRFYGG-NILCKVIKY
Limulus_polyphemus3               ---------TNMTRMYYFLLHLCISDLITAFFHVLPQLAWDAA-HRFYGG-NVLCKVVKY
```

```
Platynothrus_peltifer5          ---------SKMNRMYYFLFHLCIADLITAFFNVLPQLIWDIT-YRFYGG-NVLCKLIKY
Platynothrus_peltifer1          ---------LKMNRMYYFLLHLCIADLITAFFNVLPQLAWDIT-YRFYGG-NILCKTIKY
Platynothrus_peltifer3          ---------LKMNRMYYFLLHLCIADLITAFFNVIPQLAWEIT-HRFYGG-NFLCKTIKY
Achipteria_coleoptrata2         ---------LKMNRMYYFLQHLSISDMITAFFNVLPQLAWEIT-HRFYGG-NILCKTIKY
Platynothrus_peltifer2          ---------LKMTRMYYYLLHLCISDLMTGFFTVLPQLAWDIT-YRFNGG-NILCKAVKS
Platynothrus_peltifer4          ---------FKMTRMYYFLLHLCISDIITGCFTVMPQLAWDIT-HRFNGG-NILCKLVKC
Ixodes_scapularis2              --------RSKMTRMYYFLIHLCVSDLTTAFLSVLPQLGWDAT-HRFRGG-NLVCKAVKF
Ixodes_scapularis1              --------RSKMTRMYYFLLHLCVSDLITAFLTVLPQLGWDAT-YRFQGG-NLACKAVKF
Ixodes_scapularis3              --------RSKMSRMYYFLLHLCVSDLITAFFTVLPQLGWDAT-YRFRGG-NLACKTVKF
Cotesia_vestalis                ------GRQRKLTRMYFFMMHLSIADFITGIFNILPQLSWDIT-FRFQGG-PAMCKIVKF
Microplitis_demolitor           ------GRQRKLTRMYFFMLHLSIADFVTGIFNVLPQLAWDIT-FRFQGG-YGLCKIIKF
Zorotypus_caudelli              ------GRRHRLTRMHFFVMHLSIADLINGLLNVLPQLAWDIT-FRFQGG-QILCKMVKF
Nasonia_giraulti                ------GRRQRLTRMHFFVMHLSVADLITGLLNVLPQLAWDVT-FRFQGG-PILCKLVKF
Nasonia_vitripennis             ------GKRQRLTRMHFFVMHLSVADLITGLLNVLPQLAWDVT-FRFQGG-PILCKLVKF
Varroa_destructor2              A-----RRSSSLSRVQLFMMHLSIADILVALLNILPQLAWDIT-ARFYGG-ALLCKFVKY
Leuctra_sp                      -------RRQRLARLHFFILHLSLADLVNALFNVLPQLVWKST-FRFVGG-TVLCKLVKF
Tricholepidion_gertschi         ------------------------------------------------------------
Perla_marginata                 --------NRKLTRMHFFILHLTIADMVTAVFSVMPQMLWKAT-YRFAGG-PTLCKVVKV
Crioscolia_alcione              ------ER-RKFTRMYFFIMHLSVADMLTGLLNVLPQLAWDIT-FRFQGG-ALLCKAVKF
Mischocyttarus-_flavitarsis     -----------------------------------------------QGG-MVLCKLVKF
Polistes_metricus               ------AH-KKLSRMYFFIMNLSIADLLTGLLDVLPQLAWDIT-FRFQGG-LVLCKLVKF
Polistes_canadensis             ------AH-KKLSRMYFFIMNLSIADLLTGLLDVLPQLAWDIT-FRFQGG-LVLCKLVKF
Chrysis_viridula                ------GRRRKFTRMHFFILHLSTADLITGLFNVLPQLAWDIT-FRFQGG-NILCKLVKF
Argochrysis_armilla             ------------------------------------------------GG-NVLCKLVKF
Chyphotes_mellipes              ------QR-RKFTRMYFFIMHLSVADLLTGLLNVLPQLAWDIT-FRFKGG-PILCKLVKF
Brachycistis_timberlakei        ------CR-RKFTRMYFFIMHLSIADLLTGLLDVLPQLAWDIT-FRFQGG-PVLCKMVKF
Sphaeropthalma_orestes          ------GR-RKFTRMYFFIMHLSVADLITGLCDVLPQLAWDIT-FRFQGG-PVLCKIVKY
Homalodisca_vitripennis         ------TGNKKLPRMYFFILHLCVADLTTGLLNVFPQLCWKIT-YRFKGG-PMLCKVVKY
Cercopis_vulnerata              ------GGRKKLSRMYYFILHLCIADLVTALLNVFPQLIWDIT-FRFKGG-PFLCKLVKY
Nilaparvata_lugens              ------GGRKKLSRMYYFILHLCIADLVTGLLNVFPQLIWDIT-YRFRGG-PLLCKLVKY
Achipteria_coleoptrata1         --------KIKMNRMYYFLLHLIIADYLVAFFNVLPQLIWDIT-YRFYGG-NLLCKVIKY
Ephemera_danica                 --------RAKISRMYFYIMHLSVADLITAFFNVLPQLAWEIT-YRYCLS--QLCRGVKF
Leptopilina_clavipes            -----------------------VADLLTAVLNVLPQMAWKIT-FRFQGG-PFLCKLIKF
Ganaspis_sp                     ------SQRKKLTRMHFFIMNLSVADLLTAILNVLPQMAWKIT-FRFQGG-PILCKLIKF
Leptopilina_boulardi            ------GQRKKLTRMHFFIMNLSVADLLTAILNVFPQMVWKIT-FRFQGG-QILCKLIKF
Mantis_religiosa                -------RRKKLSRMYYFILHLSIADLITALLSVLPQLAWDIT-YRFHGG-PLLCKLIKY
Blattella_germanica             -------PRKKLSRMYYFILHLSIADLCTAFLSILPQLAWDVT-YRFRGG-PIMCKVVKY
Zootermopsis_nevadensis         -------PRKKLSRMYYFILHLSVADLCTAFLSVLPQLAWDVT-YRFYGG-PLLCKIVKY
Stigmatomma_oregonense          -------QRRKFTRMYFFILHLCVADLLTDLLNVLPQLAWDIT-FRFQGG-AVLCKLIKF
Dinoponera_quadriceps           -------QRRKFSRMYFFILHLCIADLLTGLFDVLPQLAWDIT-FRFQGG-AVLCKLIKF
Harpegnathos_saltator           -------QRHKFTRMYFFILHLSIADLLTGLLDVLPQLAWDIT-FRFQGG-AVLCKLIKF
Cerapachys_biroi                -------QRRKFTRMYFFILHLSIADLLTGLLDVLPQLAWDIT-FRFQGG-AVLCKLIKF
Monomorium_pharaonis            -------QRRKFTRMYFFILHLSVADLLTGLLDVLPQLAWDIT-FRFQGG-AVLCKLIKF
Tetramorium_bicarinatum         -------QRRKFTRMYFFILHLSVADLLTGLLDVLPQLAWDIT-FRFQGG-AVLCKLIKF
Solenopsis_invicta              -------QRRKFTRMYFFILHLSVADLLTGLLDVLPQLAWDIT-FRFQGG-AVLCKLIKF
Atta_cephalotes                 -------QRRKFTRMYFFIMHLSVADLLTGLFDVLPQLAWDIT-FRFQGG-AVLCKLIKF
Acromyrmex_echinatior           -------QRRKFTRMYFFIMHLSVADLLTGLFDVLPQLAWDIT-FRFQGG-AVLCKLIKF
Pogonomyrmex_barbatus           -------QRRKFTRMYFFILHLSIADLLTGLLDVLPQLAWDIT-FRFQGG-AVLCKLIKF
Wasmannia_auropunctata          -------QRRKFTRMYFFILHLSVADLLTGLLDVLPQLAWDIT-FRFQGG-AVLCKLIKF
Vollenhovia_emeryi              -------QRRKFTRMYFFILHLSVADLLTGLLDVLPQLAWDIT-FRFQGG-AALCKLIKY
Orussus_abietinus               -------GRRKLSRIQFFILHLSIADLLTGLLNVLPQLAWDIT-FRFQGG-DLLCRLVKF
Blaberus_atropos                ------------------------------------------------------------
Limulus_polyphemus2             --------RIKMTRMYYFLQHLCISDLITAFFHVLPQLAWDVT-YRFYGG-NVLCKIVKY
Isonychia_bicolor               --------RSKITRMYFYIFHLSIADLVTAFLNVIPQLAWEIT-YRFKGG-DELCKIIKF
Occasjapyx_japonicus            --------RKKMSRMYYFILHLSIADLITGFFNVLTQLCWEVT-YRFVGG-NFMCKFIKY
Baetis_sp                       --------RVKITRMYFYIFHLSIADLVTAFFNVLPQLAWELT-YRFKGG-NLLCKGVKF
Panonychus_ulmi                 --------RIKLRRMYFFLLHLSIADLITAFTNVLPQLIWEIT-EHFYGG-QILCKGIKY
Frankliniella_occidentalis      ------GGRRKLSRMFYFILHLSVADMVTAFFSVLPQLVW-VT-FRFHGG-NFLCKAVKF
Eurylophella_sp                 --------RAKISRMYFYILHLSIADLVTAFFNVIPQLAWEVT-YRFRGG-DILCKAVKF
Telenomus_podisi                ------GRRKKLTRMYFFILHLSVADLITAFFDVLPQLAWDIT-FRFQGG-PILCKLVKL
Aposthonia_japonica             -------RRKKLSRMYFFIVHLSIADLITAFFNVLPQLGWDVT-YRFIGG-PILCKIIKF
Haploembia_palaui               -------RRKKLSRMYYFIVHLSIADLITAFFNVLPQLGWDVT-YRFIGG-PILCKVIKF
Halyomorpha_halys               ------ATRTKVPRMNFFMLHLCVADMCTAWLNILPQLAWEVT-FRFKGG-EFLCKSVKF
Stenobothrus_lineatus           --------------------QLSVVDLLNGLLNVLPQLAWEVT-FRWRGG-AVLCKLVKF
Inocellia_crassicornis          ------SGCRKLSRMYFFILHLSIADLVTAFFNVLPQLSWDIT-FRFKGG-PLLCKAVKY
Tenthredo_koehleri              ------APRKKLPRIYFFILHLSIADLLTALLNVFPQLIWEIT-FRFQGG-PVLCKFVKF
Corydalus_cornutus              ---------------------------VTAFFNVLPQLIWEVT-YRFRGG-EILCKAVKF
Chrysopa_pallens                ------TGRKKLSRIYFFILHLSIADLVTAFLNVLPQLAWEIT-YRFQGG-PVLCKAVKF
Meloe_violaceus                 ------AGRKKLSRMYFFILHLSVADLITAFLSVLPQLAWDIT-YRFYGG-YFLCKAIKY
Aethina_tumida                  ------AGRKKLSRMYFFILHLSIADLITAFLSVLAQLAWDIT-YRFRGG-YILCKLVKF
Gyrinus_marinus                 ------AGRKKLSRMYFFILHLSIADLVTAFLNVLPQLAWEVT-YRFRGG-AVLCKLVKF
Tribolium_castaneum             ------AGRKKLSRMYFFILHLSIADLITAFLSVLPQLAWDIT-YRFYGG-FLLCKVVKY
Nicrophorus_vespilloides        ------AGRKKLSRMYFFILHLSIADLITAFLSVLPQLCWDVT-FRFQGG-EVLCKLVKF
Aleochara_curtula               ------AGRKKLSRMYFFILHLSIADLITAFLSDLPQLGWEVT-YRFYGG-GALCKMVKF
Pogonus_chalceus                --------------MYFFILHLSIADLVTAFLNTLPQLGWDIT-YRFNGG-FLLCKIVKY
Sipyloidea_sipylus              -------RSKKLTRMYFFILHLSIADLITGMCNVLPQLAWDIT-YRFKGG-PVLCKIVKF
Extatosoma_tiaratum             -------KRRKLTRMYFFILHLSIADLITGFLNVLPQLAWDIT-YRFQGG-PVLCKIIKY
```

```
Aretaon_asperrimus                  -------RKKKLTRMYFFILHLSIADLISGLLEVLPQLAWDIT-FRFQGG-PVLCKFVKY
Medauroidea_extradentata            -------RHKKLTRMYFFILHLSIADLITGLLNVLPQLAWDIT-YRFQGG-PVLCKIVKY
Ramulus_artemis                     -------RRKKLTRMYFFILHLSIADLTTGLLNVLPQLAWDIT-YRFQGG-PVLCKVVKF
Galloisiana_yuasai                  ------------------------ADLITALFNVLPQLGWEIT-YRFSGG-PVLCKAVKF
Athalia_rosae                       P-----IRKKSSSRMYFFILHLSIADLITAFLNVLPQLFWEIT-YRFQGG-PILCKLVKF


                                    181                                                      240
Varroa_destructor1                  LQVFVLYLSTYVLAGMAVDRYLAIRSGIN-------------------------------
Metaseiulus_occidentalis1           MQIFVLYLSTYTLVGLSLNSSLVVRSGSS-------------------------------
Metaseiulus_occidentalis2           AQVYVLYLSTYILTGMSLDRLITMRAIESQWQTASLRRSDHDQSSDKSPQSHKIKFNNHR
Tigriopus_californicus              GQVMPIYLSSFILTLMAVDRVGVTKRHGS-------------------------------
Anurida_maritima                    LQIFVLYLSTYILISMTLDRYLVVCHHSF-----------------------------SR
Meinertellus_cundinamarcensis       FQVMPLYGSTYVLMFLAVDRYRAVCSAGP-------------------------------
Pogonognathellus_sp                 LQIFVLYLSTYVLTSMAVDRYLVVCHHSF-----------------------------TR
Triops_cancriformis                 GQIMPLYLSTYLLMFMALDRYRAVCSASN------------------------------N
Strigamia_maritima2                 LQVMVLYLSTYILVMMAVDRCRAVCWS-I------------------------------T
Catajapyx_aquilonaris               FQVMVLYLSSYILMFMAVDRYKALCCD-A------------------------------F
Daphnia_pulex                       AQVMTLYLSTYILMFMAVDRYRAVCCR-N------------------------------L
Daphnia_magna3                      ------------------------------------------------------------
Daphnia_magna2                      TQIMTLYLSTYILMFMAVDRYRAVCCR-N------------------------------L
Daphnia_magna1                      TQIMTLYLSTYILMFMAVDRYRAVCCR-N------------------------------L
Daphnia_magna4                      TQIMTLYLSTYILMFMAVDRYRAVCCR-N------------------------------L
Arachnocampa_luminosa               SQVFSVYASTFLLIVMAYDRFKCICWPIK------------------------------S
Calanus_finmarchicus                LQMLGPYLSSFLLCVTSMDRYQAICKPFK------------------------------T
Epiophlebia_superstes               LQLLGPYLSSYLLVAMALDRHRAVCRPLS------------------------------A
Ladona_fulva                        LQLLGPYLSSYLLVAMAIDRHRAVCRPLS------------------------------V
Cordulegaster_boltonii              LQLLGPYLSSYLLVAMAIDRHRAVCRPLS------------------------------V
Diaphorina_citri                    VHNVGSFLSSYILMAIAIDRYRAICHPLT------------------------------Y
Pachypsylla_venusta                 VQPSGSFLSSYILMAIAIDRYRAICQPLT------------------------------Y
Acanthocasuarina_muellerianae       ------------------------------------------------------------
Glomeris_pustulata                  ------------------------------------------------------------
Frankliniella_cephalica             ------------------------------------------------------------
Machilis_hrabei                     ------------------------------------------------------------
Carabus_granulatus                  ------------------------------------------------------------
Dendroctonus_frontalis              -----------------------ICYPLT------------------------------Y
Lepeophtheirus_salmonis             GQMIGPYLSSYVLIMTAIDRYHAICFPFS------------------------------K
Caligus_rogercresseyi               CQMIGPYLSSYVLIMTAIDRYHAICNPLS------------------------------K
Parhyale_hawaiensis                 GQLVGPYLNAYILVVTAIDRYQVICRPLS------------------------------N
Hyalella_azteca                     GQLVGPYMNSYILVVTAIDRYQVICQPLS------------------------------N
Strigamia_maritima1                 GQLLGPYLSSYILVGMAIDRYLAICFPLN-------------------------------
Conwentzia_psociformis              ------------------------------------------------------------
Liposcelis_bostrychophila           G-----------------------------------------------------------
Tetranychus_urticae                 LQILGPYLSSFTLCATAIDRYRAICFPLE------------------------------S
Osmylus_fulvicephalus               S-----------------------------------------------------------
Ornithodoros_turicata               ---------SYVLVVTAVDRYQAICFPLS------------------------------N
Acerentomon_sp                      MQIFGPYLSSYILTVTALDRYRAICFPLQ------------------------------N
Ixodes_ricinus                      GQLLGPYLSSYVLVVTAADRYQAICFPLS------------------------------N
Folsomia_candida                    ------------------------------------------------------------
Limulus_polyphemus1                 LQILGPYLSSYVLMVTAIDRYHAICFPLR------------------------------N
Limulus_polyphemus3                 LQILGPYLSSYVLMVTAIDRYQAICFPLT------------------------------R
Platynothrus_peltifer5              LQILGPYLSSYILVITAIDRYKAICFPLS------------------------------N
Platynothrus_peltifer1              LQILGPYLSSYVLVMTSIDRYQAICYPLS------------------------------N
Platynothrus_peltifer3              LQILGPYLSSYVLVMTAIDRYQAICYPLS------------------------------N
Achipteria_coleoptrata2             LQIFGPYLSSYVLVMTAIDRYQAICNPLS------------------------------N
Platynothrus_peltifer2              MQILGPYLSSYVLVVTAIDRYQAICFPLS------------------------------N
Platynothrus_peltifer4              LQLFGPYLSSYVLVVTAIDRYQAICFPLT------------------------------N
Ixodes_scapularis2                  GQLLGPYLSSYVLVVTAVDRYQAICFPLS------------------------------N
Ixodes_scapularis1                  GQLLGPYLSSYVLVVTAVDRYQAICFPLS------------------------------N
Ixodes_scapularis3                  GQLLGPYLSSYVLVVTAADRYQAICFPLS------------------------------N
Cotesia_vestalis                    LQPFGNYLSSYVLTATAIDRYQAICNPFS------------------------------Y
Microplitis_demolitor               LQPFGNYLSSYVLTATAIDRYQAICYPFN------------------------------Y
Zorotypus_caudelli                  ------------------------------------------------------------
Nasonia_giraulti                    CQPLGSYLSSYVLIATAVDRYHAICYPLS------------------------------Y
Nasonia_vitripennis                 CQPLGSYLSSYVLIATAVDRYHAICYPLS------------------------------Y
Varroa_destructor2                  AQVLVLYLSTYILTGMSLDRLVSM------------------------------------
Leuctra_sp                          GQTLGPHLTAFILVATLWDRTHAFCY----------------------------------
Tricholepidion_gertschi             ------------------------------------------------------------
Perla_marginata                     GQALG-------------------------------------------------------
Crioscolia_alcione                  GQPLGHYLSSYILTAAAIDRYQAICHPLS------------------------------Y
Mischocyttarus-_flavitarsis         FQPLGLYLSSYILTATAIDRYHAICHPLS------------------------------Y
Polistes_metricus                   FQPFGLYLSSYILTATAIDRYHAICHPLS------------------------------Y
Polistes_canadensis                 FQPFGLYLSSYILTATAIDRYHAICHPLS------------------------------Y
Chrysis_viridula                    MQPLGPYLSSYVLMATAIDRYHAICHPLS------------------------------Y
Argochrysis_armilla                 IQPLGPYLSSYV-MATAIDRYHAICHPLS------------------------------Y
Chyphotes_mellipes                  GQPLGPYLSSYVLMATAIDRYHAICHPLS------------------------------Y
Brachycistis_timberlakei            GQPLGPYLSSYVLMATAIDRYHAICHPLS------------------------------Y
```

```
Sphaeropthalma_orestes             IQPIGPYLSSYVLTATAIDRYHAICHPLS------------------------------Y
Homalodisca_vitripennis            GQTMGPYLSSYILVATALDRYKAICEPLN------------------------------Y
Cercopis_vulnerata                 GQTLGPYLSSYVLMSTAMDRYKAICHPLT------------------------------Y
Nilaparvata_lugens                 GQTLGPYLSSYVLMATAMDRYKAICHPLT------------------------------Y
Achipteria_coleoptrata1            LQILGPYLSSYVLVMTSIDRYQAICYPLT------------------------------N
Ephemera_danica                    SQLLGPYLSSFVLVLTAVDRYEAICRPLT------------------------------Y
Leptopilina_clavipes               GQPIGPYLSSYILTATAIDRYHAICFPFS------------------------------Y
Ganaspis_sp                        VQPIGPYLSSYILTATAIDRYHAICFPFS------------------------------Y
Leptopilina_boulardi               GQPIGPYLSSYILTATAIDRYHAICFPFS------------------------------Y
Mantis_religiosa                   GQTL--------------------------------------------------------
Blattella_germanica                GQTLGPYLSAYILMVTALDRYQAVCHPLA------------------------------Y
Zootermopsis_nevadensis            GQTLGPYLSAYILMATALDRYQAVCHPLA------------------------------Y
Stigmatomma_oregonense             GQPFGLYLSSYILTVTAMDRYYAICHPFS------------------------------Y
Dinoponera_quadriceps              GQPFGIYLSSYILTVAAVDRYYAICHPFS------------------------------Y
Harpegnathos_saltator              GQPFGLYLSSYILTVTAMDRYYAICHPFS------------------------------Y
Cerapachys_biroi                   GQPFGLYLSSYILTVTAMDRYYAICHPFS------------------------------Y
Monomorium_pharaonis               GQPFGLYLSSYILTVTAFDRYYAICHPFS------------------------------Y
Tetramorium_bicarinatum            GQPFGLYLSSYILTVTALDRYYAICHPFS------------------------------Y
Solenopsis_invicta                 GQPFGLYLSSYILTVTAIDRYHAICHPFS------------------------------Y
Atta_cephalotes                    GQPFGLYLSSYILTVTAIDRYHAICHPFS------------------------------Y
Acromyrmex_echinatior              GQPFGLYLSSYILTVTAMDRYHAICHPFS------------------------------Y
Pogonomyrmex_barbatus              GQPFGLYLSSYILTVTAIDRHYAICYPFS------------------------------Y
Wasmannia_auropunctata             GQPFGLYLSSYILTVTAIDRYQAICHPFS------------------------------Y
Vollenhovia_emeryi                 GQPFGLYLSSYILTVTAMDRYYAICHPFS------------------------------Y
Orussus_abietinus                  GQPFGLYLSSYVLTATAIDRYRAICKPLD------------------------------S
Blaberus_atropos                   ------------------------------------------------------------
Limulus_polyphemus2                MQILGPYLSSYVLLVTAIDRYQAICFPLT------------------------------S
Isonychia_bicolor                  LQLLGPYLSSFILVLTAVDRFHAICLPLS------------------------------Y
Occasjapyx_japonicus               MQILGPYLSSYVLVMTAIDRYQAICFPLS------------------------------N
Baetis_sp                          GQILGPYLSSIILVLTAIDRYYAICQPLA------------------------------H
Panonychus_ulmi                    LQLLGPYLSSFTLCATAIDRYRAICYPLE------------------------------S
Frankliniella_occidentalis         GQPLGVYLSSYVLTATACDRYRAICHPLT------------------------------Y
Eurylophella_sp                    GQLLGPYLSSYVLVLTAVDRYFAICRPLS------------------------------Y
Telenomus_podisi                   GQPFGPYLSSYVLVVTAIDRYHAICHPLN------------------------------Y
Aposthonia_japonica                CQTLGPYLSSYVLLATAVDRYRAICSPLV------------------------------Y
Haploembia_palaui                  CQTLGPYLSSYVLLATAVDRYRAICSPLV------------------------------Y
Halyomorpha_halys                  GQTLGPYLSSYVLTVTALDRYQAICHPLT------------------------------Y
Stenobothrus_lineatus              GQPLGPYLSSSVLVVTALDRHQAVCRPLT------------------------------Y
Inocellia_crassicornis             NQLLGPYLSAYILSATAIDRHQAICYPLT------------------------------Y
Tenthredo_koehleri                 GQTLGPYLSSYVLAATALDRYQAVRHPLI------------------------------Y
Corydalus_cornutus                 AQPVGPYLSSYVLTATAIDRHQAICHPLT------------------------------Y
Chrysopa_pallens                   GQPLGTYLSSYILSATAIDRHQAICYPLT------------------------------Y
Meloe_violaceus                    GQTLGPYLSSYVLMATAIDRHQAICYPLT------------------------------Y
Aethina_tumida                     GQTLGPYLSSYVLMATAIDRHQAICYPLT------------------------------Y
Gyrinus_marinus                    GQTLGPYLSSYVLMATAIDRHQAICYPLT------------------------------Y
Tribolium_castaneum                GQTLGPYLSSYVLMATAIDRHQAICYPLT------------------------------Y
Nicrophorus_vespilloides           GQTLGPYLSSYILMATAIDRHQAICYPLT------------------------------Y
Aleochara_curtula                  GQTLGPYLSSYILMATAIDRHQAICYP---------------------------------
Pogonus_chalceus                   CQTLGPYLSSYILMATAIDRHQAICYPLT------------------------------Y
Sipyloidea_sipylus                 IQPLGPYLSSYVLLATALDRYQAICHPMT------------------------------Y
Extatosoma_tiaratum                LQPLGPYLSSYVLLATALDRYQAICHPMT------------------------------Y
Aretaon_asperrimus                 FQPLGPYLSSYVLLATALDRYQAICQPMT------------------------------Y
Medauroidea_extradentata           FQPLGPYLSSYVLLATALDRYQAICQPMT------------------------------Y
Ramulus_artemis                    FQPLGPYLSSYVLIATALDRYQAICHPMT------------------------------Y
Galloisiana_yuasai                 GQTLGPYLSSYVLLATALDRFQAICHPLT------------------------------Y
Athalia_rosae                      GQPLGPYLSSYVLMVTALDRYQAICHPLN------------------------------Y


                                   241                                                      300
Varroa_destructor1                 -----RPIVVVRTILSVSWLVAAVLASPQL---YIFSFQKL-----PNG----AH-DCWA
Metaseiulus_occidentalis1          -----STSSRLWVILGAGWFLSALLASPQV---YIFSFKQL-----SNG----VY-DCWG
Metaseiulus_occidentalis2          CRV--GYRKFAKKLIVFAWFLAAALSIPQL---FLFSYTRI-----HDH--GSWY-DCKA
Tigriopus_californicus             -----VEPKPLRPLIQGVWLVAILCALPQP---FIFSIKDI-----DDK---GIY-DCWA
Anurida_maritima                   NHY--GGLKSPKVMVAISWILSLIFASPQA---VIFSQSEI-----RPG----VF-DCWT
Meinertellus_cundinamarcensis      SPQ--AALKEARLMVCAAWILSIILAIPQA---LVFTHREI-----APG----IQ-DCWA
Pogonognathellus_sp                NHY--GGLRGPKILVILSWILSFIFASPQA---FIFSFKEL-----TMNDQTKVY-DCWS
Triops_cancriformis                LHW--NSLKVAKMMVTSAWVLSLLLAIPQA---IIFSEQYI-----NPDGTTTET-DCWV
Strigamia_maritima2                GHW--NSLRAAKLMIVGAWVLAFLAAIPQA---IIFGKMEI-----RPG----VH-DCWA
Catajapyx_aquilonaris              SPW--NSLLAAKIMVASAWVMAFGFATPQA---VIFTLKKI-----SPG----VQ-DCWG
Daphnia_pulex                      -HW--NSLKVAKCFVAASWVMAILFSIPQA---VIFHEEEI-----SVG----VT-DCWV
Daphnia_magna3                     -TW--NSRKVAKCFVAASWVMSMLFAIPQA---VIFHEEEI-----SVG----VT-DCWV
Daphnia_magna2                     -HW--NSLKVAKCFVAASWVMSMLFAIPQA---VIFHEEEI-----SVG----VT-DCWV
Daphnia_magna1                     -HW--NSLKVAKCFVAASWVMSMLFAIPQA---VIFHEEEI-----SVG----VT-DCWV
Daphnia_magna4                     -HW--NSLKVAKCFVAASWVMSMLFAIPQA---VIFHEEEI-----SVG----VT-DCWV
Arachnocampa_luminosa              CSW--NYR-HALVPVFFSWILAAVVSSPQL---FLFKIQHM-----SVQHY--TVETCSV
Calanus_finmarchicus               CSW--EPK-NSDKLVGGAWVLAILCCIPQL---LIFNTSSQ-----IGCNSGEMFEKCQT
Epiophlebia_superstes              RVF--LPR-SPRRLVAIAWALSFLFSLPQA---FVFSYRRA-----TE----TEW-DCWA
```

```
Ladona_fulva                    RAF--LPR-SPRRLVAIAWALSALFSLPQA---FVFSYQKA-----TE----TEW--CWA
Cordulegaster_boltonii          RAF--LPR-SPRRLVAIAWALSFLFSLPQA---FIFSYRKA-----TE----TEW-DCWA
Diaphorina_citri                HTW--NSR-RSRIMIMTAWSTAFTFCIPQI---TIFSYQEV-----KP----GIY-DCWA
Pachypsylla_venusta             HSW--NSR-RSRIMILIAWSAAFTFCIPQV-QISIFSYQEI-----KP----GVY-DCWA
Acanthocasuarina_muellerianae   ------------------------------------------------------------
Glomeris_pustulata              ------------------------------------------------------------
Frankliniella_cephalica         -------------------------------------SIEP-----VE----GGY-DCWA
Machilis_hrabei                 ------------------------------------------------------------
Carabus_granulatus              ------------------------------------------------------------
Dendroctonus_frontalis          CSW--TSR-RSKFMMYVAWIASLLCCIPQV---IIFSFQEV-----EP----RVY-DCWA
Lepeophtheirus_salmonis         CTW--TPK-RSNIMIGLAWLVSLALCVPQI---IIFGSGE-----------KSH-SCSA
Caligus_rogercresseyi           CTW--TPK-RSNIMIGLAWLVSFALCIPQA---IIFGSSE-----------AKY-SCSA
Parhyale_hawaiensis             CRW--RPG-HSIRLIGAATFLSLWLSVPQL---FIFSLRSK-----NG-----IQ-DCWA
Hyalella_azteca                 CRW--TPG-HSIRLISCATAISLLFSIPQL---FNFSFREK-----NG-----IY-DCWA
Strigamia_maritima1             -SW--AAT-RSKGMVCVAWVVSVVFSSPQM---FLFSYKYV-----SV--L-KYW-ECWV
Conwentzia_psociformis          ------------------------------------------------------------
Liposcelis_bostrychophila       ------------------------------------------------------------
Tetranychus_urticae             RS---ALN-RSKLKVSIAWGLALICCLPQL---FIFSYRQI-----PN--SPGVY-ECWG
Osmylus_fulvicephalus           ------------------------------------------------------------
Ornithodoros_turicata           CSW--TPT-KSKLMIAFAWAVSLLCCVPQL---FIFSYQQV-----EN----GVY-DCWG
Acerentomon_sp                  SRW--TER-RYKAMIAVAWTATLCLCSPQV---FIFSYMKV-----GTNQSGDIY-DCWA
Ixodes_ricinus                  CSW--TPT-KSKLLISAAWVASLLCCVPQV---FIFSYQEI-----SP----RVF-DCWG
Folsomia_candida                ------------------------------------------------------------
Limulus_polyphemus1             CVW--TPR-KSKLMIIAAWTISMLCCVPQV---FIFSYQKV-----LI-EP-ITF-DCWG
Limulus_polyphemus3             CTW--TPR-RSKLMIVGAWIISILCCSPQA---FIFSYQQV-----SS-DP-LVF-DCWG
Platynothrus_peltifer5          CSW--TPK-RSQLMISIAWVLSLIFCAPQL---FIFSYQQI-----PG-TLGNVY-DCWG
Platynothrus_peltifer1          CAW--TPK-RSQLMISFAWIIALLCCIPQV---IIFSYQQI-----PG-YD--II-DCWG
Platynothrus_peltifer3          CAW--TPS-RSKLMILMAWIVSLLCCAPQI---IIFSYQEI-----G---S-QIF-DCWG
Achipteria_coleoptrata2         CKW--TPG-RSKLMITIAWIVTSFCCSPQI---FIFSYQQI-----PG-TD-AIY-DCWG
Platynothrus_peltifer2          CSW--TPR-RAKLMISCAWIISIVCSLPQA---FIFSYQEV-----PE-TN-HIY-DCWG
Platynothrus_peltifer4          CHW--TSR-KSKLMITIAWIISILCSAPQA---FIFSYQEI-----PR-TN-GVR-DCWG
Ixodes_scapularis2              CSW--SPT-KCKLLIAGAWVAALVCCVPQV---FIFSYQEV-----APGV----F-DCWG
Ixodes_scapularis1              CSW--TPT-KSKLLICAAWVAALLCCVPQV---FIFSYQEV-----SPEV----F-DCWG
Ixodes_scapularis3              CSW--TPT-KSKLLISAAWVASLLCCVPQV---FIFSYQEI-----SPKV----F-DCWG
Cotesia_vestalis                CR----------------------------------------------------------
Microplitis_demolitor           CRT--SSL-KSRVMVYGAWSLSFVLCIPQV---IVFSYQEI-----SPGV----W-DCWA
Zorotypus_caudelli              ------------------------------------------------------------
Nasonia_giraulti                CRT--TSR-RSRITVYVAWLLALLFCLPQV---FIFSYQEI-----SAGV----W-DCWA
Nasonia_vitripennis             CRT--TSR-RSRITVYVAWLLALLFCLPQV---FIFSYQEI-----SAGV----W-DCWA
Varroa_destructor2              ------------------------------------------------------------
Leuctra_sp                      ------------------------------------------------------------
Tricholepidion_gertschi         ------------------------------------------------------------
Perla_marginata                 ------------------------------------------------------------
Crioscolia_alcione              CSS--TSR-KARIMLYSAWVVALVFCLPQI---FIFSYREI-----SPNV----W-DCWA
Mischocyttarus-_flavitarsis     CSV--TSR-KSRIMVYCAWSLSLILCIPQL---FVFSYQEI-----TPNI----W-DCWA
Polistes_metricus               CSV--TSR-KSRIMVYCAWSFALILCIPQL---FVFSYQEV-----MPRI----W-DCWA
Polistes_canadensis             CSV--TSR-QSRIMVYCAWSFALILCIPQL---FVFSYQEV-----MPRI----W-DCWA
Chrysis_viridula                CGS--SLK-RSKIMVYSAWAVTLILCIPQV---IVFSYQEV-----TPKV----W-DCWA
Argochrysis_armilla             CGS--SLK-RSKIMVYSAWAVALILCIPQV---VVFSYQEV-----TPKV----W-DCWA
Chyphotes_mellipes              CRT--TSK-RSRVMVYGAWIITLFLCLPQV---FIFSYQEI-----MPSV----W-DCWA
Brachycistis_timberlakei        CST--TSR-RSRIMVYGAWVLALLLCLPQV---IVFSYKEV-----EQQV----W-DCWA
Sphaeropthalma_orestes          CST--TSR-RSRIMVYAAWIFSLILCLPQV---YVFSYEEI-----MPNV----W-DCWA
Homalodisca_vitripennis         SNW--TSR-RSALTVSAAWSLTLLFCIPQVSQVIIFSYQEI-----APGV----Y-DCWA
Cercopis_vulnerata              CSW--TSR-RSRTMVRVAWIVSILCIPQV---NIFSYQEI-----APGI----Y-DCWA
Nilaparvata_lugens              CSW--TSH-HARSMVQCAWATALLFCIPQV---TIFSYMEVPG---SPGD----Y-DCWA
Achipteria_coleoptrata1         CQW--TPT-RSQLMITLAWIIALLCCIPQV---LIFSYQQIP----GT----DIY-DCWG
Ephemera_danica                 CTW--PAR-RAWFLVAGAWTLALLCCAPQL---FVFSYRPVQQ---QPDV----F-DCWA
Leptopilina_clavipes            CRT--TSR-RSRIMVYSAWIIALILCTPQV---FVFSYQLI-----SPGI----W-DCWA
Ganaspis_sp                     CRT--TSR-RSRIMVYCAWIIALILCTPQV---FVFSYQQI-----SAGI----W-DCWA
Leptopilina_boulardi            CRT--TSR-RSRIMVYSAWIIALILCTPQI---FIFSYQLI-----SPGV----W-DCWA
Mantis_religiosa                ------------------------------------------------------------
Blattella_germanica             CSW--TSR-RSKFMVWTAWVFALGFCVPQ------------------------------
Zootermopsis_nevadensis         CSW--TSR-RSRAMVWMAWVFALIFCIPQV---S--------------------------
Stigmatomma_oregonense          CRV--TSR-RSRMMVYGAWTLATILCIPQV---FIFSYQEI-----STGV----W-ECWA
Dinoponera_quadriceps           CSV--TSR-RSKMMVYVVWLLAVVLCVPQV---FIFSYQEI-----SPNV----W-DCWA
Harpegnathos_saltator           CNV--TSR-RSKMMVYAAWTLAVVLCVPQI---FIFSYQEI-----SPNV----W-ECWA
Cerapachys_biroi                CAV--TSR-RSKMMVYGAWVLAAILCVPQV---FVFSYKEI-----SPGV----W-ECWA
Monomorium_pharaonis            CGV--TSQ-RSKMMVYGAWALAVILCVPQI---FIFSYMEI-----APDI----W-ECWA
Tetramorium_bicarinatum         CSV--TSR-RSKMMVYGAWTLSVALSVPQI---FIFSYTEI-----APGV----W-ECWA
Solenopsis_invicta              CGV--TSR-RSKMMVYGAWVLATMLCVPQI---FIFSYMEI-----SPGV----W-ECWA
Atta_cephalotes                 CSV--TSR-RSKMMVYGTWMLAAMLCVPQI---FIFSYMEI-----SPGV----W-ECWA
Acromyrmex_echinatior           CSV--TSR-RSKMMVYGTWTLAAMLCVPQI---FIFSYMEI-----SPGV----W-ECWA
Pogonomyrmex_barbatus           CGI--TSR-RSKMMVYGAWMLAVILCIPQI---FIFSYTEI-----SPGV----W-ECWA
Wasmannia_auropunctata          CGV--TSR-RSKMMVYGAWTLAAALCVPQI---VIFSYTEI-----SPGV----W-ECWA
Vollenhovia_emeryi              CGI--TSR-RSKMMVYGAWVLAATLCVPQI---FIFSYTEI-----SPGV----W-ECWA
Orussus_abietinus               LDHRICLL-QSRNMIRCSWCLALLFSLPQV---FVFSYKEI-----SPGV----W-DCWA
Blaberus_atropos                ------------------------------------------------------------
```

```
Limulus_polyphemus2              CVW--TPR-KSKIMIANAWIISILCCFPQI---FIFSYQKVSE---DPVT----F-DCWG
Isonychia_bicolor                CTW--PAK-KAKLLVSGAWVLALLCCLPQV---LIFSYQPI-----TPGG--DVF-DCWA
Occasjapyx_japonicus             CSW--TPK-RSKVMIGISWVISLMFCAP--------------------------------
Baetis_sp                        CTW--PVR-RARALVCVAWALALACCTPQV---FIFSYQPIG----------DTY-DCWG
Panonychus_ulmi                  RSR---LN-RSNLKVSIAWGLALICCLPQL---LIFSYHQIPN---NPQV----Y-ECWG
Frankliniella_occidentalis       CSW--TSR-RAKTMVWAAWGVAICFSAPQV---RSEGYTAGQRQTLEANSV-PCV-NCCN
Eurylophella_sp                  CTW--PAR-RARLLVSFTWVLALLFCLPQL---AVFSYQRVQNAPDKQDQ----Y-DCWA
Telenomus_podisi                 CRT--TSR-RSRFMVYAAWILAFLLCTPQA---YVFSFQEV-----WPGV----S-DCWA
Aposthonia_japonica              HSW--SFR-RAKIMVLIAWIISLWFCVPQA---VVFSYQEL-----EPGV----W-DCWA
Haploembia_palaui                HSW--SFG-RAKVMVFIAWTISLLFCVPQS---IIFSYQEL-----KPGV----W-DCWA
Halyomorpha_halys                CSW--SSK-RARGMIMGAWALSITFCTPQL---TIFSYQEV-----APGV----F-DCWA
Stenobothrus_lineatus            CSW--TYR-RSRAMMQVAW-----------------------------------------
Inocellia_crassicornis           CSW--TSR-CSRAMIWMAWAASLAFCIPQL---IIFSYQEI-----EPNV----W-DCWG
Tenthredo_koehleri               CSI--TSR-RSKMTVYFAWCLSLLLCVPQT---LVFSYKEV-----SSGV----W-DCWA
Corydalus_cornutus               CSW--TSR-RSKGMVYLAWLCSLIFCIPQV---FIFSYQIVP---DAPGV----Y-DCWA
Chrysopa_pallens                 CSW--TSQ-RSQVMVWTAWIISLIFCIPQL---FIFSYQEV-----SPGI----W-DCWG
Meloe_violaceus                  CSW--TSR-RSKVMVYIAWCTSLIFCLPQL---FIFRYKEL-----SDGI----Y-DCWA
Aethina_tumida                   CSW--TSR-RSKFMVYIAWITSLVFCIPQL---TIFSYQEV-----DDGE----F-DCWA
Gyrinus_marinus                  CSW--TSR-RSKVMVYTAWVAALAFCIPQL---NIFSYQEI-----APGV----Y-DCWA
Tribolium_castaneum              CSW--TSR-RSKVMVYLAWVASLAFCIPQL---TIFTYTSV-----GEDE----Y-DCWA
Nicrophorus_vespilloides         CSW--TSR-RSRVMVWIAWVLSLAFCIPQI---TIFSYQHV-----ENEE----Y-DCWA
Aleochara_curtula                ------------------------------------------------------------
Pogonus_chalceus                 CSW--TSR-RSKVMVWLAWATSLAFCIPQV---TIFAYQEV-----APGI----N-DCWA
Sipyloidea_sipylus               CSS--DSR-RSRVMIWMAWCVSLAFCIPQV---IIFSFQET-----VPGV----Y-DCWG
Extatosoma_tiaratum              CST--DSR-RSRVMVWMAWCVSLAFCIPQV---IIFSYQET-----APGV----Y-DCWA
Aretaon_asperrimus               CSS--DSR-RSRVMVWMAWCISLVFCIPQV---IIFSYQET-----EPGV----H-DCWA
Medauroidea_extradentata         CST--DSR-RSRAMIWLAWCVSLVFCIPQV---IIFSYQET-----APGV----F-DCWA
Ramulus_artemis                  CST--DSR-RSRVMIWLAWCVSLGFCIPQV---IIFSYQET-----ASGV----Y-DCWA
Galloisiana_yuasai               CSW--TSR-RSRTMVWLAWAFS--------------------------------------
Athalia_rosae                    CSV--TSR-RSKIMVYLAWGLSLALCIPQL---VVFSYQEA-----SPGV----W-DCWG

                                 301                                                      360
Varroa_destructor1               TFEP-PITSFRYVLFFITAV----------------------------------------
Metaseiulus_occidentalis1        TFDP-PLTSVRYVVYFIAMA----------------------------------------
Metaseiulus_occidentalis2        NFSWHRFGQRIYTTYFVVLI----------------------------------------
Tigriopus_californicus           DFGGEKWLEKGYVVTFLFTV----------------------------------------
Anurida_maritima                 FL--GPSGVKAYVTWFVISV----------------------------------------
Meinertellus_cundinamarcensis    DFPGGQQGAKIYVLWFVIAV----------------------------------------
Pogonognathellus_sp              TFIE-PWGVRAYVTWFVVCA----------------------------------------
Triops_cancriformis              RFIE-PWGAKAYVTWFVISI----------------------------------------
Strigamia_maritima2              HFEQ-PWGEKAYVTWFVLSI----------------------------------------
Catajapyx_aquilonaris            TFVE-PWGTKAYVTWFVISI----------------------------------------
Daphnia_pulex                    QFVE-PWGAKAYVTWFV------------------------------------------V
Daphnia_magna3                   QFAE-PWGAKAYVTWFV------------------------------------------V
Daphnia_magna2                   QFAE-PWGAKAYVTWFVVSIFGAPLLVVAVCYXXXXXXXXXWVQFAEPWGAKAYVTWFVV
Daphnia_magna1                   QFAE-PWGAKAYVTWFV------------------------------------------V
Daphnia_magna4                   QFAE-PWGAKAYVTWFV------------------------------------------V
Arachnocampa_luminosa            KWLS-KKHEGLYLLFHMSTQ----------------------------------------
Calanus_finmarchicus             NFPT-WVTPSQYILYFSFAN----------------------------------------
Epiophlebia_superstes            TFEP-PWTQKAYVTWYGISV----------------------------------------
Ladona_fulva                     TFDP-PWTQKAYVTWYGISV----------------------------------------
Cordulegaster_boltonii           TFDP-PWTQKAYVTWYGISV----------------------------------------
Diaphorina_citri                 SWDQ-EWGERAYVTWFSITV----------------------------------------
Pachypsylla_venusta              SWDQ-EWGERIYITWFSVSV----------------------------------------
Acanthocasuarina_muellerianae    ------------------------------------------------------------
Glomeris_pustulata               ------------------------------------------------------------
Frankliniella_cephalica          TFVP-EWGQRAYVTWYTCSV----------------------------------------
Machilis_hrabei                  ------------------------------------------------------------
Carabus_granulatus               ------------------------------------------------------------
Dendroctonus_frontalis           TFDN-EWGEQAYVVCYSVTV----------------------------------------
Lepeophtheirus_salmonis          TFAQ-DWGQKAYVTWFAVSN----------------------------------------
Caligus_rogercresseyi            SFVV-DWGQKAYVTWFAVSN----------------------------------------
Parhyale_hawaiensis              SFIQ-PWGVVAYVTFYSIAV----------------------------------------
Hyalella_azteca                  DFIP-GWGVTAYVTWYAIAV----------------------------------------
Strigamia_maritima1              DFTL--EEERAYVTWYFVAN----------------------------------------
Conwentzia_psociformis           ------------------------------------------------------------
Liposcelis_bostrychophila        ------------------------------------------------------------
Tetranychus_urticae              TFIQ-PYGERLYVTWYALTS----------------------------------------
Osmylus_fulvicephalus            ------------------------------------------------------------
Ornithodoros_turicata            TYAE-PWGVTAYVTWYGVSV----------------------------------------
Acerentomon_sp                   TFIE-PWGAKTYVTWYAFTI----------------------------------------
Ixodes_ricinus                   TYVE-PWGLRAYVTWYGVSV----------------------------------------
Folsomia_candida                 ------------------------------------------------------------
Limulus_polyphemus1              VFIQ-PWGEKVYVLWYALSQ----------------------------------------
Limulus_polyphemus3              TFIQ-PWGEKVYVLWYTISQ----------------------------------------
Platynothrus_peltifer5           IFPQ-PYGERIYVTWYAITV----------------------------------------
```

Platynothrus_peltifer1	TFPQ-PFGERIYVSWYAISF--
Platynothrus_peltifer3	TFSQ-PYGERIYVTWYAVSV--
Achipteria_coleoptrata2	TFQK-PWGERIYVTWYAVSV--
Platynothrus_peltifer2	TFPQ-PWGERIYVTWYAISV--
Platynothrus_peltifer4	TFPQ-PYGERIYVTWYAITV--
Ixodes_scapularis2	TFA-QPWGLRAYVTWYSVSV--
Ixodes_scapularis1	TYV-ESWGLRAYVTWYGMSV--
Ixodes_scapularis3	TYV-EPWGLRAYVTWYGVSV--
Cotesia_vestalis	--
Microplitis_demolitor	SFS-LPYGERIYVTWYSITI--
Zorotypus_caudelli	--
Nasonia_giraulti	TFT-VPYGERAYVTWYSVTV--
Nasonia_vitripennis	TFT-VPYGERAYVTWYSVTV--
Varroa_destructor2	--
Leuctra_sp	--
Tricholepidion_gertschi	--
Perla_marginata	--
Crioscolia_alcione	TFD-LPYGERAYVTWYAVTV--
Mischocyttarus-_flavitarsis	TFD-IKFGERAYVTWYSLMV--
Polistes_metricus	TFD-IKFGEKAYVTWYSLMV--
Polistes_canadensis	TFD-IQFGEKAYVTWYSLMV--
Chrysis_viridula	VFN-VAYGERIYVTWYSTMV--
Argochrysis_armilla	VFD-VAYGERIYVTWYSTMV--
Chyphotes_mellipes	TFD-PTYGEKAYVTWYSITV--
Brachycistis_timberlakei	TFD-LEYGERAYVTWYSVTV--
Sphaeropthalma_orestes	TFG-LHYGERAYVTWYSVTV--
Homalodisca_vitripennis	TFP-FPWGERVYVTWYSVSV--
Cercopis_vulnerata	TFP-EPWGEKLYVTWYSVSV--
Nilaparvata_lugens	TFP-RDWGERAYVTWYSVSV--
Achipteria_coleoptrata1	TFPLQPYGERIYVTWYAISF--
Ephemera_danica	TFE-PGWGERAYVSWYATSV--
Leptopilina_clavipes	TFT-VPYGQEAYVTWYSISV--
Ganaspis_sp	TFK-MPYGQEAYVTWYSISV--
Leptopilina_boulardi	TFT-VPYGQEAYVTWYSISV--
Mantis_religiosa	--
Blattella_germanica	--
Zootermopsis_nevadensis	--
Stigmatomma_oregonense	IFY-LKYGERAYITWYSVTQ--
Dinoponera_quadriceps	TFN-VKYGERSYVTWYSVTQ--
Harpegnathos_saltator	TFH-LKYGERAYITWYSVTQ--
Cerapachys_biroi	TFY-LKYGERAYITWYSVTQ--
Monomorium_pharaonis	TFY-MPYGERAYITWYSVTQ--
Tetramorium_bicarinatum	TFY-LKYGERAYITWYSVMQ--
Solenopsis_invicta	TFY-LKYGERAYITWYSVTQ--
Atta_cephalotes	IFY-LKYGERAYITWYSVMQ--
Acromyrmex_echinatior	TFY-LKYGERAYITWYSVMQ--
Pogonomyrmex_barbatus	TFY-LKYGERAYITWYSIMQ--
Wasmannia_auropunctata	TFY-LKYGERAYITWYSVTQ--
Vollenhovia_emeryi	TFN-LKYGERAYITWYSVTQ--
Orussus_abietinus	TFE-LSYGERAYVTWYCLAV--
Blaberus_atropos	--
Limulus_polyphemus2	IFI-QPWGEKVYVLWYAVSQ--
Isonychia_bicolor	TFP-PYWGERAYVSWYTVSV--
Occasjapyx_japonicus	--
Baetis_sp	TFP-EGWGERAYVTWYSGSV--
Panonychus_ulmi	TFI-QPYGERLYVTWYAFSS--
Frankliniella_occidentalis	LFI---
Eurylophella_sp	TFQ-PAWGERAYVTWYAGSV--
Telenomus_podisi	TFN-LPYGERIYVTWYSVTV--
Aposthonia_japonica	TFI-KPWGERAYVTWYSVFS--
Haploembia_palaui	TFI-QPWGERTYITWYSVFA--
Halyomorpha_halys	VFS-EPYGERVYVTWYSISV--
Stenobothrus_lineatus	--
Inocellia_crassicornis	TFP-EPWGAQAYVTWYSLSI--
Tenthredo_koehleri	TFDLTPYGQQTYVTWYSISV--
Corydalus_cornutus	TFS-ETWGEKAYVTWY--
Chrysopa_pallens	TFP-EKWGEKTYVTWYSISV--
Meloe_violaceus	VFE-EPWGAKAYVTWYSISV--
Aethina_tumida	TFQ-EPWGQKAYVIWYCVSV--
Gyrinus_marinus	TFP-QAWGQKAYVTWYGISV--
Tribolium_castaneum	TFQ-EPWGKRAYVTWYSISV--
Nicrophorus_vespilloides	TFL-PAWGEKAYVTWYGISV--
Aleochara_curtula	--
Pogonus_chalceus	TFP-QPWGARAYVTWYSISV--
Sipyloidea_sipylus	TFA-SDWGETTYVTWYSVSV--
Extatosoma_tiaratum	TFA-GHWGERTYVTWYSVSV--
Aretaon_asperrimus	TFV-EKWGERTYVTWYSISV--

```
Medauroidea_extradentata         TFP-EAWGEKTYVTWYSVSV----------------------------------------
Ramulus_artemis                  TFS-GAWGETTYVTWYSVSV----------------------------------------
Galloisiana_yuasai               ------------------------------------------------------------
Athalia_rosae                    TFY-SPLGERAYVTWFSISA----------------------------------------

                                 361                                                      420
Varroa_destructor1               --LFIPVTLMALCYTYLSWII---------------------------------------
Metaseiulus_occidentalis1        --LIVPALIMGACYSYLCRAS---------------------------------------
Metaseiulus_occidentalis2        --LGVPVVMMLFCYIQICLIIVRIQRHH---MTKTTSAPAN-------------------
Tigriopus_californicus           --FFLPFIVITSSYCIVTYRIWSYSRAT---QGSKASPQMSLAS--------NR-VVLSS
Anurida_maritima                 --FLLPLLICGISYGAICFKVVSFSLHE---SNNRKLNEV-SR-----------------
Meinertellus_cundinamarcensis    --FMAPLTVIAACYGIISLKVWQFSSPQ---STTAEPLAA--------------------
Pogonognathellus_sp              --FILPLVIIGASYGAICCKVISFNLPN---ES---------------------------
Triops_cancriformis              --FALPLLIIGISYGAISLRLWRYSQEA---HRSEEMTRGHKRT--------IGLP-AGA
Strigamia_maritima2              --FIIPLIVIAASYGFICYTLWIYDEEH---GRSGDVI----AL--------R-------
Catajapyx_aquilonaris            --FVMPLLVISSSYGAISYRIWTYNSEP---RCWT-------------------------
Daphnia_pulex                    SIFGAPLLVIGVCYGVICRQIWIYSQSA---LPSSQQPPTSSAY--------P--ALTSE
Daphnia_magna3                   SIFGAPLLVVAVCYGVICRQIWIYSQSA---QPSLLPS-DNSAI--------PSHSSITE
Daphnia_magna2                   SIFGAPLLVVAVCYGVICRQIWIYSQSA---QPSLLPS-DNSAI--------PSHSSITE
Daphnia_magna1                   SIFGAPLLVVAVCYGVICRQIWIYSQSA---QPSLLPS-DNSAI--------PSHSSITE
Daphnia_magna4                   SIFGAPLLVVAVCYGVICRQIWIYSQSA---QPSLLPS-DNSAI--------PSHSSITE
Arachnocampa_luminosa            --FLIPLFVLTFLYSRIFMTVSKNIKQK---HASIRFERESSKTATEINQLNQENSLNTQ
Calanus_finmarchicus             --FFIPLILLFSNCFICKTIWASSSNLNWNRRTTKKENKM--------------------
Epiophlebia_superstes            --FAVPLVLLLYAYAGICSVLRRNHREK---TRPHDERFLS--------RG---------
Ladona_fulva                     --FAVPLVLLLYAYAGICRALRRNHREK---LRPQEDGLIP--------RG---------
Cordulegaster_boltonii           --FAVPLVLLLYAYAGICRALRRNHREK---MRPQQDGIIP--------RG---------
Diaphorina_citri                 --FIIPLFCLVYTYTCICVALWQNTTYP--------------------------------
Pachypsylla_venusta              --FIIPLICLIYTYTCICLAL-KNTAYP--------------------------------
Acanthocasuarina_muellerianae    ------------------------------------------------------------
Glomeris_pustulata               ------------------------------------------------------------
Frankliniella_cephalica          --FMIPLTVLLFTYSRICCEIWRSASSK---HAPALVDGTC--------KTCPHV-----
Machilis_hrabei                  ------------------------------------------------------------
Carabus_granulatus               ------------------------------------------------------------
Dendroctonus_frontalis           --FIIPLFVLVYTYTCICREIWRCSDSS---LRPRSM-----------------------
Lepeophtheirus_salmonis          --FFLPLIILIYCYGCICKCIWDNFNSK---IPLNPGVEVR-------SITCCKNKGRAE
Caligus_rogercresseyi            --FFFPLLILIYCYGRICKTIWDNFNSK---VPMSSD--AG-------GVTCCGNRGRAE
Parhyale_hawaiensis              --FFVPLILLVFFSVCIIATLLRHNFQV---PIQCNYIRQSYST---TSVHCLHHSPH--
Hyalella_azteca                  --FFIPLVLLIFFSGCICCALLRYNFQV---PNQSSYVSRSHSQ---AGQESPNNETTS-
Strigamia_maritima1              --TFVPLLVLVYTYSAICWAVWRNFRHK---KNSPKGDFDS------AASSSPTT-----
Conwentzia_psociformis           ------------------------------------------------------------
Liposcelis_bostrychophila        ------------------------------------------------------------
Tetranychus_urticae              --FFIPFVILIFTFSNICLELWRNGRRI---KK---------------------------
Osmylus_fulvicephalus            ------------------------------------------------------------
Ornithodoros_turicata            --FFVPLAVLTFTYIYICRAIWHNLYLK---RKSSEGSRDK-------------------
Acerentomon_sp                   --FIIPLLVLIYTYFNICRTIWRSKNWI---STKVSPVKRC------VKVSQLYEDGGA-
Ixodes_ricinus                   --FFVPLAVLSFTYVCICRSIWRNLYLK---RKSSDAESWK-------------------
Folsomia_candida                 ------------------------------------------------------------
Limulus_polyphemus1              --FFIPLIVITFTCVRICKNVWYNFHLR---GK---------------------------
Limulus_polyphemus3              --FFIPLLVITFTYVNVTKTVWNNYYLR---KK---------------------------
Platynothrus_peltifer5           --FVIPCIIILFAHVCICREICNNLHRK---RESIEEIKNC------IHYSFKRN-----
Platynothrus_peltifer1           --FFVPFILLTFTHVCICREIWRNVHQK---RKSVKMEQNK------IIQNDDS------
Platynothrus_peltifer3           --FIIPFIILTFTHVHICREIWLNLHKK---RKSFKINRNK------SSSSCNNR-----
Achipteria_coleoptrata2          --FIIPFIIIVVTHYHICREIWLNLHKK---RKSFKLNKNR-------------------
Platynothrus_peltifer2           --FFIPLVILTFTHIYICREIWVNLNLK---RQTGRRETQT------INQNIDNS-----
Platynothrus_peltifer4           --FFIPLIIITYTYVYICREVWANVRRK---RQTLKPEISL------IEVRKNW------
Ixodes_scapularis2               --FFVPLAVLSFTYVCICRSIWRNLYLK---RKSSDAD----------------------
Ixodes_scapularis1               --FFVPLMVLTFTYVCICRSIWRNLYLK---RKSSDAES---------------------
Ixodes_scapularis3               --FFVPLVVLSFTYVCICRSIWRNLYLK---RKSSDAES---------------------
Cotesia_vestalis                 ------------------------------------------------------------
Microplitis_demolitor            --FLLPFLVLLYTYASICANIRRNTEIS---DRFDHVN----------------------
Zorotypus_caudelli               ------------------------------------------------------------
Nasonia_giraulti                 --FLLPFCVLTFTYAEICCSIWRNREVM---VLASHER----------------------
Nasonia_vitripennis              --FLLPFCVLTFTYAEICCSIWRNREVM---VLASHER----------------------
Varroa_destructor2               ------------------------------------------------------------
Leuctra_sp                       ------------------------------------------------------------
Tricholepidion_gertschi          -----------------------TRGRP---VIIHQGAT---------------------
Perla_marginata                  ------------------------------------------------------------
Crioscolia_alcione               --FLLPFAILLYTYVGICIGIWRSSNIL---GATDNRK----------------------
Mischocyttarus-_flavitarsis      --FLLPFIVLVYTYVGICVGVWQSNKMS---DAVDDRK----------------------
Polistes_metricus                --FLLPFIVLVYTYVGICVGVWQSNKMS---GAVDDRT----------------------
Polistes_canadensis              --FLLPFIVLVYTYVGICVGVWQSNKMS---GAVDDRA----------------------
Chrysis_viridula                 --FLLPFTVLLYTYTGICIGIWRSNKMS---GVEDWRK----------------------
Argochrysis_armilla              --FLLPFTVLLYTYTGICIGIWRSNKMT---SAENWRK----------------------
Chyphotes_mellipes               --FLLPLIVLTYTYTGICIGIYKSNRMS---GVNDVRK----------------------
Brachycistis_timberlakei         --FLLPLIVLIYTYVGICVGIWRSNKMT---GMVDVIG----------------------
Sphaeropthalma_orestes           --FLLPLVVLIYTYTGICIGIWQSNKMT---GIMDVKI----------------------
```

```
Homalodisca_vitripennis              --FLVPLFVLIYTYFCICWTLWRYSGLP---DS-S-------------------------
Cercopis_vulnerata                   --FLIPLCVLIYTYTCICRELWRNAGFP---ESTS-------------------------
Nilaparvata_lugens                   --FILPLIVLIYTYWCICRELWRNAGFP---VTKA-------------------------
Achipteria_coleoptrata1              --FFIPFILLTATHVCICREIW--------------------------------------
Ephemera_danica                      --FLVPLMVLVVAYFRICQALWRNYNLK---QRAQQNGL---------------------
Leptopilina_clavipes                 --FLLPFTVLVYTYTGICIGVWKNSGVS---DPLEIGYK---------------------
Ganaspis_sp                          --FLLPFTVLIYTYTGICIGVWKNSGVS---DPLEIGFA---------------------
Leptopilina_boulardi                 --FLLPFTVLVYTYTGICIGVWKNSGVS---DPLEIGYK---------------------
Mantis_religiosa                     ------------------------------------------------------------
Blattella_germanica                  ------------------------------------------------------------
Zootermopsis_nevadensis              ------------------------------------------------------------
Stigmatomma_oregonense               --FLLPFAVLVYTYVRICISVWTSNKMS---RVIDL------------------------
Dinoponera_quadriceps                --FLLPFIVLVYTYTRICITIWTSNKMS---GVVDL------------------------
Harpegnathos_saltator                --FLLPFIVLAYTYMRICISIWASSKIS---GVVDF------------------------
Cerapachys_biroi                     --FLLPFIVLVYTYTQICRSIWTSGKMS---GVVDF------------------------
Monomorium_pharaonis                 --FLIPFIVLIYTYTKICVSIWTSNKIS---GIVDF------------------------
Tetramorium_bicarinatum              --FLLPFLVLTYTYTKICMAIWTSSKMS---GVVDL------------------------
Solenopsis_invicta                   --FLIPFIALTYTYTKICVSIWTSSKMS---GVIDL------------------------
Atta_cephalotes                      --FLLPFIVLVYTYTRICISIWRSSKLS---GLIDL------------------------
Acromyrmex_echinatior                --FLLPFIVLVYTYTRICISIWRSSKLS---GLIDL------------------------
Pogonomyrmex_barbatus                --FLLPFIVLVYTYTRICMSIWTSSKMS---GVVDL------------------------
Wasmannia_auropunctata               --FLLPFMVLVYTYTRICLSIWRSSKMS---GVVEL------------------------
Vollenhovia_emeryi                   --FLLPFIVLVYTYTRICMSIWTSSKMS---GVVDL------------------------
Orussus_abietinus                    --FLLPLCVLVYTYSGICRVVRRRSEVG---VATSKS-----------------------
Blaberus_atropos                     ------------------------------------------------------------
Limulus_polyphemus2                  --FFIPLVVIAFTYIRICKSVWYNLQMR---RQASNC-----------------------
Isonychia_bicolor                    --FLAPLLILFFTYTCICRAIWHNYNAK---KIASDGFT---------------------
Occasjapyx_japonicus                 ------------------------------------------------------------
Baetis_sp                            --FLIPLLILIFTYTCICQAIWRNYNAK---KESVAGLP---------------------
Panonychus_ulmi                      --FFIPFTIIIFTFSNICSELWKN------------------------------------
Frankliniella_occidentalis           ------------------------------------------------------------
Eurylophella_sp                      --FLVPLAILVYAYLCICFAIWRNYNNK---KQFSAQGI---------------------
Telenomus_podisi                     --FVLPYCVLIYTYTGICIGIWNNRGLS---GLPNSS-----------------------
Aposthonia_japonica                  --FIIPLSVLTYTYSCICRKIWRNTGAG---FHII-------------------------
Haploembia_palaui                    --FIIPLSVLTYTYSCICRKIWRNTGAG---FHII-------------------------
Halyomorpha_halys                    --FVVPLVSLIYTYTCICLTIWHNTGPL---SASA-------------------------
Stenobothrus_lineatus                ------------------------------------------------------------
Inocellia_crassicornis               --FIIPLCVLVFTYTCICREIWRTARHG---ILRPATTP---------------------
Tenthredo_koehleri                   --FILPLIILVYTYTSICIVIWNNNGIV---GEAR---S---------------------
Corydalus_cornutus                   ------------------------------------------------------------
Chrysopa_pallens                     --FIIPLSILVFTYTRICCEIWRNANIG---MPLR---A---------------------
Meloe_violaceus                      --FIVPLIVLTVTYTSICIEIWHSSESS---L--R---P---------------------
Aethina_tumida                       --FIVPLIVLVYTYTCICKDIWHSSEIS---M--R---P---------------------
Gyrinus_marinus                      --FIVPLTVLTTTYGCICREIWQSAAAG---DLGG---P---------------------
Tribolium_castaneum                  --FMVPLVVLIFTYTSICIEIWQSSESS---L--R---P---------------------
Nicrophorus_vespilloides             --FIIPLVVLIVTYSCICREIWHSAVGE---LGMR---P---------------------
Aleochara_curtula                    ------------------------------------------------------------
Pogonus_chalceus                     --FIVPLIVLIFTYGAICRVIWQSAGGE---LGLR---S---------------------
Sipyloidea_sipylus                   --FIVPLCVLVYTYSCICHEIWEKSGFR---AAHA-------------------------
Extatosoma_tiaratum                  --FIVPLCVLVFTYSRICHEIWEKSGFR---AAHA-------------------------
Aretaon_asperrimus                   --FVIPLCVLVYTYSCICREIWEKSGFR---AAHA-------------------------
Medauroidea_extradentata             --FIIPLCVLVYTYSRICHEIWEKSGFR---AAHA-------------------------
Ramulus_artemis                      --FIIPLCVLVYTYSCICHEIWEKSGFR---AAHA-------------------------
Galloisiana_yuasai                   ------------------------------------------------------------
Athalia_rosae                        --FILPLLVLGYTNVSICLAIWRNTGIA---GEAR---G---------------------
```

```
                                     421                                                      480
Varroa_destructor1                   ------------------------------------------------------------
Metaseiulus_occidentalis1            ------------------------------------------------------------
Metaseiulus_occidentalis2            --------------------------------------------------------FG--
Tigriopus_californicus               VGDGS-------------------------------------------------KNE---
Anurida_maritima                     ----------------------------------------------------YSKNSRS-
Meinertellus_cundinamarcensis        ------------------------------------------------ICCARCKRSSK-
Pogonognathellus_sp                  ------------------------------------------------------------
Triops_cancriformis                  AGAAL---RPTSQRWRRASRLLGRFFLQRRER--------AT--HQRNSC-------G--
Strigamia_maritima2                  -----------------------------------------------A----VGNGTGG-
Catajapyx_aquilonaris                ---------------------------ASKWK--------WL--DLKKCLCCTRKESGR-
Daphnia_pulex                        TGSTL--------------SVMRRWILRASLR--------WQ--KSRSSSNNGTKNHSAI
Daphnia_magna3                       TGSTL--------------SVMRRWFLRAGMR--------WQ--KSRNSSNAIKNNS---
Daphnia_magna2                       TGSTL--------------SVMRRWFLRAGMR--------WQ--KSRNSSNAIKNNS---
Daphnia_magna1                       TGSTL--------------SVMRRWFLRAGMR--------WQ--KSRNSSNAIKNNS---
Daphnia_magna4                       TGSTL--------------SVMRRWFLRAGMR--------WQ--KSRNSSNAIKNNS---
Arachnocampa_luminosa                NGLITEVKHDSDNSIFRDKKFGILWWIRSRFNKGIRHKILFTSTKKRGKNIKFGKTGNFL
Calanus_finmarchicus                 ------------------------------------------------------------
Epiophlebia_superstes                ------------------------------------------------------------
Ladona_fulva                         ------------------------------------------------------------
```

```
Cordulegaster_boltonii              ------------------------------------------------------------
Diaphorina_citri                    ------------------------------------------------------------
Pachypsylla_venusta                 ------------------------------------------------------------
Acanthocasuarina_muellerianae       ------------------------------------------------------------
Glomeris_pustulata                  ------------------------------------------------------------
Frankliniella_cephalica             ------------------------------------------------------------
Machilis_hrabei                     ------------------------------------------------------------
Carabus_granulatus                  ------------------------------------------------------------
Dendroctonus_frontalis              ------------------------------------------------------------
Lepeophtheirus_salmonis             EGTIR-----------------YR-WKSTK--------------RALGSTAEYSKRNDTI
Caligus_rogercresseyi               EGMIR-----------------YR-WKSSK--------------RSRNNQNEV--HQNTE
Parhyale_hawaiensis                 -----------------------------K--------------V-------YHHRGNSM
Hyalella_azteca                     -----------------------------R--------------K-------YGRTLDDT
Strigamia_maritima1                 ------------------------------------------------------------
Conwentzia_psociformis              ------------------------------------------------------------
Liposcelis_bostrychophila           ------------------------------------------------------------
Tetranychus_urticae                 ------------------------------------------------------------
Osmylus_fulvicephalus               ------------------------------------------------------------
Ornithodoros_turicata               ------------------------------------------------------------
Acerentomon_sp                      -----------------------------K--------------KHRGCNGFY-------
Ixodes_ricinus                      ------------------------------------------------------------
Folsomia_candida                    ------------------------------------------------------------
Limulus_polyphemus1                 ------------------------------------------------------------
Limulus_polyphemus3                 ------------------------------------------------------------
Platynothrus_peltifer5              -----------------------------K--------------EFSTS-SLKSTSLTSF
Platynothrus_peltifer1              -----------------------------D--------------RVSSCS----STNDSI
Platynothrus_peltifer3              -----------------------------N--------------DKLDCDSVNSPETSEH
Achipteria_coleoptrata2             ------------------------------------------------------------
Platynothrus_peltifer2              -----------------------------N--------------SY-------------S
Platynothrus_peltifer4              -----------------------------------------------------------G
Ixodes_scapularis2                  ------------------------------------------------------------
Ixodes_scapularis1                  -------------------------WKGER------------------------------
Ixodes_scapularis3                  -------------------------WKGSR------------------------------
Cotesia_vestalis                    ------------------------------------------------------------
Microplitis_demolitor               ------------------------------------------------------------
Zorotypus_caudelli                  ------------------------------------------------------------
Nasonia_giraulti                    ------------------------------------------------------------
Nasonia_vitripennis                 ------------------------------------------------------------
Varroa_destructor2                  ------------------------------------------------------------
Leuctra_sp                          ------------------------------------------------------------
Tricholepidion_gertschi             ------------------------------------------------------------
Perla_marginata                     ------------------------------------------------------------
Crioscolia_alcione                  ------------------------------------------------------------
Mischocyttarus-_flavitarsis         ------------------------------------------------------------
Polistes_metricus                   ------------------------------------------------------------
Polistes_canadensis                 ------------------------------------------------------------
Chrysis_viridula                    ------------------------------------------------------------
Argochrysis_armilla                 ------------------------------------------------------------
Chyphotes_mellipes                  ------------------------------------------------------------
Brachycistis_timberlakei            ------------------------------------------------------------
Sphaeropthalma_orestes              ------------------------------------------------------------
Homalodisca_vitripennis             ------------------------------------------------------------
Cercopis_vulnerata                  ------------------------------------------------------------
Nilaparvata_lugens                  ------------------------------------------------------------
Achipteria_coleoptrata1             ------------------------------------------------------------
Ephemera_danica                     ------------------------------------------------------------
Leptopilina_clavipes                ------------------------------------------------------------
Ganaspis_sp                         ------------------------------------------------------------
Leptopilina_boulardi                ------------------------------------------------------------
Mantis_religiosa                    ------------------------------------------------------------
Blattella_germanica                 ------------------------------------------------------------
Zootermopsis_nevadensis             ------------------------------------------------------------
Stigmatomma_oregonense              ------------------------------------------------------------
Dinoponera_quadriceps               ------------------------------------------------------------
Harpegnathos_saltator               ------------------------------------------------------------
Cerapachys_biroi                    ------------------------------------------------------------
Monomorium_pharaonis                ------------------------------------------------------------
Tetramorium_bicarinatum             ------------------------------------------------------------
Solenopsis_invicta                  ------------------------------------------------------------
Atta_cephalotes                     ------------------------------------------------------------
Acromyrmex_echinatior               ------------------------------------------------------------
Pogonomyrmex_barbatus               ------------------------------------------------------------
Wasmannia_auropunctata              ------------------------------------------------------------
Vollenhovia_emeryi                  ------------------------------------------------------------
Orussus_abietinus                   ------------------------------------------------------------
Blaberus_atropos                    ------------------------------------------------------------
Limulus_polyphemus2                 ------------------------------------------------------------
```

```
Isonychia_bicolor               ------------------------------------------------------------
Occasjapyx_japonicus            ------------------------------------------------------------
Baetis_sp                       ------------------------------------------------------------
Panonychus_ulmi                 ------------------------------------------------------------
Frankliniella_occidentalis      ------------------------------------------------------------
Eurylophella_sp                 ------------------------------------------------------------
Telenomus_podisi                ------------------------------------------------------------
Aposthonia_japonica             ------------------------------------------------------------
Haploembia_palaui               ------------------------------------------------------------
Halyomorpha_halys               ------------------------------------------------------------
Stenobothrus_lineatus           ------------------------------------------------------------
Inocellia_crassicornis          ------------------------------------------------------------
Tenthredo_koehleri              ------------------------------------------------------------
Corydalus_cornutus              ------------------------------------------------------------
Chrysopa_pallens                ------------------------------------------------------------
Meloe_violaceus                 ------------------------------------------------------------
Aethina_tumida                  ------------------------------------------------------------
Gyrinus_marinus                 ------------------------------------------------------------
Tribolium_castaneum             ------------------------------------------------------------
Nicrophorus_vespilloides        ------------------------------------------------------------
Aleochara_curtula               ------------------------------------------------------------
Pogonus_chalceus                ------------------------------------------------------------
Sipyloidea_sipylus              ------------------------------------------------------------
Extatosoma_tiaratum             ------------------------------------------------------------
Aretaon_asperrimus              ------------------------------------------------------------
Medauroidea_extradentata        ------------------------------------------------------------
Ramulus_artemis                 ------------------------------------------------------------
Galloisiana_yuasai              ------------------------------------------------------------
Athalia_rosae                   ------------------------------------------------------------

                                481                                                      540
Varroa_destructor1              ------------------------------------------------------------
Metaseiulus_occidentalis1       ------------------------------------------------------------
Metaseiulus_occidentalis2       ------------------------------------------------------------
Tigriopus_californicus          ------DDDDGATMETAVYEMKVINGRNGSSSS-----HRSSC-KSTTRSINTSCTI--Y
Anurida_maritima                ---------------------------------------------VSSSPETQSEVVIEE
Meinertellus_cundinamarcensis   ----SSSDRDTVL----------------------------------------------L
Pogonognathellus_sp             ------------------------------------------------------------
Triops_cancriformis             ------DLEEDFPATTGHVLVESQSSSSGSKPVAESLPLRESDTASSTSKSSSH------
Strigamia_maritima2             ----GDTV----------------------------------------------------
Catajapyx_aquilonaris           --QSPSSVED-----------------------------NDVLEQAESSPLPQKATQLEL
Daphnia_pulex                   --ANTSQLSDTIPMRSLAT-------------------------QPASNPLSAEAKVI--
Daphnia_magna3                  --ATASQLS------------------------ADPIPMRSLATQQASNPLTTENKISPM
Daphnia_magna2                  --ATTSQLSDTIPMRSLAT----Q---------------------QASNPLSTENKISPM
Daphnia_magna1                  --ATASQLSDTIP-----------------------------------------NKISPM
Daphnia_magna4                  --ATASQLSDTIPMRSLAT----Q-QASNPLTTPDTIPMRSLATQQASNPLTTENKISPM
Arachnocampa_luminosa           ----MN--DQTMPKE---YEMRLMLKRKETLS------DSEK--YSMRLDKNSVY-----
Calanus_finmarchicus            ---DSPKLYITMAN------GKP-------------------------------------
Epiophlebia_superstes           ------------------------------------------------------------
Ladona_fulva                    ------------------------------------------------------------
Cordulegaster_boltonii          ------------------------------------------------------------
Diaphorina_citri                ------------------------------------------------------------
Pachypsylla_venusta             ------------------------------------------------------------
Acanthocasuarina_muellerianae   ------------------------------------------------------------
Glomeris_pustulata              ------------------------------------------------------------
Frankliniella_cephalica         -----------HAYA---YRFAPHQQPAPVSSN---------------------S-----
Machilis_hrabei                 ------------------------------------------------------------
Carabus_granulatus              ------------------------------------------------------------
Dendroctonus_frontalis          ------------------------------------------------------------
Lepeophtheirus_salmonis         EEVVMNNLTLSVSND---NQDNPNQIQAASSFTFDRCGKLRKSKNKVLIDLDSPT-----
Caligus_rogercresseyi           ENMMMDNFSLSVNQG---TRDNPNQIKNATSFTFDTFSHVQKGNKRVIIDLSSPV-----
Parhyale_hawaiensis             --NL-S--CPTV------YRSKPIGSLPHCSL-----------HSRSLSEKAFFG-----
Hyalella_azteca                 --THGS--SSTVNDV---NYGNAVRILSYSSA-----------TNKARNTVIEHG-----
Strigamia_maritima1             ----ATHLS--VPQ-----------NVNHIQR-------------------NGSC-----
Conwentzia_psociformis          ------------------------------------------------------------
Liposcelis_bostrychophila       ------------------------------------------------------------
Tetranychus_urticae             ------------------------------------------------------------
Osmylus_fulvicephalus           ------------------------------------------------------------
Ornithodoros_turicata           --------ELSSRRT---YRFAGRQRKMSS------------------------------
Acerentomon_sp                  --RERRSLWKRMSMS---SNSQSMTNSNVSAETI-------KLTLDEIDELSTVN-----
Ixodes_ricinus                  -----------GSRA---YRFKGTAQN---------------------------G-----
Folsomia_candida                ------------------------------------------------------------
Limulus_polyphemus1             ------------------------------------------------------------
Limulus_polyphemus3             ------------------------------------------------------------
Platynothrus_peltifer5          --SG----PIKSPIT---YSIRPSKRLRRIHSQI-------DLKKDLRNSLTNLS-----
Platynothrus_peltifer1          --ARSTNLLVTVGRS---YRFKGRGRVEVSVD----------------------G-----
```

```
Platynothrus_peltifer3            --RSGTRLLVTVGKS---YRFKGKGQVEVSVE----------------------K-----
Achipteria_coleoptrata2           -----------VGKS---YRFKSRGKLEVSVD----------------------K-----
Platynothrus_peltifer2            --RTRKCLLIALNRV---YRLGRRQDTETAEIQI-------NCDNQEVRPLHTVV-----
Platynothrus_peltifer4            -------------------KLSKSSDDKSRLQSD-------RC-----------------
Ixodes_scapularis2                ------------------------------------------------------------
Ixodes_scapularis1                -----------------AYRFRGLAHN---------------------------------
Ixodes_scapularis3                -----------------AYRFKGTAQN---------------------------------
Cotesia_vestalis                  ------------------------------------------------------------
Microplitis_demolitor             ------------------------------------------------------------
Zorotypus_caudelli                ------------------------------------------------------------
Nasonia_giraulti                  ------------------------------------------------------------
Nasonia_vitripennis               ------------------------------------------------------------
Varroa_destructor2                ------------------------------------------------------------
Leuctra_sp                        ------------------------------------------------------------
Tricholepidion_gertschi           ----------------------VGQ-----------------------------------
Perla_marginata                   ------------------------------------------------------------
Crioscolia_alcione                ------------------------------------------------------------
Mischocyttarus-_flavitarsis       ------------------------------------------------------------
Polistes_metricus                 ------------------------------------------------------------
Polistes_canadensis               ------------------------------------------------------------
Chrysis_viridula                  ------------------------------------------------------------
Argochrysis_armilla               ------------------------------------------------------------
Chyphotes_mellipes                ------------------------------------------------------------
Brachycistis_timberlakei          ------------------------------------------------------------
Sphaeropthalma_orestes            ------------------------------------------------------------
Homalodisca_vitripennis           ------------------------------------------------------------
Cercopis_vulnerata                ------------------------------------------------------------
Nilaparvata_lugens                ------------------------------------------------------------
Achipteria_coleoptrata1           ------------------------------------------------------------
Ephemera_danica                   ----------------------LQP-N---------------------------------
Leptopilina_clavipes              ------------------------------------------------------------
Ganaspis_sp                       ------------------------------------------------------------
Leptopilina_boulardi              ------------------------------------------------------------
Mantis_religiosa                  ------------------------------------------------------------
Blattella_germanica               ------------------------------------------------------------
Zootermopsis_nevadensis           ------------------------------------------------------------
Stigmatomma_oregonense            ------------------------------------------------------------
Dinoponera_quadriceps             ------------------------------------------------------------
Harpegnathos_saltator             ------------------------------------------------------------
Cerapachys_biroi                  ------------------------------------------------------------
Monomorium_pharaonis              ------------------------------------------------------------
Tetramorium_bicarinatum           ------------------------------------------------------------
Solenopsis_invicta                ------------------------------------------------------------
Atta_cephalotes                   ------------------------------------------------------------
Acromyrmex_echinatior             ------------------------------------------------------------
Pogonomyrmex_barbatus             ------------------------------------------------------------
Wasmannia_auropunctata            ------------------------------------------------------------
Vollenhovia_emeryi                ------------------------------------------------------------
Orussus_abietinus                 ------------------------------------------------------------
Blaberus_atropos                  ------------------------------------------------------------
Limulus_polyphemus2               ------------------------------------------------------------
Isonychia_bicolor                 ----------------------TK------------------------------------
Occasjapyx_japonicus              ------------------------------------------------------------
Baetis_sp                         ----------------------PTDSK---------------------------------
Panonychus_ulmi                   ------------------------------------------------------------
Frankliniella_occidentalis        ------------------------------------------------------------
Eurylophella_sp                   ----------------------HHQH----------------------------------
Telenomus_podisi                  ------------------------------------------------------------
Aposthonia_japonica               ------------------------------------------------------------
Haploembia_palaui                 ------------------------------------------------------------
Halyomorpha_halys                 ------------------------------------------------------------
Stenobothrus_lineatus             ------------------------------------------------------------
Inocellia_crassicornis            ----------------------PMSV----------------------------------
Tenthredo_koehleri                ------------------------------------------------------------
Corydalus_cornutus                ------------------------------------------------------------
Chrysopa_pallens                  ----------------------MS------------------------------------
Meloe_violaceus                   ----------------------RS------------------------------------
Aethina_tumida                    ----------------------RNSL----------------------------------
Gyrinus_marinus                   ------------------------------------------------------------
Tribolium_castaneum               ----------------------RS------------------------------------
Nicrophorus_vespilloides          ----------------------RNVQ----------------------------------
Aleochara_curtula                 ------------------------------------------------------------
Pogonus_chalceus                  ----------------------PRLA----------------------------------
Sipyloidea_sipylus                ------------------------------------------------------------
Extatosoma_tiaratum               ------------------------------------------------------------
Aretaon_asperrimus                ------------------------------------------------------------
Medauroidea_extradentata          ------------------------------------------------------------
```

```
Ramulus_artemis                    ------------------------------------------------------------
Galloisiana_yuasai                 ------------------------------------------------------------
Athalia_rosae                      ------------------------------------------------------------


                                   541                                                      600
Varroa_destructor1                 ------------------------SKRSLSHTKLNPIRMTMVMVIVFVLCWTPFCCAQLY
Metaseiulus_occidentalis1          ------------------------SKRLMSDAKLKTIRMTMVMVLVFVLCWTPFCCAQLY
Metaseiulus_occidentalis2          ------------VN----TAGFFESNRRITKAKVRMVKMTFTVVLCFIVCWSPYSIAELL
Tigriopus_californicus             RP---NLAHNSHAPTGGFVRVQTEQIVLLSKAKRKSLSMTAIVSVCFAVCWLPWCVTMLL
Anurida_maritima                   APTPK-------------DVGSRRLAQQISEVKIKTLKLTLVVVLCFFVCWSPFCVTQLI
Meinertellus_cundinamarcensis      RP------------IQSRNTSQRSQSARLSRARMRTLRLTLAVVACFIICWAPFCVSQLV
Pogonognathellus_sp                ------------------------------------------------------------
Triops_cancriformis                ---------GK-KIKMEPFPLRRSNSHRITRAKIKTIQLTLLVIISFVVCWAPFCITQLV
Strigamia_maritima2                --------TDS-CRLYSRRVAGVSNPNPISEAKVKTLKLTFLVVVCFFVCWSPFCITQLV
Catajapyx_aquilonaris              -------AGRL-KSKVG-CNVKQKRKSMISDAKIKTLQLTLAVVICFFVCWAPFCIAQLI
Daphnia_pulex                      RSLPPPLSPQC-CQQMP-LRRSNSNQNRITKAKMKTIKLTLAVVLCFVACWAPFCITQLI
Daphnia_magna3                     LPQPPPAAPQR-CQQMS-LRRSNSNQNRITKAKMKTIKLTLAVVLCFVACWAPFCITQLI
Daphnia_magna2                     LPQPPPAAPQR-CQQMS-LRRSNSNQNRITKAKMKTIKLTLAVVLCFVACWAPFCITQLI
Daphnia_magna1                     LPQPPPAAPQR-CQQMS-LRRSNSNQNRITKAKMKTIKLTLAVVLCFVACWAPFCITQLI
Daphnia_magna4                     LPQPPPAAPQR-CQQMS-LRRSNSNQNRITKAKMKTIKLTLAVVLCFVACWAPFCITQLI
Arachnocampa_luminosa              -------S---KSFKSNFPIRQTFSGKALTRSKIKTLKLTLTVVITYVMCSLPFYVCTFI
Calanus_finmarchicus               -------------KSKLSVQNFRSTSVMVSRAKIKSVKLTLVIIACYILCSAPFIVGQLL
Epiophlebia_superstes              ------------------APPRTHSVGTISKAKMKTLRLTIVAVACYILCSCPFVLSQLW
Ladona_fulva                       ------------------APPRTHSMGTISRAKMRTLRLTIVAVACYILCSCPFVLSQLW
Cordulegaster_boltonii             ------------------APLRTHSMGTISRAKMRTLRLTIVAVACYILCSCPFVLSQLW
Diaphorina_citri                   ---------------------TTSPHKIISRAKINTIKQTIAVITLYIVCSLPFIFTQLW
Pachypsylla_venusta                ---------------------APSPHKIISRAKINTVKQTITVITLYIICSLPFIFAQLW
Acanthocasuarina_muellerianae      --------------------------KIISRAKINTVKQTVAVITLYIICSLPFIFAQLW
Glomeris_pustulata                 ----------------------------------KTVKLTVVVILSYIACSMPYISAMLW
Frankliniella_cephalica            -----------FAYARSQQQATQRTNPLISRAKINTVKQTVLVIAIYIICSAPFICVQLW
Machilis_hrabei                    ---------------------------GISRAKIKTVKLTVVVIACYVTCSTPFICAELW
Carabus_granulatus                 -----------------------------SRAKINTVKQTIAVIAMYIICSTPFICAQLW
Dendroctonus_frontalis             ----------------SKQSTKRDRVPLISRAKINTVKQTIAVIVLYLACSAPFIVAQLW
Lepeophtheirus_salmonis            -----------QTKNKKSLKPRTHSIQGISRAKIKTIKLTVVVIAGYIACSAPFICVQLW
Caligus_rogercresseyi              -----------QKQKRKSLKPRSHSIQGISRAKIKTIKLTVVVIVGYIACSAPFICVQL-
Parhyale_hawaiensis                -------QKMKQNSLSMKKQRVSRIMSPISRAKIKTVKLTLIVIFFFVACSAPFISVQLW
Hyalella_azteca                    -------CR----YRFQKTPGAKTVMSPISRAKIKTIKLTMIVIFFFIVCSAPFITSLMW
Strigamia_maritima1                -------RF----KASESVNPRSHGLQRISRAKIKTVKLTVVVITCYILCSMPYTCAMMW
Conwentzia_psociformis             ------------------------------------------------------------
Liposcelis_bostrychophila          ------------------------------------------------------------
Tetranychus_urticae                -----------------ANLSQSHSTAYFSKAKIKTVKLTVTIIICYICCSSPFNVVQIW
Osmylus_fulvicephalus              ------------------------------------------------------------
Ornithodoros_turicata              -------------ANGSFVAPRSHSVRGLSRAKIKTVKITVVVIVFYVVCSSPFICVQMW
Acerentomon_sp                     -------EN----SEILDMAMRST---------IVPV-------------TEPQPVTQQS
Ixodes_ricinus                     -------TK----QDSSFVGPRSHSVRGLSRAKIKTVKITVVVIALYVVCSSPFICVQMW
Folsomia_candida                   ------------------------------------------------------------
Limulus_polyphemus1                ------------------NSSRSHSMRGLSRAKVKTVRITVVVIACYIICSTPFIVVQLW
Limulus_polyphemus3                ------------------NFIHCRSVPDLSRAKVKTIRITIVVIACYIICSTPFLAVQLW
Platynothrus_peltifer5             -------VK----VNDLNSTPRSHSLRFVSRAKIKTVKITIVIMLCYVICSMPFISVQLW
Platynothrus_peltifer1             -------PK----SR-GTYNPRSHSLNGLSRAKIKTVKITVVVILCYIICSSPFICVQLW
Platynothrus_peltifer3             -------DC----GKEDYNSPRTHSIHGLTRAKIKTVKITIVIIMCYIFCSTPFICVQLW
Achipteria_coleoptrata2            -------DF----KE--DFSPRTHSIHGLSRAKIKTVKITVVIILCYIMCSTPFICVQLW
Platynothrus_peltifer2             -------TF----QESVSSSPRVNTFNRLSRAKIKTVKITVVVILCYVICSSPFICVQLW
Platynothrus_peltifer4             -------------LANNLLNPRSHSIYRISKAKIKTVKVTVVVVICYIVCSSPFICVQVW
Ixodes_scapularis2                 -----------------------HSVRGMSRAKVKTVKITVVVIALYIVCSSPFICVQMW
Ixodes_scapularis1                 ----------GTKQDSSFVGPRSHSVRGLSRAKIKTVKITVVVIALYVVCSSPFICVQMW
Ixodes_scapularis3                 ----------GTKQDSSF-----------------TVKITVVVIALYVVCSSPFICVQMW
Cotesia_vestalis                   ------------------------------------------------------------
Microplitis_demolitor              -------------NNKKINFNNKNRQPLISRAKIKTVKQMITVVSLYVMTSSPFIGCQLW
Zorotypus_caudelli                 ------------------------------------------------------------
Nasonia_giraulti                   --------------QQALTKEGRSQTTLISKAKINTVKQTLAVVTLYAASSIPFVGCQLW
Nasonia_vitripennis                --------------QQALTKEGRSQTTLISKAKINTVKQTLAVVTLYAASSIPFVGCQLW
Varroa_destructor2                 ------------------------------------------------------------
Leuctra_sp                         ------------------------------------------------------------
Tricholepidion_gertschi            -----------ADVRPAPVNPRTHSMKGISRAKIKTVKLTVVVIACYIFCSSPFISVQLW
Perla_marginata                    ------------------------------------------------------------
Crioscolia_alcione                 ------------IKG--DVFVEKNRSTFVSKAIISSVKQTIIVVTLYLVTGSPFIGCQLW
Mischocyttarus-_flavitarsis        ------------YIRS-ISSCTRNRSSFISKAMINTVKQTIAVITLYAITSTPFIGCELW
Polistes_metricus                  ------------YIRS-ISNYSRNRSSFISKAMINTVKQTIAVITLYAITSIPFIGCELW
Polistes_canadensis                ------------YIRS-ISNYSRNRSSFISKAMINTVKQTIAVITLYAITSIPFIGCELW
Chrysis_viridula                   ------------KVRNNGNFLERNRSPFISKAMINTMRQTIVVISLYAITSIPFIGCQLW
Argochrysis_armilla                ------------KSRNSVNILERNRSPFISKAMINTMRQTIVVISLYAITSIPFIGCQLW
Chyphotes_mellipes                 ------------SVKG-KKSFDQNRTPLVSKAMINTVKQTIAVISLYTITSTPFIGCQLW
Brachycistis_timberlakei           ------------RGGKSGKKLEGNRSPFISKAMINTVRQTIAVISLYTISSTPFIGCQLW
Sphaeropthalma_orestes             ------------RAKG-SNLPPRNRSPFISRAMINTVKQTIAVISLYTITSTPFIGCQLW
Homalodisca_vitripennis            -----------------------SPHHLISRAKINTIKQTVAVILLYISCSSPFICAQLW
```

```
Cercopis_vulnerata                  -----------------------SPHRLISRAKINTIKQTVAVIALYISCSSPFICAQLW
Nilaparvata_lugens                  -----------------------SPHRLISRAKVNTVKQTVAVIALYVACSSPFIVAQLW
Achipteria_coleoptrata1             ------------------------------------------------------------
Ephemera_danica                     ----------FHFYPRIPMEPRTHSLRGISRAKLRSIKLTIVVIACYVICSTPFIGAQLW
Leptopilina_clavipes                ---------------KNSNIIKKKAKSINNKGKSKYDETNNCCC----------------
Ganaspis_sp                         ---------------KNSHFTKKKQSPLITKARVNTMKQTIVVVTLYFLTWSPFIGCELW
Leptopilina_boulardi                ---------------KNSNIIKKKQSPLITKARVNTMKQTIVVVTLYFLTWSPFIGCELW
Mantis_religiosa                    ------------------------------------------------------------
Blattella_germanica                 ------------------------------------------------------------
Zootermopsis_nevadensis             ------------------------------------------------------------
Stigmatomma_oregonense              ------------KKGSKDNFLQRNRDPFISNALINTVKQTIVVVTLYIVTSIPFIGCELW
Dinoponera_quadriceps               ------------RKGNKASFSQRNRDPFISKALINTVKQTIIVVILYIVTSTPFIGCQLW
Harpegnathos_saltator               ------------KK---ASFSQRNRDPFISKALINTVKQTIVVVTLYIVTSIPFIGCQLW
Cerapachys_biroi                    ------------KQGNKASFSLRNRDPFISKALINTIKQTIVVVSLYIITSIPFIGCELW
Monomorium_pharaonis                ------------KKGNKANFSQRNRDPLISKAMINTIKQMIVVVTLYIITNTPFIGCELW
Tetramorium_bicarinatum             ------------KKNNRASFFQRNRDPLISKAMINTVKQMIIVVTLYIITNTPFIGCELW
Solenopsis_invicta                  ------------KKGNKASFCQRNRDPLISKAMINTVKQMIVVVTLYIITNTPFIGCELW
Atta_cephalotes                     ------------KKSNKVSFCQRNRDPLISKAMINTVKQMIVVVILYIVTNTPFIGCELW
Acromyrmex_echinatior               ------------KRSNKISFCQRNRDPLISKAMINTVKQMIVVVTLYIVTNTPFIGCELW
Pogonomyrmex_barbatus               ------------KRRNKASFSQRNRDPLISKAMINTVKQTIVVVTLYIITNIPFIGCELW
Wasmannia_auropunctata              ------------KRDNKASFCQRNRDPLISKAMINTVKQMIIVVTLYIVTNIPFIGCELW
Vollenhovia_emeryi                  ------------KRSNKASFFQRNRDPIISKAMINTVKQTIVVVTLYILTNSPFIGCELW
Orussus_abietinus                   ------------------PRGDIRAPATISRAKIRTAWQTIVVVALYIASSTPFIGCELW
Blaberus_atropos                    ----------------------------------NTVKQMVAVLFLYVACSAPFISAQLW
Limulus_polyphemus2                 ---------------ETFTVPRSHCMRGLSRSKVKTFKITVVVITCYIICSTPFIIVQLW
Isonychia_bicolor                   ---------------QTNIQPRVHSIRGISRAKIKTVKLTVVVIACYIACSSPFISAQLW
Occasjapyx_japonicus                ------------------------------------------------------------
Baetis_sp                           ----------VLGNG-RVMHPRVHSMRGISRAKMKTIKLTVVVIACYVVCSTPFICAQLW
Panonychus_ulmi                     ------------------------------------------------------------
Frankliniella_occidentalis          ------------------------------------------------------------
Eurylophella_sp                     -----------I--VRNMNNPRVHSIRGISRAKMKTVKLTVVVIACYIFCS---------
Telenomus_podisi                    ---------------KNANFRGDNRANLITKARIKTVKQTIAVITLYATTNSPFIGCELW
Aposthonia_japonica                 -----------------------QTSPVISRAKINTVKQMVIVILFYIICSTPFTFSLLW
Haploembia_palaui                   -----------------------KSSPVISRAKINTVKQMVIVIFFYIICSTPFTC----
Halyomorpha_halys                   ------------------------PHKVVSRAKINTIKQTVAVIALYIACSLPFISAQLW
Stenobothrus_lineatus               ------------------------------------------------------------
Inocellia_crassicornis              -----------TTAPTHGPVRGRRTQPFVSRAKINTVKQTVAVIVTYIVCSMPFISAQLW
Tenthredo_koehleri                  ---------------T--HGGLLRQRTVISRAKINSIKQMIAVISLYAASSSPFIASLLW
Corydalus_cornutus                  ------------------------------------------------------------
Chrysopa_pallens                    --------------SP--PCTPKRRAPLISRAKINTVKQTVAVIILYIASSSPFISAQLW
Meloe_violaceus                     ------------------SQKAGKRTPLISRAKINTVKQTIAVIVMYIVCSTPFILAQLW
Aethina_tumida                      -----------VGK-------KRTKMPLISRAKINTVKQTIAVIFMYIICSSPFMFVQLW
Gyrinus_marinus                     ------------------------------------------------------------
Tribolium_castaneum                 --------------SQ--KSAPGKRTPLISRAKINTVKQTIAVIVMYIACSTPFILAQLW
Nicrophorus_vespilloides            -----------TGNGK--SQQTGKRAPLISRAKINTVKQTIAVIVMHIVCSTPFISGQLW
Aleochara_curtula                   ------------------------------------------------------------
Pogonus_chalceus                    -----------PDSPP--RCARSRRAPLISRAKINTVKQTVAVIAMYIACSTPFICAQLW
Sipyloidea_sipylus                  ---------------A--RSSPLKMNPTISRAKINTVKQTVAVIVLYIACSSPFIGAQLW
Extatosoma_tiaratum                 ---------------A--RSSPLKMNPTISRAKINTVKQTVSVIVLYISCSSPFIGAQLW
Aretaon_asperrimus                  ---------------A--RSSPLKMNPTISRAKINTVKQTVAVIVLYISCSLPFIGAQLW
Medauroidea_extradentata            ---------------A--RSSPLKINPTISRAKINTVKQTVAVIVLYISCSSPFIGAQLW
Ramulus_artemis                     ---------------A--RSSPLKMNPTISRAKINTVKQTVAVIMLYISCSSPFIGAQLW
Galloisiana_yuasai                  ------------------------------------------------------------
Athalia_rosae                       ---------------T--DGTLLRQGSVISRAKINSIKQMVAVISFYAASSSPFVASLLW


                                    601                                    CXNPW motif       660
Varroa_destructor1                  LES-T-----GQ---VPSIFITLFLL-VPNL------NSCANPWVCLTFSTTLRRKLTDA
Metaseiulus_occidentalis1           LVF-G-----GE---EASTFVTMCLM-VPNL------NSCANPWVYLSFSTDLRRRLVNF
Metaseiulus_occidentalis2           LAY-KIAG--GE---HVSPPFMVFLL-LASL------NSAVNPWIYTAFTNSFACK----
Tigriopus_californicus              MSF-DIQV-GGD---KVHPLMVIFAL-LASL------NSTTNPWIYLCFSSAVLQQVKHL
Anurida_maritima                    LVY-NPTL-------PVGPGNVILLL-LPSL------NSCTNPWIYLAFSESLCNQLRVF
Meinertellus_cundinamarcensis       YNF-NPPT-DQK---QMSGVTVIFLL-LASL------NSCTNPWLYLAFSGSLLNHLRVC
Pogonognathellus_sp                 ------------------------------------------------------------
Triops_cancriformis                 LVY-DPPS-N--------------------------------------------------
Strigamia_maritima2                 LTF-NPPS-PDA---QIGSVEVILLL-LASL------NSCTNPWIYVAFSGSLLNQLRMC
Catajapyx_aquilonaris               LVY-NPPE--DR---DVDPVTVILLL-LASL------NSCTNPWIYLTFSGSLLNQLRV-
Daphnia_pulex                       MVY-CPPT-SQA---DVSPVAVIILL-LASL------NSCSNPWIYLAFSGSLLNQMRVC
Daphnia_magna3                      MVY-CPPT-SQA---DVSPVAVIILL-LASL------NSCSNPWIYLAFSGSLLNQMRVC
Daphnia_magna2                      MVY-CPPT-SQA---DVSPVAVIILL-LASL------NSCSNPWIYLAFSGSLLNQMRVC
Daphnia_magna1                      MVY-CPPT-SQA---DVSPVAVIILL-LASL------NSCSNPWIYLAFSGSLLNQMRVC
Daphnia_magna4                      MVY-CPPT-SQA---DVSPVAVIILL-LASL------NSCSNPWIYLAFSGSLLNQMRVC
Arachnocampa_luminosa               HFLLGLSAHNHSSLFTK----TLVYI-TISTNMLFQLNSCANPFIYLFFNGNICKLAKRL
Calanus_finmarchicus                ATL-GPPH-IRSKIGLQ---MEPLFW-LMTL------NSLVNPWIYIWFNRNTLFVRQTS
Epiophlebia_superstes               VTW-YPGA-TESSFWKG-AAFTILSL-MSCL------NSCVNPWIYMAFNEDIRVALKER
Ladona_fulva                        ATW-YPGA-TESSFWRG-AAFTILSL-LPCL------NSCVNPWIYMAFNEEIRVALRER
Cordulegaster_boltonii              ATW-YPGA-TESPFWRG-AAFTILSL-LPCL------NSCVNPWIYMAFNEDIRIALKER
```

```
Diaphorina_citri                    QTW-NLNQ-TVYPVFQDNAVLTIIML-LSSL------NSCVNPWIYLVSNRELIAALRTL
Pachypsylla_venusta                 ETW-NLNQ-TVYPVFQDSA-LTIIML-LSSL------NSCVNPWIYLVSNRELITALWTL
Acanthocasuarina_muellerianae       QTW-NLNE-SFYPVFQDNAVLTIIML-LSSL------NSCVNPWIYLFSNRKLITALRTL
Glomeris_pustulata                  LVW-YPEA-RNTPFGRS-AAFTILAL-LASL------NSCANPWIYLCFNENVAQTLGDV
Frankliniella_cephalica             AAW-GNA---DNPFFQG-RYMTILTL-LFSL------NSCVNPWIYLAFNNDLVRMLWLL
Machilis_hrabei                     AAW-DPNA-VHSSFWQG-PAFTILTL-LASL------NSCVNPWLYLVFNPNLVRDFRHG
Carabus_granulatus                  ATW-DPE----STFLNG-AAYTILTL-LYSL------NSCVNPWIYLAFNRELPRLLLRH
Dendroctonus_frontalis              ATF-DPK----SAFLEG-STFTILTL-LYSL------NSCVNPWIYLFFNRELPRLLIRH
Lepeophtheirus_salmonis             VTF-GTVT-ESIRK----------------------------------------------
Caligus_rogercresseyi               ------------------------------------------------------------
Parhyale_hawaiensis                 SVW-DPNA-KNSPFSSG-E-----------------------------------------
Hyalella_azteca                     SVW-DP------------------------------------------------------
Strigamia_maritima1                 VAY-DKRA-QETAFYKG-GLFPVLVL-VASL------NSCVNPWVYLLFNKNLVHTLRHY
Conwentzia_psociformis              ------------------------------------------------------------
Liposcelis_bostrychophila           ------------------------------------------------------------
Tetranychus_urticae                 AYW-APGA-QESSLWRG-PLITIAML-LPSL------NSCVNPWIYLFFNRNLLSTLDKL
Osmylus_fulvicephalus               ------------------------------------------------------------
Ornithodoros_turicata               MYW-SPDA-ESSELWSS-STVTILML-LNSL------NSCVNPWIYLAFNKNLLNALRTA
Acerentomon_sp                      ISF-FQSV-SS----------TSS-A-LPVTRTTSNTSSCESTSVH--------------
Ixodes_ricinus                      MYW-SPDA-DMADTWTN-TAGSSVTQ-AEGSYIRCGKTKCRNVFSSLLSTSNV---GKQF
Folsomia_candida                    ------------------------------------------------------------
Limulus_polyphemus1                 AYW-SPYA-QTSPIWKG-PTVAILML-LASL------NSCVNPWIYLAFNHNLVTALKHI
Limulus_polyphemus3                 AYW-SPYA-QNSPIWKG-PTVAILML-LASL------NSCVNPWIYLAFNHNLITALKQL
Platynothrus_peltifer5              VYW-FPSA-QSFFQTS---ELSNIY-----------------------------------
Platynothrus_peltifer1              AYW-WPNA-QNSTFWSG-KCSENSFI-IQFR------YS-----FQ--------------
Platynothrus_peltifer3              AYW-WPNA-QQSPIWNV-KYTYLSNN-FA-------------------------------
Achipteria_coleoptrata2             AYW-VPTA-QKSHIWNV-KYTYNIYL-MFFS------IIDVTIAFKILYSE---------
Platynothrus_peltifer2              AYW-WPNA-STTTFWNG-MFSLFII-----------------------------------
Platynothrus_peltifer4              AYW-YPSA-QETSFWTG-KH----------------------------------------
Ixodes_scapularis2                  MYW-SPD-VDLADVWIN-ATVTILML-LNSL------NSCVNPWVYLFFNRSLVHTLRHQ
Ixodes_scapularis1                  MYW-SPH-VDMANPWIS-E-----------------------------------------
Ixodes_scapularis3                  MYW-SPD-AD--------------------------------------------------
Cotesia_vestalis                    ------------------------------------------------------------
Microplitis_demolitor               ATW-DPQ-AANSSFING-PAFTILTL-MSSL------TSCVNPWIYLGFNRELRNILSNY
Zorotypus_caudelli                  ------------------------------------------------------------
Nasonia_giraulti                    ATW-DPF-ASSSAFFDG-PIFTILSL-LSSL------TSCVNPWIYLTFSYELRAALTKF
Nasonia_vitripennis                 ATW-DPF-ASSSAFFDG-PIFTILSL-LSSL------TSCVNPWIYLTFSYELRAALTKF
Varroa_destructor2                  ------------------------------------------------------------
Leuctra_sp                          ------------------------------------------------------------
Tricholepidion_gertschi             ATW-DPN-ASKSPFWTG-TTFTIFTL-LASL------NSCVNPWIYLTFNRDLVRLLNQV
Perla_marginata                     ------------------------------------------------------------
Crioscolia_alcione                  ATW-DPR-AISSPFFSG-AAFTILTL-LSSL------TSCVNPWIYLSFNRELRAALTDY
Mischocyttarus-_flavitarsis         VTW-DTE-AIKSSFANG-PAFTILTL-LNSL------TSCVNPWIYLSFNRELRQNLMNY
Polistes_metricus                   ISW-DPE-APKSSFANG-PAFTILAL-LNSL------TSCVNPWIYLSFNRELRQTLMNY
Polistes_canadensis                 VSW-DTK-AFESSFANG-PAFTILAL-LNSL------TSCVNPWIYLSFNRELRQTLMNY
Chrysis_viridula                    ATW-DPS-ASSSSFING-PAFTILSL-LSSL------TSCVNPWIYLSFNRELRAVLKDN
Argochrysis_armilla                 ATW-DPS-ASSSPFITG-PAFTILSL-LSSL------TSCVNPWIYLSFNRELRSALKDN
Chyphotes_mellipes                  ATW-YPK-ATVSPFFSG-ATFTILAL-LSSL------TSCVNPWIYLSFNTELRVALTDY
Brachycistis_timberlakei            ATW-DPK-AAFSPFFTG-AAFTILAL-LSSL------TSCVNPWIYLSFNRELRLALKEY
Sphaeropthalma_orestes              ATW-DPK-ASSSPFFSG-PAFTILAL-LSSL------TSCVNPWIYLSFNQELRVALMDY
Homalodisca_vitripennis             ASW-GPR----QPFFDG-AAFTILSL-LSSL------NSCVNPWIYLSFNRELLRSLWRA
Cercopis_vulnerata                  ASW-DPN-ALSSPFLQG-ATFTILTL-LS-------------------------------
Nilaparvata_lugens                  AVW-DPG-AVHSPFFQG-ATFTILSL-LSSL------NSCVNPWIYLVSNKELMRGLRQL
Achipteria_coleoptrata1             ------------------------------------------------------------
Ephemera_danica                     ATW-DPE-AMQSPFWSG-PTFTIVTL-LASL------NSCVNPWIYLAFNPELARLLITR
Leptopilina_clavipes                ------------------------------------------------------------
Ganaspis_sp                         MAW-DPIRAANSSIFDG-PLFTVLSL-LSCL------TSCVNPWIYMAFNSELRILLINF
Leptopilina_boulardi                MAW-DPVRASNSSIFDG-PLFTVLSL-LSCL------TSCVNPWIYIAFNSELRILLLNF
Mantis_religiosa                    ------------------------------------------------------------
Blattella_germanica                 ------------------------------------------------------------
Zootermopsis_nevadensis             ------------------------------------------------------------
Stigmatomma_oregonense              ATW-DPK-ASTSPFFSG-ATFTILSL-LNSL------TSCVNPWIYFTFNSELRVAVTNF
Dinoponera_quadriceps               ATW-DPG-AATSPFHT--AAFTILCL-LNSL------TSCINPWIYFAFNRELRSALTNL
Harpegnathos_saltator               ATW-DPR-AAISPFLTG-AAFTILCL-LNSL------TSCVNPWIYFAFNRELRVALTNF
Cerapachys_biroi                    ATW-DPK-ASTSPFFTG-AAFTILSL-LNSL------TSCVNPWIYFAFNRELRAALTNF
Monomorium_pharaonis                ATW-DPK-ASSSPFFTG-AAFTILSL-LNSL------TSCVNPWIYFAFNKELRMALINF
Tetramorium_bicarinatum             ATW-DPT-ASSSPFFSG-AAFTILSL-LNSL------TSCVNPWIYFAFNKELRGALTSS
Solenopsis_invicta                  ATW-NPK-ASSSPFITG-AAFTILSL-LNSL------TSCVNPWIYFAFNKELRAALTNF
Atta_cephalotes                     ATW-DPK-ASTSPFFTG-AAFTILSL-LNSL------TSCVNPWIYFAFNKELRVALTCF
Acromyrmex_echinatior               ATW-DPK-ASTSPFFTG-AAFTILSL-LNSL------TSCVNPWIYFAFNKELRVALTCF
Pogonomyrmex_barbatus               ATW-DPK-ASTSPFFTG-AAFTILSL-LNSL------TSCVNPWIYFAFNKELRTALTNF
Wasmannia_auropunctata              ATW-DPK-ASTSPFFSG-AAFTILSL-LNSL------TSCVNPWIYFAFNKELRAALINF
Vollenhovia_emeryi                  ATW-DPT-ASSSPFFTG-AAFTILSL-LNSL------TSCVNPWIYFAFNKELRAALTNF
Orussus_abietinus                   ATW-DPG-ASSSAFLNG-PAFTILAL-LSSL------NGCVNPWVHLGFDPELREVILRH
Blaberus_atropos                    ATW-DDL-AHTRPFFSG-TAFTILAL-LYSL------NSCMNPWIYLTFNRELIRFLTQQ
Limulus_polyphemus2                 AYW-SPN-AKNSSIWKG-PTVAILML-LASL------NSCVNPWIYLAFNYNLVTALREV
Isonychia_bicolor                   ATW-DPM-AMHS------------------------------------------------
```

```
Occasjapyx_japonicus                 ------------------------------------------------------------
Baetis_sp                            ATW-DPD-AMQSPFWSG-TQPTTHLHSMFLL------HHAR-------------------
Panonychus_ulmi                      ------------------------------------------------------------
Frankliniella_occidentalis           ------------------------------------------------------------
Eurylophella_sp                      ------------------------------------------------------------
Telenomus_podisi                     AVW-SDT-AASSPFITG-PAFTILSL-LSSL------TSCVNPWIYLTFNYELRKILLKR
Aposthonia_japonica                  ATW-DVT-AVELPFFKG-PAFTILTL-LSSL------NSCVNPWIYLTFNKELIALLVNC
Haploembia_palaui                    ------------------------------------------------------------
Halyomorpha_halys                    ATW-DPH-ATSSPFLSG-GTFTILTL-LSSL------NSCVNPWIYLTSNQDLMEALWET
Stenobothrus_lineatus                ------------------------------------------------------------
Inocellia_crassicornis               VTW-YPH-AMESPFFSG-STFTILTL-LSSL------NSCVNPWIFLLFNKDLFRLLKRL
Tenthredo_koehleri                   VTW-DRN-ASASPFYSG-AAFTILTL-LSSL------NSCVNPWIYLTLNRDLSRALINR
Corydalus_cornutus                   ------------------------------------------------------------
Chrysopa_pallens                     ATW-DPY-AYKSPFWTG-ATFTILTL-LSSL------NSCVNPWIYLSFNKELRKLLKHI
Meloe_violaceus                      ATW-DPQ----NPFSEG-PVFVILAL-LYSL------NSCVNPWIYLTFNRELPRLLVKH
Aethina_tumida                       ATV-DPT----NPFFQG-PIFTILTL-LYSL------NSCVNPWIYLAFNRELPKYLLRH
Gyrinus_marinus                      ------------------------------------------------------------
Tribolium_castaneum                  ATW-DPQ----SPFIDG-PVFVILTL-LYSL------NSCVNPWIYLAFNRELPRLLLRH
Nicrophorus_vespilloides             ATW-DPE-AVNSPFLNG-PVFTIVSL-LYSL------NSCFNPWIYLAFNRELPRLLIRH
Aleochara_curtula                    ------------------------------------------------------------
Pogonus_chalceus                     ATW-DPE-ANESAFFNG-AAFTILTL-LSSL------NSCVNPWIYLAFNRELPRLLLRH
Sipyloidea_sipylus                   ATW-DPH-ASDSPFFTG-AAFTILTL-LSSL------NSCVNPWIYLAFNTELVQLLAR-
Extatosoma_tiaratum                  ATW-DPH-ATESPFFTG-AAFTILAL-LSSL------NSCVNPWIYLAFNTELVHLLATC
Aretaon_asperrimus                   ATW-DPH-AAQSAFFTG-AAFTILAL-LSSL------NSCVNPWIYLAFNTELVHLLTNR
Medauroidea_extradentata             ATW-DPH-ASESAFLTG-AAFTILAL-LSSL------NSCVNPWIYLAFNTELVRLLLKK
Ramulus_artemis                      ATW-DPH-ASESAFFTG-AAFTILAL-LSSL------NSCVNPWIYLAFNTELVRLLVKR
Galloisiana_yuasai                   ------------------------------------------------------------
Athalia_rosae                        VTW-DPG-AVNSPFFEG-AAFAILTL-MSSL------NSCVNPWIYLTLNRELPRTLFAR

                                     661                                                      720
Varroa_destructor1                   LSFFGF-------CEHLS------------------------------------------
Metaseiulus_occidentalis1            CSLIHL-------RGNRY------------------------------------------
Metaseiulus_occidentalis2            -SLRTL-------KPEVH------------------------------------------
Tigriopus_californicus               VGMRST-------IGGPDS-----------------------------------------
Anurida_maritima                     LGIGLR-------RGSSGE-----------------------------------------
Meinertellus_cundinamarcensis        LGLGIG-------RAQDGD-----------------------------------------
Pogonognathellus_sp                  ------------------------------------------------------------
Triops_cancriformis                  ------------------------------------------------------------
Strigamia_maritima2                  LGLGLT-------RPKDIA-----------------------------------------
Catajapyx_aquilonaris                ------------------------------------------------------------
Daphnia_pulex                        SILLLR-------STHLF------------------------------------------
Daphnia_magna3                       LGLRWL-------RGQDKD-----------------------------------------
Daphnia_magna2                       LGLRWL-------RGQDKD-----------------------------------------
Daphnia_magna1                       LGLRWL-------RGQDKD-----------------------------------------
Daphnia_magna4                       LGLRWL-------RGQDKD-----------------------------------------
Arachnocampa_luminosa                KTKV--------------------------------------------------------
Calanus_finmarchicus                 NENTTA-LQLSQMNEVFSPS----------------------------------------
Epiophlebia_superstes                LSARVR----YLLRPSFRPD----------------------------------------
Ladona_fulva                         LPGRVR----DAVRSLFRFN----------------------------------------
Cordulegaster_boltonii               LPGRVR----DLLRPIFHPD----------------------------------------
Diaphorina_citri                     CSNLRF------------SD----------------------------------------
Pachypsylla_venusta                  CFNINV------------KE----------------------------------------
Acanthocasuarina_muellerianae        CVNLVL------------SE----------------------------------------
Glomeris_pustulata                   ICCRKC------------RN----------------------------------------
Frankliniella_cephalica              V----S------------CQ----------------------------------------
Machilis_hrabei                      LKKLMC------------CG----------------------------------------
Carabus_granulatus                   Y---AA------------TS----------------------------------------
Dendroctonus_frontalis               F---LA------------SN----------------------------------------
Lepeophtheirus_salmonis              ------------------------------------------------------------
Caligus_rogercresseyi                ------------------------------------------------------------
Parhyale_hawaiensis                  ------------------------------------------------------------
Hyalella_azteca                      ------------------------------------------------------------
Strigamia_maritima1                  LCCSKE----NEMKSHRTL-----------------------------------------
Conwentzia_psociformis               ------------------------------------------------------------
Liposcelis_bostrychophila            ------------------------------------------------------------
Tetranychus_urticae                  FCYCST----NTNINAGN------------------------------------------
Osmylus_fulvicephalus                ------------------------------------------------------------
Ornithodoros_turicata                CCLAKV----SGRGCSYLA-----------------------------------------
Acerentomon_sp                       ------------------------------------------------------------
Ixodes_ricinus                       NCSIML----R-------------------------------------------------
Folsomia_candida                     ------------------------------------------------------------
Limulus_polyphemus1                  CCHERS----QGYSPQVPV-----------------------------------------
Limulus_polyphemus3                  CCRSVL----QDYMAQSTE-----------------------------------------
Platynothrus_peltifer5               ------------------------------------------------------------
Platynothrus_peltifer1               ------------------------------------------------------------
Platynothrus_peltifer3               ------------------------------------------------------------
```

```
Achipteria_coleoptrata2          ------------------------------------------------------------
Platynothrus_peltifer2           ------------------------------------------------------------
Platynothrus_peltifer4           ------------------------------------------------------------
Ixodes_scapularis2               ICRCPDAKNVSSGIAGTGSCPSAVI-----------------------------------
Ixodes_scapularis1               ------------------------------------------------------------
Ixodes_scapularis3               ------------------------------------------------------------
Cotesia_vestalis                 ------------------------------------------------------------
Microplitis_demolitor            LKKLLC-KQNVINYDI--------------------------------------------
Zorotypus_caudelli               ------------------------------------------------------------
Nasonia_giraulti                 LRSLIK-RDRTSRFGK--------------------------------------------
Nasonia_vitripennis              LRSLIK-RDRTSRFER--------------------------------------------
Varroa_destructor2               ------------------------------------------------------------
Leuctra_sp                       ------------------------------------------------------------
Tricholepidion_gertschi          L-----------------------------------------------------------
Perla_marginata                  ------------------------------------------------------------
Crioscolia_alcione               FFTSNDEYSLTYD-----------------------------------------------
Mischocyttarus-_flavitarsis      FYNRNDEYSQTYYGQR--------------------------------------------
Polistes_metricus                FCNHNE-HSQKYYGQR--------------------------------------------
Polistes_canadensis              FCNRNE-HSQKYYGQR--------------------------------------------
Chrysis_viridula                 LCCRSD-YSTTYD-----------------------------------------------
Argochrysis_armilla              LWCRNN-YSPTYD-----------------------------------------------
Chyphotes_mellipes               LCRRQD-YSLTYG-----------------------------------------------
Brachycistis_timberlakei         FFRRED-YSLAYD-----------------------------------------------
Sphaeropthalma_orestes           FCNSHD-YLPTYD-----------------------------------------------
Homalodisca_vitripennis          SICTGS-PRHRNLRGS--------------------------------------------
Cercopis_vulnerata               ------------------------------------------------------------
Nilaparvata_lugens               LGCKYA-LS-SDYRGN--------------------------------------------
Achipteria_coleoptrata1          ------------------------------------------------------------
Ephemera_danica                  GRV---------------------------------------------------------
Leptopilina_clavipes             ------------------------------------------------------------
Ganaspis_sp                      LRKITR-HKYSPAS----------------------------------------------
Leptopilina_boulardi             LRKITR-HKYSPTS----------------------------------------------
Mantis_religiosa                 ------------------------------------------------------------
Blattella_germanica              ------------------------------------------------------------
Zootermopsis_nevadensis          ------------------------------------------------------------
Stigmatomma_oregonense           FYNKRD-YSLTHGNC---------------------------------------------
Dinoponera_quadriceps            FFKRKD-YSPAY------------------------------------------------
Harpegnathos_saltator            FCKRKD-YSLAY------------------------------------------------
Cerapachys_biroi                 FCRKKD-YSLTY------------------------------------------------
Monomorium_pharaonis             LCGKKD-YSLTYGNHNVSL-----------------------------------------
Tetramorium_bicarinatum          FCRKKD-YSLTY------------------------------------------------
Solenopsis_invicta               FYRKKD-YSLTY------------------------------------------------
Atta_cephalotes                  FYRKKD-YSLTY------------------------------------------------
Acromyrmex_echinatior            FCRKKD-YSLTYGNHNLSLFSLSLSLSLYIYIYISSSHVCICIIPSNIWITRNINVIFIC
Pogonomyrmex_barbatus            FCRKKD-YSLTY------------------------------------------------
Wasmannia_auropunctata           FCRKKD-YSLTY------------------------------------------------
Vollenhovia_emeryi               FCSKKD-YSLTY------------------------------------------------
Orussus_abietinus                LQAPTR-SK---QEADRKP-----------------------------------------
Blaberus_atropos                 LFCRTQ------------------------------------------------------
Limulus_polyphemus2              CCQTSS-KSFSSQVPE--------------------------------------------
Isonychia_bicolor                ------------------------------------------------------------
Occasjapyx_japonicus             ------------------------------------------------------------
Baetis_sp                        ------------------------------------------------------------
Panonychus_ulmi                  ------------------------------------------------------------
Frankliniella_occidentalis       ------------------------------------------------------------
Eurylophella_sp                  ------------------------------------------------------------
Telenomus_podisi                 FGRLFC-YNYAYSIDR--------------------------------------------
Aposthonia_japonica              GASDLH-----YRGS---------------------------------------------
Haploembia_palaui                ------------------------------------------------------------
Halyomorpha_halys                IICRKN-KDRPENVG---SG----------------------------------------
Stenobothrus_lineatus            ------------------------------------------------------------
Inocellia_crassicornis           FVNNLD-SSKT-KYTR--GG----------------------------------------
Tenthredo_koehleri               LYPTRG-SSIKSQEN-----P----------------RQSI-------------------
Corydalus_cornutus               ------------------------------------------------------------
Chrysopa_pallens                 ICSKTY-KNKNYSTA-------------------------I-------------------
Meloe_violaceus                  CT---A-SSKNYKSA------------------------N--------------------
Aethina_tumida                   YT---A-TNKKLKSS------------------------SM-------------------
Gyrinus_marinus                  ------------------------------------------------------------
Tribolium_castaneum              YT---A-SSKNYRSA------------------------T--------------------
Nicrophorus_vespilloides         YT---S-N-RNYREA---------------------------------------------
Aleochara_curtula                ------------------------------------------------------------
Pogonus_chalceus                 YAATS----KNYRSA--GTGN---------------------------------------
Sipyloidea_sipylus               ------------------------------------------------------------
Extatosoma_tiaratum              GNPRA----NADLIG---------------------------------------------
Aretaon_asperrimus               ASAGA----HADLVS---------------------------------------------
Medauroidea_extradentata         GDPRT----NADLFG---------------------------------------------
Ramulus_artemis                  GDPRT----NADLFG---------------------------------------------
```

```
Galloisiana_yuasai                 ------------------------------------------------------------
Athalia_rosae                      IYSSG----RSASYARTREGR----------------RRCV-------------------


                                   721                                                      780
Varroa_destructor1                 ----------------------------------VYRSKNA-ITIS--------------
Metaseiulus_occidentalis1          ----------------------------------GEESRKPCTRAN--------------
Metaseiulus_occidentalis2          ----------------LI----------------TV------------------------
Tigriopus_californicus             ----------------LG----------------GADADRPEPQT---------HHRTKR
Anurida_maritima                   ----------------SI----------------GAEQDIILRTKKFTKPRPDTRPHQDK
Meinertellus_cundinamarcensis      ----------------SI----------------GDEPEG-KTPPP-----IQKQTNQDG
Pogonognathellus_sp                ------------------------------------------------------------
Triops_cancriformis                ------------------------------------------------------------
Strigamia_maritima2                ----------------SI----------------GDEEPQPQQQP------TNKLPSTKS
Catajapyx_aquilonaris              ------------------------------------------------------------
Daphnia_pulex                      -----------------I----------------EQ-----------------------L
Daphnia_magna3                     ----------------SI----------------GEDDRRGAAGP------AGGQPADTN
Daphnia_magna2                     ----------------SI----------------GEDDRRGAAGP------AGGQPADTN
Daphnia_magna1                     ----------------SI----------------GEDDRRGAAGP------AGGQPADTN
Daphnia_magna4                     ----------------SI----------------GEDDRRGAAGP------AGGQPADTN
Arachnocampa_luminosa              ------------------------------------------------------------
Calanus_finmarchicus               ----SPTPTR------LRR---NLSKNSVLRES-----SFIEVV----------------
Epiophlebia_superstes              ----TTKRVRHQEASMSAS---SSSRG----------SSQPKIRANSEEGRN-------G
Ladona_fulva                       ----TSKRLRHNDAITYAS---SSSRTR---SS--RESPQMEIKTLNSPVKDPPS-----
Cordulegaster_boltonii             ----TNKRARHHDATASAS---LSSRTR---SS--RESPQPEVKATAESSKDEER---DG
Diaphorina_citri                   ------DTELT-MDTASAS---G--S--------------------KECGRRTESDLTF-
Pachypsylla_venusta                ------ENRTV--DTASGN---SSSA--------------------RECCRRTDSNVTMG
Acanthocasuarina_muellerianae      ------EAKVA-MDTASGM---SSS-----------------------RERRNESEMTLG
Glomeris_pustulata                 ------SSATNEHGAPIVG---VFSRSSL------SDTNQRSTHVHTLAHGQG-------
Frankliniella_cephalica            ------------TGHPSGS---VLSA-----HSGGSGSSQSNTTQHRTCFTD--------
Machilis_hrabei                    ------GDYLHNSGIPSGG---GFDRGTLFRSSSTSDSKAPSANQNKIAVTRTSVSSQSG
Carabus_granulatus                 ------KNYR---SAGTGN---SASN-----SSGGV--------ESTS------------
Dendroctonus_frontalis             ------KSYR---GAPDGQ---TLSN-----SSGEQ--------VTSLKLTRAS------
Lepeophtheirus_salmonis            ------------------------------------------------------------
Caligus_rogercresseyi              ------------------------------------------------------------
Parhyale_hawaiensis                ------------------------------------------------------------
Hyalella_azteca                    ------------------------------------------------------------
Strigamia_maritima1                ------------ATTYSRT---SFSE------S-----VGPRVALQSLPPSSN-------
Conwentzia_psociformis             ------------------------------------------------------------
Liposcelis_bostrychophila          ------------------------------------------------------------
Tetranychus_urticae                ---------------RSKT---LDDS------S-----VNI-------------------
Osmylus_fulvicephalus              ------------------------------------------------------------
Ornithodoros_turicata              ------------RDTTDGT---NDSN------A-----HSV-------------------
Acerentomon_sp                     ------------------------------------------------------------
Ixodes_ricinus                     ------------------------------------------------------------
Folsomia_candida                   ------------------------------------------------------------
Limulus_polyphemus1                ------------ENI-NNT---TSRS------S-----QTD-------------------
Limulus_polyphemus3                ------------ATSNNNN---ANRS------Y-----PTE-------------------
Platynothrus_peltifer5             ------------------------------------------------------------
Platynothrus_peltifer1             ------------------------------------------------------------
Platynothrus_peltifer3             ------------------------------------------------------------
Achipteria_coleoptrata2            ------------------------------------------------------------
Platynothrus_peltifer2             ------------------------------------------------------------
Platynothrus_peltifer4             ------------------------------------------------------------
Ixodes_scapularis2                 --LADLTT-QGT-EAQSVL----SYKSSPTDTSARLATAV-----------VGKR-----
Ixodes_scapularis1                 ------------------------------------------------------------
Ixodes_scapularis3                 ------------------------------------------------------------
Cotesia_vestalis                   ------------------------------------------------------------
Microplitis_demolitor              ------D----TSYVNSNGS--STKSSIIVTMF-RFAGSMYH-----R-QT-YQERS---
Zorotypus_caudelli                 ------------------------------------------------------------
Nasonia_giraulti                   ------Y----QFYFLCQY-----------------------------------------
Nasonia_vitripennis                ------A----SSNANSNETRSSKRSSFISRMS-RYTSFIIY-----G-PMRTIEIV---
Varroa_destructor2                 ------------------------------------------------------------
Leuctra_sp                         ------------------------------------------------------------
Tricholepidion_gertschi            ------------------------------------------------------------
Perla_marginata                    ------------------------------------------------------------
Crioscolia_alcione                 -----QSMRRSDSFETSTR------STLFSRIS-RLTGSA----MFR-------------
Mischocyttarus-_flavitarsis        -----GMMRRSDSNDTSTR------ASLMSRIS-RYASSI----IPR-------------
Polistes_metricus                  ------MMRRSDSNDTSTR------TSLMSRIS-RYTSSI----IPR-------------
Polistes_canadensis                ------MMRRSDSNDTSTR------ISLMSRIS-RYTSSI----IRR-------------
Chrysis_viridula                   -----QELHPANSTETSTR------SSFISRIF-WYAGSM----IFR-------------
Argochrysis_armilla                -----QELHPANSNETSTR------SSLISRIS-RYAGSM----IFR-------------
Chyphotes_mellipes                 -----RNTHRSNSHENSTR------SSLISRIS-RYAGTA----IFR-------------
Brachycistis_timberlakei           -----RDMQRSDWNEASTR------SSLISRIS-RYANSV----ISH-------------
Sphaeropthalma_orestes             -----RDVRRGNSDETSTR------SSLISRIS-RYAGSV----IFR-------------
Homalodisca_vitripennis            -------A-------SSCR-----------------------------------------
Cercopis_vulnerata                 ------------------------------------------------------------
```

```
Nilaparvata_lugens                  -------NSAAESGSSGCGAGSCSKRSEVTNTTSL---------LPRK-PLVAEQ-----
Achipteria_coleoptrata1             ------------------------------------------------------------
Ephemera_danica                     ------------------------------------------------------------
Leptopilina_clavipes                ------------------------------------------------------------
Ganaspis_sp                         ----DEASKNDHSNEPSTTS------SLISKVSV-Y--PN-V--R----PNYSEA-----
Leptopilina_boulardi                ----DDNSKIGHSNEPSTTS------SLISKVST-C--PTTV--K----PIYTEV-----
Mantis_religiosa                    ------------------------------------------------------------
Blattella_germanica                 ------------------------------------------------------------
Zootermopsis_nevadensis             ------------------------------------------------------------
Stigmatomma_oregonense              ------------------------------------------------------------
Dinoponera_quadriceps               ----DAVAQQNASDAASTT------SSFISRIS-RL------------------------
Harpegnathos_saltator               ----DVIARQNTSDAPSIT------SSFISRIS-RL------------------------
Cerapachys_biroi                    ----DIDAHQNASDVPSTT------SSFISRIS-RL------------------------
Monomorium_pharaonis                ------------------------------------------------------------
Tetramorium_bicarinatum             ----DIDARQNASDVPSST------SSFISRIS-RL------------------------
Solenopsis_invicta                  ----DIDARQNASDVPSST------SSFISRIS-RL------------------------
Atta_cephalotes                     ----DIDARQNASDVPSST------SSFISRIS-RL------------------------
Acromyrmex_echinatior               HFLSDIDARQNTSDVPSST------SSFISRIS-RL------------------------
Pogonomyrmex_barbatus               ----DIDARQNALDVPSST------SPFISRIS-RL------------------------
Wasmannia_auropunctata              ----DIDVRQNASDVPSTT------SSFISRIS-RL------------------------
Vollenhovia_emeryi                  ----DIDVRQNASDVPSST------SSFISRIS-RL------------------------
Orussus_abietinus                   ------------------------------------------------------------
Blaberus_atropos                    ------------------------------------------------------------
Limulus_polyphemus2                 ----------NTDNNTFSRSERCSSYPLSVRTGSRHKFPDGSTTRTRK-RLCRELGSK--
Isonychia_bicolor                   ------------------------------------------------------------
Occasjapyx_japonicus                ------------------------------------------------------------
Baetis_sp                           ------------------------------------------------------------
Panonychus_ulmi                     ------------------------------------------------------------
Frankliniella_occidentalis          ------------------------------------------------------------
Eurylophella_sp                     ------------------------------------------------------------
Telenomus_podisi                    ------STRSGSSNENSTR------SSFISRIS-RYAS---------F-VMHGPA-----
Aposthonia_japonica                 ----------SSSKERSRLSKLVSWRST--------------------------------
Haploembia_palaui                   ------------------------------------------------------------
Halyomorpha_halys                   ----------GSSGRPHQS---CTLAGQHTH-----------------------------
Stenobothrus_lineatus               ------------------------------------------------------------
Inocellia_crassicornis              -----MSKASNSSSTVETAVGRLSWLSKSFRSS-KASF----------------------
Tenthredo_koehleri                  ----EI-IRWGSSASSSGT----SIRSYASRMS-RYAN---------S-VLRRSSDSS--
Corydalus_cornutus                  ------------------------------------------------------------
Chrysopa_pallens                    ----TNSGGSNSSQGVEST----TVRVPL-----RISF-------------YTRNDGG--
Meloe_violaceus                     ----AGNSGSNSSGEPQST----SLRPFS-----KWSF-------------CNSPKSN--
Aethina_tumida                      ----GNNSGSNSSGEIQST----SLRTLS-----RLSR-------------NNSGRL---
Gyrinus_marinus                     ------------------------------------------------------------
Tribolium_castaneum                 ----GGNSASNSSGDAQST----SLRPFS-----RWSL-------------CNSARS---
Nicrophorus_vespilloides            ----AGNSTSNSSGGVEST----SLRPFT-----RWSF-------------CNGNRA---
Aleochara_curtula                   ------------------------------------------------------------
Pogonus_chalceus                    -------SASNSSGGVEST----SLRPFS-----KWSL-------------CASAKSS--
Sipyloidea_sipylus                  ------------------------------------------------------------
Extatosoma_tiaratum                 -----------GSGS-STT----SWRNSK-----VYRL-------------V--------
Aretaon_asperrimus                  -----------GSGS-SST----SWRNSR-----TYRL-------------V--------
Medauroidea_extradentata            -----------GSGS-STT----SWRNSK-----VYRL-------------V--------
Ramulus_artemis                     -----------GSGS-STT----SWRNSK-----VYRL-------------V--------
Galloisiana_yuasai                  ------------------------------------------------------------
Athalia_rosae                       ----STETPCNSSGS-STG----TSVESY-------------------------------

                                    781                                                      840
Varroa_destructor1                  ILQYRPPNRSKRQPRAYRGA----------------------------------------
Metaseiulus_occidentalis1           AIVCE---KPARQLRGYRIS----------------------------------------
Metaseiulus_occidentalis2           ------------------------------------------------------------
Tigriopus_californicus              ILFTRKAEPLKGNPLGARPHPLFALQDQE-IVRA---GIRSEPNLS-SHHR---PNV--K
Anurida_maritima                    SR---------------------TMKQVS-TLDA---IENQGAFQLQLETK---SEV--L
Meinertellus_cundinamarcensis       G--GGGTK--SDL---V-------------------------------------------
Pogonognathellus_sp                 ------------------------------------------------------------
Triops_cancriformis                 ------------------------------------------------------------
Strigamia_maritima2                 EMV---------------------------------------------------------
Catajapyx_aquilonaris               ------------------------------------------------------------
Daphnia_pulex                       LFFIRVSRK--SRN---------I------------------------------------
Daphnia_magna3                      HRFGRGPRR--AQT---RDQHRQVMAEQQ-LLPA---GEQHPAMLATKCTR---ADI--V
Daphnia_magna2                      HRFGRGPRR--AQT---RDQHRQVMAEQQ-LLPA---GEQHPAMLATKCTR---ADI--V
Daphnia_magna1                      HRFGRGPRR--AQT---RDQHRQVMAEQQ-LLPA---GEQHPAMLATKCTR---ADI--V
Daphnia_magna4                      HRFGRGPRR--AQT---RDQHRQVMAEQQ-LLPA---GEQHPAMLATKCTR---ADI--V
Arachnocampa_luminosa               ------------------------------------------------------------
Calanus_finmarchicus                ----GL-------------------QE---------------------------------
Epiophlebia_superstes               GR----------------------------------------------------------
Ladona_fulva                        VKKNGVVAEPKTEK----------------------------------------------
Cordulegaster_boltonii              CRMIGLADRTKEEK---------AVKESAVLLSN-----------A--------------
Diaphorina_citri                    --LNRTTYSNPDEN---------GALIPLIILTF-----------AYTCIC---RAI--I
```

```
Pachypsylla_venusta                TYLNRQSCNDD--T---------YVSEDNLI-----------------------------
Acanthocasuarina_muellerianae      TFLNRHEDT--------------GM-SDDSL-----------------------------
Glomeris_pustulata                 ------------------------------------------------------------
Frankliniella_cephalica            ------------------------------------------------------------
Machilis_hrabei                    VLLKRRTLNGDKQP---------T------------------------------------
Carabus_granulatus                 ------------------------------------------------------------
Dendroctonus_frontalis             ---------PGRDR---------H------------------------------------
Lepeophtheirus_salmonis            ------------------------------------------------------------
Caligus_rogercresseyi              ------------------------------------------------------------
Parhyale_hawaiensis                ------------------------------------------------------------
Hyalella_azteca                    ------------------------------------------------------------
Strigamia_maritima1                N-FLRVNDNK---L---------NVRVAAVSDSA-----------LYSCEK--------R
Conwentzia_psociformis             ------------------------------------------------------------
Liposcelis_bostrychophila          ------------------------------------------------------------
Tetranychus_urticae                -------------------------EPHCSS-----------------------IN---R
Osmylus_fulvicephalus              ------------------------------------------------------------
Ornithodoros_turicata              -------------------------VSHCEGDHA-----------RIKCSP---SKR--R
Acerentomon_sp                     ------------------------------------------------------------
Ixodes_ricinus                     ------------------------------------------------------------
Folsomia_candida                   ------------------------------------------------------------
Limulus_polyphemus1                -------------------------NEICTSYPL-----------SLRCGV---HY----
Limulus_polyphemus3                -------------------------NEVNSSYPL-----------SFFIAQ---EE----
Platynothrus_peltifer5             ------------------------------------------------------------
Platynothrus_peltifer1             ------------------------------------------------------------
Platynothrus_peltifer3             ------------------------------------------------------------
Achipteria_coleoptrata2            ------------------------------------------------------------
Platynothrus_peltifer2             ------------------------------------------------------------
Platynothrus_peltifer4             ------------------------------------------------------------
Ixodes_scapularis2                 ----GATDVEEDL---------------------------------------------QC
Ixodes_scapularis1                 ------------------------------------------------------------
Ixodes_scapularis3                 ------------------------------------------------------------
Cotesia_vestalis                   ------------------------------------------------------------
Microplitis_demolitor              -----------------------------------------TDFILT------------R
Zorotypus_caudelli                 ------------------------------------------------------------
Nasonia_giraulti                   ------------------------------------------------------------
Nasonia_vitripennis                -----------------------------------------NKLYI--------------
Varroa_destructor2                 ------------------------------------------------------------
Leuctra_sp                         ------------------------------------------------------------
Tricholepidion_gertschi            ------------------------------------------------------------
Perla_marginata                    ------------------------------------------------------------
Crioscolia_alcione                 ------------------------------------------------------------
Mischocyttarus-_flavitarsis        ------------------------------------------------------------
Polistes_metricus                  ------------------------------------------------------------
Polistes_canadensis                ------------------------------------------------------------
Chrysis_viridula                   ------------------------------------------------------------
Argochrysis_armilla                ------------------------------------------------------------
Chyphotes_mellipes                 ------------------------------------------------------------
Brachycistis_timberlakei           ------------------------------------------------------------
Sphaeropthalma_orestes             ------------------------------------------------------------
Homalodisca_vitripennis            ------------------------------------------------------------
Cercopis_vulnerata                 ------------------------------------------------------------
Nilaparvata_lugens                 -------------------PLPEVTPIRRWVVTIPPNVSREERECLQLFSGLNNERILPR
Achipteria_coleoptrata1            ------------------------------------------------------------
Ephemera_danica                    ------------------------------------------------------------
Leptopilina_clavipes               ------------------------------------------------------------
Ganaspis_sp                        -------------------------RERNIS-----------------------------
Leptopilina_boulardi               -------------------------RERNIS-----------------------------
Mantis_religiosa                   ------------------------------------------------------------
Blattella_germanica                ------------------------------------------------------------
Zootermopsis_nevadensis            ------------------------------------------------------------
Stigmatomma_oregonense             ------------------------------------------------------------
Dinoponera_quadriceps              ------------------------------------------------------------
Harpegnathos_saltator              ------------------------------------------------------------
Cerapachys_biroi                   ------------------------------------------------------------
Monomorium_pharaonis               ------------------------------------------------------------
Tetramorium_bicarinatum            ------------------------------------------------------------
Solenopsis_invicta                 ------------------------------------------------------------
Atta_cephalotes                    ------------------------------------------------------------
Acromyrmex_echinatior              ------------------------------------------------------------
Pogonomyrmex_barbatus              ------------------------------------------------------------
Wasmannia_auropunctata             ------------------------------------------------------------
Vollenhovia_emeryi                 ------------------------------------------------------------
Orussus_abietinus                  ------------------------------------------------------------
Blaberus_atropos                   ------------------------------------------------------------
Limulus_polyphemus2                ---NESTDVGE--NRGSVTQTETT--------------S---------------------
Isonychia_bicolor                  ------------------------------------------------------------
Occasjapyx_japonicus               ------------------------------------------------------------
```

```
Baetis_sp                          ------------------------------------------------------------
Panonychus_ulmi                    ------------------------------------------------------------
Frankliniella_occidentalis         ------------------------------------------------------------
Eurylophella_sp                    ------------------------------------------------------------
Telenomus_podisi                   ------------------TK----ERISR-------------------------------
Aposthonia_japonica                ------------------------------------------------------------
Haploembia_palaui                  ------------------------------------------------------------
Halyomorpha_halys                  --------------------------YAKWVVALPPNLSESDKTMLRL----------FR
Stenobothrus_lineatus              ------------------------------------------------------------
Inocellia_crassicornis             ----QHVHN--NI-----IQS---------------------------------------
Tenthredo_koehleri                 ---SNKE-----------LQPSNRIR----------------------------------
Corydalus_cornutus                 ------------------------------------------------------------
Chrysopa_pallens                   ---ANG------------KQ--------------------------------------TY
Meloe_violaceus                    ---GNK------------YT--------------------------------------SR
Aethina_tumida                     ----NR------------CS--------------------------------------VR
Gyrinus_marinus                    ------------------------------------------------------------
Tribolium_castaneum                ----NK------------YP--------------------------------------TR
Nicrophorus_vespilloides           ---AAK------------FN--------------------------------------PR
Aleochara_curtula                  ------------------------------------------------------------
Pogonus_chalceus                   ---SAINHNGNNNTNGHAIGSGD----FRARYPAPPASSAASTLIRLPHAPAHSPQQLLM
Sipyloidea_sipylus                 ------------------------------------------------------------
Extatosoma_tiaratum                --------------SGSVFKQRDAVRERRSAPLRTIYGSLDLELL---------------
Aretaon_asperrimus                 --------------SGSMFKSRDSVRERRWAMAMPTKIPIATALMAGRDLPSTSEEV-AS
Medauroidea_extradentata           --------------NGSAFKQRDAVRERRWAMAMPTKIPIATALMAGRDAPSTSQDV-A-
Ramulus_artemis                    --------------SGSAFKQRDSVRERRWAMAMPTKIPIATALMVGRDIPSTSQDA-A-
Galloisiana_yuasai                 ------------------------------------------------------------
Athalia_rosae                      -----------------------ASRMSRYAYAI-----------LGKSDTANSKEF-HC

                                   841                                       885
Varroa_destructor1                 ---------------------------------------------
Metaseiulus_occidentalis1          ---------------------------------------------
Metaseiulus_occidentalis2          ---------------------------------------------
Tigriopus_californicus             NPLPA------------------S-----NQNK---SWT-KT---
Anurida_maritima                   ---------------------------------------------
Meinertellus_cundinamarcensis      ---------------------------------------------
Pogonognathellus_sp                ---------------------------------------------
Triops_cancriformis                ---------------------------------------------
Strigamia_maritima2                ---------------------------------------------
Catajapyx_aquilonaris              ---------------------------------------------
Daphnia_pulex                      ---------------------------------------------
Daphnia_magna3                     THLGN------------------TKKLQFQQNH---QHVEKIG--
Daphnia_magna2                     THLGN------------------TKKLQFQQNH---QHVEKIG--
Daphnia_magna1                     THLGN------------------TKKLQFQQNH---QHVEKIG--
Daphnia_magna4                     THLGN------------------TKKLQFQQNH---QHVEKIG--
Arachnocampa_luminosa              ---------------------------------------------
Calanus_finmarchicus               ---------------------------------------------
Epiophlebia_superstes              ---------------------------------------------
Ladona_fulva                       ---------------------------------------------
Cordulegaster_boltonii             ----------------------PV---------------------
Diaphorina_citri                   N--FSMKNSIRRHQFSRSSRTSRDS---ETENELCHTIVDSDG--
Pachypsylla_venusta                --------VSRPRQWVVSVPDG-----------------------
Acanthocasuarina_muellerianae      --------TLRSRQWVVTLPDANQT---R----------------
Glomeris_pustulata                 ---------------------------------------------
Frankliniella_cephalica            ---------------------------------------------
Machilis_hrabei                    --------AVEMRNWKCDETNRPLPLLAETSDFIQCAIGENE---
Carabus_granulatus                 --------LRPFSKWSLCASTKSTPRY------------------
Dendroctonus_frontalis             --------LTNGRQWMVTAT-------------------------
Lepeophtheirus_salmonis            ---------------------------------------------
Caligus_rogercresseyi              ---------------------------------------------
Parhyale_hawaiensis                ---------------------------------------------
Hyalella_azteca                    ---------------------------------------------
Strigamia_maritima1                HSSPNLNVKSAASTLVLCSQ----QHHHDTQNHLKDGVTMETQCA
Conwentzia_psociformis             ---------------------------------------------
Liposcelis_bostrychophila          ---------------------------------------------
Tetranychus_urticae                NGSRTIFVSQKMSKYSDDKPSVEAHG-------------------
Osmylus_fulvicephalus              ---------------------------------------------
Ornithodoros_turicata              NGFRS-STDVEMRTAQDCGADT-----------------------
Acerentomon_sp                     ---------------------------------------------
Ixodes_ricinus                     ---------------------------------------------
Folsomia_candida                   ---------------------------------------------
Limulus_polyphemus1                ---TS-SFRLKKTKITGCRPCPNIQDLHDEAETFSA---------
Limulus_polyphemus3                ---PV-MLRNRLKKKQ-----------------------------
Platynothrus_peltifer5             ---------------------------------------------
Platynothrus_peltifer1             ---------------------------------------------
Platynothrus_peltifer3             ---------------------------------------------
Achipteria_coleoptrata2            ---------------------------------------------
Platynothrus_peltifer2             ---------------------------------------------
```

```
Platynothrus_peltifer4             ---------------------------------------------
Ixodes_scapularis2                 S-------------------------LEKTS--------------
Ixodes_scapularis1                 ---------------------------------------------
Ixodes_scapularis3                 ---------------------------------------------
Cotesia_vestalis                   ---------------------------------------------
Microplitis_demolitor              RQS------------------------------------------
Zorotypus_caudelli                 ---------------------------------------------
Nasonia_giraulti                   ---------------------------------------------
Nasonia_vitripennis                ---------------------------------------------
Varroa_destructor2                 ---------------------------------------------
Leuctra_sp                         ---------------------------------------------
Tricholepidion_gertschi            ---------------------------------------------
Perla_marginata                    ---------------------------------------------
Crioscolia_alcione                 ---------------------------------------------
Mischocyttarus-_flavitarsis        ---------------------------------------------
Polistes_metricus                  ---------------------------------------------
Polistes_canadensis                ---------------------------------------------
Chrysis_viridula                   ---------------------------------------------
Argochrysis_armilla                ---------------------------------------------
Chyphotes_mellipes                 ---------------------------------------------
Brachycistis_timberlakei           ---------------------------------------------
Sphaeropthalma_orestes             ---------------------------------------------
Homalodisca_vitripennis            ---------------------------------------------
Cercopis_vulnerata                 ---------------------------------------------
Nilaparvata_lugens                 SQQQQ----HNQQRAIR-TSSSPCR-AARCRLDL-----------
Achipteria_coleoptrata1            ---------------------------------------------
Ephemera_danica                    ---------------------------------------------
Leptopilina_clavipes               ---------------------------------------------
Ganaspis_sp                        ---------------------------------------------
Leptopilina_boulardi               ---------------------------------------------
Mantis_religiosa                   ---------------------------------------------
Blattella_germanica                ---------------------------------------------
Zootermopsis_nevadensis            ---------------------------------------------
Stigmatomma_oregonense             ---------------------------------------------
Dinoponera_quadriceps              -ASSKIFG-------------------------------------
Harpegnathos_saltator              -ASSKIFG-------------------------------------
Cerapachys_biroi                   -ASSKIFG-------------------------------------
Monomorium_pharaonis               ---------------------------------------------
Tetramorium_bicarinatum            -ASSKIFG-------------------------------------
Solenopsis_invicta                 -ASSKIFG-------------------------------------
Atta_cephalotes                    -ASSKIFG-------------------------------------
Acromyrmex_echinatior              -ASSKIFG-------------------------------------
Pogonomyrmex_barbatus              -ISSKIFE-------------------------------------
Wasmannia_auropunctata             -ASSKIFG-------------------------------------
Vollenhovia_emeryi                 -ASSKIFG-------------------------------------
Orussus_abietinus                  ---------------------------------------------
Blaberus_atropos                   ---------------------------------------------
Limulus_polyphemus2                -EPGKFVTRDKDKTLC-----------------------------
Isonychia_bicolor                  ---------------------------------------------
Occasjapyx_japonicus               ---------------------------------------------
Baetis_sp                          ---------------------------------------------
Panonychus_ulmi                    ---------------------------------------------
Frankliniella_occidentalis         ---------------------------------------------
Eurylophella_sp                    ---------------------------------------------
Telenomus_podisi                   ---------------------------------------------
Aposthonia_japonica                ---------------------------------------------
Haploembia_palaui                  ---------------------------------------------
Halyomorpha_halys                  TPNRNVWSS-LEAKYETTDSKLSPE-L------------------
Stenobothrus_lineatus              ---------------------------------------------
Inocellia_crassicornis             ---------------------------------------------
Tenthredo_koehleri                 ---------------------------------------------
Corydalus_cornutus                 ---------------------------------------------
Chrysopa_pallens                   TTNYHPSVRYTPKKWTVAIEELPP---------------------
Meloe_violaceus                    LTQRPYDAQSNTRRWIVTTTT------------------------
Aethina_tumida                     TANQNYITKYDAKDWIVATAT------------------------
Gyrinus_marinus                    ---------------------------------------------
Tribolium_castaneum                VPHRPYVAQYNARRWIVTTTT------------------------
Nicrophorus_vespilloides           QPQRQYVTQYNARRWIVTTT-------------------------
Aleochara_curtula                  ---------------------------------------------
Pogonus_chalceus                   QQPSPFLGRYHARRWIVTTSA------------------------
Sipyloidea_sipylus                 ---------------------------------------------
Extatosoma_tiaratum                ---------------------------------------------
Aretaon_asperrimus                 ---------------------------------------------
Medauroidea_extradentata           ---------------------------------------------
Ramulus_artemis                    ---------------------------------------------
Galloisiana_yuasai                 ---------------------------------------------
Athalia_rosae                      LQSSSLRGIRNELE-------------------------------
```

Supplementary Figure S4. Discrimination between oxytocin/vasopressin-like and crustacean cardioactive peptide receptors. Sequence logo frequency plots and alignments of sequence stretches used to distinguish oxytocin/vasopressin-like from closely related crustacean cardioactive peptide (CCAP) receptors. (A) Sequence logo of the end of transmembrane domain 2 and the beginning of extracellular loop 1 of 68 oxytocin/vasopressin-like receptors (upper panel) and 68 CCAP receptors (lower panel). (B) Sequence logo of transmembrane domain 7 of 68 oxytocin-like receptors (upper panel) and 68 CCAP receptors (lower panel). Individual sequences and of those conserved motifs are shown for oxytocin/vasopressin-like (C) and CCAP receptors (D). Conserved residues are highlighted in green. Major differences of oxytocin/vasorpessin-like to CCAP receptor sequences are shown in red.

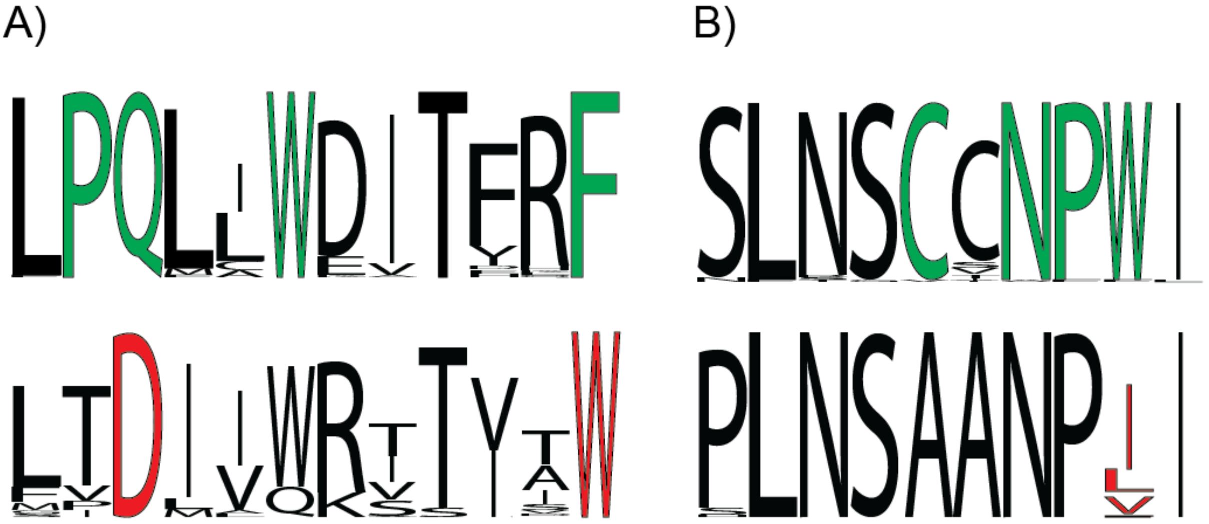

(C)

**Transmembrane domain 2/extracellular loop1 (*X*PQ*X*$_2$W*X*$_{5\text{-}6}$F)**		**Transmembrane domain 7 (C*X*NPW)**	
tr|Q6Q4A4|Q6Q4A4_THETS	LPQLIWDITEKF	tr|Q6Q4A4|Q6Q4A4_THETS	SLNSCVNPFI
tr|B1NWV5|B1NWV5_TRICA	LPQLAWDITYRF	tr|B1NWV5|B1NWV5_TRICA	SLNSCVNPWI
gi|332020179|gb|EGI60623.1|	LPQLAWDITFRF	gi|332020179|gb|EGI60623.1|	SLTSCVNPWI
sp|Q75W84|ANR_EISFO	LPQLLWDVTHRF	sp|Q75W84|ANR_EISFO	SLNSCINPWI
sp|Q7YW31|CTR1_OCTVU	LPQMIWDITFLF	sp|Q7YW31|CTR1_OCTVU	SLNSCTNPWI
tr|K9JBV2|K9JBV2_DAPPU	LPQLAWDITYRF	tr|K9JBV2|K9JBV2_DAPPU	SLNSCSNPWI
sp|P30518|V2R_HUMAN	LPQLAWKATDRF	sp|P30518|V2R_HUMAN	SLNSCTNPWI
tr|B3XZZ4|B3XZZ4_PROAN	LPQLLWDVTDQF	tr|B3XZZ4|B3XZZ4_PROAN	SLNSCSNPWI
tr|Q25397|Q25397_LYMST	LPQLIWDITERF	tr|Q25397|Q25397_LYMST	SLNSCCNPWI
tr|H7CEK0|H7CEK0_TRISC	LPQLIWEITFRF	tr|H7CEK0|H7CEK0_TRISC	SLNSCCNPWI
tr|H3K0J0|H3K0J0_CALMI	LPQLIWEITFRF	tr|H3K0J0|H3K0J0_CALMI	SLNSCCNPWI
tr|H2T2H1|H2T2H1_TAKRU	FPQLIWDITFRF	tr|H2T2H1|H2T2H1_TAKRU	SLNSCCNPWI
tr|D0UYC9|D0UYC9_CYPNE	LPQLIWDITFRF	tr|D0UYC9|D0UYC9_CYPNE	SLNSCCNPWI
tr|H1ADE8|H1ADE8_ORYLA	LPQLIWDITFRF	tr|H1ADE8|H1ADE8_ORYLA	SLNSCCNPWI
tr|H2LDT4|H2LDT4_ORYLA	LPQLIWDITFRF	tr|H2LDT4|H2LDT4_ORYLA	SLNSCCNPWI
tr|E4W698|E4W698_DANRE	LPQLIWDITFRF	tr|E4W698|E4W698_DANRE	SLNSCCNPWI
sp|Q90334|ITR_CATCO	LPQLIWDITFRF	sp|Q90334|ITR_CATCO	SLNSCCNPWI
tr|E4W699|E4W699_DANRE	LPQLIWDITFRF	tr|E4W699|E4W699_DANRE	SLNSCCNPWI
tr|G3N7A8|G3N7A8_GASAC	LPQLIWDITFRF	tr|G3N7A8|G3N7A8_GASAC	SLNSCCNPWI
tr|H7CEB8|H7CEB8_AMPOC	LPQLIWDITFRF	tr|H7CEB8|H7CEB8_AMPOC	SLNSCCNPWI
tr|H1ADE9|H1ADE9_ORYLA	LPQLIWDITFRF	tr|H1ADE9|H1ADE9_ORYLA	SLNSCCNPWI
tr|H3DAW1|H3DAW1_TETNG	LPQLIWDITFRF	tr|H3DAW1|H3DAW1_TETNG	SLNSCCNPWI
tr|H2S576|H2S576_TAKRU	LPQLIWDITFRF	tr|H2S576|H2S576_TAKRU	SLNSCCNPWI
tr|H2S575|H2S575_TAKRU	LPQLIWDITFRF	tr|H2S575|H2S575_TAKRU	SLNSCCNPWI
tr|G3WCZ9|G3WCZ9_SARHA	LPQLLWDITFHF	tr|G3WCZ9|G3WCZ9_SARHA	SLNSCCNPWI
tr|F6U716|F6U716_MONDO	LPQLLWDITFHF	tr|F6U716|F6U716_MONDO	SLNSCCNPWI
tr|G5BX96|G5BX96_HETGA	LPQLLWDITFRF	tr|G5BX96|G5BX96_HETGA	SLNSCCNPWI
sp|Q28756|OXYR_SHEEP	LPQLLWDITFRF	sp|Q28756|OXYR_SHEEP	SLNSCCNPWI
sp|P56449|OXYR_BOVIN	LPQLLWDITFRF	sp|P56449|OXYR_BOVIN	SLNSCCNPWI
tr|C4PD02|C4PD02_BUBBU	LPQLLWDITFRF	tr|C4PD02|C4PD02_BUBBU	SLNSCCNPWI
tr|G1SQ05|G1SQ05_RABIT	LPQLLWDITFRF	tr|G1SQ05|G1SQ05_RABIT	SLNSCCNPWI
tr|H0VZP0|H0VZP0_CAVPO	LPQLLWDITFRF	tr|H0VZP0|H0VZP0_CAVPO	SLNSCCNPWI
tr|G1PUQ9|G1PUQ9_MYOLU	LPQLLWDITFRF	tr|G1PUQ9|G1PUQ9_MYOLU	SLNSCCNPWI
tr|G3TTL6|G3TTL6_LOXAF	LPQLLWDITFRF	tr|G3TTL6|G3TTL6_LOXAF	SLNSCCNPWI

tr|G3UGJ6|G3UGJ6_LOXAF	LPQLLWDITFRF	tr|G3UGJ6|G3UGJ6_LOXAF	SLNSCCNPWI
sp|P32306|OXYR_PIG	LPQLLWDITFRF	sp|P32306|OXYR_PIG	SLNSCCNPWI
tr|E3V2E5|E3V2E5_CANFA	LPQLLWDITFRF	tr|E3V2E5|E3V2E5_CANFA	SLNSCCNPWI
tr|G1M104|G1M104_AILME	LPQLLWDITFRF	tr|G1M104|G1M104_AILME	SLNSCCNPWI
tr|G3H112|G3H112_CRIGR	LPQLLWDITFRF	tr|G3H112|G3H112_CRIGR	SLNSCCNPWI
tr|E0V872|E0V872_MICOH	LPQLLWDITFRF	tr|E0V872|E0V872_MICOH	SLNSCCNPWI
sp|P97926|OXYR_MOUSE	LPQLLWDITFRF	sp|P97926|OXYR_MOUSE	SLNSCCNPWI
sp|P70536|OXYR_RAT	LPQLLWDITFRF	sp|P70536|OXYR_RAT	SLNSCCNPWI
tr|F2YMM4|F2YMM4_SAISC	LPQLLWDITYRF	tr|F2YMM4|F2YMM4_SAISC	SLNSCCNPWI
tr|F7ILY7|F7ILY7_CALJA	LPQLLWDITFRF	tr|F7ILY7|F7ILY7_CALJA	SLNSCCNPWI
tr|H0WII7|H0WII7_OTOGA	LPQLLWDITFRF	tr|H0WII7|H0WII7_OTOGA	SLNSCCNPWI
sp|P56494|OXYR_MACMU	LPQLLWDITFRF	sp|P56494|OXYR_MACMU	SLNSCCNPWI
tr|E3V2E7|E3V2E7_MACFA	LPQLLWDITFRF	tr|E3V2E7|E3V2E7_MACFA	SLNSCCNPWI
sp|P30559|OXYR_HUMAN	LPQLLWDITFRF	sp|P30559|OXYR_HUMAN	SLNSCCNPWI
tr|G1QUE2|G1QUE2_NOMLE	LPQLLWDITFRF	tr|G1QUE2|G1QUE2_NOMLE	SLNSCCNPWI
tr|G3R8I4|G3R8I4_GORGO	LPQLLWDITFRF	tr|G3R8I4|G3R8I4_GORGO	SLNSCCNPWI
tr|Q5D7U5|Q5D7U5_CHICK	LPQLIWDITFRF	tr|Q5D7U5|Q5D7U5_CHICK	SLNSCCNPWI
tr|G3USG7|G3USG7_MELGA	LPQLIWDITFRF	tr|G3USG7|G3USG7_MELGA	SFRGVSAFWL
tr|Q3LRX3|Q3LRX3_TARGR	LPQLIWDITFRF	tr|Q3LRX3|Q3LRX3_TARGR	SLNSCCNPWI
tr|F7C7K9|F7C7K9_XENTR	LPQLIWDITFRF	tr|F7C7K9|F7C7K9_XENTR	SLNSCCNPWI
tr|Q6WNV4|Q6WNV4_LITCT	LPQLIWDITFRF	tr|Q6WNV4|Q6WNV4_LITCT	SLNSCCNPWI
sp|Q90252|MTR_BUFMA	LPQLIWDITFRF	sp|Q90252|MTR_BUFMA	SLNSCCNPWI
tr|Q8AYS5|Q8AYS5_HYLJA	LPQLIWDITFRF	tr|Q8AYS5|Q8AYS5_HYLJA	SLNSCCNPWI
tr|H7CE74|H7CE74_PROAN	LPQLIWDITFRF	tr|H7CE74|H7CE74_PROAN	SLNSCYNPWI
tr|H3BG77|H3BG77_LATCH	LPQLIWDITFRF	tr|H3BG77|H3BG77_LATCH	SLNSCCNPWI
sp|P37288|V1AR_HUMAN	LPQMCWDITYRF	sp|P37288|V1AR_HUMAN	SLNSCCNPWI
tr|A8CWP8|A8CWP8_CHICK	LPQLCWEVTHRF	tr|A8CWP8|A8CWP8_CHICK	SLNSCCNPWI
tr|H3K0J2|H3K0J2_CALMI	LPQFIWDITYRF	tr|H3K0J2|H3K0J2_CALMI	SLNSCCNPWI
sp|Q90352|AVT_CATCO	LPQLCWEITFRF	sp|Q90352|AVT_CATCO	SLNSCCNPWI
tr|Q6WNV5|Q6WNV5_LITCT	LPQLCWEITYRF	tr|Q6WNV5|Q6WNV5_LITCT	SLNSCCNPWI
tr|B3XZZ3|B3XZZ3_PROAN	LPQLCWEVTYRF	tr|B3XZZ3|B3XZZ3_PROAN	SLNSCCNPWI
tr|H7CEB4|H7CEB4_PROAN	LPQMIWEVTYRF	tr|H7CEB4|H7CEB4_PROAN	SLSSCCNPWI
sp|P47901|V1BR_HUMAN	LPQLLWDITYRF	sp|P47901|V1BR_HUMAN	NLNSCCNPWI
tr|H3K0J1|H3K0J1_CALMI	LPQLCWEITYRF	tr|H3K0J1|H3K0J1_CALMI	NLNSCCNPWI

(D)

Transmembrane domain 2/extracellular loop1 (X_2**D**X_8**W**)		Transmembrane domain 7 (**A**XNP**V/L/F**)	
gi|768420480|ref|XP_011551009.1|	LPDLIQRITISW	gi|768420480|ref|XP_011551009.1|	PLNSAANPVI
gi|913307839|ref|XP_013184425.1|	LPDLIQRITITW	gi|913307839|ref|XP_013184425.1|	PLNSAANPVI
gi|909558612|ref|XP_013134323.1|	LPDLIQRITISW	gi|909558612|ref|XP_013134323.1|	PLNSAANPVI
gi|943960983|ref|XP_014363004.1|	LPDLIQRITISW	gi|943960983|ref|XP_014363004.1|	PLNSAANPVI
gi|910347322|ref|XP_013177593.1|	LPDLIQRITISW	gi|910347322|ref|XP_013177593.1|	PLNSAANPVI
gi|913307945|ref|XP_013184483.1|	FVDILQKITIAW	gi|913307945|ref|XP_013184483.1|	PLNSAANPLI
gi|913307943|ref|XP_013184481.1|	FVDILQKITIAW	gi|913307943|ref|XP_013184481.1|	PLNSAANPLI
gi|943960974|ref|XP_014363000.1|	FVDIIQKITITW	gi|943960974|ref|XP_014363000.1|	PLNSAANPLI
gi|910348561|ref|XP_013178264.1|	FVDIIQKITITW	gi|910348561|ref|XP_013178264.1|	PLNSAANPLI
gi|910348559|ref|XP_013178263.1|	FVDIIQKITITW	gi|910348559|ref|XP_013178263.1|	PLNSAANPLI
gi|909558903|ref|XP_013134477.1|	FVDIIQKITIAW	gi|909558903|ref|XP_013134477.1|	PLNSAANPLI
gi|909558901|ref|XP_013134476.1|	FVDIIQKITIAW	gi|909558901|ref|XP_013134476.1|	PLNSAANPLI
gi|909558899|ref|XP_013134475.1|	FVDIIQKITIAW	gi|909558899|ref|XP_013134475.1|	PLNSAANPLI
gi|817089496|ref|XP_012267616.1|	GTDIAWRSTVDW	gi|817089496|ref|XP_012267616.1|	PLNSAANPII
gi|817089494|ref|XP_012267615.1|	GTDIAWRSTVDW	gi|817089494|ref|XP_012267615.1|	PLNSAANPII
gi|936698029|ref|XP_014226999.1|	LTDIVQRSTVAW	gi|936698029|ref|XP_014226999.1|	HLNSAANPII
gi|939636316|ref|XP_014294173.1|	LTDIVWRVTIAW	gi|939636316|ref|XP_014294173.1|	PLNSAANPLI
gi|939636313|ref|XP_014294172.1|	LTDIVWRVTIAW	gi|939636313|ref|XP_014294172.1|	PLNSAANPLI
gi|939276044|ref|XP_014258479.1|	STDIVWRITITW	gi|939276044|ref|XP_014258479.1|	PLNSAANPLI
gi|641666914|ref|XP_008183896.1|	MIDMIWRTTVTW	gi|641666914|ref|XP_008183896.1|	PLNSAANPII
gi|641666912|ref|XP_008183895.1|	MIDMIWRTTVTW	gi|641666912|ref|XP_008183895.1|	PLNSAANPII
gi|641666910|ref|XP_008183894.1|	MIDMIWRTTVTW	gi|641666910|ref|XP_008183894.1|	PLNSAANPII
gi|328713510|ref|XP_003245097.1|	MIDMIWRTTVTW	gi|328713510|ref|XP_003245097.1|	PLNSAANPII
gi|662202708|ref|XP_008474661.1|	LTDIVWRSSVTW	gi|662202708|ref|XP_008474661.1|	PLNSAANPII
gi|939631611|ref|XP_014282181.1|	STDIIWRITVAW	gi|939631611|ref|XP_014282181.1|	PLNSAANPVI
gi|939258178|ref|XP_014248905.1|	LTDIIWRITVAW	gi|939258178|ref|XP_014248905.1|	PLNSAANPVI
gi|939258176|ref|XP_014248904.1|	LTDIIWRITVAW	gi|939258176|ref|XP_014248904.1|	PLNSAANPVI
gi|939258174|ref|XP_014248903.1|	LTDIIWRITVAW	gi|939258174|ref|XP_014248903.1|	PLNSAANPVI
gi|929381616|ref|XP_014101257.1|	LTDIIWRITISW	gi|929381616|ref|XP_014101257.1|	PLNSAANPLI
gi|939259418|ref|XP_014249533.1|	LTDIIWRSTVEW	gi|939259418|ref|XP_014249533.1|	PLNSAANPFI
gi|817085633|ref|XP_012265516.1|	LTDIVWRSTITW	gi|817085633|ref|XP_012265516.1|	PLNSAANPII
gi|817085631|ref|XP_012265515.1|	LTDIVWRSTITW	gi|817085631|ref|XP_012265515.1|	PLNSAANPII
gi|665804934|ref|XP_008550635.1|	LTDIVWRVTIIW	gi|665804934|ref|XP_008550635.1|	PLNSAANPII
gi|665804932|ref|XP_008550634.1|	LTDIVWRVTIIW	gi|665804932|ref|XP_008550634.1|	PLNSAANPII
gi|665804930|ref|XP_008550633.1|	LTDIVWRVTIIW	gi|665804930|ref|XP_008550633.1|	PLNSAANPII
gi|665804928|ref|XP_008550632.1|	LTDIVWRVTIIW	gi|665804928|ref|XP_008550632.1|	PLNSAANPII
gi|665804926|ref|XP_008550631.1|	LTDIVWRVTIIW	gi|665804926|ref|XP_008550631.1|	PLNSAANPII
gi|665804924|ref|XP_008550630.1|	LTDIVWRVTIIW	gi|665804924|ref|XP_008550630.1|	PLNSAANPII
gi|665804922|ref|XP_008550629.1|	LTDIVWRVTIIW	gi|665804922|ref|XP_008550629.1|	PLNSAANPII
gi|665804920|ref|XP_008550628.1|	LTDIVWRVTIIW	gi|665804920|ref|XP_008550628.1|	PLNSAANPII
gi|665804918|ref|XP_008550627.1|	LTDIVWRVTIIW	gi|665804918|ref|XP_008550627.1|	PLNSAANPII
gi|665804916|ref|XP_008550626.1|	LTDIVWRVTIIW	gi|665804916|ref|XP_008550626.1|	PLNSAANPII
gi|665804914|ref|XP_008550625.1|	LTDIVWRVTIIW	gi|665804914|ref|XP_008550625.1|	PLNSAANPII
gi|755928159|ref|XP_011306641.1|	LTDIVWRSTVTW	gi|755928159|ref|XP_011306641.1|	PLNSAANPII
gi|755928161|ref|XP_011306649.1|	LTDIVWRSTVTW	gi|755928161|ref|XP_011306649.1|	PLNSAANPII

gi|755928156|ref|XP_011306628.1|	LTDIVWRSTVTW
gi|755928154|ref|XP_011306619.1|	LTDIVWRSTVTW
gi|755928151|ref|XP_011306611.1|	LTDIVWRSTVTW
gi|954560085|ref|XP_014603803.1|	LTDIIWRTTVTW
gi|954560083|ref|XP_014603802.1|	LTDIIWRTTVTW
gi|951561515|ref|XP_014479680.1|	LTDIVWRATVTW
gi|746852311|ref|XP_011056707.1|	LTDIIWRTTVAW
gi|746852309|ref|XP_011056706.1|	LTDIIWRTTVAW
gi|746852307|ref|XP_011056705.1|	LTDIIWRTTVAW
gi|746852305|ref|XP_011056704.1|	LTDIIWRTTVAW
gi|826496319|ref|XP_012541117.1|	LTDIIWKTSIVW
gi|826496313|ref|XP_012541116.1|	LTDIIWKTSIVW
gi|826496307|ref|XP_012541114.1|	LTDIIWKTSIVW
gi|759077735|ref|XP_011348644.1|	LTDIIWRTTVTW
gi|759077733|ref|XP_011348643.1|	LTDIIWRTTVTW
gi|759077731|ref|XP_011348642.1|	LTDIIWRTTVTW
gi|759077729|ref|XP_011348641.1|	LTDIIWRTTVTW
gi|759077727|ref|XP_011348640.1|	LTDIIWRTTVTW
gi|759077725|ref|XP_011348639.1|	LTDIIWRTTVTW
gi|752886184|ref|XP_011260909.1|	LTDIIWRTTVTW
gi|795082282|ref|XP_011877638.1|	LTDIIWRTTVAW
gi|795082287|ref|XP_011877639.1|	LTDIIWRTTVAW
gi|795082277|ref|XP_011877637.1|	LTDIIWRTTVAW

gi|755928156|ref|XP_011306628.1|	PLNSAANPII
gi|755928154|ref|XP_011306619.1|	PLNSAANPII
gi|755928151|ref|XP_011306611.1|	PLNSAANPII
gi|954560085|ref|XP_014603803.1|	PLNSAANPII
gi|954560083|ref|XP_014603802.1|	PLNSAANPII
gi|951561515|ref|XP_014479680.1|	PLNSAANPII
gi|746852311|ref|XP_011056707.1|	PLNSAANPII
gi|746852309|ref|XP_011056706.1|	PLNSAANPII
gi|746852307|ref|XP_011056705.1|	PLNSAANPII
gi|746852305|ref|XP_011056704.1|	PLNSAANPII
gi|826496319|ref|XP_012541117.1|	SLNSAANPII
gi|826496313|ref|XP_012541116.1|	SLNSAANPII
gi|826496307|ref|XP_012541114.1|	SLNSAANPII
gi|759077735|ref|XP_011348644.1|	PLNSAANPII
gi|759077733|ref|XP_011348643.1|	PLNSAANPII
gi|759077731|ref|XP_011348642.1|	PLNSAANPII
gi|759077729|ref|XP_011348641.1|	PLNSAANPII
gi|759077727|ref|XP_011348640.1|	PLNSAANPII
gi|759077725|ref|XP_011348639.1|	PLNSAANPII
gi|752886184|ref|XP_011260909.1|	PLNSAANPII
gi|795082277|ref|XP_011877637.1|	PLNSAANPII
gi|795082272|ref|XP_011877636.1|	PLNSAANPII
gi|768420480|ref|XP_011551009.1|	PLNSAANPVI

Supplementary Figure S5. Phylogenetic tree of inotocin receptors. (A) Enlarged view early-diverging lineages of Arthropoda (Chelicerata, Myriapoda and Crustaceans) (enlarged view of dashed rectangle in B). The same colour is used for one species. Full species name is shown only for genus, which represent more than one species (*Daphnia* and *Ixodes*). Receptor sequence numbers are the same as in Supplementary Data S2. (B) Entire phylogenetic tree of inotocin receptors**.** Partial sequences shorter than 200 residues were excluded, giving a total of 93 receptors that were selected for analyses. Vertebrate and invertebrate oxytocin/vasopressin-like receptors (OT/AVPR) as well as vertebrate neuropeptide S receptor (NPSR)/invertebrate crustacean cardioactive peptide receptor (CCAPR) and adipokinetic hormone receptor (AKHR) were also included in the analyses. According to the study of Pitti and Manoj[1] the mouse gonadotropin-releasing hormone receptor (GnRHR) was used as outgroup. In most of the cases sequences of species within the same insect order are clustering together with high confidence values, i.e. Collembola (99% confidence value), Odonata (100%), Ephemeroptera (78%), Hemiptera (55%), Hymenoptera (83%), Embioptera (100%), Phasmatodea (100%), and Coleoptera (94%). However, the tree topology is not well-resolved regarding early diverging lineages, namely the Chelicerata, Myriapoda and Crustacean receptor sequences. Phylogeny supports lineage-specific duplication of inotocin receptor for some of the species (e.g. *Limulus polyphemus*). Some of similar species could share the same WGD or large scale duplication event (*Platynothrus peltifer* and *Achipteria coleoptrata*; *Ixodes scapularis* and *Ixodes ricinus*; possibly *Metaseiulus occidentalis* and *Varroa destructor* - only one inotocin receptor of *V. destructor* was analysed; the second one is a very short partial sequence and was not included into analyses), but phylogeny does not support the duplication event at the base of Chelicerata. Two inotocin receptors of *Strigamia maritima* are very different from each other, but unfortunately it is the only species of Myriapoda in our list having two copies of receptors, hence it would be too far-fetched to draw any conclusions based on this result.

(A)

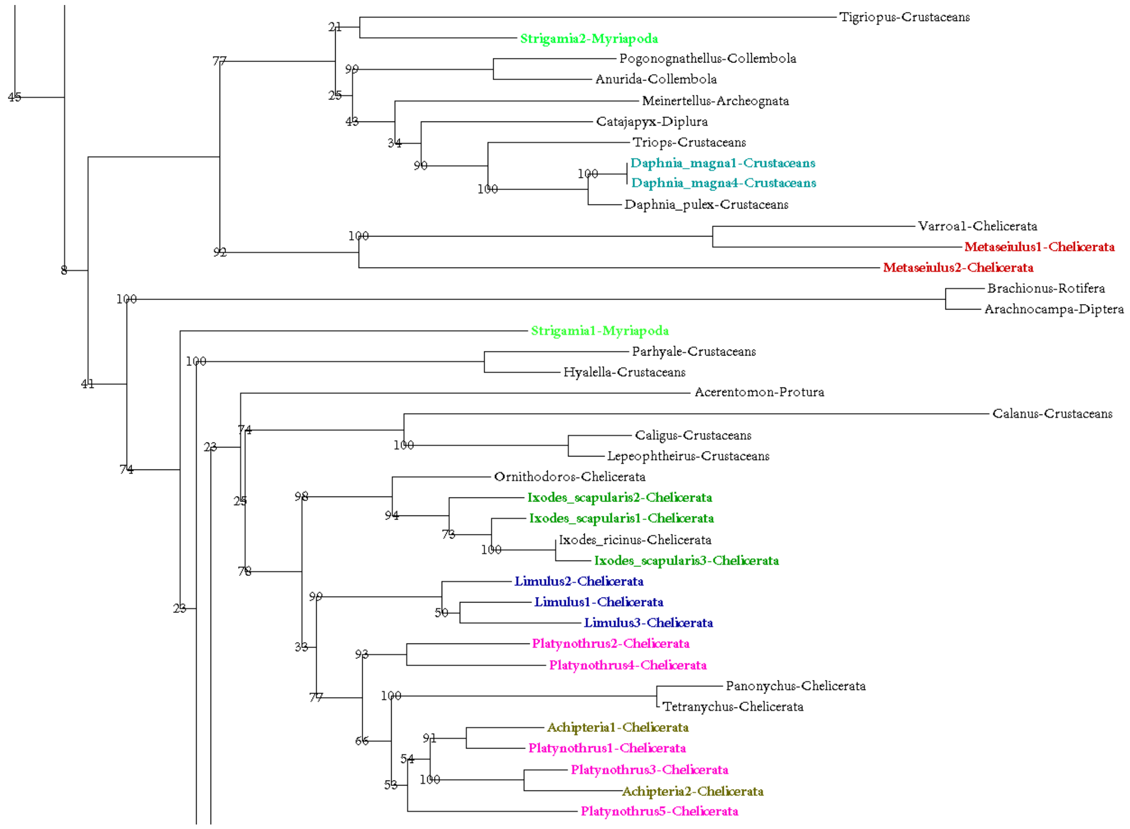

(B)

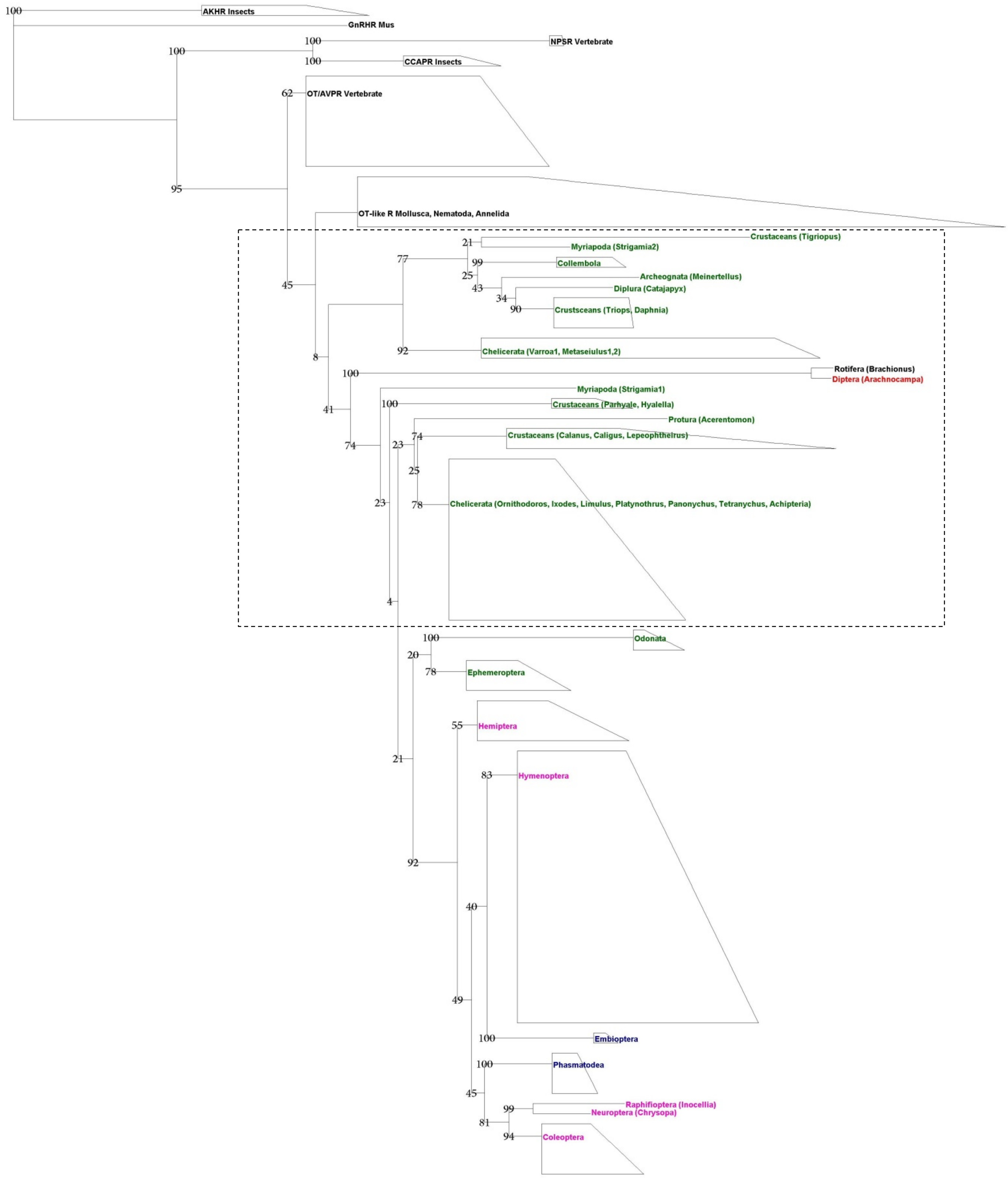

AKHR Insects
GnRHR Mus
NPSR Vertebrate
CCAPR Insects
OT/AVPR Vertebrate
OT-like R Mollusca, Nematoda, Annelida
Crustaceans (Tigriopus)
Myriapoda (Strigamia2)
Collembola
Archeognata (Meinertellus)
Diplura (Catajapyx)
Crustsceans (Triops, Daphnia)
Chelicerata (Varroa1, Metaseiulus1,2)
Rotifera (Brachionus)
Diptera (Arachnocampa)
Myriapoda (Strigamia1)
Crustaceans (Parhyale, Hyalella)
Protura (Acerentomon)
Crustaceans (Calanus, Caligus, Lepeophthelrus)
Chelicerata (Ornithodoros, Ixodes, Limulus, Platynothrus, Panonychus, Tetranychus, Achipteria)
Odonata
Ephemeroptera
Hemiptera
Hymenoptera
Embioptera
Phasmatodea
Raphifioptera (Inocellia)
Neuroptera (Chrysopa)
Coleoptera

Supplementary Figure S6. Alignment of selected inotocin-like receptors and precursors. *Arachnocampa luminosa*[2,3] (New Zealand glowworm) is the only exception among numerous transcriptomes and genomes within Diptera, where both an inotocin-like receptor (A) and precursor (B) were identified. The sequences are shown as alignment in comparison to *Tribolium catsaneum* and *Brachionus koreanus*. Two hypotheses could explain why *A. luminosa* stands out of this group: (i) the inotocin signalling system was re-acquired through horizontal (lateral) gene transfer; or (ii) there has been a sample contamination during the production of the *A. luminosa* datasets. Utilizing the phylogenetic tree of inotocin receptors, it is evident that *A. luminosa* is forming on a separate branch distinct from other insects receptors with a 100% confidence value (Supplementary Figure S5). Therefore we performed tblastn searches against the eukaryotes Transcriptome Shotgun Assembly database at NCBI and the first hits for both oxytocin-like peptide and receptor were from *Brachionus koreanus*, a planktonic microorganism, which belongs to the class Rotifera[4]. Although evolutionary distantly related to insects, the observed identity and similarity is between 81-91% for both peptide and receptor, but the identity and similarity to *Tribolium castaneum* sequences are only 27-37%. To verify if the putstive inotocin precursor and receptor are unique among other *A. luminosa* genes we performed a pairwise blast between *A. luminosa* and *B. koreanus* transcriptomes. We have found 11106 hits (out of 196675 contigs/transcripts; 5.6%) *A. luminosa* versus *B. koreanus* and 8455 hits (out of 28660 contigs/transcripts; 29.5%) *B. koreanus* versus *A. luminosa* with a very high bit score (>400). Moreover after checking the *A. luminosa* transcriptome for universal single copy genes on metazoa level we discovered that the glowworm transcriptome contains a very high number of duplications (82%), which has been illustrated by genome and transcriptome parameters of *A. luminosa* and *B. koreanus* transcriptomes on metazoa level (C). These findings strongly suggest a contamination of the *A. luminosa* sample; however horizontal gene transfer of large amounts of genetic information from some *Brachionus* species cannot be completely excluded because (i) the main cause for both to occur is the same: two species should meet each other physically (which could happen between *A. luminosa* and *Brachionus* species because the latter species is spread all over the world in fresh and semi-fresh water[4]); (ii) contamination of the samples especially of transcriptomes is a common problem in horizontal gene transfer studies[5]; (iii) the big transfers between species are reported before (entire *Wolbachia* genome >1 Mb was transferred to *Drosophila ananassae*[6]) as well as more and more evidence of horizontal gene transfer in insects accumulated during the last years[7-10]. Therefore the question remains open if the inotocin signalling system identified in *A. luminosa* is due to horizontal gene transfer from some *Brachionus* species (Rotifera), or it is an artefact due to sample contamination.

(A)

```
Tribolium       ---MY----TPKLSQMDISENS--TYLFDKHE-DRNNTDRDENLARVEVATLAIIFLVTV
Arachnocampa    -MTSYQENILPNYSEQDKFEN--SNVSLNNSNSVQQFQLNSSLLTKIEVFLLSSLFLLTV
Brachionus      MTSSYQENMLLDYSEQKKSEEELQNLSFNNSDSSEQFQINNKLLTQIEVFLLSCLFVLTV
                    *      . *: .  *:   .  ::: :  .:   ... *:::**  *: :*::**

Tribolium       IGNSTVLLALWTRR--------RYAGRKKLSRMYFFILHLSIADLITAFLSVLPQLAWDI
Arachnocampa    IGNLIVILIILLYRNMSPKRARWFKLNKNISRMSFYIIHLSIADFNVAFMSILPQLIWRQ
Brachionus      IGNLTVILIILLYRNMSSKRARWFKLNKNISRMSFYIIHLSIADLNVAFMSILPQLIWRQ
                ***  *:* :   *         :  .*::*** *:*:******: .**:*:**** *  

Tribolium       TYRFYGGFLLCKVVKYGQTLGPYLSSYVLMATAIDRHQAICYPLTYCSWTSRRSKVMVYL
Arachnocampa    SVVFNHSHFLCKFVAFSQVFSVYASTFLLIVMAYDRFKCICWPIKSCSWNYRHALVPVFF
Brachionus      SVVFNHSHFLCKFVAFSQVFSVYASTFLLIVMAYDRFKCICWPIKSCSWNCRHALIGIFV
                :  *  ..:***.* :.*.:. * *:::*:. * **.:.**:*:. ***. *:: : ::.

Tribolium       AWVASLAFCIPQLTIFTYTSVGEDEYDC---WATFQEPWGKRAYVTWYSISVFMVPLVVL
Arachnocampa    SWILAAVVSSPQLFLFKIQHMSVQHYTVETCSVKWLSKKHEGLYLLFHMSTQFLIPLFVL
Brachionus      AWIIAATISSPQLFLFKIQHMNAHVYTVETCSVKWMSKRHEGLYLLFHMSTHFLIPFFVL
                :*: : ... *** :*.   :  . *      ..: .   :  *: ::  : *::*:.**

Tribolium       IFTYTSICIEIWQSSESS---LR-PRSSQK------------------------------
Arachnocampa    TFLYSRIFMTVSKNIKQKHASIRFERESSKTATEINQLNQENSLNTQNGLITEVKHDSDN
Brachionus      TYLYSRIFLTVSRNISRKHASVRFERESSKTGTEINQLNRENSLNTQNGLITEIKNESEN
                 : *: * : : :. . .   :*  *.*.*

Tribolium       ---------------------------------------------------SA-------
```

```
Arachnocampa      SIFRDKKFGILWWIRSRFNKGIRHKILFTSTKKRGKNIKFGKTGNFLMNDQTMPKEYEMR
Brachionus        SIFRDKKTGIILWLRSHFNKSIRHKILYTSTKKSGKNIKFSRSQNFSLNN-SLPKEYEMK
                                                                     :

Tribolium         ----------------------------------P---GKRTPLISRAKINTVKQTIAVI
Arachnocampa      LMLKRKETLSDS-EKYSMRLDKNSVYSKSFKSNFPIRQTFSGKALTRSKIKTLKLTLTVV
Brachionus        LMLKKNDPLSESDNRYSMRLDKNSVYSKSFKKNFPIRQTFTGKALTKSKIKTLKLTLTVV
                                                    *         ::::**:*:* *::*:

Tribolium         VMYIACSTPFILAQLWATWDP------QSPFIDGPVF--VILTLLYSLNSCVNPWIYLAF
Arachnocampa      ITYVMCSLPFYVCTFIHFLLGLSAHNHSSLFTKTLVYITISTNMLFQLNSCANPFIYLFF
Brachionus        ITYVMCSLPFYVCTFIHFLLGLSAHNHSNLFTKILVFATISTNMLFQLNSC---------
                  : *: ** ** :. :            .. * .  *:  :  .:*:.****

Tribolium         NRELPRLLLRHYTASSKNYRSATGGNSASNSSGDAQSTSLRPFSRWSLCNSARSNKYPTR
Arachnocampa      NGNICKLAKRLKTKV---------------------------------------------
Brachionus        ------------------------------------------------------------

Tribolium         VPHRPYVAQYNARRWIVTTTT
Arachnocampa      ---------------------
Brachionus        ---------------------
```

	**Identity[a] %**				**Similarity[b] %**		
Tribolium	100			Tribolium	100		
Arachnocampa	27.5	100		Arachnocampa	28.5	100	
Brachionus	24.3	81.3	100	Brachionus	26.2	85.2	100
	Tribolium	Arachnocampa	Brachionus		Tribolium	Arachnocampa	Brachionus

[a]calculated using BLOSUM62 matrix using alignment above at http://imed.med.ucm.es/Tools/sias.html

(B)

```
                                                  mature peptide
Tribolium         ------------MSTIITSIILLVLSESLVSGCLITNCPRGGKRSKFAISENAVKPCVSC
Arachnocampa      MNENFLMMNLNDKKNLKILFILVVINLNVINACYITNCPWGGKRSQPFLDSENAHQCRKC
Brachionus        MNENNIMMNLSDRKNLIILFILIVINLNVINACYITNCPWGGKRSQPFLDFENAHQCRKC
                               ..:   :**:*:. .::..* ***** *****:  :. : .: * .*

Tribolium         GPGQSGQCFGPSICCGP-FGCLVGTPETLRCQREGFFHEREPCIAGS-APCRKNTGRCAF
Arachnocampa      ASG-LGMCFGPRICCGPDMGCLIDTKETSVCQLEDLKSN-VPCQPYGKICDKVEFGRCAT
Brachionus        ASG-LGMCFGPKICCGPDMGCLVDTKESSVCQLEDLKSN-VPCQPYGKICDKVEYGRCAT
                  . *  * **** ***** :***: * *:  ** * :  :  **   .    : : ****

Tribolium         DGICCSQDSCHADKSCASDDKSPIDLYTLINYQAELAGDK--------------------
Arachnocampa      SNLCCNPEHCLEDSTCVSEENDYSEDYKEIDTKLLKALKRLISKKRQKNENYKSHNI-DN
Brachionus        SNLCCNPEHCLEDSTCASDDIDYSEEYKEIDAKVLKAIKRLISKRRERNEKYDEGHISDK
                  . :**. : *  *.:*.*:: .  : *. *: :   * .:

Tribolium         -----
Arachnocampa      YQS--
Brachionus        YQSNF
```

	**Identity[a] %**				**Similarity[a] %**		
Tribolium	100			*Tribolium*	100		
Arachnocampa	34.9	100		*Arachnocampa*	37.3	100	
Brachionus	37.7	84.4	100	*Brachionus*	26.2	90.8	100
	Tribolium	*Arachnocampa*	*Brachionus*		*Tribolium*	*Arachnocampa*	*Brachionus*

[a]calculated using BLOSUM62 matrix using alignment above at http://imed.med.ucm.es/Tools/sias.html

(C)

**File**	**Complete Single-copy BUSCOs**	**Complete Duplicated BUSCO**	**Fragmented BUSCOs**	**Missing BUSCOs**	**Total BUSCO groups searched**
short_summary_arachnocampa_luminosa_met	67%	38%	5.2%	27%	843
short_summary_arachnocampa_luminosa_trans_met	98%	82%	1.0%	0.3%	843
short_summary_brachionus_koreanus_met	57%	10%	10%	32%	843
short_summary_brachionus_koreanus_trans_met	70%	15%	13%	16%	843

Supplementary Data S1. Putative inotocin precursors. Sequences of 144 novel inotocin precursor sequences from Arthropoda are shown. Inotocin-like peptides are highlighted in yellow. (A) 75 newly annotated precursor sequences containing the inotocin-like peptide domain. (B) 35 deposited inotocin precursor sequences derived from NCBI containing the inotocin-like peptide domain. (C) 23 newly annotated inotocin precursor sequences containing the neurophysin domain but lacking the inotocin-like peptide domain (the first sequence of *Ceratosolen solmsi marchali* is from NCBI). (D) 11 non-annotated inotocin precursor sequences presented as BLAST search results. Sequences from (A) and (B) were use to prepare the alignment illustrated in Supplementary Figure S1.

(A)

>Symphylella_vulgaris
MSSGKSDITNAFVLLTFVSTVSACFITNCPIGGKRSGHSLSSSVALRQCSPCGPGGAGRCFGKSACCVPGMGCFLGPAHTATCRLEHSYPVPCDNGTPACG
ENGNGRCAVEGLCCTGDGCFYEDTCRDSSLLTSSLHHSGSTLEDETTQ-
>Strigamia_maritima
MKSTHFVNIFIYSVFIFIMADGCYITNCPPGGKRSGNEKSGRGVRQCTPCGPGGIGRCYGPDICCGANVGCFVGTRESAICRLENLYSLPCQNEGRACGTD
GTCSADGFCCSTDQCKADESCRGKVHHTNNLQRVLDGEIDLNDVMGPQR
>Litopenaeus_vannamei
MQISAVVAMALLLGSGTACFITNCPPGGKRSSPSAQLGSVRTCAQCGPGLQGRCMGPDICCGPEIGCYMGTREAFLCRSENLVPVTCSNDDLKACGRQREG
RCASSGVCCTEVKCEFDINCVIEGVDRHRYSLLDTLTEAQWRI-
>Lepeophtheirus_salmonis
MAFGYPLITLLLIFQIANACFITNCPVGGGKKGSGSTNLGFLNSSFQYKQCSSCGPNNTGRCFGPRLCCSSEFGCFVYSNDIAKNPCQTEAYDPVPCQNNV
KSCSSVLKNGQCVFDNYCCNSSGTCRFVEEELICSVSDDKKNYEKEKELVDDLQKNMIYIRRQAYLGNGVPRLQQLNINDLEQ-
>Calanus1_finmarchicus
MITSWKILDPRIRRSWSFLIILLAQTMSASHGCFISNCPVSGKKRSVGEEQMVSFQAREEVLACPSNPAGLCYSPGLCCVQGGCYADKGCLPQMEESRENR
VEPRLNKRDLRFLYGWAPAHEKMFGQTGFSIPCIGPYCNGNEKMIMRKEKQSQEKKSDDKVLDGSSNYDSLVYNENLSNQRCNWMDLYLAR
>Caligus_rogercresseyi
MALCLLQVTESCFITNCPVGSGRKRADPGLFSPKNRRQLDECSPCGPSNEGRCYGPNLCCGSTFGCFVYAKSLQHQNPCLTEARLPLPCRNKGGSCSGILE
NGQCVFENYCCSPSGTCRFVDESICSNSTVPQLGDSDFMEDFRRRLFLARELHSL
>Tigriopus_californicus
MQITFDRRCFIVLGLLSLLKAVSVNSCFITNCPSGGKKRSELLTVASRKCPPCGPLGMGQCFGPSMCCGQYIGCHLNTPDTQVCKTENSNPIPCDNDVPRC
QSVRRGFCATNGFCCNDQGECTPEEKCLVENLSDFPSPFILRRHLSSSASLASRPTVEKAMITIPQPNAKAYQEALNLMQIIPKSNSALNKYLSRHGDVK
>Speleonectes_cf_tumulensis
MHLRPMDVLLSALCVLHVAGISTACFILDCPLMIRNRTVRTLKSSTSRQCTSCGPDDLGRCYSPNICCGPESAATSAPKNRRLPVRELLPCSL-
>Sminthurus_viridis
MINLNCGICLFLITLSVVTGCFITNCPPGGKRSGIERKTRQCEQCGPPGAGGRCYGPSICCSPELGCLIATPETYSCQLENRHTTLCQNPGATCAGPNGDI
SGGQCGAEGICCNSETCTMDETCHDEASVTRESVAKLSGLYHHRYPLDVVFQRFVDGQHSQHHRLPARLPLPTQSSSSSNDY-
>Anurida_maritima
MRKYHTSIICLVWLISVSAAFGCFITNCPPGGKRSGPLSTQARQCESCGPEGSGGRCYGPNICCSPGSGCLIGTPETVACRSEARFSTPCLNPGISCSGSG
SLSIKGQCTGDGICCSSETCTHDENCWERSMTSGTGDSQEFNQDLPLREDRFLIPLPATHEKFRSFPISQIIRVGRRKNQFEDLRNHLLHRPQMDFQPPIS
STESLYP-
>Pogonognathellus_sp
MIQFVTYNLWLGVLLVSFVAASGCFITNCPPGGKRSGTTALTVRQCERCGPPGMMGRCFGPQICCSPEFGCFFSTAETFVCQLENRFPTPCHNPGSSCSGN
GDTGLINGQCG
>Folsomia_candida
MKTSLLFAFGIVMVESLLVSGCFITNCPPGGKRSGADTKIRQCEQCGPPGMNGLCYGPHICCSSELGCLFGTSETLTCFQENLMYNTPCQNPGESCSGNDA
TNPINGQCATLGVCCSSDTCTIDDNCHKKDEQQNVSGHSRSTVAMDGSHTKEHATTMFQNPLDRWIPRRFGLGRPTGQRKHVRIGVKPDAQILAPENSLMD
RLVIFPDTSGRISSDEY-
>Occasjapyx_japonicus
MSSPVRIHPRLLALACVFCISSACFITNCPPGGKRSQQAHYKPQASRQCAVCGPNGQGRCFGPELCCGPEIGCYMRTRESSSCRAENLFPVQCENRARPCG
SERSGKCAAVGICCTEDSCTSDQTCRASPGKEAVAAAVLPRGPSRPQLPSDLVSFLEEVVDNMAPASGSEV-
>Meinertellus_cundinamarcensis
MKSSMSTPAALFALLFVVSITSSCFITNCPPGGKRAMGAPGPRGVQRQCPSCGPHRSGRCFGPDLCCGEFGCHLGGRDPVCRGEMSAPGLCTNPGRPCGVG
KCAAQGVCCSETACALDSMCLDSWPSRNVVQMNSGLASVLENMLEEVPSENGDDKK-
>Machilis_hrabei
MSADMTNSASSTFALFTLLLFVGCVTSCFITNCPPGGKRGLGSPGPRGEPRQCPSCGPRHSGRCFGPDLCCGDFGCRLGARDASCRREMSAPGLCINQGRP
CGVGVCAAQGVCCSETSCALDASCMDSWSPRQLVPVNNGLSSVLENMLEDIPMESGDDRR-
>Tricholepidion_gertschi
MVARKNPQTVVICLMAAMSAATACFITNCPPGGKRSMGQVGTHATRECTSCGPAKLGRCYGPAICCGPQIGCLVGTREAAVCQTENFYPVPCANRGGQCGG
ESGRCAATGVCCTEESCSIDPSCHVTPQETILTPVSRQMYPINSAMNNYLEEAVDALPSHVILENRR-
>Eurylophella_sp
MVHRRGATLSAMLIVHVVSACFITNCPPGGKRSSSITTVNARQCSRCGPGLSGRCFGARICCDSRRGCMLASSTEPSLLPCLAESMQTTPCRNRAASCGQS
GAGQCASQGICCLQDSCHADVKCHENYD
>Apachyus
MSFSLRFFAIFTLLLCLGTGCLITNCPKGGKRTSITVSFILKF
>Gryllotalpa_sp
MKVYYLLVFISLIVLNTACMIINCPRGGKRAMKINEISSIRSCARCGLNNAGHCYGPDICCAPELGCLIATPETVPCQEESQSPDPCIDTDLGPACHNGKG
HCGANNICCTQDSCYSDQTCQFGFPVE
>Teleogryllus_commodus
MAQFKLYSNIIVLMMILTFSCACMIINCPRGGKRSAMENNEILPIRNCARCGPNNTGHCYGPSTCCAEELGCLMGGPEAKPCEAEALSPDPCVEPALGPRC
FGGQGFCATSTLCCTQVSCHTDPSCQFAVAGHKSVGATDLALPPHRSYVYSLLNALAKNQDHNPQNNVE-
>Locusta_migratoria_manilensis

MKPQLAVLLALVVSLAAACLITNCPRGGKRASLQHRCAACGPGGQGVCLGPHICCGPRMGCRLASADADSAACRAAPPCPLDSPEMRCAAGRGRCSAPGVC
CSQDSCHIDPSCVADTTEPADRYALDIFAVNDGDRDDMRI-
>Galloisiana_yuasai
MRLKQCLRMLALLVAVVGISSACLITNCPKGGKRALNHSKDSVRQCPRCGPAKLGHCYGPAICCATQFGCLVATADTAICQGESLYPEPCANTMPASSCRG
EAGKCAANGICCTSESCHVDMSCRLTSRGEDDVIQFPDLSSDAKGVFSLFSNLGSYEPDHQAYRLPSNVERD-
>Haploembia_palaui
MINFRFARFIILITLAAVCTACLITNCPIGGKRSLLHGTESVRQCPRCGPAKLGHCFGPTICCGHEIGCLINTPDTLVCLKEHLSPDPCVGPTATNCGNRK
GKCAADGVCCTPESCEID
>Aposthonia_japonica
MVNFRFARFIILITLAVVCTGCLITNCPKGGKRSLAHGSESLRQCPRCGPAKLGHCFGPTICCGHEIGCLINTPDTLVCLKEHLSPDPCVGPSASNCGNRK
GKCAADSICCTPDSCEIDASCRFGSPQHFRNVGIYPQYVE
>Cryptocercus_wrighti
QLGTLATLAILLIGVCTACLITNCPKGGKRAGSHSQDSHPIRQVFLGYLTTLYQLLSL
>Zootermopsis_nevadensis
MKMQLGTATLLAVFISLCTACLITNCPKGGKRAGTHSQELHTIRQCARCGPAKLGHCYGPAICCGPQIGCLVATPDTARCLSEAASPVPCTAPTGAQCGEG
KFAGRCTANGVCCTHESCHIDITCQLTTSDAPELIDVSADQTNPLYSLYSSYQQENPGLGLSE-
>Gynaikothrips_ficorum
MICVKIPSIFLLLLAMTSACLITNCPRGGKRNGARPPTKGSIEELPFCTLCGPPELGGLCFGPTMCCSPTGGCELGEPHVASCLGVHTVPPLCRLPPQSMP
LNGPYSPSAPSQTRATSLVTTCGSGICAANGLCCTSDWCRADDRCLSSASQSNLVIPDHNLPANTRQEPQQAEFNDWHS-
>Halyomorpha_halys
MYRALLFLTALCLVQSCLITNCPRGGKRSLRPGLRDPDCRRCGPGLEGRCVGKTVCCGPRIGCLIGTPAVLSQCAAEPMQLRGGQRCSIGVCLADGVCCSP
DGCKMDMSCEVEQSVDVCSLAEQQVY-
>Diaphorina_citri
MYAMISRLLVVCLVGWYFFGSTWCCLINNCPTGGKRSALMALEERRQSHQKRAKLWNDRNIISCNAWGAEGVCVGPLCCDPSRSEGDPGSKCGVDYKTCGS
SREVMEEYALCLSKGLACDLNTLKCCNMYAIKVPTEVKLQLHQ-
>Liposcelis_bostrychophila
MNTFQFVVLYIFVNSVNSCLITNCPRGGKRDGSENNLIEAPRSDIKTCTPCGPNNQGVCIGRDICCGKHLGCLISSPSTKICRRVREFPESCLSSFNPCGN
GGLCVARNVCCFKNLCRIDDTCKTIPYVDLGYRLGTSDILNRLSSYSVDYPLELPSNMPIGSTSLDEVWGSKEDSIVPNYNFKIEAS-
>Menopon_gallinae
MYNIFQFQLRLGLLVATFGLVVSCLITNCPRGGKRGDNSLDKTSSYPDTNYCIPCGPDDQGVCLGKNVCCSREGCTTRSRYTDPCRSIQNTRISCLSEVNV
QTCGDKGRCKAKFICCSRDQCVMDLSCKSMPPAKYRDVNE
>Cotesia_vestalis
MMHKFLLVFFFITSSFACLITNCPRGGKRGESPLLSMPSLIKECSSCGPGDQGQCFGPHICCGASIGCYIRSPETYKCRKESLHSHPCVSGFAMCRDNTAR
CAANGICCSQESCFLDSSCKIGDEFLSDRKIDQEFSRLIGENDQMIN-
>Nasonia_giraulti
MSKVIIVLTTLVSLSYGCLITNCPRGGKRGDPTFLLENIARECPACGREEQGRCFGPHICCSPSMGCLIGTPETLRCRKESLYSRPCVAGFAMCQGNSGRC
AANGICCSQESCFIDSACKLVDETGNDRKIGAEFGAFLLENAGTNEHIL-
>Chrysis_viridula
MQYKMLTKLILFASMFAVVYGCLITNCPRGGKRGDSAVTLDHLARECPSCGPNRLGQCFGPHICCGPSIGCFIGTPETYKCRKESLFSRPCVAGYAMCRGN
SARCAANGICCSQETCHMDSTCRISDVDTRDRKEEGVDVAAVLSDNELSADSVQ-
>Polistes_metricus
MTKKILIFTSFIFISSACLITNCPRGGKRGDIEVPESPIRECLPCGPSQLGQCFGPNICCGPTFGCHIGTSQTQKCRKEQFDSSPCMAGFAMCNGNKGRCG
AGGICCSQDSCFIDPSCRFSTVDSPFRRSIDSDLKAIASEIEILKEEAK-
>Mischocyttarus_flavitarsis
MTKQILVFITSFILISSACLITNCPRGGKRVDNKVPLVTTTEYPVKDCISCGPNGQGQCFGSNICCGPTFGCHMGTSYTKKCRKEFSDQISCLSGFAMCSG
NVGRCAAIGICCSQESCFLDPICRLSDNLPVGRKFNT
>Chyphotes_mellipes
MAKELFVFAALLVVAYGCLITNCPRGGKRGGGKHGEVVPAFEDVRVTRECPSCGPNRLGRCFGPRICCGPTIGCFIGTPETYGCRGEGSPFSKPCVAGYAM
CRRNTARCAASGICCSQESCHIDASCRLSETSDGGGGNGHERRFDSDLSAILSENVGTSVEMVR-
>Pseudomasaris_vespoides
MAKELIVLIAIVVLASACLITNCPRGGKRGDIAALESVVRECPACGPNRSGQCFGPHICCGRTIGCYIGTPETYRCRKESLYSRPCIAGYAMCRGNTARCA
ANGICCSQESCFIDNTCRIPDIMAKDQKIDSDINMIFGENEISNELTQ-
>Brachycistis_timberlakei
MLKKFILIAILISLSYGCLITNCPRGGKRGEAVVSLETIARECPSCGPNRAGQCFGPHICCGPTIGCFIGTPETYRCRKESLFSRPCIAGYAMCRGNTARC
AANGICCSQESCSMDAMCKIPDALRHDRKFDSDVSSILSESEISNEII
>Crioscolia_alcione
YGCLITNCPRGGKRGEIAPLLESVARECPSCGPNHSGQCFGPHICCGPNIGCFIRTPETYRCRKESLFS
>Telenomus_podisi
MIAWVIVLVSIFSLSSACLITNCPRGGKRADQSLMLMETSIRECTSCGRDGSGRCFGPYICCGSTIGCFIGTPETYKCRKESLYSKPCIAGFAMCRGNKGR
CAANGICCSQEACYAD
>Corydalus_cornutus
MYPVVKYFVLVIVLVSVMIDSCLITNCPRGGKRNGSDRISNEQFIKQCISCGPQRSGQCFGPSICCGSFGCLVGTPETVRCARDGGFHEPEPCIAGQASCR
GDSGRCAADGICCTQESCHTDSSCAFDEERHDRSHNGRPAIPIESIFSVLSPYRS-
>Aleochara curtula
MFSSKCCLLIVGLACVVLQAVDGCLITNCPRGGKRSGKYLPDNNIKPCIACGPGHSGQCFGPDICCGPFGCLLGTHETARCRRDGNFLEPEPCIAGNASCR
RNSGRCAAEGICCSQ
>Meloe_violaceus
ATNKFLLVLIVLTIFTNYFINGCLITNCPRGGKRGNTRYLQKDTQIKQCINCGPGNNGQCFGPQICCGPFGCLFNTIDTVRCQKDGNFHQHEPCIAGFMNC
HHYNGRCATDGICCNQDSCHIDKTCSITTTIGFIENEKTRNFLETSPADLYNFINYQSELNSE-
>Priacma_serrata
AFFVLNSFLADSCLITNCPRGGKRSRYNEENIKQCISCGPAHSGQCFGPNICCGLFGCLIGTSETIRCQREGFFHEREPCIAGNAFCRKKHRTMCQRGHML
HARLLPHRQNYAHLTTNKQN-
>Gyrinus_marinus
ALLDICDSCLITNCPRGGKRSGRSQHENNIKQCISCGPGRSGQCFGSNICCGPFGCLMGTSDTIRCHREGYFQDSEPC
>Arachnocampa_luminosa
MMNLNDKKNLKILFILVVINLNVINACYITNCPWGGKRSQPFLDSENAHQCRKCASGLGMCFGPRICCGPDMGCLIDTKETSVCQLEDLKSNVPCQPYGKI
CDKVEFGRCATSNLCCNPEHCLEDSTCVSEENDYSEDYKEIDTKLLKALKRLISKKRQKNENYKSHNIDNYQS-

>Pseudomallada_prasinus
MNNNSLKATIVTFVIFTTLVHSCLITNCPRGGKRSNLLDSKISTDSIKP
>Atelura_formicaria1
ACFITNCPPGGKRSMGQMGLHVNKQCTSCGPARTGRCYGPAICCGPRFGCLVGTRDTAVCQTENFTPVPCGNRGPSCGESGRCAANGVCCTEETCTIDPSC
RISNEDISSSRQMFPITNSVSMYLEELNDDLPARVGSDRR-
>Atelura_formicaria2
SLSKGILSVTIFFLAVISVTTACFITNCPPGGKRSMGQMGLHANKQCTSCGPARMGRCYGPAICCGPRFGCLVGTRDTAVCQ
>Solenopsis_invicta
MLKELVVLASLIFLSYACLITNCPRGGKRGDIAPSLGTVERECPSCGPNRLGQCFGPHICCGPSIGCFIGTPETYRCRKESLYTRPCIAGYAMCHGNTARC
ASNGICCSQESCHMDTSCRISDVVGNDRKLDANLNVIFPDNEVSSDILQ-
>Ixodes_ricinus
MALSHMLLLAVVGGTSACFITNCPPGGKRSSEPSPARLVSSQFRSSLTVSWCSLLGVDFQRMSFYRMPDY
>Scolopendra_subspinipes1
HWLLFFYVIGMTSSCFITNCPPGGKRSLGEHKRTLRQCTACGPGSQGRCFGPDLCCGEFFGCYLKTRESAICRFENLSPIPCDNDVLSCGPDGAKCAADGL
CCSSDQCKMEERCR
>Scolopendra_subspinipes2
MRSSTCHWILYLSLVGLVSACYIINCIDNDGRKRSIDHQKKAIRQCMSCGPGMKGRCFGPDLCCIESFGCFTQTPESAVCHLENLSPVPCDNDTPSCGLGA
NCAVDGLCCNTSQCKRDERC
>Lithobius_forficatus
MASHAAQSSVFLVVLLCVFHITLACFITNCPPGGKRSLGDQKGTTRQCTACGPGGLGHCFGPNLCCGPQIGCFLHTRGKCHLPF-
>Homarus_americanus
MQLGVVVVVMTVVVGSTTACFITNCPPGGKRSGPTAQLGRTRTCTACGPGLQGRCLGPEICCVLGIGCFLGTREARMCHAENLVPVTCANRDLKSCGRMQE
GRCAAAGLCCTEMKCEFDSSCTVEGREERVGKQRAERQHLTFLSSLPEDQWNL-
>Calanus_finmarchicus3
MITSLKILDPSIRRSWRFLMILLAHTIPDSHGCFISNCPVSGKKRSVGEEQLVSFQAREEVLACPSNPAGLCYSPGLCCIQGACYADM
>Dermatophagoides_farina
MFRRESFSMMKIILVLCLIWICLFNDVRGCFITNCPRAGKRSIMTESNLGSNHKKKIKIKCTRCGPSLSGRCYGPNICCSPLTGCNVGGFTAARCSLEAYH
PILCTNPGTVCGPNGKGVCALNATCCTNGKYFICFFYCKKN
>Onthophagus_taurus
MVKLIIFTLILCIIQDQLSHGCLITNCPRGGKRNGRINSLDTNIKQCISCGPARSGQCFGPGICCGPFGCLIGTPDTLKCLKEGQFHENEPCIAGNSSCRR
NSGRCASEGICCTQGNF-
>Nicrophorus_vespilloides
MHSPPSTMFILKSSALLLVLAVVFVGLCDSCLITNCPRGGKRAMQDNTQIQCITCGPGRSGQCFGPGICCGPFGCLLGTQETVKCQREGFFHGREPCIAGS
APCRKNTGRCAAEGLCCSQGIIIQLLLLLHLRLTHRLHHPMMI-
>Oryctes_borbonicus
MNSLLLIGVFCIAGDQLIEGCLITNCPRGGKRSGKMSHLAAANQCVSCGPGHTGQCFGPNICCGPFGCLVGTAETLRCQKDGFFHEAEPCIAGNSPCRRNT
GRCATDGVCCSQGIRYIDS
>Dendroctonus_ponderosae
MTSRSSSKIAKMISQILMFILLSNILVSGCLITNCPRGGKRSGKYGLSTSNNIKQVIQFSLIQCICIPCGPGGAGQCFGPSICCGPFGCLLGTPETIRCQR
EGMFHESEPCIAGNSN
>Leptinotarsa_decemlineata1
MVRSVPLTIIYLFIFVMYVESCLITNCPKGGKRGGEFSTSESNIKTVSINLNNYKHILQCISCGPGHTGKCFGPNICCGPFGCLLGSPETERCHKEGLFQE
TEPCIAGFSTCRKNTGRCAAEKICCTQGIWTFFLVSN-
>Leptinotarsa_decemlineata2
MSKTVSLFTLFLSVFVIFVESCLITNCPIGGKRSGKFSLTESNIKQCISCGPGHTGQCFGPNICCGPFGCLMGSHETFGCQRGGFHESEPCIAGFSSCRKN
TGRCATENICCTQGIFKIRSYT-
>Hypothenemus_hampei
MVSKRNQLICTAGSNSTKLSIFMACLITVSGCLITNCPRGGKRSGKFLEAVVLFKCIPCGPGGTGQCFGPKICCGPFGCLLDTPDSIHCQNEGMFYDLEPC
TAGTMNCRKNTGRCATNGICCSQGKRTVLVNSSSVYN-
>Anoplophora_glabripennis
MFKNILLLVSLLVVFGEIFVNCCLITNCPRGGKRSGKFGALEANIKFQCVSCGPGHSGQCFGPSICCGPFGCLMGTPETLRCQREGFFHEREPCIAGGASC
RKNTGRCAIDGICCSQGNNSGFLKKLKNNLFLSLQILVMSTNNARWKTKAGFFPITLWEWICII-
>Agrilus_planipennis
MYTNVCLVFVIVLCYSLVFGCLITNCPRGRKRNGKMEGDIQPVCTSCGPGLTGQCFGPNICCGQFGCLIGTSDSLKCKYEGLFNGPEPCIAGNSSCRWKH
GRCATQGVCCNQGKCFTSFYLLLNVLDISPYQGIIRRKVLLNKNLTNVGTLNIE
>Homalodisca_vitripennis
MRGAVVLTVCSLATACLITNCPKGGKRSQSTIDNTLGTEIVSLVACLRCGPGLQGRCLGPAVCCGPRLGCLVATPGIVRQCAGAAEGPSPHGKPCITPSGL
GVCATDGVCCNSGGLYFPSPQIRHIYRNFEISQY
>Varroa destructor
MKLHVLVLANIVGLSLTCFITNCPIGGKRSDTGFGLVQFSSDFRKCPPCGPGSTGQCFGPNICCNSESCLIDAGDSPHLRSCKREALKLKPCTNTGMRCGS
ENKGHCALNRFCCTS
>Ephemera_danica
MHRKYEMATKVYLTLTALIIVQVTTACFITNCPPGGKRSSAVVSETQPKSVIRQFPITCSSYFVQCARCGPGLTGRCFGPRICCESSRGCLLGSPLEVFML
PCLVESLQPTPCRNQASNCGLTGDELCAAPGICCSQGK
>Ladona_fulva
MYSRQGMSMDQRQWLLVAVIFALLGLTSACFITNCPPGGKRSDVAFGRIPLRQVMVVSSVLSFKCSRCGFGGRCFGPAICCSLKTGCILAEKSNNLPLLRP
CAIEAGLPGACISGSKRCGNSGGRCASDGICCNDGKRN
>Catajapyx_aquilonaris
MSPFRVHQTLLAVACIFCITSACFITNCPPGGKRSQSHYKPQAIRQVSSAQCAVCGPNGQGRCFGPELCCGPEIGCYLRTREASACRAENLFPVQCENKAR
PCGSERSGKCAAMGLCCTEADSCTSDSACRGAPSKEVVAASRGQRPQQLPSDLVSFLEEVVENIAPASAPEV
>Blattella_germanica
MQLALVFVLFCAVCRACLITNCPKGGKRAGVQSQDSTVSIRQCARCGPAKLGHCYGPAICCGPQIGCLIATPETTRCLSEAASPSPCTTPSGAPCGSGKNA
GRCAANGVCCTHESCHIDVSCHPSATDIAIGNTDQLNSFYNLYNVISSAYQQENP
>Pachypsylla_venusta
MIPKIFFFLIFLCFLNFSFSCLISNCPKGGKRFDKKFPQNKKEIFDKSKVSTLYIGD
>Triops_cancriformis
IHSAPCFITNCPPGGKRSFPMSSSQHFL

>**Brachionus_koreanus** gb|GBXV01012066.1| TSA: Brachionus sp. 'koreanus' Rotifer_07959 transcribed RNA sequence
MNENNIMMNLSDRKNLIILFILIVINLNVINACYITNCPWGGKRSQPFLDFENAHQCRKCASGLGMCFGPKICCGPDMGCLVDTKESSVCQLEDLKSNVPC
QPYGKICDKVEYGRCATSNLCCNPEHCLEDSTCASDDIDYSEEYKEIDAKVLKAIKRLISKRRERNEKYDEGHISDKYQSNF

(B)

>Camponotus_floridanus Gruber, C. W. & Muttenthaler, M. PLoS One 7, e32559 (2012)
MLKQLVICASLIFLSHACLIVNCPRGGKRSDIASFLKTVTRECPTCGPNHMGQCFGPRICCGPNIGCFIGTPETYRCRKESLYTKPCIAGYAMCRGNTARC
AANGICCSQ
>Limulus polyphemus gi|926609726|ref|XP_013784878.1| PREDICTED: conopressin/neurophysin-like [Limulus polyphemus]
MHWTSFIMILGFCLAVTIKGCFITNCPPGGKRSIGTFATHYTRECGQCGPGGMGQCQGPDICCSDIFGCF
IKTRESVICRYENLQVIPCNKKGKICETVPHGYCAAPGICCSAIQCAVDDECPTGVLDKGVAYKYDHLRF
VLLPWNMNGKLPD
>Ixodes scapularis gi|506953581|gb|AGM20426.1| inotocin preproprotein [Ixodes scapularis]
MALSHMLLLAVVGGTSACFITNCPPGGKRSSEPSPARLCPRCGPAGRGVCYSADVCCAGSMCILNDALAT
LSCRAEALHGVACHVPGKRCGTDGRCAIRGYCCGPDGCTKDSSCSGGVPTDQFGSAVDILEYGMSER
>Metaseiulus occidentalis gi|391348425|ref|XP_003748448.1| PREDICTED: isotocin-neurophysin IT 1-like [Metaseiulus occidentalis]
MKLVALYFLSVSSLSWACFITNCPIGGKRSAAFGLQLYSNEYRRCPPCGPGSSGQCFGPGICCTPDYCVL
DPVGLSTCKSEALKMTPCNINRPRCGAENKGYCALNRLCCTSDSCTLDESCSSAKDRDILRESLMLN
>Tetranychus urticae gi|1005954275|ref|XP_015785540.1| PREDICTED: neurophysin 1-like [Tetranychus urticae]
MLASFILLTFILNFHSSQACFITNCPPGGKRSHGDVDHMRECLRCGPDNRGHCVSPNICCGTEFGCLINN
HYSSPCRAENLIPTPCKIPGKLCSSGEGVCTSNGICCNSDGCFNDSTCENDKLSNMQRIFTSD
>**Nilaparvata lugens** gi|411530692|gb|AFW19795.1| arginine vasopressin-like peptide [Nilaparvata lugens]
MKNTTSIGIMLFYAVGLAAGCLITNCPRGGKRGLHSYMKKLAEEKMALAQVPLQCARCGPEGEGYCMGPS
ICCGPQLGCLLASPATLNQCAGSVLPPQSPNSPAPTCYMPDGYGVCSTDGVCCNSESCRIDPTCEVDPDH
NVCNVWTEPKNMAL
>**Dinoponera quadriceps** gi|951576040|ref|XP_014485987.1| PREDICTED: neurophysin 1-like [Dinoponera quadriceps]
MLRELVVLASLIFLSYACLITNCPHGGKRGDIAPSLGVVTRECPLCGPNHLGQCFGPHICCGPSIGCFIG
TPETYRCRKESLYARPCIAGYAMCRGNTARCATNGICCSQESCHMDTSCRISDVVGNDRKMDTDLNVILS
SNEVSHEILQ
>**Monomorium pharaonis** gi|826427284|ref|XP_012527594.1| PREDICTED: neurophysin 1-like [Monomorium pharaonis]
MLKEFVVFASLIFFSYGCLITNCPRGGKRGDIVPSLGTVARECSSCGPNRLGQCFGPHICCGPSIGCFIG
TPETYRCRKESLYTRPCIAGYAMCRGNTARCALNGICCSQESCHMDTSCKISNVVGNNQKLDDNLNVILS
GNEISSEIPQ
>**Vollenhovia emeryi** gi|795056828|ref|XP_011871987.1| PREDICTED: neurophysin 1-like [Vollenhovia emeryi]
MLKEFVIFASLIFLSYACLITNCPRGGKRGDIAPSLGTVVRECPSCGPNRLGQCFGPYICCGPSIGCFIG
TPETYRCRKESLYTRPCVAGYAMCRGNTARCASSGICCSQESCHMDTSCRIPDVVGNDKKLDANLNVILP
GSEVSSEILQ
>**Linepithema humile** gi|815804199|ref|XP_012222809.1| PREDICTED: neurophysin 1-like [Linepithema humile]
MLKELIVFANLILLSYACLITNCPRGGKRGDIAPLETIARECPSCGPNHLGQCFGPHICCGPSIGCFIGT
PETYRCRKESLYTRPCIAGYAMCRGKTARCATNGICCSQESCHMDTSCRISNIVDNNRKIDTDLNMIIPG
NEVSSEILQ
>**Atta cephalotes** gi|801367097|ref|XP_012061245.1| PREDICTED: neurophysin 1-like [Atta cephalotes]
MLKELIVFASLIFLSYACLITNCPRGGKRSDVASSLRTVIRECPSCGPNHLGQCFGPYICCGPSIGCFIG
TPETFRCRKESLYTRPCIAGYAMCRGNTARCASNGICCSQAFCHMDTSCKISDVDNDRKLDDNVNVILPG
EVSNEILQ
>**Wasmannia auropunctata** gi|780704521|ref|XP_011702880.1| PREDICTED: neurophysin 1-like [Wasmannia auropunctata]
MLKELVVLMSLIFLSYACLITNCPRGGKRNNIASSLETVARECPSCGPNHQGQCFGPYICCGPSIGCFIG
TPETYRCRKESLYTRPCIAGYAMCRGNTARCASNGICCSQESCNMDTSCKISDVGNDRKLDANLNVILSG
NEVSSDLF
>**Pogonomyrmex barbatus** gi|769867110|ref|XP_011645557.1| PREDICTED: neurophysin 1-like [Pogonomyrmex barbatus]
MLKKIVVFASLIFLSYACLITNCPRGGKRSDLAPLGNVVRECPSCGPNHLGQCFGPHICCGPNIGCFIGT
SETYRCRKESLYIRPCIAGYAMCRGNTARCASNGICCSQESCYIDSSCRISNAVGSDRKLDADLNVILPG
NEVSNEMFQ
>**Cerapachys biroi** gi|759033592|ref|XP_011329433.1| PREDICTED: neurophysin 1-like [Cerapachys biroi]
MLKKLVVFATLIFLSYACLITNCPRGGKRGDIAPSLGTVARECPSCGPNHLGQCFGPHICCGPSIGCFIG
TSETYRCRKESLYTRPCVAGYAMCRGNTARCATNGICCSQESCHMDTSCRISDVGNDRKIDADLNVMFPG
NEASSETFP
>**Harpegnathos saltator** gi|749788059|ref|XP_011147500.1| PREDICTED: neurophysin 1-like [Harpegnathos saltator]
MLRELVVFASLIFLSYACLITNCPRGGKRGDIIPSLGTVTRECPPCGPNHLGQCFGPHICCGPTIGCFIG
TPETYRCRKESPYARPCIAGYAMCRGNTARCATNGICCSQDSCHMDTSCRISDVVSNDRKMDADLSAILS
SNEASHEIIQ
>**Acromyrmex echinatior** gi|746868398|ref|XP_011065328.1| PREDICTED: neurophysin 1-like [Acromyrmex echinatior]

MLKELIVFASLIFLSYACLITNCPRGGKRSDIASLKTVIRECPSCGPNHLGQCFGPYICCGPSIGCFIGT
PETFRCRKESLYTRPCIAGYAMCRGKTARCASDGICCSQASCHMDTSCKISDVGNDRNLDDNVNVILPGN
EVSNEILQ
>**Polistes canadensis** gi|954542367|ref|XP_014601007.1| PREDICTED: neurophysin 1-like [Polistes canadensis]
MTKKILIFTSFIFISSACLITNCPRGGKRGDIEVPESPIRECLPCGPSQLGQCFGPNICCGPTFGCHIGT
SQTQKCRKEQFDSSPCMAGFAMCNGNKGRCGAGGICCSQDSCFIDPSCRFSTVDSSFRRSIDSDLKAIAS
EIEILKEEAK
>**Microplitis demolitor** gi|665817708|ref|XP_008557637.1| PREDICTED: neurophysin 1 [Microplitis demolitor]
MNLPGTMIRKIFAVVFLLSTSFACLITNCPRGGKRGESPLLSMPSLIKECSSCGPNDQGQCFGPHICCGA
TIGCYIKGPETYKCRKESLYSHPCVSGFAMCRDNTARCAANGICCSQESCHVDPSCRVGDEYLSERKISQ
EFSRLIADNDQLIN
>**Trichogramma pretiosum** gi|936696156|ref|XP_014226062.1| PREDICTED: neurophysin 1-like [Trichogramma pretiosum]
MLKVIVFVFVMLFSLSHCCLITNCPRGGKRGDPTFFLENIARECPVCGHEEQGHCFGPHICCSPSMGCLI
GTSETLSCRKESLYSRPCVAGFAMCQNNNGRCAANGICCSQESCFIDPACKLVDENNDRKIGAEFGAFLL
ENSGRNEHIL
>**Copidosoma floridanum** gi|936606102|ref|XP_014216967.1| PREDICTED: neurophysin 1-like [Copidosoma floridanum]
MLKVSLVLLALVSVAYSCLITNCPRGGKRGDPTFLLENIARECPACGHEEQGRCFGPHICCGPSMGCHIG
TLETLRCRKESLYSRPCVAGFAMCQGNSGRCAANGICCSQESCFTDSACKLVDESGNDRKIGAEFGAFLL
ENAGANERILR
>**Athalia rosae** gi|817077789|ref|XP_012261240.1| PREDICTED: neurophysin 1-like [Athalia rosae]
MFQKTAIILLLVSSALGCLIINCPRGGKRNGALLPLKNPIRECPACGPEMQGQCFGPKICCGPSIGCFFG
TAETHNCRKESLYSRPCIAGFAMCRGNTGRCAAGGICCSQESCHADSNCKVTDDFNANNQGISFDLNTLF
SENTSLNDQ
>**Fopius arisanus** gi|755956374|ref|XP_011303419.1| PREDICTED: neurophysin 1-like [Fopius arisanus]
MSKIILIMSALICMSSACLITNCPRGGKRGENPLLSLHSLAAECPSCGPGHQGQCFGPNICCGTTIGCFI
GTPETYKCRTESLYSRPCIAGFAMCRDNTGRCAANGICCSQENCHADSSCRVGDEFSDRKIVQEYSRLSP
ENE
>**Nasonia vitripennis** gi|156543816|ref|XP_001606547.1| PREDICTED: neurophysin 1 [Nasonia vitripennis]
MSKVIIVLTTLVALSYGCLITNCPRGGKRGDPTFLLENIARECPACGREEQGRCFGPHICCSPSMGCLIG
TPETLRCRKESLYSRPCVAGFAMCQGNSGRCAANGICCSQESCFIDSACKLVDETGNDRKIGAEFGAFLL
ENAGTNEHIL
>**Orussus abietinus** gi|817209540|ref|XP_012280828.1| PREDICTED: uncharacterized protein LOC105699950 [Orussus abietinus]
MKELLALYVLISAGYCCLITNCPRGGKRSHHSESPETTIEKFPLAGLYDQSRYPSLHVYNDQFIDSYARS
ADTTKSATEACWENKEFLNDNGNNQRKKMKLTELFKRRRCKSKFINDTSIPSPNILI
>**Cephus cinctus** gi|1000762296|ref|XP_015604200.1| PREDICTED: neurophysin 1-like [Cephus cinctus]
MLKLCTVIMVFISISFACLITNCPRGGKRSGAITSLENIVRECPSCGADKQGQCFGPRICCGPSLGCLIG
TPETHKCRKESLFSRPCIAGYAMCRGNTGRCAANGICCTQESCYVDDTCRITDDVNNDHRIISELNGFLN
EGNVLADHNQ
>**Neodiprion lecontei** gi|998512075|ref|XP_015516596.1| PREDICTED: neurophysin 1-like [Neodiprion lecontei]
MFRKIVVILFLVSSTLGCLIINCPRGGKRDGGFNPFKYQVRECSTCGPDRQGQCFGPKICCGPSIGCFFG
TAETHKCRKESLYSRPCTAGFAMCRGNTGRCAANGICCSQESCYVDPNCKVRSDIIQMSKQVTSFDMDKI
YSESNSLNDE
>**Diachasma alloeum** gi|970901303|ref|XP_015116397.1| PREDICTED: neurophysin 1-like [Diachasma alloeum]
MSKIILIISALVCVSCACLITNCPRGGKRGENPLLSLHSLAAECPSCGPGHQGQCFGPNICCGTAIGCFI
GTPETYKCRTESLYSRPCIAGFAMCRDNTGRCAANGICCSQENCHVDSSCRISDEFNDRKIGQEYSRLSP
ENEQ
>**Polistes dominula** gi|972190475|ref|XP_015176922.1| PREDICTED: oxytocin-neurophysin 1-like [Polistes dominula]
MTKKILIFTSFIFITSACLITNCPRGGKRGDIEVPESIRECLPCGPNQLGQCFGPKICCGPTFGCHIGTS
QTQKCRKEQFDTSPCLAGFSMCNGNKGRCGAAGICCSQNSCFIDPSCRFPTFDDWPGRQKIDSDLKTIAS
EILKEEAM
>**Daphnia pulex** gi|321460843|gb|EFX71881.1| putative vasopressin-like neuropeptide preprohormone [Daphnia pulex]
MAGLWTFCLIALSMTEMIIPLTAKPCFITNCPPGGKRSSQLVEPSSYLECAPCGPAGKGTCLGANLCCGS
HFGCFFKTEETNVCLLTNLKSTQICNQHFWKTDLKSASCSLNGDKIDGICVADLLCCSLGNLPQDDL
>**Tribolium castaneum** gi|145651811|ref|NP_001078831.1| oxytocin/vasopressin-like peptide precursor [Tribolium castaneum]
MSTIITSIILLVLSESLVSGCLITNCPRGGKRSKFAISENAVKPCVSCGPGQSGQCFGPSICCGPFGCLV
GTPETLRCQREGFFHEREPCIAGSAPCRKNTGRCAFDGICCSQDSCHADKSCASDDKSPIDLYTLINYQA
ELAGDK
>**Sarcoptes scabiei** gi|934146681|gb|KPL97556.1| vasopressin-like protein [Sarcoptes scabiei]
MLFQQTNYLFRSISILVMIVLIVENVRGCFITNCPPAGKRSMLAAIGSSTQTRRECIRCGPGLSGRCYGP
SICCSPLFGCNVGGFASSRCALEAFNPMLCSNPGSACGPNGKGICAINSTCCTDSKI
>**Daphnia magna** gi|941105145|gb|JAK93241.1| Oxytocin/vasopressin peptide precursor, partial [Daphnia magna]
VKDVQSRVLFDGCLTTLPINKFKLFSSTCVKTKYIVLIMAALWTLCLIAFSILEMTMPSAAKPCFITNCP
PGGKRSGHVSEDPSSFQGVAEERPLLSHQCASCGPGGKGTCFGASLCCGSEFGCFFKTNETNICLLTNLK
SSRSCDERFWQIYFKSAPCSLNGDKLDGICVADRLCCSLGQCKQNFAC
>**Trachymyrmex_zeteki** gi|1012971628|gb|KYQ46204.1| Vasotocin-neurophysin VT [Trachymyrmex zeteki]
MLKELIVFASLIFLSYACLITNCPRGGKRSDIASSLRTVIRECPSCGPNHLGQCFGPYICCGPSIGCFIG
TPETSRCRKESLYTRPCIAGYAMCRGNTARCASNGICCSQASCHMDTSCKISDVGNDRKLDDNVNVILPG

NEVSNEILQ
>**Trachymyrmex_septentrionalis** gi|1009421053|gb|KYN39529.1| Vasotocin-neurophysin VT 1 [Trachymyrmex septentrionalis]
MLKELIVFASLIFLSYACLITNCPRGGKRSDVASSLKTVIRECPSCGPNHLGQCFGPYICCGPSIGCFIG
TPETFQCRKESLYTRPCIAGYAMCRGNTARCASNGICCSQASCHMDTSCKISDVGNDRKLDDNVNVILPG
NEVSNEILQ
>**Trachymyrmex_cornetzi** gi|1009399678|gb|KYN19822.1| Vasotocin-neurophysin VT [Trachymyrmex cornetzi]
MLKELIVFASLIFLSYACLITNCPRGGKRSDIASSFKTVIRECPSCGPNHLGQCFGPYICCGPSIGCFIG
TPETFRCRKESLYTRPCIAGYAMCRGNTARCASNGICCSQASCHMDTSCKISDVGNDRKLDDNVNVILPG
NEVSNEILQ

(C)

>Ceratosolen solmsi marchali gi|766922501|ref|XP_011505828.1| PREDICTED: isotocin-neurophysin IT 2-like [Ceratosolen solmsi marchali]
MTNYPRGGKRAMFANPTFLLDKIAREFGPHICCGPTMGCLLGTPEAHHCQKESLYSQPYVAGFAMCRGNS
GRCAANGIMNNIPKNHVSLIHHAN
>Frankliniella_occidentalis
QCTPCGPGGEGLCVGPGICCSPVFGCVLTSRGGCGRGAVFAPRCSPSSAALAAASAAAVPLDAPCGADLTPDGSAPGRCAAQGVCCTH
>Oncopeltus_fasciatus
FLLLFKCKIYFFQQAFPRCEPDREGRCLGRAICCGPKIGCLVGTPAILSQCSEQPPELPYSKPYSIGVCLSDGGLLFIG
>Calanus_finmarchicus2
PVSGKKRSLHVGEEQVRDGELTCPSNPAGLCYSPGLCCVQGGCYADGGCLPGLTERGEEQIKTRESVSKRGLRFLYGMAPSQMNLFGQEGMAIPCIGPYCN
GKGARSHGKQKQRKRKQSQEHVNRDQALLGSSNYDSAVSDDQFLKHNCDWMDLYLAR
>Prorhinotermes_simplex
QCARCGPAKLEHCYGPAICCGPQIGCLVATPETARCLTEAASPVPCIAPTGVQCGEGKLAGRCTANGVCCTHGKQSSTMYCLYVFNQAQVQL-
>Scylla_paramamosain gi|957760967|gb|ALQ28600.1| vasotocin-neurophysin, partial [Scylla paramamosain]
GRCVGPDICCGARIGCFLGSRETRMCRTENMVPITCYNSDLKPCGRMQEGRCGAPGICCTENKCEMNDDCVAEDTQGEEVEESQRGGRPRLDLLAAARDRW
EEQ
>Ceuthophilus_sp
CARCGPAKLGHCYGPAICCAPQFGCLIGTPDTDPCQREDQSPIPCIGTGSGPRCGEQGHCTANEVCCTQESCHIDPTCRLTLPAEGGLPDGPDATTDLSDR
NLVYSLFNAISTYQ
>Tenthredo_koehleri
CPACGPDRQGQCFGPKICCGPSIGCFIDTAETHKCRKESLYSRPCTAGFAMCRGNTGRCAADGICCSQESCQVDPNCKITDDSITS
>Tetrodontophora_bielanensis
IPEQHNTNKVRECASCGPVGQGGRCYGPETCCSPNFGCMVGTPETHTCLIEDRINSVCHNPGIPCTVNVDNGNINGFCATG
>Campodea_augens
GRCFSPDLCCGPEIGCYFRTKESSVCKSENLFPVPCENPAKSCGTDRNGKCAGQGLCCTEDSCTVDSSCHVILKDLPPPPPAAVAPPSPWLKYPSVFRVLQ
QQQQPNTDLSSATGALWDDMLE
>Medauroidea_extradentata
VTHFNSSPGFKHKKCPRCGPAKLGHCYGDNICCGVEIGCLLATPDTQVCQRESMTPDPCVGPIGGAPCGNSGKCAAPGVCCTPDSCTIDPTCHQTLSQEMA
SLYLYNAGNARGVSQISDVDDK-
>Blaberus_atropos
IFFLCNVDIKIMHLKYLDTKQCARCGPAKLGHCYGPAVCCGPQIGCLIATPDTARCLNEAASPIPCTA
>Periplaneta_americana
GCLIATPDTARCLSEAASPVPCIAPTGAQCGEGKSAGRCTANGVCCTHGEASQNIYNLQYSFITSTTFLLPYLLEILKYISQ
>Tetramorium_bicarinatum
GDIAPLGTIVRECPSCGPNHLGQCFGPHICCGPSIGCFIGTSETYRCRKESLYTRPCVAGYAMCRGNTARCASNGICCSQDSCYVDASCKNSDGVRNDQKF
DANLNEVLPGNEFSREIM-
>Inocellia_crassicornis
FGCLVGTPETIRCERDGAFQEREPCIAGRSNCRGDTGRCAADGICCTQGNYE-
>Osmylus_fulvicephalus
SVYFFGKSMYTLQCISCGPQRSGQCFGPAICCGPFGCLIATAETTRCERDGAFHEREPCIAGKSSCRGDTGRCAFDGICCTQGMCTVQLKLGLKSLPNAIR
LI-
>Lepicerus_sp
VNCPRGGKRGELPFLSIQGLVKECQSCGPGHQGQCFGPNICCGTNIGCFIGTPETYKCKMESLYSRPCIAGFSMCRDNNGRC
>Aethina_tumida
CVSCGPGHTGQCFGPSICCGPFGCLMGTSETIRCQREGFFQEREPCIAGSSFCRKNTGRCAIDGICCNQESCHVDKHCSLDEKKSSENLMGMEL
>Pogonus_chalceus
CIACGPGHSGQCFGPNICCGPFGCLIGTSETLKCQREGFFQEPEPCIAGNGYCRKNTGRCAADGVCCTQ
>**Dastarcus_helophoroides**
QCIPCGPARTGQCFGPNICCGPFGCLVGTPDTIKCQREGFFHERDPCIAGNSSCRKNTGRCAADGICCS
>Diabrotica_virgifera_virgifera
GQCFGPNICCGPFGCLLGSPETVRCQREGSFHEREPCIAGSGFCRKNTGRCATDNICCDQDSCYTDRQCSSDEKLKAEALSGLDIYNFLNTY
>Baetis_sp
VAPAATASNLALLPCVAESLFPEPCSNGPTVRCGRGAKGVCAAPGVCCFHNSCETDASCAKMRRVNAIARESNAFA
>Isonychia_bicolor
ACLQCARCGFGLAGRCFGPRICCDTRLGCMVGSAMEPLQAELLPCLAESIFPAACHNHAATCGRMATGQCAATGLCCYQDSCHPDMTCRGRSE

(D)

Atta colombica contig16192, whole genome shotgun sequence
Sequence ID: gb|LKEW01016192.1|Length: 13960
Number of Matches: 2
Range 1: 8764 to 8997GenBankGraphics
Query: Polistes dominula gi|972190475|ref|XP_015176922.1|

Alignment statistics for match #1

Score	Expect	Method	Identities	Positives	Gaps	Frame
96.3 bits(238)	6e-23	Compositional matrix adjust.	47/78(60%)	56/78(71%)	4/78(5%)	-2

```
Query  41    CLPCGPNQLGQCFGPKICCGPTFGCHIGTSQTQKCRKEQFDTSPCLAGFSMCNGNKGRCG  100
             C  CGPN LGQCFGP ICCGP+ GC IGT +T +CRKE   T PC+AG++MC GN  RC
Sbjct  8997  CPSCGPNHLGQCFGPYICCGPSIGCFIGTPETFRCRKESLYTRPCIAGYAMCRGNTARCA  8818

Query  101   AAGICCSQ----NSCFID  114
             + GICCSQ    NSC ++
Sbjct  8817  SNGICCSQGKLKNSCKLE  8764
```

Range 2: 9826 to 9873GenBankGraphics

Alignment statistics for match #2

Score	Expect	Method	Identities	Positives	Gaps	Frame
37.0 bits(84)	0.025	Composition-based stats.	14/16(88%)	15/16(93%)	0/16(0%)	-2

```
Query  17    ACLITNCPRGGKRGDI  32
             ACLITNCPRGGKR D+
Sbjct  9873  ACLITNCPRGGKRSDV  9826
```

Lasius niger Lnig_2.1_6499, whole genome shotgun sequence
Sequence ID: gb|LBMM01006435.1|Length: 10190
Number of Matches: 3
Range 1: 5184 to 5393GenBankGraphics
Query: Atta cephalotes gi|801367097|ref|XP_012061245.1|

Alignment statistics for match #1

Score	Expect	Method	Identities	Positives	Gaps	Frame
133 bits(335)	4e-36	Compositional matrix adjust.	60/70(86%)	67/70(95%)	0/70(0%)	+3

```
Query  42    ECPSCGPNHLGQCFGPYICCGPSIGCFIGTPETFRCRKESLYTRPCIAGYAMCRGNTARC  101
             +CPSCGPNHLGQCFGP+ICCG +IGCFIGTPET+ CRKESLYT+PC+AGYAMCRGNTARC
Sbjct  5184  QCPSCGPNHLGQCFGPHICCGANIGCFIGTPETYHCRKESLYTKPCVAGYAMCRGNTARC  5363

Query  102   ASNGICCSQA  111
             A+NGICCSQ
Sbjct  5364  ATNGICCSQG  5393
```

Range 2: 4298 to 4423GenBankGraphics

Alignment statistics for match #2

Score	Expect	Method	Identities	Positives	Gaps	Frame
74.7 bits(182)	2e-15	Compositional matrix adjust.	34/42(81%)	39/42(92%)	0/42(0%)	+2

```
Query  1     MLKELIVFASLIFLSYACLITNCPRGGKRSDVASSLRTVIRE  42
             MLK+L++FASLIFLSYACLITNCPRGGKRSD+A SL T +RE
Sbjct  4298  MLKKLVIFASLIFLSYACLITNCPRGGKRSDIAPSLTTALRE  4423
```

Range 3: 5551 to 5664GenBankGraphics
Alignment statistics for match #3

Score	Expect	Method	Identities	Positives	Gaps	Frame
45.8 bits(107)	2e-05	Compositional matrix adjust.	24/38(63%)	33/38(86%)	2/38(5%)	+1

```
Query  113   CHMDTSCKISDV-DNDRKLDDNVNVILPG-EVSNEILQ  148
             C++D+SCKISDV  NDRK+D ++N+I PG EVS+E+LQ
Sbjct  5551  CYIDSSCKISDVVANDRKIDADLNMIFPGNEVSSELLQ  5664
```

Cyphomyrmex costatus contig18246, whole genome shotgun sequence
Sequence ID: gb|LKEX01018246.1|Length: 14919
Number of Matches: 1
Range 1: 12261 to 12491GenBankGraphics
Query: Atta cephalotes gi|801367097|ref|XP_012061245.1|

Alignment statistics for match #1

Score	Expect	Method	Identities	Positives	Gaps	Frame
136 bits(343)	4e-37	Compositional matrix adjust.	66/79(84%)	71/79(89%)	2/79(2%)	-2

```
Query  43     CPSCGPNHLGQCFGPYICCGPSIGCFIGTPETFRCRKESLYTRPCIAGYAMCRGNTARCA  102
              CPSCGPNHLGQCFGPYICCGPSIGCFIGTPETF+CRKE+L+TRPCIAGYAMCR NTARCA
Sbjct  12491  CPSCGPNHLGQCFGPYICCGPSIGCFIGTPETFQCRKENLHTRPCIAGYAMCRRNTARCA  12312

Query  103    SNGICCSQAFCHMDTSCKI  121
              SNGICCSQ    +  SC+
Sbjct  12311  SNGICCSQG--KLKNSCRF  12261
```

Cyphomyrmex costatus contig15288, whole genome shotgun sequence
Sequence ID: gb|LKEX01015288.1|Length: 17512
Number of Matches: 1
Range 1: 12730 to 12858GenBankGraphics
Query: Atta cephalotes gi|801367097|ref|XP_012061245.1|

Alignment statistics for match #1

Score	Expect	Method	Identities	Positives	Gaps	Frame
75.1 bits(183)	1e-15	Compositional matrix adjust.	37/43(86%)	38/43(88%)	0/43(0%)	+1

```
Query  1      MLKELIVFASLIFLSYACLITNCPRGGKRSDVASSLRTVIREC  43
              MLKE IVFASLIFLS ACLITNCPRGGKRSD+ SSLRT IRE
Sbjct  12730  MLKEFIVFASLIFLSSACLITNCPRGGKRSDIISSLRTDIREV  12858
```

Nasonia longicornis strain IV7(U) Contig18019, whole genome shotgun sequence
Sequence ID: gb|ADAP01018019.1|Length: 7333
Number of Matches: 2
Range 1: 6887 to 7273GenBankGraphics
Query: Monomorium pharaonis gi|826427284|ref|XP_012527594.1|

Alignment statistics for match #1

Score	Expect	Method	Identities	Positives	Gaps	Frame
126 bits(316)	6e-39	Compositional matrix adjust.	62/129(48%)	81/129(62%)	23/129(17%)	+2

```
Query  42    ECSSCGPNRLGQCFGPHICCGPSIGCFIGTPETYRCRKESLYTRPCIAGYAMCRGNTARC  101
             +C +CG    G+CFGPHICC PS+GC IGTPET RCRKESLY+RPC+AG+AMC+GN+ RC
Sbjct  6887  QCPACGREEQGRCFGPHICCSPSMGCLIGTPETLRCRKESLYSRPCVAGFAMCQGNSGRC  7066

Query  102   ALNGICCSQ-----------------------ESCHMDTSCKISNVVGNNQKLDDNLNVI  138
             A NGICCSQ                       ESC +D++CK+ +  GN++K+
Sbjct  7067  AANGICCSQGKTSVINMFVKQEKYF*FP*IFLESCFIDSACKLVDETGNDRKIGAEFGAF  7246

Query  139   LSGNEISSE  147
             L  N  ++E
Sbjct  7247  LLENAGTNE  7273
```

Range 2: 6694 to 6825GenBankGraphics

Alignment statistics for match #2

Score	Expect	Method	Identities	Positives	Gaps	Frame
54.3 bits(129)	6e-39	Compositional matrix adjust.	26/44(59%)	30/44(68%)	0/44(0%)	+1

```
Query  1     MLKEFVVFASLIFFSYGCLITNCPRGGKRGDIVPSLGTVARECS  44
             M K  +V  +L+  SYGCLITNCPRGGKRGD    L  +ARE S
Sbjct  6694  MSKVIIVLTTLVSLSYGCLITNCPRGGKRGDPTFLLENIAREVS  6825
```

Steganacarus magnus, whole genome shotgun sequence
Sequence ID: gb|LBFN01029022.1|Length: 1266
Number of Matches: 1
Range 1: 516 to 923GenBankGraphics
Query: Ixodes scapularis gi|506953581|gb|AGM20426.1|

Alignment statistics for match #1

Score	Expect	Method	Identities	Positives	Gaps	Frame
68.6 bits(166)	4e-13	Compositional matrix adjust.	49/137(36%)	67/137(48%)	26/137(18%)	-2

```
Query  1    MALSHMLLLAVVGG-TSACFITNCPPGGKRSSEPSPARL---------------------  38
            + LS + L+A++ G T+ACFITNCPPGGKRS+
Sbjct  923  LNLSILTLIALIFGLTTACFITNCPPGGKRSAFIKNNNQKQVI*KTNGFLIRNYNRCVI*  744

Query  39   -CPRCGPAGRGVCYSADVCCAGSM-CILNDALATLSCRAEALHGVACHVPGKRCGT-DGR  95
             C RCGP G G C+ AD+CC  ++ C    + L  + C  E  +    C + GK C   +G
Sbjct  743  *CSRCGPFGVGRCFGADICCGATIGCHFKNRLTEV-CLTENYNPHPCKIDGKSCDNGNGV  567

Query  96   CAIRGYCCGPDGCTKDS  112
            CA+   CC P  CT +S
Sbjct  566  CALNSTCCTPGKCTLNS  516
```

Hypochthonius rufulus scaffold114652_cov135, whole genome shotgun sequence
Sequence ID: gb|LBFL01028909.1|Length: 27086
Number of Matches: 2
Range 1: 2097 to 2183GenBankGraphics
Query: Ixodes scapularis gi|506953581|gb|AGM20426.1|

Alignment statistics for match #1

Score	Expect	Method	Identities	Positives	Gaps	Frame
43.5 bits(101)	7e-11	Composition-based stats.	20/29(69%)	25/29(86%)	1/29(3%)	-1

```
Query  3     LSHMLLLAVVGG-TSACFITNCPPGGKRS  30
             +S + +LAV+ G T+ACFITNCPPGGKRS
Sbjct  2183  MSILTILAVIFGITTACFITNCPPGGKRS  2097
```

Range 2: 1763 to 1984GenBankGraphics

Alignment statistics for match #2

Score	Expect	Method	Identities	Positives	Gaps	Frame
42.4 bits(98)	7e-11	Compositional matrix adjust.	26/75(35%)	36/75(48%)	3/75(4%)	-2

```
Query  39    CPRCGPAGRGVCYSADVCCAGSM-CILNDALATLSCRAEALHGVACHVPGKRCGT-DGRC  96
             C RCGP+G G C+  D+CC   + C   + + T+ CR E +    C   G+ C    G C
Sbjct  1984  CTRCGPSGLGRCFGPDICCGEEIGCHFKNQITTI-CRTENISPRLCRNEGRTCNNGKGIC  1808

Query  97    AIRGYCCGPDGCTKD  111
             AI   CC    C  +
Sbjct  1807  AIDSVCCSTSKCHNN  1763
```

Platynothrus peltifer, whole genome shotgun sequence
Sequence ID: gb|LBFO01089471.1|Length: 3080
Number of Matches: 2
Range 1: 990 to 1037GenBankGraphics
Query: Ixodes scapularis gi|506953581|gb|AGM20426.1|

Alignment statistics for match #1

Score	Expect	Method	Identities	Positives	Gaps	Frame
39.7 bits(91)	3e-08	Composition-based stats.	15/16(94%)	16/16(100%)	0/16(0%)	-1

```
Query  15    TSACFITNCPPGGKRS  30
             T+ACFITNCPPGGKRS
Sbjct  1037  TTACFITNCPPGGKRS  990
```

Range 2: 674 to 880GenBankGraphics

Alignment statistics for match #2

Score	Expect	Method	Identities	Positives	Gaps	Frame
37.7 bits(86)	3e-08	Compositional matrix adjust.	25/69(36%)	31/69(44%)	1/69(1%)	-2

```
Query  36   ARLCPRCGPAGRGVCYSADVCCAGSMCILNDALATLSCRAEALHGVACHVPGKRCGT-DG  94
            A+ C RCGPAG G C+  D+CC  ++        T  C  E      C   GK C    G
Sbjct  880  AKQCIRCGPAGIGRCFGPDICCGEAIGCHFKNRFTAVCSTEDYSPHPCQNDGKTCANGKG  701

Query  95   RCAIRGYCC  103
             CA+   CC
Sbjct  700  VCALDSTCC  674
```

Achipteria coleoptrata scaffold1020_cov175, whole genome shotgun sequence
Sequence ID: gb|LBFM01000221.1|Length: 64589
Number of Matches: 2
Range 1: 14022 to 14162GenBankGraphics
Query: Ixodes scapularis gi|506953581|gb|AGM20426.1|

Alignment statistics for match #1

Score	Expect	Method	Identities	Positives	Gaps	Frame
44.3 bits(103)	1e-04	Compositional matrix adjust.	28/55(51%)	34/55(61%)	8/55(14%)	+3

```
Query  1      MALSHMLLLAVVGGTSACFITNCPPGGKRSSEPSPARLCPRCGPAGRGVCYSADV  55
              M LS M L+ V+  TSACFITNCPPGGKR S+PS      R GP  + +    D+
Sbjct  14022  MILSIMTLIVVL--TSACFITNCPPGGKR-SDPS-----LRDGPKKQVILLFNDL  14162
```

Range 2: 14197 to 14394GenBankGraphics

Alignment statistics for match #2

Score	Expect	Method	Identities	Positives	Gaps	Frame
41.6 bits(96)	0.001	Compositional matrix adjust.	28/67(42%)	34/67(50%)	3/67(4%)	+1

```
Query  39     CPRCGPAGRGVCYSADVCCAGSM-CILNDALATLSCRAEALHGVACHVPGKRCGT-DGRC  96
              C RCGP+G G C+  D+CC  S+ C LN+   T  C  E      CH  GK C    G C
Sbjct  14197  CTRCGPSGIGHCFGPDICCGPSIGCHLNNRF-TAVCSTEDYSPHPCHNEGKLCANGKGIC  14373

Query  97     AIRGYCC  103
              A+   CC
Sbjct  14374  ALESTCC  14394
```

Rhipicephalus microplus strain Deutsch Ctg100269, whole genome shotgun sequence
Sequence ID: gb|ADMZ02000298.1|Length: 711
Number of Matches: 1
Range 1: 221 to 313GenBankGraphics
Query: Metaseiulus occidentalis gi|391348425|ref|XP_003748448.1|

Alignment statistics for match #1

Score	Expect	Method	Identities	Positives	Gaps	Frame
33.1 bits(74)	2.3	Compositional matrix adjust.	15/31(48%)	20/31(64%)	0/31(0%)	+2

```
Query  1    MKLVALYFLSVSSLSWACFITNCPIGGKRSA  31
            M  +     L +  ++ ACFITNCP GGKRS+
Sbjct  221  MTPLNFLMLGLVGMTSACFITNCPPGGKRSS  313
```

Centruroides exilicauda breed non domesticated species of scorpion Contig13139, whole genome shotgun sequence
Sequence ID: gb|AXZI01013139.1|Length: 1479
Number of Matches: 2
Range 1: 1049 to 1120GenBankGraphics
Query: Limulus polyphemus gi|926609726|ref|XP_013784878.1|

Alignment statistics for match #1

Score	Expect	Method	Identities	Positives	Gaps	Frame
36.6 bits(83)	0.17	Compositional matrix adjust.	16/24(67%)	17/24(70%)	0/24(0%)	+2

```
Query  14    LAVTIKGCFITNCPPGGKRSIGTF  37
             L V    CFITNCPPGGKRS+ T
Sbjct  1049  LIVLTSACFITNCPPGGKRSLHTI  1120
```

Range 2: 1323 to 1466GenBankGraphics
Alignment statistics for match #2

Score	Expect	Method	Identities	Positives	Gaps	Frame
31.6 bits(70)	8.8	Compositional matrix adjust.	19/51(37%)	24/51(47%)	3/51(5%)	+3

```
Query  61    ICCSDIFGCFIKTRESVICRYENLQVIPCNKKGKICETVPHGYCAAPGICC  111
             +CC    GC I       IC+ ++      C K G+IC    HG C   GICC
Sbjct  1323  LCCGSTVGCIINEEGIPICQ-QSFDYHHCRKNGRICRL--HGICTTNGICC  1466
```

Mesobuthus martensii Contig352499, whole genome shotgun sequence
Sequence ID: gb|AYEL01091269.1|Length: 16655
Number of Matches: 1
Range 1: 9791 to 9898GenBankGraphics
Query: Limulus polyphemus gi|926609726|ref|XP_013784878.1|

Alignment statistics for match #1

Score	Expect	Method	Identities	Positives	Gaps	Frame
36.6 bits(83)	0.19	Compositional matrix adjust.	18/36(50%)	23/36(63%)	5/36(13%)	+2

```
Query  7     IMILGFCLA-----VTIKGCFITNCPPGGKRSIGTF  37
             +M++ F L+      V    CFITNCPPGGKRS+ T
Sbjct  9791  LMVMHFPLSFATLIVLTNACFITNCPPGGKRSLHTI  9898
```

Supplementary Data S2. Putative inotocin receptors. Sequences of 120 novel and 33 previously annotated inotocin receptor sequences from arthropods. (A) 92 newly annotated putative inotocin receptor sequences. (B) 33 deposited inotocin receptor sequences derived from NCBI. (C) 28 non-annotated inotocin receptors are presented as BLAST search results. Conserved motifs (XPQX_2W$X_{5\text{-}6}$F at the end of transmembrane domain 2 and the beginning of extracellular loop 1, and C*X*NPW in transmembrane domain 7) are highlighted in yellow; see also Supplementary Figure S6. Sequences from (A) and (B) were use to prepare the alignment illustrated in Supplementary Figure S3.

(A)

```
>Catajapyx_aquilonaris
LRDNVSGLVEQDSRDETLAAVEIATLAIILLVAILGNMFMVVGIWRQLQFRPMSRMYFFMLHLSVADLLVAVFNILPQLLWDITYRFQGSDALCRTVKYFQ
VMVLYLSSYILMFMAVDRYKALCCDAFSPWNSLLAAKIMVASAWVMAFGFATPQAVIFTLKKISPGVQDCWGTFVEPWGTKAYVTWFVISIFVMPLLVISS
SYGAISYRIWTYNSEPRCWTASKWKWLDLKKCLCCTRKESGRQSPSSVEDNDVLEQAESSPLPQKATQLELAGRLKSKVGCNVKQKRKSMISDAKIKTLQL
TLAVVICFFVCWAPFCIAQLILVYNPPEDRDVDPVTVILLLLASLNSCTNPWIYLTFSGSLLNQLRV
>Blattella_germanica
MENASQAVNGSTTAEDLDRDEVLARAEIATLSVMFTVTVVGNFAVLLVLSARPRKKLSRMYYFILHLSIADLCTAFLSILPQLAWDVTYRFRGGPIMCKVV
KYGQTLGPYLSAYILMVTALDRYQAVCHPLAYCSWTSRRSKFMVWTAWVFALGFCVPQ
>Parhyale_hawaiensis
MLDEPLPTMSEELVLNVSVNASEDAINFDILSVQNNTSFGSRDEVLAAFEVLTLASIFVVTIVGNCIVIFKHFMRRKKFRRMNYFIFALSLSD
LVTAMFNVLPQLIWDVTFRFKGGNVLCKCVKFGQLVGPYLNAYILVVTAIDRYQVICRPLSNCRWRPGHSIRLIGAATFLSLWLSVPQLFIFSLRSKNGIQ
DCWASFIQPWGVVAYVTFYSIAVFFVPLILLVFFSVCIIATLLRHNFQVPIQCNYIRQSYSTTSVHCLHHSPHKVYHHRGNSMNLSCPTVYRSKPIGSLPH
CSLHSRSLSEKAFFGQKMKQNSLSMKKQRVSRIMSPISRAKIKTVKLTLIVIFFFVACSAPFISVQLWSVWDPNAKNSPFSSGE-
>Frankliniella_occidentalis
APQVEVAVLGVLLVLTVLGNAVVLLALYARRRCGGRRKLSRMFYFILHLSVADMVTAFFSVLPQLVWVTFRFHGGNFLCKAVKFGQPLGVYLSSYVLTATA
CDRYRAICHPLTYCSWTSRRAKTMVWAAWGVAICFSAPQVRSEGYTAGQRQTLEANSVPCVNCCNLFI
>Ephemera_danica
IATLAIIFGVTVLGNSCVLAAISFRRAKISRMYFYIMHLSVADLITAFFNVLPQLAWEITYRYCLSQLCRGVKFSQLLGPYLSSFVLVLTAVDRYEAICRP
LTYCTWPARRAWFLVAGAWTLALLCCAPQLFVFSYRPVQQQPDVFDCWATFEPGWGERAYVSWYATSVFLVPLMVLVVAYFRICQALWRNYNLKQRAQQNG
LLQPNFHFYPRIPMEPRTHSLRGISRAKLRSIKLTIVVIACYVICSTPFIGAQLWATWDPEAMQSPFWSGPTFTIVTLLASLNSCVNPWIYLAFNPELARL
LITRGRV
>Ladona_fulva
MATLAAMLVISIVGNGAVMVALWARPRSSPRRRLSRMHLLVLHLCTADLITAAFTVLPQMAWEVTYRFAPQAAPLCKPLKVLQLLGPYLSSYLLVAMAIDR
HRAVCRPLSVRAFLPRSPRRLVAIAWALSALFSLPQAFVFSYQKATETEWCWATFDPPWTQKAYVTWYGISVFAVPLVLLLYAYAGICRALRRNHREKLRP
QEDGLIPRGAPPRTHSMGTISRAKMRTLRLTIVAVACYILCSCPFVLSQLWATWYPGATESSFWRGAAFTILSLLPCLNSCVNPWIYMAFNEEIRVALRER
LPGRVRDAVRSLFRFNTSKRLRHNDAITYASSSSRTRSSRESPQMEIKTLNSPVKDPPSVKKNGVVAEPKTEK
>Platynothrus_peltifer3
MTGNGSLTVKRDEKWVLLEIGILSLIFILIVFGNSCVLIALQVRKLKMNRMYYFLLHLCIADLITAFFNVIPQLAWEITHRFYGGNFLCKTIKYLQILGPY
LSSYVLVMTAIDRYQAICYPLSNCAWTPSRSKLMILMAWIVSLLCCAPQIIIFSYQEIGSQIFDCWGTFSQPYGERIYVTWYAVSVFIIPFIILTFTHVHI
CREIWLNLHKKRKSFKINRNKSSSSCNNRNDKLDCDSVNSPETSEHRSGTRLLVTVGKSYRFKGKGQVEVSVEKDCGKEDYNSPRTHSIHGLTRAKIKTVK
ITIVIIMCYIFCSTPFICVQLWAYWWPNAQQSPIWNVKYTYLSNNFA
>Platynothrus_peltifer2
MEVSTAISEETDGIPFETSSIKPNITGRNENLALIEVGTLGTIFVLIVFGNCCVLLALALRRLKMTRMYYYLLHLCISDLMTGFFTVLPQLAWDITYRFNG
GNILCKAVKSMQILGPYLSSYVLVVTAIDRYQAICFPLSNCSWTPRRAKLMISCAWIISIVCSLPQAFIFSYQEVPETNHIYDCWGTFPQPWGERIYVTWY
AISVFFIPLVILTFTHIYICREIWVNLNLKRQTGRRETQTINQNIDNSNSYSRTRKCLLIALNRVYRLGRRQDTETAEIQINCDNQEVRPLHTVVTFQESV
SSSPRVNTFNRLSRAKIKTVKITVVVILCYVICSSPFICVQLWAYWWPNASTTTFWNGMFSLFII
>Platynothrus_peltifer1
MEEIGTTVTLEELEDKLPETIIANVTKDSRDEHLAVFEIATLGLIFLLIVFGNSCVLIALIMRKLKMNRMYYFLLHLCIADLITAFFNVLPQLAWDITYRF
YGGNILCKTIKYLQILGPYLSSYVLVMTSIDRYQAICYPLSNCAWTPKRSQLMISFAWIIALLCCIPQVIIFSYQQIPGYDIIDCWGTFPQPFGERIYVSW
YAISFFFVPFILLTFTHVCICREIWRNVHQKRKSVKMEQNKIIQNDDSDRVSSCSSTNDSIARSTNLLVTVGRSYRFKGRGRVEVSVDGPKSRGTYNPRSH
SLNGLSRAKIKTVKITVVVILCYIICSSPFICVQLWAYWWPNAQNSTFWSGKCSENSFIIQFRYSFQ
>Platynothrus_peltifer4
MSNVIMSINNTTSVLYNISGSDKRDENLAIMEVSILALIFILIIIGNLCVLIALAVKRFKMTRMYYFLLHLCISDIITGCFTVMPQLAWDITHRFNGGNIL
CKLVKCLQLFGPYLSSYVLVVTAIDRYQAICFPLTNCHWTSRKSKLMITIAWIISILCSAPQAFIFSYQEIPRTNGVRDCWGTFPQPYGERIYVTWYAITV
FFIPLIIITYTYVVYICREVWANVRRKRQTLKPEISLIEVRKNWGKLSKSSDDKSRLQSDRCLANNLLNPRSHSIYRISKAKIKTVKVTVVVVICYIVCSSP
FICVQVWAYWYPSAQETSFWTGKH
>Platynothrus_peltifer5
METVSDLITFSREQEFDFELLNDSNNVNKSSFEIRDSRNESLAIIEISLLTFMFISILIGNIFVLTALIVRDSKMNRMYYFLFHLCIADLITAFFNVLPQL
IWDITYRFYGGNVLCKLIKYLQILGPYLSSYILVITAIDRYKAICFPLSNCSWTPKRSQLMISIAWVLSLIFCAPQLFIFSYQQIPGTLGNVYDCWGIFPQ
PYGERIYVTWYAITVFVIPCIIILFAHVCICREICNNLHRKRESIEEIKNCIHYSFKRNKEFSTSSLKSTSLTSFSGPIKSPITYSIRPSKRLRRIHSQID
LKKDLRNSLTNLSVKVNDLNSTPRSHSLRFVSRAKIKTVKITIVIMLCYVICSMPFISVQLWVYWFPSAQSFFQTSELSNIY-
>Achipteria_coleoptrata2
MSSDSSDMSVENISQTIIFQIINDDNMTANTSLAVKRNEKLVLLEIGILLLIFLLIVFGNSCVLIALQINKLKMNRMYYFLQHLSISDMITAFFNVLPQLA
WEITHRFYGGNILCKTIKYLQIFGPYLSSYVLVMTAIDRYQAICNPLSNCKWTPGRSKLMITIAWIVTSFCCSPQIFIFSYQQIPGTDAIYDCWGTFQKPW
GERIYVTWYAVSVFIIPFIIIVVTHYHICREIWLNLHKKRKSFKLNKNRVGKSYRFKSRGKLEVSVDKDFKEDFSPRTHSIHGLSRAKIKTVKITVVIILC
YIMCSTPFICVQLWAYWVPTAQKSHIWNVKYTYNIYLMFFSIIDVTIAFKILYSE
>Achipteria_coleoptrata1
ENITEELKNNTTVSDTRDEHLAIFEIATLSLLFVLILFGNSCVLLALLMRKIKMNRMYYFLLHLIIADYLVAFFNVLPQLIWDITYRFYGGNLLCKVIKYL
QILGPYLSSYVLVMTSIDRYQAICYPLTNCQWTPTRSQLMITLAWIIALLCCIPQVLIFSYQQIPGTDIYDCWGTFPLQPYGERIYVTWYAISFFFIPFIL
LTATHVCICREIW
```

>Varroa_destructor1
MAGNIVVLVLVLQSKPKSAHLSRIYYFLLHLSIADILVGIFNISPQLVWDIYFRFPLGNFACKIVKFLQVFVLYLSTYVLAGMAVDRYLAIRSGINRPIVV
VRTILSVSWLVAAVLASPQLYIFSFQKLPNGAHDCWATFEPPITSFRYVLFFITAVLFIPVTLMALCYTYLSWIISKRSLSHTKLNPIRMTMVMVIVFVLC
WTPFCCAQLYLESTGQVPSIFITLFLLVPNLNSCANPWVCLTFSTTLRRKLTDALSFFGFCEHLSVYRSKNAITISILQYRPPNRSKRQPRAYRGA
>Varroa_destructor2
EARRSSSLSRVQLFMMHLSIADILVALLNILPQLAWDITARFYGGALLCKFVKYAQVLVLYLSTYILTGMSLDRLVSM
>Ornithodoros_turicata
SYVLVVTAVDRYQAICFPLSNCSWTPTKSKLMIAFAWAVSLLCCVPQLFIFSYQQVENGVYDCWGTYAEPWGVTAYVTWYGVSVFFVPLAVLTFTYIYICR
AIWHNLYLKRKSSEGSRDKELSSRRTYRFAGRQRKMSSANGSFVAPRSHSVRGLSRAKIKTVKITVVVIVFYVVCSSPFICVQMWMYWSPDAESSELWSSS
TVTILMLLNSLNSCVNPWIYLAFNKNLLNALRTACCLAKVSGRGCSYLARDTTDGTNDSNAHSVVSHCEGDHARIKCSPSKRRNGFRSSTDVEMRTAQDCG
ADT
>Panonychus_ulmi
RDENLAIIEIITLSAIFVMIVFGNTSVLLALILSRIKLRRMYFFLLHLSIADLITAFTNVLPQLIWEITEHFYGGQILCKGIKYLQLLGPYLSSFTLCATA
IDRYRAICYPLESRSRLNRSNLKVSIAWGLALICCLPQLLIFSYHQIPNNPQVYECWGTFIQPYGERLYVTWYAFSSFFIPFTIIIFTFSNICSELWKN
>Hyalella_azteca
SDLVTAAFNVFPQLMWDITFRFRGGDFLCRSVKFGQLVGPYMNSYILVVTAIDRYQVICQPLSNCRWTPGHSIRLISCATAISLLFSIPQLFNFSFREKNG
IYDCWADFIPGWGVTAYVTWYAIAVFFIPLVLLIFFSGCICCALLRYNFQVPNQSSYVSRSHSQAGQESPNNETTSRKYGRTLDDTTHGSSSTVNDVNYGN
AVRILSYSSATNKARNTVIEHGCRYRFQKTPGAKTVMSPISRAKIKTIKLTMIVIFFFIVCSAPFITSLMWSVWDP
>Lepeophtheirus_salmonis
VSYNTSLNTTIPSSSPNSLLHTKLWVLGIIIVLTILGNSIVILAIATRFIKMTRMYFFILHLSIADVLTAFLTLLPELIWTYTSPNFYGGAFLCKAVKFGQ
MIGPYLSSYVLIMTAIDRYHAICFPFSKCTWTPKRSNIMIGLAWLVSLALCVPQIIIFGSGEKSHSCSATFAQDWGQKAYVTWFAVSNFFLPLIILIYCYG
CICKCIWDNFNSKIPLNPGVEVRSITCCKNKGRAEEGTIRYRWKSTKRALGSTAEYSKRNDTIEEVVMNNLTLSVSNDNQDNPNQIQAASSFTFDRCGKLR
KSKNKVLIDLDSPTQTKNKKSLKPRTHSIQGISRAKIKTIKLTVVVIAGYIACSAPFICVQLWVTFGTVTESIRK-
>Triops_cancriformis
SARDEKLAIAEIATLAVIFLLALSSNVFMLVAIWRQRQYRPLSRMYFFMLHLSLADVLVAVFNILPQLLWDITYRFLGGDLLCRFVKYGQIMPLYLSTYLL
MFMALDRYRAVCSASNNLHWNSLKVAKMMVTSAWVLSLLLAIPQAIIFSEQYINPDGTTTETDCWVRFIEPWGAKAYVTWFVISIFALPLLIIGISYGAIS
LRLWRYSQEAHRSEEMTRGHKRTIGLPAGAAGAALRPTSQRWRRASRLLGRFFLQRRERATHQRNSCGDLEEDFPATTGHVLVESQSSSSGSKPVAESLPL
RESDTASSTSKSSSHGKKIKMEPFPLRRSNSHRITRAKIKTIQLTLLVIISFVVCWAPFCITQLVLVYDPPSN
>Caligus_rogercresseyi
EGNSLHQTKLWVLGIIIVLTILGNSIVILAIATRHIKMTRMYFFILHLSIADVLTAFLTLLPELIWTYTSPNFYGGGALCKIVKFCQMIGPYLSSYVLIMT
AIDRYHAICNPLSKCTWTPKRSNIMIGLAWLVSFALCIPQAIIFGSSEAKYSCSASFVVDWGQKAYVTWFAVSNFFFPLLILIYCYGRICKTIWDNFNSKV
PMSSDAGGVTCCGNRGRAEEGMIRYRWKSSKRSRNNQNEVHQNTEENMMMDNFSLSVNQGTRDNPNQIKNATSFTFDTFSHVQKGNKRVIIDLSSPVQKQK
RKSLKPRSHSIQGISRAKIKTIKLTVVVIVGYIACSAPFICVQL
>Pachypsylla_venusta
MDESNGTLVNYNDTDSDERDEDLARVEILTLLIILVITIVGNIMVLFALYSRRHFQKQIKMPRMYYFILHLSIADLITAFFNVLPQLWWDINYRFPGSSNW
LCKLVKFVQPSGSFLSSYILMAIAIDRYRAICQPLTYHSWNSRRSRIMILIAWSAAFTFCIPQVQISIFSYQEIKPGVYDCWASWDQEWGERIYITWFSVS
VFIIPLICLIYTYTCICLALKNTAYPAPSPHKIISRAKINTVKQTITVITLYIICSLPFIFAQLWETWNLNQTVYPVFQDSALTIIMLLSSLNSCVNPWIY
LVSNRELITALWTLCFNINVKEENRTVDTASGNSSSARECCRRTDSNVTMGTYLNRQSCNDDTYVSEDNLIVSRPRQWVVSVPDG
>Homalodisca_vitripennis
MANDLVFNSSLASDLERDEELARAEIAVLAVIFVVTVTGNTAVLVALYARRFCTGNKKLPRMYFFILHLCVADLTTGLLNVFPQLCWKITYRFKGGPMLCK
VVKYGQTMGPYLSSYILVATALDRYKAICEPLNYSNWTSRRSALTVSAAWSLTLLFCIPQVSQVIIFSYQEIAPGVYDCWATFPFPWGERVYVTWYSVSVF
LVPLFVLIYTYFCICWTLWRYSGLPDSSSPHHLISRAKINTIKQTVAVILLYISCSSPFICAQLWASWGPRQPFFDGAAFTILSLLSSLNSCVNPWIYLSF
NRELLRSLWRASICTGSPRHRNLRGSASSCR
>Acerentomon_sp
MANQSLNVGDPWINETQVAEHVLNVGDPWTNETQEAEDGFKYERNEDLARIEIAILAAIFCATVIGNVLVLVALFNTKKKMTRMYYFMMHLSIADLFTAFF
NVLTQLIWEITYRFAGGNYLCKFVKYMQIFGPYLSSYILTVTALDRYRAICFPLQNSRWTERRYKAMIAVAWTATLCLCSPQVFIFSYMKVGTNQSGDIYD
CWATFIEPWGAKTYVTWYAFTIFIIPLLVLIYTYFNICRTIWRSKNWISTKVSPVKRCVKVSQLYEDGGAKKHRGCNGFYRERRSLWKRMSMSSNSQSMTN
SNVSAETIKLTLDEIDELSTVNENSEILDMAMRSTIVPVTEPQPVTQQSISFFQSVSSTSSALPVTRTTSNTSSCESTSVH
>Polistes_metricus
MNETTNSSFTAANEEEDIRDENLAKWEIVLLANILIFTIFGNSLVLLSLYLRRYRAHKKLSRMYFFIMNLSIADLLTGLLDVLPQLAWDITFRFQGGLVLC
KLVKFFQPFGLYLSSYILTATAIDRYHAICHPLSYCSVTSRKSRIMVYCAWSFALILCIPQLFVFSYQEVMPRIWDCWATFDIKFGEKAYVTWYSLMVFLL
PFIVLVYTYVGICVGVWQSNKMSGAVDDRTYIRSISNYSRNRSSFISKAMINTVKQTIAVITLYAITSIPFIGCELWISWDPEAPKSSFANGPAFTILALL
NSLTSCVNPWIYLSFNRELRQTLMNYFCNHNEHSQKYYGQRMMRRSDSNDTSTRTSLMSRISRYTSSIIPR-
>Wasmannia_auropunctata
MSHDLNSSSSSPPLSSDRPQDDARDEDLARWEIAVLTSIFLVTLIGNALILLALYVRRRYQRRKFTRMYFFILHLSVADLLTGLLDVLPQLAWDITFRFQG
GAVLCKLIKFGQPFGLYLSSYILTVTAIDRYQAICHPFSYCGVTSRRSKMMVYGAWTLAAALCVPQIVIFSYTEISPGVWECWATFYLKYGERAYITWYSV
TQFLLPFMVLVYTYTRICLSIWRSSKMSGVVELKRDNKASFCQRNRDPLISKAMINTVKQMIIVVTLYIVTNIPFIGCELWATWDPKASTSPFFSGAAFTI
LSLLNSLTSCVNPWIYFAFNKELRAALINFFCRKKDYSLTYDIDVRQNASDVPSTTSSFISRI
SRLASSKIFG-
>Aethina_tumida
MYGLKMNVTSTGETIEELERAAKERDESLAKIEVAVLAVIFIVTVIGNSTVILALWARRRCAGRKKLSRMYFFILHLSIADLITAFLSVLAQLAWDITYRF
RGGYILCKLVKFGQTLGPYLSSYVLMATAIDRHQAICYPLTYCSWTSRRSKFMVYIAWITSLVFCIPQLTIFSYQEVDDGEFDCWATFQEPWGQKAYVIWY
CVSVFIVPLIVLVYTYTCICKDIWHSSEISMRPRNSLVGKKRTKMPLISRAKINTVKQTIAVIFMYIICSSPFMFVQLWATVDPTNPFFQGPIFTILTLLY
SLNSCVNPWIYLAFNRELPKYLLRHYTATNKKLKSSSMGNNSGSNSSGEIQSTSLRTLSRLSRNNSGRLNRCSVRTANQNYITKYDAKDWIVATAT-
>Pogonus_chalceus
MYFFILHLSIADLVTAFLNTLPQLGWDITYRFNGGFLLCKIVKYCQTLGPYLSSYILMATAIDRHQAICYPLTYCSWTSRRSKVMVWLAWATSLAFCIPQV
TIFAYQEVAPGINDCWATFPQPWGARAYVTWYSISVFIVPLIVLIFTYGAICRVIWQSAGGELGLRSPRLAPDSPPRCARSRRAPLISRAKINTVKQTVAV
IAMYIACSTPFICAQLWATWDPEANESAFFNGAAFTILTLLSSLNSCVNPWIYLAFNRELPRLLLRHYAATSKNYRSAGTGNSASNSSGGVESTSLRPFSK
WSLCASAKSSSAINHNGNNNTNGHAIGSGDFRARYPAPPASSAASTLIRLPHAPAHSPQQLLMQQPSPFLGRYHARRWIVTTSA-
>Medauroidea_extradentata
MGDGRNCSLATSEDWCQLPNMLVANHSVKNSTNSTDGYDRDEDLAHAEIATLAVMFIITMVGNSCILYALCARMRHKKLTRMYFFILHLSIADLITGLLNV
LPQLAWDITYRFQGGPVLCKIVKYFQPLGPYLSSYVLLATALDRYQAICQPMTYCSTDSRRSRAMIWLAWCVSLVFCIPQVIIFSYQETAPGVFDCWATFP
EAWGEKTYVTWYSVSVFIIPLCVLVYTYSRICHEIWEKSGFRAAHAARSSPLKINPTISRAKINTVKQTVAVIVLYISCSSPFIGAQLWATWDPHASESAF
LTGAAFTILALLSSLNSCVNPWIYLAFNTELVRLLLKKGDPRTNADLFGGSGSSTTSWRNSKVYRLVNGSAFKQRDAVRERRWAMAMPTKIPIATALMAGR
DAPSTSQDVA-

>Extatosoma_tiaratum
MCVSRNCTSEDSDMPPLQVLDVFVGNYSAENSTNSSSGYDRDEDLARGEIATLAIMFIVTVVGNSCILFALCARRKRRKLTRMYFFILHLSIADLITGFLN
VLPQLAWDITYRFQGGPVLCKIIKYLQPLGPYLSSYVLLATALDRYQAICHPMTYCSTDSRRSRVMVWMAWCVSLAFCIPQVIIFSYQETAPGVYDCWATF
AGHWGERTYVTWYSVSVFIVPLCVLVFTYSRICHEIWEKSGFRAAHAARSSPLKMNPTISRAKINTVKQTVSVIVLYISCSSPFIGAQLWATWDPHATESP
FFTGAAFTILALLSSLNSCVNPWIYLAFNTELVHLLATCGNPRANADLIGGSGSSTTSWRNSKVYRLVSGSVFKQRDAVRERRSAPLRTIYGSLDLELL-
>Sipyloidea_sipylus
MGFNRNFSLPDKSENMVQSPLHVLEMFAANNYSVDNGNGNSNGSNSTSSGYDRDEDLARAEIATLAVMLLVTVIGNSCILYALCARRRSKKLTRMYFFILH
LSIADLITGMCNVLPQLAWDITYRFKGGPVLCKIVKFIQPLGPYLSSYVLLATALDRYQAICHPMTYCSSDSRRSRVMIWMAWCVSLAFCIPQVIIFSFQE
TVPGVYDCWGTFASDWGETTYVTWYSVSVFIVPLCVLVYTYSCICHEIWEKSGFRAAHAARSSPLKMNPTISRAKINTVKQTVAVIVLYIACSSPFIGAQL
WATWDPHASDSPFFTGAAFTILTLLSSLNSCVNPWIYLAFNTELVQLLAR
>Nasonia_giraulti
MEESATSMIAVPAEDWRDESLAVWEVIVLVLILTTTLMGNVLVLFAIYLKRCRGRRQRLTRMHFFVMHLSVADLITGLLNVLPQLAWDVTFRFQGGPILCK
LVKFCQPLGSYLSSYVLIATAVDRYHAICYPLSYCRTTSRRSRITVYVAWLLALLFCLPQVFIFSYQEISAGVWDCWATFTVPYGERAYVTWYSVTVFLLP
FCVLTFTYAEICCSIWRNREVMVLASHERQQALTKEGRSQTTLISKAKINTVKQTLAVVTLYAASSIPFVGCQLWATWDPFASSSAFFDGPIFTILSLLSS
LTSCVNPWIYLTFSYELRAALTKFLRSLIKRDRTSRFGKYQFYFLCQY-
>Telenomus_podisi
MPVESQTVKNVTEDFRDESLAQWEVGVLAAIFVITIIGNSLVLLAIYLRRYRGRRKKLTRMYFFILHLSVADLITAFFDVLPQLAWDITFRFQGGPILCKL
VKLGQPFGPYLSSYVLVVTAIDRYHAICHPLNYCRTTSRRSRFMVYAAWILAFLLCTPQAYVFSFQEVWPGVSDCWATFNLPYGERIYVTWYSVTVFVLPY
CVLIYTYTGICIGIWNNRGLSGLPNSSKNANFRGDNRANLITKARIKTVKQTIAVITLYATTNSPFIGCELWAVWSDTAASSPFITGPAFTILSLLSSLTS
CVNPWIYLTFNYELRKILLKRFGRLFCYNYAYSIDRSTRSGSSNENSTRSSFISRISRYASFVMHGPATKERISR-
>Ramulus_artemis
MGASSNCSYGATEDWRQPSLDMLAANFSAENSSSANSTYGYDRDENLAHAEIATLATMFAITLVGNFCILYALCARMRRKKLTRMYFFILHLSIADLTTGL
LNVLPQLAWDITYRFQGGPVLCKVVKFFQPLGPYLSSYVLIATALDRYQAICHPMTYCSTDSRRSRVMIWLAWCVSLGFCIPQVIIFSYQETASGVYDCWA
TFSGAWGETTYVTWYSVSVFIIPLCVLVYTYSCICHEIWEKSGFRAAHAARSSPLKMNPTISRAKINTVKQTVAVIMLYISCSSPFIGAQLWATWDPHASE
SAFFTGAAFTILALLSSLNSCVNPWIYLAFNTELVRLLVKRGDPRTNADLFGGSGSSTTSWRNSKVYRLVSGSAFKQRDSVRERRWAMAMPTKIPIATA
LMVGRDIPSTSQDAA-
>Stigmatomma_oregonense
MSYDPINSSSPSPSPLSLSLPSSSSERTSNDARDEYLARWEIAVLTSIFLITLIGNALVLFALYVRRRHQRRKFTRMYFFILHLCVADLLTDLLNVLPQLA
WDITFRFQGGAVLCKLIKFGQPFGLYLSSYILTVTAMDRYYAICHPFSYCRVTSRRSRMMVYGAWTLATILCIPQVFIFSYQEISTGVWECWAIFYLKYGE
RAYITWYSVTQFLLPFAVLVYTYVRICISVWTSNKMSRVIDLKKGSKDNFLQRNRDPFISNALINTVKQTIVVVTLYIVTSIPFIGCELWATWDPKASTSP
FFSGATFTILSLLNSLTSCVNPWIYFTFNSELRVAVTNFFYNKRDYSLTHGNC-
>Chyphotes_mellipes
MYRDRNATARGCTAGERDERDEYLARWEIAVLVTILVVTILGNGLVLLSLHVRRHRQRRKFTRMYFFIMHLSVADLLTGLLNVLPQLAWDITFRFKGGPIL
CKLVKFGQPLGPYLSSYVLMATAIDRYHAICHPLSYCRTTSKRSRVMVYGAWIITLFLCLPQVFIFSYQEIMPSVWDCWATFDPTYGEKAYVTWYSITVFL
LPLIVLTYTYTGICIGIYKSNRMSGVNDVRKSVKGKKSFDQNRTPLVSKAMINTVKQTIAVISLYTITSTPFIGCQLWATWYPKATVSPFFSGATFTILAL
LSSLTSCVNPWIYLSFNTELRVALTDYLCRRQDYSLTYGRNTHRSNSHENSTRSSLISRISRYAGTAIFR-
>Ganaspis_sp
MENGTLINDTRDEELAKWEIGILACFFVIIIAGNSLVLFSIYLKRYYSQRKKLTRMHFFIMNLSVADLLTAILNVLPQMAWKITFRFQGGPILCKLIKFVQ
PIGPYLSSYILTATAIDRYHAICFPFSYCRTTSRRSRIMVYCAWIIALILCTPQVFVFSYQQISAGIWDCWATFKMPYGQEAYVTWYSISVFLLPFTVLIY
TYTGICIGVWKNSGVSDPLEIGFAKNSHFTKKKQSPLITKARVNTMKQTIVVVTLYFLTWSPFIGCELWMAWDPIRAANSSIFDGPLFTVLSLLSCLTSCV
NPWIYMAFNSELRILLINFLRKITRHKYSPASDEASKNDHSNEPSTTSSLISKVSVYPNVRPNYSEARERNIS-
>Tetramorium_bicarinatum
MLHDFNSSSLTSPSPPSPLPPDDARDEYLARWEIAVLTSIFLVTLIGNSLILLALYARRRYQRRKFTRMYFFILHLSVADLLTGLLDVLPQLAWDITFRFQ
GGAVLCKLIKFGQPFGLYLSSYILTVTALDRYYAICHPFSYCSVTSRRSKMMVYGAWTLSVALSVPQIFIFSYTEIAPGVWECWATFYLKYGERAYITWYS
VMQFLLPFLVLTYTYTKICMAIWTSSKMSGVVDLKKNNRASFFQRNRDPLISKAMINTVKQMIIVVTLYIITNTPFIGCELWATWDPTASSSPFFSGAAFT
ILSLLNSLTSCVNPWIYFAFNKELRGALTSSFCRKKDYSLTYDIDARQNASDVPSSTSSFISRISRLASSKIFG-
>Leptopilina_boulardi
MENGTLINDTRDEELAKWEIGILACLFVIIIIGNSLVLFSIYLKRHYGQRKKLTRMHFFIMNLSVADLLTAILNVFPQMVWKITFRFQGGQILCKLIKFGQ
PIGPYLSSYILTATAIDRYHAICFPFSYCRTTSRRSRIMVYSAWIIALILCTPQIFIFSYQLISPGVWDCWATFTVPYGQEAYVTWYSISVFLLPFTVLVY
TYTGICIGVWKNSGVSDPLEIGYKKNSNIIKKKQSPLITKARVNTMKQTIVVVTLYFLTWSPFIGCELWMAWDPVRASNSSIFDGPLFTVLSLLSCLTSCV
NPWIYIAFNSELRILLLNFLRKITRHKYSPTSDDNSKIGHSNEPSTTSSLISKVSTCPTTVKPIYTEVRERNIS-
>Brachycistis_timberlakei
MKNFCETLRESHRGPSCPREQTGLHDNPGDMFADRNSSEVQTSDELDDERDEELAKWELVVLASILVITLVGNGLVLLALYARRYHCRRKFTRMYFFIMHL
SIADLLTGLLDVLPQLAWDITFRFQGGPVLCKMVKFGQPLGPYLSSYVLMATAIDRYHAICHPLSYCSTTSRRSRIMVYGAWVLALLLCLPQVIVFSYKEV
EQQVWDCWATFDLEYGERAYVTWYSVTVFLLPLIVLIYTYVGICVGIWRSNKMTGMVDVIGRGGKSGKKLEGNRSPFISKAMINTVRQTIAVISLYTISST
PFIGCQLWATWDPKAAFSPFFTGAAFTILALLSSLTSCVNPWIYLSFNRELRLALKEYFFRREDYSLAYDRDMQRSDWNEASTRSSLISRISRYANSVISH
-
>Crioscolia_alcione
MEGMPEDQNSSEEIDGRDEYLAKWEITVLTSILLLTLLGNTLVLVSLYIRKRYERRKFTRMYFFIMHLSVADMLTGLLNVLPQLAWDITFRFQGGALLCKA
VKFGQPLGHYLSSYILTAAAIDRYQAICHPLSYCSSTSRKARIMLYSAWVVALVFCLPQIFIFSYREISPNVWDCWATFDLPYGERAYVTWYAVTVFLLPF
AILLYTYVGICIGIWRSSNILGATDNRKIKGDVFVEKNRSTFVSKAIISSVKQTIIVVTLYLVTGSPFIGCQLWATWDPRAISSPFFSGAAFTILTLLSSL
TSCVNPWIYLSFNRELRAALTDYFFTSNDEYSLTYDQSMRRSDSFETSTRSTLFSRISRLTGSAMFR-
>Sphaeropthalma_orestes
MYNDRNNTTTLGEAKDDTRDEELAKWEIGVLTSIFIITILGNSLVLFALYIRRRRGRRKFTRMYFFIMHLSIADLITGLCDVLPQLAWDITFRFQGGPVLC
KIVKYIQPIGPYLSSYVLTATAIDRYHAICHPLSYCSTTSRRSRIMVYAAWIFSLILCLPQVYVFSYEEIMPNVWDCWATFGLHYGERAYVTWYSVTVFLL
PLVVLIYTYTGICIGIWQSNKMTGIMDVKIRAKGSNLPPRNRSPFISRAMINTVKQTIAVISLYTITSTPFIGCQLWATWDPKASSSPFFSGPAFTILALL
SSLTSCVNPWIYLSFNQELRVALMDYFCNSHDYLPTYDRDVRRGNSDETSTRSSLISRISRYAGSVIFR-
>Nicrophorus_vespilloides
HHSLRGSPSSEREERGSSSHRDCNFSHHLHSHHXLWTRRRYAGRKKLSRMYFFILHLSIADLITAFLSVLPQLCWDVTFRFQGGEVLCKLVKFGQTLGPYL
SSYILMATAIDRHQAICYPLTYCSWTSRRSRVMVWIAWVLSLAFCIPQITIFSYQHVENEEYDCWATFLPAWGEKAYVTWYGISVFIIPLVVLIVTYSCIC
REIWHSAVGELGMRPRNVQTGNGKSQQTGKRAPLISRAKINTVKQTIAVIVMHIVCSTPFISGQLWATWDPEAVNSPFLNGPVFTIVSLLYSLNSCFNPWI
YLAFNRELPRLLIRHYTSNRNYREAAGNSTSNSSGGVESTSLRPFTRWSFCNGNRAAAKFNPRQPQRQYVTQYNARRWIVTTT-
>Meloe_violaceus
MHFNQDYQRNMNISIENTTKSLRDDTEESPTDRDESLARIEIATLAVIFLITVIGNSTVLIALWARRRFAGRKKLSRMYFFILHLSVADLITAFLSVLPQL
AWDITYRFYGGYFLCKAIKYGQTLGPYLSSYVLMATAIDRHQAICYPLTYCSWTSRRSKVMVYIAWCTSLIFCLPQLFIFRYKELSDGIYDCWAVFEEPWG
AKAYVTWYSISVFIVPLIVLTVTYTSICIEIWHSSESSLRPRSSQKAGKRTPLISRAKINTVKQTIAVIVMYIVCSTPFILAQLWATWDPQNPFSEGPVFV

ILALLYSLNSCVNPWIYLTFNRELPRLLVKHCTASSKNYKSANAGNSGSNSSGEPQSTSLRPFSKWSFCNSPKSNGNKYTSRLTQRPYDAQSNTRRWIVTT
TT-
>Inocellia_crassicornis
MNGTELIKSAPETNGNERDEVLAQAEIITSAVIFVVTVIGNGTVLLALFARQKYSGCRKLSRMYFFILHLSIADLVTAFFNVLPQLSWDITFRFKGGPLLC
KAVKYNQLLGPYLSAYILSATAIDRHQAICYPLTYCSWTSRCSRAMIWMAWAASLAFCIPQLIIFSYQEIEPNVWDCWGTFPEPWGAQAYVTWYSLSIFII
PLCVLVFTYTCICREIWRTARHGILRPATTPPMSVTTAPTHGPVRGRRTQPFVSRAKINTVKQTVAVIVTYIVCSMPFISAQLWVTWYPHAMESPFFSGST
FTILTLLSSLNSCVNPWIFLLFNKDLFRLLKRLFVNNLDSSKTKYTRGGMSKASNSSSTVETAVGRLSWLSKSFRSSKASFQHVHNNIIQS-
>Cercopis_vulnerata
EELARAEIAVLAAIFVITVTGNTVVLVALYGRRFCGGRKKLSRMYYFILHLCIADLVTALLNVFPQLIWDITFRFKGGPFLCKLVKYGQTLGPYLSSYVLM
STAMDRYKAICHPLTYCSWTSRRSRTMVRVAWIVSLILCIPQVNIFSYQEIAPGIYDCWATFPEPWGEKLYVTWYSVSVFLIPLCVLIYTYTCICRELWRN
AGFPESTSSPHRLISRAKINTIKQTVAVIALYISCSSPFICAQLWASWDPNALSSPFLQGATFTILTLLS
>Aposthonia_japonica
MNSSAEKDSDGRDEDLARVEVTVLAIIFAITLVGNLLVLVALLVLRRKKLSRMYFFIVHLSIADLITAFFNVLPQLGWDVTYRFIGGPILCKIIKFCQTLG
PYLSSYVLLATAVDRYRAICSPLVYHSWSFRRAKIMVLIAWIISLWFCVPQAVVFSYQELEPGVWDCWATFIKPWGERAYVTWYSVFSFIIPLSVLTYTYS
CICRKIWRNTGAGFHIIQTSPVISRAKINTVKQMVIVILFYIICSTPFTFSLLWATWDVTAVELPFFKGPAFTILTLLSSLNSCVNPWIYLTFNKELIALL
VNCGASDLHYRGSSSSKERSRLSKLVSWRST-
>Tenthredo_koehleri
MSNSTAENRSFVESECNLTISSEIDDSRDEYLAYWEVATLALIFVVAIAGNSLVLTAFYFRRYDAPRKKLPRIYFFILHLSIADLLTALLNVFPQLIWEIT
FRFQGGPVLCKFVKFGQTLGPYLSSYVLAATALDRYQAVRHPLIYCSITSRRSKMTVYFAWCLSLLLCVPQTLVFSYKEVSSGVWDCWATFDLTPYGQQTY
VTWYSISVFILPLIILVYTYTSICIVIWNNNGIVGEARSTHGGLLRQRTVISRAKINSIKQMIAVISLYAASSSPFIASLLWVTWDRNASASPFYSGAAFT
ILTLLSSLNSCVNPWIYLTLNRDLSRALINRLYPTRGSSIKSQENPRQSIEIIRWGSSASSSGTSIRSYASRMSRYANSVLRRSSDSSSNKELQPSNRIR-
>Gyrinus_marinus
MNTTANATDQTFDRDENLAQFEIATLAVIFVVTVIGNGTVLLALWTRRRCAGRKKLSRMYFFILHLSIADLVTAFLNVLPQLAWEVTYRFRGGAVLCKLVK
FGQTLGPYLSSYVLMATAIDRHQAICYPLTYCSWTSRRSKVMVYTAWVAALAFCIPQLNIFSYQEIAPGVYDCWATFPQAWGQKAYVTWYGISVFIVPLTV
LTTTYGCICREIWQSAAAGDLGGP
>Chrysis_viridula
MSVEQNLNVTETEEDVRDEYLAKWEIIVLATILYMTLVGNSLLLFALYLKRHRGRRRKFTRMHFFILHLSTADLITGLFNVLPQLAWDITFRFQGGNILCK
LVKFMQPLGPYLSSYVLMATAIDRYHAICHPLSYCGSSLKRSKIMVYSAWAVTLILCIPQVIVFSYQEVTPKVWDCWAVFNVAYGERIYVTWYSTMVFLLP
FTVLLYTYTGICIGIWRSNKMSGVEDWRKKVRNNGNFLERNRSPFISKAMINTMRQTIVVISLYAITSIPFIGCQLWATWDPSASSSSFINGPAFTILSLL
SSLTSCVNPWIYLSFNRELRAVLKDNLCCRSDYSTTYDQELHPANSTETSTRSSFISRIFWYAGSMIFR-
>Baetis_sp
MDSSVDNSTTTTVDAVPTRDEQLARVEIATLAFIFAVTVIGNSLILSAILFRRRVKITRMYFYIFHLSIADLVTAFFNVLPQLAWELTYRFKGGNLLCKGV
KFGQILGPYLSSIILVLTAIDRYYAICQPLAHCTWPVRRARALVCVAWALALACCTPQVFIFSYQPIGDTYDCWGTFPEGWGERAYVTWYSGSVFLIPLLI
LIFTYTCICQAIWRNYNAKKESVAGLPPTDSKVLGNGRVMHPRVHSMRGISRAKMKTIKLTVVVIACYVVCSTPFICAQLWATWDPDAMQSPFWSGTQPTT
HLHSMFLLHHAR
>Haploembia_palaui
MVHRKRQNDVMNSSVASDGRDEDLARVEVTVLAIIFAITLVGNLLVLLALLVLRRKKLSRMYYFIVHLSIADLITAFFNVLPQLGWDVTYRFIGGPILCKV
IKFCQTLGPYLSSYVLLATAVDRYRAICSPLVYHSWSFGRAKVMVFIAWTISLLFCVPQSIIFSYQELKPGVWDCWATFIQPWGERTYITWYSVFAFIIPL
SVLTYTYSCICRKIWRNTGAGFHIIKSSPVISRAKINTVKQMVIVIFFYIICSTPFTC
>Cordulegaster_boltonii
MATLAAMLVVSVVGNGAVLIALWARPRPSPRRRLSRMHLLVLHLCTADLITAAFTVLPQMAWEATYRFAPEAAPLCKPLKVLQLLGPYLSSYLLVAMAIDR
HRAVCRPLSVRAFLPRSPRRLVAIAWALSFLFSLPQAFIFSYRKATETEWDCWATFDPPWTQKAYVTWYGISVFAVPLVLLLYAYAGICRALRRNHREKMR
PQQDGIIPRGAPLRTHSMGTISRAKMRTLRLTIVAVACYILCSCPFVLSQLWATWYPGATESPFWRGAAFTILSLLPCLNSCVNPWIYMAFNEDIRIALKE
RLPGRVRDLLRPIFHPDTNKRARHHDATASASLSSRTRSSRESPQPEVKATAESSKDEERDGCRMIGLADRTKEEKAVKESAVLLSNAPV-
>Isonychia_bicolor
VGNTCVLIAIRHRRSKITRMYFYIFHLSIADLVTAFFNVIPQLAWEITYRFKGGDELCKIIKFLQLLGPYLSSFILVLTAVDRFHAICLPLSYCTWPAKKA
KLLVSGAWVLALLCCLPQVLIFSYQPITPGGDVFDCWATFPPYWGERAYVSWYTVSVFLAPLLILFFTYTCICRAIWHNYNAKKIASDGFTTKQTNIQPRV
HSIRGISRAKIKTVKLTVVVIACYIACSSPFISAQLWATWDPMAMHS
>Aretaon_asperrimus
MDVSRNCSSAAPEEGDQHSLQILDEIVGNCSGSSANITNSTGGYDRDENLARAEIATLAVMFIITVIGNSCILYALCLRKRKKKLTRMYFFILHLSIADLI
SGLLEVLPQLAWDITFRFQGGPVLCKFVKYFQPLGPYLSSYVLLATALDRYQAICQPMTYCSSDSRRSRVMVWMAWCISLVFCIPQVIIFSYQETEPGVHD
CWATFVEKWGERTYVTWYSISVFVIPLCVLVYTYSCICREIWEKSGFRAAHAARSSPLKMNPTISRAKINTVKQTVAVIVLYISCSLPFIGAQLWATWDPH
AAQSAFFTGAAFTILALLSSLNSCVNPWIYLAFNTELVHLLTNRASAGAHADLVSGSGSSSTSWRNSRTYRLVSGSMFKSRDSVRERRWAMAMPTKIP
IATALMAGRDLPSTSEEVAS-
>Epiophlebia_superstes
GGRDEELAKAEIATLAAMLAVSVIGNGAVLVALWVRPRPSPRRRLSRMHLLVLYLCTADLITAAFTVLPQMAWEATYRFAPQAAPLCKPLKVLQLLGPYLS
SYLLVAMALDRHRAVCRPLSARVFLPRSPRRLVAIAWALSFLFSLPQAFVFSYRRATETEWDCWATFEPPWTQKAYVTWYGISVFAVPLVLLLYAYAGICS
VLRRNHREKTRPHDERFLSRGAPPRTHSVGTISKAKMKTLRLTIVAVACYILCSCPFVLSQLWVTWYPGATESSFWKGAAFTILSLMSCLNSCVNPWIYMA
FNEDIRVALKERLSARVRYLLRPSFRPDTTKRVRHQEASMSASSSSRGSSQPKIRANSEEGRNGGR-
>Eurylophella_sp
FVVTTIGNSCVLVALSLRRAKISRMYFYILHLSIADLVTAFFNVIPQLAWEVTYRFRGGDILCKAVKFGQLLGPYLSSYVLVLTAVDRYFAICRPLSYCTW
PARRARLLVSFTWVLALLFCLPQLAVFSYQRVQNAPDKQDQYDCWATFQPAWGERAYVTWYAGSVFLVPLAILVYAYLCICFAIWRNYNNKKQFSAQGIHH
QHIVRNMNNPRVHSIRGISRAKMKTVKLTVVVIACYIFCS
>Pogonognathellus_sp
MATNSNTTNEISEFPENKSAAAVPDSGRNESLANIEIAVLSVILFFAVVGNGLMLLALRRQLQFRPMSRMYFFMLNLSVADLMVAFGNILPQLAWDITYRF
KGNDLLCRVVKFLQIFVLYLSTYVLTSMAVDRYLVVCHHSFTRNHYGGLRGPKILVILSWILSFIFASPQAFIFSFKELTMNDQTKVYDCWSTFIEPWGVR
AYVTWFVVCAFILPLVIIGASYGAICCKVISFNLPNES
>Meinertellus_cundinamarcensis
LAIVEVTVLAVVLFVALAGNTMALFSLRLLRRLRPLSRMYFFMQQLSIADLMVAVFNVLPQLIWDITYRFKGGDVLCRLVKFFQVMPLYGSTYVLMFLAVD
RYRAVCSAGPSPQAALKEARLMVCAAWILSIILAIPQALVFTHREIAPGIQDCWADFPGGQQGAKIYVLWFVIAVFMAPLTVIAACYGIISLKVWQFSSPQ
STTAEPLAAICCARCKRSSKSSSDRDTVLLRPIQSRNTSQRSQSARLSRARMRTLRLTLAVVACFIICWAPFCVSQLVYNFNPPTDQKQMSGVTVIFLLLA
SLNSCTNPWLYLAFSGSLLNHLRVCLGLGIGRAQDGDSIGDEPEGKTPPPIQKQTNQDGGGGGGTKSDLV-
>Chrysopa_pallens
ILTLAIIFVVTLLGNGAVLLALYNRQKCTGRKKLSRIYFFILHLSIADLVTAFLNVLPQLAWEITYRFQGGPVLCKAVKFGQPLGTYLSSYILSATAIDRH
QAICYPLTYCSWTSQRSQVMVWTAWIISLIFCIPQLFIFSYQEVSPGIWDCWGTFPEKWGEKTYVTWYSISVFIIPLSILVFTYTRICCEIWRNANIGMPL
RAMSSPPCTPKRRAPLISRAKINTVKQTVAVIILYIASSSPFISAQLWATWDPYAYKSPFWTGATFTILTLLSSLNSCVNPWIYLSFNKELRKLLKHIICS
KTYKNKNYSTAITNSGGSNSSQGVESTTVRVPLRISFYTRNDGGANGKQTYTTNYHPSVRYTPKKWTVAIEELPP-

>Corydalus_cornutus
VTAFFNVLPQLIWEVTYRFRGGEILCKAVKFAQPVGPYLSSYVLTATAIDRHQAICHPLTYCSWTSRRSKGMVYLAWLCSLIFCIPQVFIFSYQIVPDAPG
VYDCWATFSETWGEKAYVTWY
>Aleochara_curtula
RYAGRKKLSRMYFFILHLSIADLITAFLSDLPQLGWEVTYRFYGGGALCKMVKFGQTLGPYLSSYILMATAIDRHQAICYP
>Cotesia_vestalis
DNSTSLDTRDENLAFWEIIVLVINLIITLIGNCFIFFALYLRRYHGRQRKLTRMYFFMMHLSIADFITGIFNILPQLSWDITFRFQGGPAMCKIVKFLQPF
GNYLSSYVLTATAIDRYQAICNPFSYCR
>Galloisiana_yuasai
ADLITALFNVLPQLGWEITYRFSGGPVLCKAVKFGQTLGPYLSSYVLLATALDRFQAICHPLTYCSWTSRRSRTMVWLAWAFS
>Liposcelis_bostrychophila
MNTKAREFGNKGKRKTSTRSQKHLSHNEKMDEEARQSSIPDINLTADPTMELVRKNDSTDDFGRNEDLAQIEIGTLGVILIVTILGNGLVLLALYAKKCKA
GKKTLSRMYFYIFHLCVADLITAFLNVLPQLFWDITYRFKGNQFLCKSVKYG
>Zootermopsis_nevadensis
EVSEACNLTTANSTSGLDRDEALAQAEIATLATMLTVTVLGNFAVLLALSARPRKKLSRMYYFILHLSVADLCTAFLSVLPQLAWDVTYRFYGGPLLCKIV
KYGQTLGPYLSAYILMATALDRYQAVCHPLAYCSWTSRRSRAMVWMAWVFALIFCIPQVS
>Conwentzia_psociformis
IPYKMNDFNATDQNYGFEYFLNSTASDAVIEAGTDLAAASDNGDERNEELAKIEILILIVIFNVTLIGNLVVLLALSARKRYSGHRKLTRMFFFILHLSLA
DLISAFLNVLPQMIWK
>Stenobothrus_lineatus
QLSVVDLLNGLLNVLPQLAWEVTFRWRGGAVLCKLVKFGQPLGPYLSSSVLVVTALDRHQAVCRPLTYCSWTYRRSRAMMQVAW
>Leptopilina_clavipes
VADLLTAVLNVLPQMAWKITFRFQGGPFLCKLIKFGQPIGPYLSSYILTATAIDRYHAICFPFSYCRTTSRRSRIMVYSAWIIALILCTPQVFVFSYQLIS
PGIWDCWATFTVPYGQEAYVTWYSISVFLLPFTVLVYTYTGICIGVWKNSGVSDPLEIGYKKNSNIIKKKAKSINNKGKSKYDETNNCCC-
>Folsomia_candida
PLHEDNFSLTPNLSLVNTTLTPASPRDEFLAKIEQSVLAIIFILTIVGNFLVLMGIYFRKQKMTRMYYFIAHLAIADLTTAFFQVLTQLAWEVTYRFQGG
>Occasjapyx_japonicus
LESPRMNASSNASATGGMNGYERNEDLAKVEIAILSTIFVLTVIGNSMVLVAIFLKRKKMSRMYYFILHLSIADLITGFFNVLTQLCWEVTYRFVGGNFMC
KFIKYMQILGPYLSSYVLVMTAIDRYQAICFPLSNCSWTPKRSKVMIGISWVISLMFCAP
>Zorotypus_caudelli
LLVIILLVTLVGNGIVLFAIYVRRCRGRRHRLTRMHFFVMHLSIADLINGLLNVLPQLAWDITFRFQGGQILCKMVKF
>Perla_marginata
EVTSLAAIFLTALAGNVCVLLALYGHRNRKLTRMHFFILHLTIADMVTAVFSVMPQMLWKATYRFAGGPTLCKVVKVGQALG
>Osmylus_fulvicephalus
RTTMALMNNSVGMSGVPTPSLELVGVLAAGGGGGLLLPVDPTSANSTAMHDYDRDEALAQVEIGTLALIFGVTLLGNGAVLIALYIRRRYSGRKKLSRMYF
FILHLSIADLVTAFFNVLPQLAWEVTYRFRGGGVLCKAVKFS
>Leuctra_sp
LVTAVVANLLVLTALILRSRRQRLARLHFFILHLSLADLVNALFNVLPQLVWKSTFRFVGGTVLCKLVKFGQTLGPHLTAFILVATLWDRTHAFCY
>Mantis_religiosa
AILSTLFVVIVLGNSSVLLALSSRRRKKLSRMYYFILHLSIADLITALLSVLPQLAWDITYRFHGGPLLCKLIKYGQTL
>Arachnocampa_luminosa
MTSYQENILPNYSEQDKFENSNVSLNNSNSVQQFQLNSSLLTKIEVFLLSSLFLLTVIGNLIVILIILLYRNMSPKRARWFKLNKNISRMSFYIIHLSIAD
FNVAFMSILPQLIWRQSVVFNHSHFLCKFVAFSQVFSVYASTFLLIVMAYDRFKCICWPIKSCSWNYRHALVPVFFSWILAAVVSSPQLFLFKIQHMSVQH
YTVETCSVKWLSKKHEGLYLLFHMSTQFLIPLFVLTFLYSRIFMTVSKNIKQKHASIRFERESSKTATEINQLNQENSLNTQNGLITEVKHDSDNSIFRDK
KFGILWWIRSRFNKGIRHKILFTSTKKRGKNIKFGKTGNFLMNDQTMPKEYEMRLMLKRKETLSDSEKYSMRLDKNSVYSKSFKSNFPIRQTFSGKALTRS
KIKTLKLTLTVVITYVMCSLPFYVCTFIHFLLGLSAHNHSSLFTKTLVYITISTNMLFQLNSCANPFIYLFFNGNICKLAKRLKTKV-
>Diaphorina_citri
MVVHNVGSFLSSYILMAIAIDRYRAICHPLTYHTWNSRRSRIMIMTAWSTAFTFCIPQITIFSYQEVKPGIYDCWASWDQEWGERAYVTWFSITVFIIPLF
CLVYTYTCICVALWQNTTYPTTSPHKIISRAKINTIKQTIAVITLYIVCSLPFIFTQLWQTWNLNQTVYPVFQDNAVLTIIMLLSSLNSCVNPWIYLVSNR
ELIAALRTLCSNLRFSDDTELTMDTASASGSKECGRRTESDLTFLNRTTYSNPDENGALIPLIILTFAYTCICRAIINFSMKNSIRRHQFSRSSRTSRDSE
TENELCHTIVDSDG
>Blaberus_atropos
NTVKQMVAVLFLYVACSAPFISAQLWATWDDLAHTRPFFSGTAFTILALLYSLNSCMNPWIYLTFNRELIRFLTQQLFCRTQ
>Frankliniella_cephalica
SIEPVEGGYDCWATFVPEWGQRAYVTWYTCSVFMIPLTVLLFTYSRICCEIWRSASSKHAPALVDGTCKTCPHVHAYAYRFAPHQQPAPVSSNSFAYARSQ
QQATQRTNPLISRAKINTVKQTVLVIAIYIICSAPFICVQLWAAWGNADNPFFQGRYMTILTLLFSLNSCVNPWIYLAFNNDLVRMLWLLVSCQTGHPSGS
VLSAHSGGSGSSQSNTTQHRTCFTD
>Tricholepidion_gertschi
TRGRPVIIHQGATVGQADVRPAPVNPRTHSMKGISRAKIKTVKLTVVVIACYIFCSSPFISVQLWATWDPNASKSPFWTGTTFTIFTLLASLNSCVNPWIY
LTFNRDLVRLLNQVL
>Carabus_granulatus
SRAKINTVKQTIAVIAMYIICSTPFICAQLWATWDPESTFLNGAAYTILTLLYSLNSCVNPWIYLAFNRELPRLLLRHYAATSKNYRSAGTGNSASNSSGG
VESTSLRPFSKWSLCASTKSTPRY
>Acanthocasuarina_muellerianae
KIISRAKINTVKQTVAVITLYIICSLPFIFAQLWQTWNLNESFYPVFQDNAVLTIIMLLSSLNSCVNPWIYLFSNRKLITALRTLCVNLVLSEEAKVAM
DTASGMSSSRERRNESEMTLGTFLNRHEDTGMSDDSLTLRSRQWVVTLPDANQTR-
>Anurida_maritima
SDFLCRLIKFLQIFVLYLSTYILISMTLDRYLVVCHHSFSRNHYGGLKSPKVMVAISWILSLIFASPQAVIFSQSEIRPGVFDCWTFLGPSGVKAYVTWFV
ISVFLLPLLICGISYGAICFKVVSFSLHESNNRKLNEVSRYSKNSRSVSSSPETQSEVVIEEAPTPKDVGSRRLAQQISEVKIKTLKLTLVVVLCFFVCWS
PFCVTQLILVYNPTLPVGPGNVILLLLPSLNSCTNPWIYLAFSESLCNQLRVFLGIGLRRGSSGESIGAEQDIILRTKKFTKPRPDTRPHQDKSRTMKQVS
TLDAIENQGAFQLQLETKSEVL-
>Machilis_hrabei
GISRAKIKTVKLTVVVIACYVTCSTPFICAELWAAWDPNAVHSSFWQGPAFTILTLLASLNSCVNPWLYLVFNPNLVRDFRHGLKKLMCCGGDYLHNSGIP
SGGGFDRGTLFRSSSTSDSKAPSANQNKIAVTRTSVSSQSGVLLKRRTLNGDKQPTAVEMRNWKCDETNRPLPLLAETSDFIQCAIGENE
>Glomeris_pustulata
KTVKLTVVVILSYIACSMPYISAMLWLVWYPEARNTPFGRSAAFTILALLASLNSCANPWIYLCFNENVAQTLGDVICCRKCRNSSATNEHGAPIVGVFSR
SSLSDTNQRSTHVHTLAHGQG-

```
>Dendroctonus_frontalis
ICYPLTYCSWTSRRSKFMMYVAWIASLLCCIPQVIIFSFQEVEPRVYDCWATFDNEWGEQAYVVCYSVTVFIIPLFVLVYTYTCICREIWRCSDSSLRPRS
MSKQSTKRDRVPLISRAKINTVKQTIAVIVLYLACSAPFIVAQLWATFDPKSAFLEGSTFTILTLLYSLNSCVNPWIYLFFNRELPRLLIRHFLASNKSYR
GAPDGQTLSNSSGEQVTSLKLTRASPGRDRHLTNGRQWMVTAT-
>Mischocyttarus-_flavitarsis
QGGMVLCKLVKFFQPLGLYLSSYILTATAIDRYHAICHPLSYCSVTSRKSRIMVYCAWSLSLILCIPQLFVFSYQEITPNIWDCWATFDIKFGERAYVTWY
SLMVFLLPFIVLVYTYVGICVGVWQSNKMSDAVDDRKYIRSISSCTRNRSSFISKAMINTVKQTIAVITLYAITSTPFIGCELWVTWDTEAIKSSFANGPA
FTILTLLNSLTSCVNPWIYLSFNRELRQNLMNYFYNRNDEYSQTYYGQRGMMRRSDSNDTSTRASLMSRISRYASSIIPR-
>Argochrysis_armilla
GGNVLCKLVKFIQPLGPYLSSYVMATAIDRYHAICHPLSYCGSSLKRSKIMVYSAWAVALILCIPQVVVFSYQEVTPKVWDCWAVFDVAYGERIYVTWYST
MVFLLPFTVLLYTYTGICIGIWRSNKMTSAENWRKKSRNSVNILERNRSPFISKAMINTMRQTIVVISLYAITSIPFIGCQLWATWDPSASSSPFITGPAF
TILSLLSSLTSCVNPWIYLSFNRELRSALKDNLWCRNNYSPTYDQELHPANSNETSTRSSLISRISRYAGSMIFR-
>Calanus_finmarchicus
ADILTAIFTLIPEIAWTLTLPAFWGGDAVCKIIKFLQMLGPYLSSFLLCVTSMDRYQAICKPFKTCSWEPKNSDKLVGGAWVLAILCCIPQLLIFNTSSQ
IGCNSGEMFEKCQTNFPTWVTPSQYILYFSFANFFIPLLILLFSNCFICKTIWASSSNLNWNRRTTKKENKMDSPKLYITMANGKPKSKLSVQNFRSTSVM
VSRAKIKSVKLTLVIIACYILCSAPFIVGQLLATLGPPHIRSKIGLQMEPLFWLMTLNSLVNPWIYIWFNRNTLFVRQTSNENTTALQLSQMNEVFSPSSP
TPTRLRRNLSKNSVLRESSFIEVVGLQE-
>Tigriopus_californicus
MATTLLSSDIEAKMDKEPQDNPYNSIPDSNETKGSLDLNFTTDGGPDFSRDQDLATAEVVVQAVIFVLALVGNGLVLVALIRQLRRKPSSRMYRLMYHLSI
ADLLVAVLNVLPQLIWDITHRFHGPDILCRLVKFGQVMPIYLSSFILTLMAVDRVGVTKRHGSVEPKPLRPLIQGVWLVAILCALPQPFIFSIKDIDDKGI
YDCWADFGGEKWLEKGYVVTFLFTVFFLPFIVITSSYCIVTYRIWSYSRATQGSKASPQMSLASNRVVLSSVGDGSKNEDDDDGATMETAVYEMKVINGRN
GSSSSHRSSCKSTTRSINTSCTIYRPNLAHNSHAPTGGFVRVQTEQIVLLSKAKRKSLSMTAIVSVCFAVCWLPWCVTMLLMSFDIQVGGDKVHPLMVIFA
LLASLNSTTNPWIYLCFSSAVLQQVKHLVGMRSTIGGPDSLGGADADRPEPQTHHRTKRILFTRKAEPLKGNPLGARPHPLFALQDQEIVRAGIRSEPNLS
SHHRPNVKNPLPASNQNKSWTKT
>Atta_cephalotes
MSHDLNSSSSSELPLDDDARDEYLARWEIAVLTSIFLITIIGNVLVLLALYARRRYQRRKFTRMYFFIMHLSVADLLTGLFDVLPQLAWDITFRFQGGAVL
CKLIKFGQPFGLYLSSYILTVTAIDRYHAICHPFSYCSVTSRRSKMMVYGTWMLAAMLCVPQIFIFSYMEISPGVWECWAIFYLKYGERAYITWYSVMQFL
LPFIVLVYTYTRICISIWRSSKLSGLIDLKKSNKVSFCQRNRDPLISKAMINTVKQMIVVVILYIVTNTPFIGCELWATWDPKASTSPFFTGAAFTILSLL
NSLTSCVNPWIYFAFNKELRVALTCFFYRKKDYSLTYDIDARQNASDVPSSTSSFISRISRLASSKIFG-
>Brachionus_koreanus gb|GBXV01023685.1| TSA: Brachionus sp. 'koreanus' Rotifer_01189 transcribed RNA sequence
MTSSYQENMLLDYSEQKKSEEELQNLSFNNSDSSEQFQINNKLLTQIEVFLLSCLFVLTVIGNLTVILIILLYRNMSSKRARWFKLNKNISRMSFYIIHLS
IADLNVAFMSILPQLIWRQSVVFNHSHFLCKFVAFSQVFSVYASTFLLIVMAYDRFKCICWPIKSCSWNCRHALIGIFVAWIIAATISSPQLFLFKIQHMN
AHVYTVETCSVKWMSKRHEGLYLLFHMSTHFLIPFFVLTYLYSRIFLTVSRNISRKHASVRFERESSKTGTEINQLNRENSLNTQNGLITEIKNESENSIF
RDKKTGIILWLRSHFNKSIRHKILYTSTKKSGKNIKFSRSQNFSLNNSLPKEYEMKLMLKKNDPLSESDNRYSMRLDKNSVYSKSFKKNFPIRQTFTGKAL
TKSKIKTLKLTLTVVITYVMCSLPFYVCTFIHFLLGLSAHNHSNLFTKILVFATISTNMLFQLNSC
```

(B)

```
>Tetranychus_urticae gi|1005961572|ref|XP_015789083.1| PREDICTED: isotocin receptor-like
MDLSSFGSLEFSTLSVFSTNETSSTLPSLVLPFDVNLTSSRYLKAVSTGSPSASSLSSSSAHSRDENLAILEIITLSAIFVMIVFGNTSVLLALILSRIKL
RRMYFFLLHLSIADLITAFANVLPQLIWEITERFYGGNVLCKGIKYLQILGPYLSSFTLCATAIDRYRAICFPLESRSALNRSKLKVSIAWGLALICCLPQ
LFIFSYRQIPNSPGVYECWGTFIQPYGERLYVTWYALTSFFIPFVILIFTFSNICLELWRNGRRIKKANLSQSHSTAYFSKAKIKTVKLTVTIIICYICCS
SPFNVVQIWAYWAPGAQESSLWRGPLITIAMLLPSLNSCVNPWIYLFFNRNLLSTLDKLFCYCSTNTNINAGNRSKTLDDSSVNIEPHCSSINRNGSRTIF
VSQKMSKYSDDKPSVEAHG
>Nasonia_vitripennis gi|288558761|ref|NM_001172274.1| Nasonia vitripennis neuropeptide receptor (ITR), mRNA
MEESATSMIAAPAEDWRDESLAVWEVIVLALILTTTFMGNVLVLFAIYLKRCRGKRQRLTRMHFFVMHLSVADLITGLLNVLPQLAWDVTFRFQGGPILCK
LVKFCQPLGSYLSSYVLIATAVDRYHAICYPLSYCRTTSRRSRITVYVAWLLALLFCLPQVFIFSYQEISAGVWDCWATFTVPYGERAYVTWYSVTVFLLP
FCVLTFTYAEICCSIWRNREVMVLASHERQQALTKEGRSQTTLISKAKINTVKQTLAVVTLYAASSIPFVGCQLWATWDPFASSSAFFDGPIFTILSLLSS
LTSCVNPWIYLTFSYELRAALTKFLRSLIKRDRTSRFERASSNANSNETRSSKRSSFISRMSRYTSFIIYGPMRTIEIVNKLYI-
>Orussus_abietinus gi|817193573|ref|XP_012272252.1| PREDICTED: vasopressin V1b receptor-like
MIHEDEMDAEDPPTTTNPVEDFRDSDLARWEIFTLAAILLAILLENSLVLLALYLRGRRKLSRIQFFILHLSIADLLTGLLNVLPQLAWDITFRFQGGDLL
CRLVKFGQPFGLYLSSYVLTATAIDRYRAICKPLDSLDHRICLLQSRNMIRCSWCLALLFSLPQVFVFSYKEISPGVWDCWATFELSYGERAYVTWYCLAV
FLLPLCVLVYTYSGICRVVRRRSEVGVATSKSPRGDIRAPATISRAKIRTAWQTIVVVALYIASSTPFIGCELWATWDPGASSSAFLNGPAFTILALLSSL
NGCVNPWVHLGFDPELREVILRHLQAPTRSKQEADRKP
>Polistes_canadensis gi|954548184|ref|XP_014616661.1| PREDICTED: vasopressin V1a receptor-like
MNETTNSSFTVANEEKDTRDENLAKWEIVLLANILIFTIFGNSLVLLSLYLRKYRAHKKLSRMYFFIMNLSIADLLTGLLDVLPQLAWDITFRFQGGLVLC
KLVKFFQPFGLYLSSYILTATAIDRYHAICHPLSYCSVTSRQSRIMVYCAWSFALILCIPQLFVFSYQEVMPRIWDCWATFDIQFGEKAYVTWYSLMVFLL
PFIVLVYTYVGICVGVWQSNKMSGAVDDRAYIRSISNYSRNRSSFISKAMINTVKQTIAVITLYAITSIPFIGCELWVSWDTKAFESSFANGPAFTILALL
NSLTSCVNPWIYLSFNRELRQTLMNYFCNRNEHSQKYYGQRMMRRSDSNDTSTRISLMSRISRYTSSIIRR
>Metaseiulus_occidentalis1 gi|391341506|ref|XP_003745071.1| PREDICTED: oxytocin receptor-like
MNSSAGNFGDARDETIAHVRITVLCFCLLFTLGGNLFVFLLVTRNRPKSVHQLHIYHFMVHLSLADMLVGVFNILPQIVWEIYFRFRWGNIACKFVKFMQI
FVLYLSTYTLVGLSLNSSLVVRSGSSSTSSRLWVILGAGWFLSALLASPQVYIFSFKQLSNGVYDCWGTFDPPLTSVRYVVVYFIAMALIVPALIMGACYSY
LCRASSKRLMSDAKLKTIRMTMVMVLVFVLCWTPFCCAQLYLVFGGEEASTFVTMCLMVPNLNSCANPWVYLSFSTDLRRRLVNFCSLIHLRGNRYGEESR
KPCTRANAIVCEKPARQLRGYRIS
>Metaseiulus_occidentalis2 gi|391328891|ref|XP_003738916.1| PREDICTED: vasopressin V1a receptor-like
MEYNITDTDFVPQPENWVLSVKCATLVIIFFLTLSSNLFVLHAVLLRNRQNRPLSRVQLFMLHLTVADILVALLNILPQLAWDLTIQFRGGAVLCKLVKFA
QVYVLYLSTYILTGMSLDRLITMRAIESQWQTASLRRSDHDQSSDKSPQSHKIKFNNHRCRVGYRKFAKKLIVFAWFLAAALSIPQLFLFSYTRIHDHGSW
YDCKANFSWHRFGQRIYTTYFVVLILGVPVVMMLFCYIQICLIIVRIQRHHMTKTTSAPANFGVNTAGFFESNRRITKAKVRMVKMTFTVVLCFIVCWSPY
SIAELLLAYKIAGGEHVSPPFMVFLLLASLNSAVNPWIYTAFTNSFACKSLRTLKPEVHLITV
>Strigamia_maritima1 Chipman AD et al. PLoS Biol. 2014 Nov 25;12(11):e1002005. doi: 10.1371/journal.pbio.1002005. eCollection 2014
MSNESHWEDDLTMDNATSNAPPPQSSAIADAKLTVLALMFATIMLGNTIVITSLLIRRKKLTRMYFFILHLCIADLIVAFLHVLPQLCWDVTHRFQGNDFL
CKVIKFGQLLGPYLSSYILVGMAIDRYLAICFPLNSWAATRSKGMVCVAWVVSVVFSSPQMFLFSYKYVSVLKYWECWVDFTLEEERAYVTWYFVANTFVP
LLVLVYTYSAICWAVWRNFRHKKNSPKGDFDSAASSSPTTATHLSVPQNVNHIQRNGSCRFKASESVNPRSHGLQRISRAKIKTVKLTVVVITCYILCSMP
```

YTCAMMWVAYDKRAQETAFYKGGLFPVLVLVASLNSCVNPWVYLLFNKNLVHTLRHYLCCSKENEMKSHRTLATTYSRTSFSESVGPRVALQSLPPSSNNF
LRVNDNKLNVRVAAVSDSALYSCEKRHSSPNLNVKSAASTLVLCSQQHHHDTQNHLKDGVTMETQCA
>Strigamia_maritima2 Chipman AD et al. PLoS Biol. 2014 Nov 25;12(11):e1002005. doi: 10.1371/journal.pbio.1002005. eCollection 2014
MATHAEGDSYTRTNPPPTKTIPMDDNNPNYNNKTTDGIMDIRDENLASIEIAVQSIILVVAIISNSFVFAALCCQRRFRPTSRMYLFMLHLSVADLLVAFL
SLLPQLIWDVTFRFQGSDILCRVVKYLQVMVLYLSTYILVMMAVDRCRAVCWSITGHWNSLRAAKLMIVGAWVLAFLAAIPQAIIFGKMEIRPGVHDCWAH
FEQPWGEKAYVTWFVLSIFIIPLIVIAASYGFICYTLWIYDEEHGRSGDVIALRAVGNGTGGGDTVTDSCRLYSRRVAGVSNPNPISEAKVKTLKLTFLVV
VCFFVCWSPFCITQLVLTFNPPSPDAQIGSVEVILLLLASLNSCTNPWIYVAFSGSLLNQLRMCLGLGLTRPKDIASIGDEEPQPQQQQPTNKLPSTKSEM
V
>Ixodes_scapularis1 gi|215501697|gb|EEC11191.1| vasopressin v1a recpetor, putative
MDVPSNQKPEFAASVPTVLNFASTNLTASTETRDESLALVELAVLTLIFAFTVMGNSTVLLALATRRSKMTRMYYFLLHLCVSDLITAFLTVLPQLGWDAT
YRFQGGNLACKAVKFGQLLGPYLSSYVLVVTAVDRYQAICFPLSNCSWTPTKSKLLICAAWVAALLCCVPQVFIFSYQEVSPEVFDCWGTYVESWGLRAYV
TWYGMSVFFVPLMVLTFTYVCICRSIWRNLYLKRKSSDAESWKGERAYRFRGLAHNGTKQDSSFVGPRSHSVRGLSRAKIKTVKITVVVIALYVVCSSPFI
CVQMWMYWSPHVDMANPWISE
>Ixodes_scapularis3 gi|215501196|gb|EEC10690.1| vasopressin v1a recpetor, putative, partial
MDLVANQDQPAFSTSLPDSLDDVPVNETFWTGTRDESLAQIELGVLTAIFTFTVIGNTTVLAALAARRSKMSRMYYFLLHLCVSDLITAFFTVLPQLGWDA
TYRFRGGNLACKTVKFGQLLGPYLSSYVLVVTAADRYQAICFPLSNCSWTPTKSKLLISAAWVASLLCCVPQVFIFSYQEISPKVFDCWGTYVEPWGLRAY
VTWYGVSVFFVPLVVLSFTYVCICRSIWRNLYLKRKSSDAESWKGSRAYRFKGTAQNGTKQDSSFTVKITVVVIALYVVCSSPFICVQMWMYWSPDAD
>Ixodes_scapularis2 gi|215493320|gb|EEC02961.1| vasopressin v1a recpetor, putative, partial
QVEVAVLTFIFGFTVLGNTCVLVALAARRSKMTRMYYFLIHLCVSDLTTAFLSVLPQLGWDATHRFRGGNLVCKAVKFGQLLGPYLSSYVLVVTAVDRYQA
ICFPLSNCSWSPTKCKLLIAGAWVAALVCCVPQVFIFSYQEVAPGVFDCWGTFAQPWGLRAYVTWYSVSVFFVPLAVLSFTYVCICRSIWRNLYLKRKSSD
ADHSVRGMSRAKVKTVKITVVVIALYIVCSSPFICVQMWMYWSPDVDLADVWINATVTILMLLNSLNSCVNPWVYLFFNRSLVHTLRHQICRCPDAKNVSS
GIAGTGSCPSAVILADLTTQGTEAQSVLSYKSSPTDTSARLATAVVGKRGATDVEEDLQCSLEKTS
>Ixodes_ricinus gi|1016843980|gb|JAR88791.1| putative vasopressin v1a receptor
MDLVANQDQPAFSTSLPDPLDDVPVNDTSWTGTRDESLAQIELGVLTAIFTFTVIGNTTVLAALAARRSKMSRMYYFLLHLCVSDLITAFFTVLPQLGWDA
TYRFRGGNLACKTVKFGQLLGPYLSSYVLVVTAADRYQAICFPLSNCSWTPTKSKLLISAAWVASLLCCVPQVFIFSYQEISPRVFDCWGTYVEPWGLRAY
VTWYGVSVFFVPLAVLSFTYVCICRSIWRNLYLKRKSSDAESWKGSRAYRFKGTAQNGTKQDSSFVGPRSHSVRGLSRAKIKTVKITVVVIALYVVCSSPF
ICVQMWMYWSPDADMADTWTNTAGSSVTQAEGSYIRCGKTKCRNVFSSLLSTSNVGKQFNCSIMLR
>Limulus_polyphemus1 gi|926615389|ref|XP_013772773.1| PREDICTED: oxytocin receptor-like
MSTLDFGETPEILNVSFMNSSTIINNTKDKGIQRDETIARIEVGVLASIFTLTILGNMCVLVALAVRRIKMARMYYFLLHLCISDLITAFFNILPQLAWDV
TYRFYGGNILCKVIKYLQILGPYLSSYVLMVTAIDRYHAICFPLRNCVWTPRKSKLMIIAAWTISMLCCVPQVFIFSYQKVLIEPITFDCWGVFIQPWGEK
VYVLWYALSQFFIPLIVITFTCVRICKNVWYNFHLRGKNSSRSHSMRGLSRAKVKTVRITVVVIACYIICSTPFIVVQLWAYWSPYAQTSPIWKGPTVAIL
MLLASLNSCVNPWIYLAFNHNLVTALKHICCHERSQGYSPQVPVENINNTTSRSSQTDNEICTSYPLSLRCGVHYTSSFRLKKTKITGCRPCPNIQDLHDE
AETFSA
>Limulus_polyphemus2 gi|926636334|ref|XP_013784095.1| PREDICTED: oxytocin receptor-like
MSTLAKGETTGFLNLSFVNLSTITNNTKADVVQRDETVARIEVAVLATIFTLTVIGNTIVLVALVVRRIKMTRMYYFLQHLCISDLITAFFHVLPQLAWDV
TYRFYGGNVLCKIVKYMQILGPYLSSYVLLVTAIDRYQAICFPLTSCVWTPRKSKIMIANAWIISILCCFPQIFIFSYQKVSEDPVTFDCWGIFIQPWGEK
VYVLWYAVSQFFIPLVVIAFTYIRICKSVWYNLQMRRQASNCETFTVPRSHCMRGLSRSKVKTFKITVVVITCYIICSTPFIIVQLWAYWSPNAKNSSIWK
GPTVAILMLLASLNSCVNPWIYLAFNYNLVTALREVCCQTSSKSFSSQVPENTDNNTFSRSERCSSYPLSVRTGSRHKFPDGSTTRTRKRLCRELGSKNES
TDVGENRGSVTQTETTSEPGKFVTRDKDKTLC
>Limulus_polyphemus3 gi|926627326|ref|XP_013779233.1| PREDICTED: oxytocin receptor-like
MSSLKEEQTTELTNASFVNFSPIENITVGDTQRDETIARVEVGVLAIIFILTIIGNTCVLVALAVRRTNMTRMYYFLLHLCISDLITAFFHVLPQLAWDAA
HRFYGGNVLCKVVKYLQILGPYLSSYVLMVTAIDRYQAICFPLTRCTWTPRRSKLMIVGAWIISILCCSPQAFIFSYQQVSSDPLVFDCWGTFIQPWGEKV
YVLWYTISQFFIPLLVITFTYVNVTKTVWNNYYLRKKNFIHCRSVPDLSRAKVKTIRITIVVIACYIICSTPFLAVQLWAYWSPYAQNSPIWKGPTVAILM
LLASLNSCVNPWIYLAFNHNLITALKQLCCRSVLQDYMAQSTEATSNNNNANRSYPTENEVNSSYPLSFFIAQEEPVMLRNRLKKKQ
>Daphnia_magna1 gi|940945035|gb|JAK30878.1| Vasopressin V2 receptor
MASSSLLSNVTNVTTELASSSRDEKLASIEIGTLSFILLLAVTSNLTMLIAIWRQRRNRPLSRMYFFMMHLSLADLLVALFNILPQLAWDITYRFHGGDVL
CRFVKYTQIMTLYLSTYILMFMAVDRYRAVCCRNLHWNSLKVAKCFVAASWVMSMLFAIPQAVIFHEEEISVGVTDCWVQFAEPWGAKAYVTWFVVSIFGA
PLLVVAVCYGVICRQIWIYSQSAQPSLLPSDNSAIPSHSSITETGSTLSVMRRWFLRAGMRWQKSRNSSNAIKNNSATASQLSDTIPNKISPMLPQPPPAA
PQRCQQMSLRRSNSNQNRITKAKMKTIKLTLAVVLCFVACWAPFCITQLIMVYCPPTSQADVSPVAVIILLLASLNSCSNPWIYLAFSGSLLNQMRVCLGL
RWLRGQDKDSIGEDDRRGAAGPAGGQPADTNHRFGRGPRRAQTRDQHRQVMAEQQLLPAGEQHPAMLATKCTRADIVTHLGNTKKLQFQQNHQHVEKIG
>Daphnia_magna2 gi|942393099|gb|JAN78479.1| Vasopressin V2 receptor
MASSSLLSNVTNVTTELASSSRDEKLASIEIGTLSFILLLAVTSNLTMLIAIWRQRRNRPLSRMYFFMMHLSLADLLVALFNILPQLAWDITYRFHGGDVL
CRFVKYTQIMTLYLSTYILMFMAVDRYRAVCCRNLHWNSLKVAKCFVAASWVMSMLFAIPQAVIFHEEEISVGVTDCWVQFAEPWGAKAYVTWFVVSIFGA
PLLVVAVCYXXXXXXXXXXWVQFAEPWGAKAYVTWFVVSIFGAPLLVVAVCYGVICRQIWIYSQSAQPSLLPSDNSAIPSHSSITETGSTLSVMRRWFLRAG
MRWQKSRNSSNAIKNNSATTSQLSDTIPMRSLATQQASNPLSTENKISPMLPQPPPAAPQRCQQMSLRRSNSNQNRITKAKMKTIKLTLAVVLCFVACWAP
FCITQLIMVYCPPTSQADVSPVAVIILLLASLNSCSNPWIYLAFSGSLLNQMRVCLGLRWLRGQDKDSIGEDDRRGAAGPAGGQPADTNHRFGRGPRRAQT
RDQHRQVMAEQQLLPAGEQHPAMLATKCTRADIVTHLGNTKKLQFQQNHQHVEKIG
>Daphnia_magna3 gi|939881383|gb|JAJ41077.1| Vasopressin V2 receptor
MASSSLLSNVTNVTTELASSSRDEKLASIEIGTLSFILLLAVTSNLTMLIAIWRQRRNRPLSRMYFFMMHLSLADLLVALFNILPQLAWDITYRFHGGDVL
CRFVKYTWNSRKVAKCFVAASWVMSMLFAIPQAVIFHEEEISVGVTDCWVQFAEPWGAKAYVTWFVVSIFGAPLLVVAVCYGVICRQIWIYSQSAQPSLLP
SDNSAIPSHSSITETGSTLSVMRRWFLRAGMRWQKSRNSSNAIKNNSATASQLSADPIPMRSLATQQASNPLTTENKISPMLPQPPPAAPQRCQQMSLRRS
NSNQNRITKAKMKTIKLTLAVVLCFVACWAPFCITQLIMVYCPPTSQADVSPVAVIILLLASLNSCSNPWIYLAFSGSLLNQMRVCLGLRWLRGQDKDSIG
EDDRRGAAGPAGGQPADTNHRFGRGPRRAQTRDQHRQVMAEQQLLPAGEQHPAMLATKCTRADIVTHLGNTKKLQFQQNHQHVEKIG
>Daphnia_magna4 gi|942156180|gb|JAM92944.1| Vasopressin V2 receptor
MASSSLLSNVTNVTTELASSSRDEKLASIEIGTLSFILLLAVTSNLTMLIAIWRQRRNRPLSRMYFFMMHLSLADLLVALFNILPQLAWDITYRFHGGDVL
CRFVKYTQIMTLYLSTYILMFMAVDRYRAVCCRNLHWNSLKVAKCFVAASWVMSMLFAIPQAVIFHEEEISVGVTDCWVQFAEPWGAKAYVTWFVVSIFGA
PLLVVAVCYGVICRQIWIYSQSAQPSLLPSDNSAIPSHSSITETGSTLSVMRRWFLRAGMRWQKSRNSSNAIKNNSATASQLSDTIPMRSLATQQASNPLT
TPDTIPMRSLATQQASNPLTTENKISPMLPQPPPAAPQRCQQMSLRRSNSNQNRITKAKMKTIKLTLAVVLCFVACWAPFCITQLIMVYCPPTSQADVSPV
AVIILLLASLNSCSNPWIYLAFSGSLLNQMRVCLGLRWLRGQDKDSIGEDDRRGAAGPAGGQPADTNHRFGRGPRRAQTRDQHRQVMAEQQLLPAGEQHPA
MLATKCTRADIVTHLGNTKKLQFQQNHQHVEKIG
>Daphnia_pulex tr|K9JBV2|K9JBV2_DAPPU Oxytocin/vasopressin receptor-like protein OS=Daphnia pulex PE=2 SV=1
MSTNMSNATEEPSSSLRDEKLASVEISTLSVILVLAVTSNLTMLIAIWRQRRNRPLSRMYFFMMHLSLADLLVALFNILPQLAWDITYRFQGGDVLCRFVK
YAQVMTLYLSTYILMFMAVDRYRAVCCRNLHWNSLKVAKCFVAASWVMAILFSIPQAVIFHEEEISVGVTDCWVQFVEPWGAKAYVTWFVVSIFGAPLLVI
GVCYGVICRQIWIYSQSALPSSQQPPTSSAYPALTSETGSTLSVMRRWILRASLRWQKSRSSSNNGTKNHSAIANTSQLSDTIPMRSLATQPASNPLSAEA

KVIRSLPPPLSPQCCQQMPLRRSNSNQNRITKAKMKTIKLTLAVVLCFVACWAPFCITQLIMVYCPPTSQADVSPVAVIILLLASLNSCSNPWIYLAFSGS
LLNQMRVCSILLLRSTHLFIEQLLFFIRVSRKSRNI
>Microplitis_demolitor gi|665813630|ref|XP_008555379.1| PREDICTED: vasopressin V1a receptor-like
MMGNSTQPDTRDENLAIWEITVLAINLIITTIGNCLIFFALYLRRYHGRQRKLTRMYFFMLHLSIADFVTGIFNVLPQLAWDITFRFQGGYGLCKIIKFLQ
PFGNYLSSYVLTATAIDRYQAICYPFNYCRTSSLKSRVMVYGAWSLSFVLCIPQVIVFSYQEISPGVWDCWASFSLPYGERIYVTWYSITIFLLPFLVLLY
TYASICANIRRNTEISDRFDHVNNNKKINFNNKNRQPLISRAKIKTVKQMITVVSLYVMTSSPFIGCQLWATWDPQAANSSFINGPAFTILTLMSSLTSCV
NPWIYLGFNRELRNILSNYLKKLLCKQNVINYDIDTSYVNSNGSSTKSSIIVTMFRFAGSMYHRQTYQERSTDFILTRRQS
>Vollenhovia_emeryi gi|795106077|ref|XP_011881673.1| PREDICTED: vasopressin V1a receptor-like
MSRDLNSSSSTSPSLSEWPSNDARDEYLAKWEIAVLTSIFLVTLIGNALVLLALYIRRRYQRRKFTRMYFFILHLSVADLLTGLLDVLPQLAWDITFRFQG
GAALCKLIKYGQPFGLYLSSYILTVTAMDRYYAICHPFSYCGITSRRSKMMVYGAWVLAATLCVPQIFIFSYTEISPGVWECWATFNLKYGERAYITWYSV
TQFLLPFIVLVYTYTRICMSIWTSSKMSGVVDLKRSNKASFFQRNRDPIISKAMINTVKQTIVVVTLYILTNSPFIGCELWATWDPTASSSPFFTGAAFTI
LSLLNSLTSCVNPWIYFAFNKELRAALTNFFCSKKDYSLTYDIDVRQNASDVPSSTSSFISRISRLASSKIFG
>Cerapachys_biroi gi|759037579|ref|XP_011350516.1| PREDICTED: vasopressin V1a receptor-like
MSYDLSSSSLPLSLSLPSSPSSSSSPSSLEVSDDLRDEYLARWEIAVLTSIFLITIIGNGLVLFALYARRCYQRRKFTRMYFFILHLSIADLLTGLLDVLP
QLAWDITFRFQGGAVLCKLIKFGQPFGLYLSSYILTVTAMDRYYAICHPFSYCAVTSRRSKMMVYGAWVLAAILCVPQVFVFSYKEISPGVWECWATFYLK
YGERAYITWYSVTQFLLPFIVLVYTYTQICRSIWTSGKMSGVVDFKQGNKASFSLRNRDPFISKALINTIKQTIVVVSLYIITSIPFIGCELWATWDPKAS
TSPFFTGAAFTILSLLNSLTSCVNPWIYFAFNRELRAALTNFFCRKKDYSLTYDIDAHQNASDVPSTTSSFISRISRLASSKIFG
>Harpegnathos_saltator gi|749796630|ref|XP_011151734.1| PREDICTED: vasopressin V1a receptor-like
MSYDLNSSSLSLPPSEQKTSVDARDEYLAKWEIAVLTSIFLITLIGNALVLFALYVRRRYQRHKFTRMYFFILHLSIADLLTGLLDVLPQLAWDITFRFQG
GAVLCKLIKFGQPFGLYLSSYILTVTAMDRYYAICHPFSYCNVTSRRSKMMVYAAWTLAVVLCVPQIFIFSYQEISPNVWECWATFHLKYGERAYITWYSV
TQFLLPFIVLAYTYMRICISIWASSKISGVVDFKKASFSQRNRDPFISKALINTVKQTIVVVTLYIVTSIPFIGCQLWATWDPRAAISPFLTGAAFTILCL
LNSLTSCVNPWIYFAFNRELRVALTNFFCKRKDYSLAYDVIARQNTSDAPSITSSFISRISRLASSKIFG
>Dinoponera_quadriceps gi|951533502|ref|XP_014470481.1| PREDICTED: vasopressin V1a receptor-like isoform X2
MSDDLNSTSSPPSASEQTPVDGRNEDLAKWEIALLTSIFLITLIGNTLVLLALYARKRRQRRKFSRMYFFILHLCIADLLTGLFDVLPQLAWDITFRFQGG
AVLCKLIKFGQPFGIYLSSYILTVAAVDRYYAICHPFSYCSVTSRRSKMMVYVVWLLAVVLCVPQVFIFSYQEISPNVWDCWATFNVKYGERSYVTWYSVT
QFLLPFIVLVYTYTRICITIWTSNKMSGVVDLRKGNKASFSQRNRDPFISKALINTVKQTIIVVILYIVTSTPFIGCQLWATWDPGAATSPFHTAAFTILC
LLNSLTSCINPWIYFAFNRELRSALTNLFFKRKDYSPAYDAVAQQNASDAASTTSSFISRISRLASSKIFG
>Monomorium_pharaonis gi|826425402|ref|XP_012527228.1| PREDICTED: vasopressin V1a receptor-like isoform X2
MSRDLNSSSSPSEWPLDDARDEYLARWEIAVLTSIFLLTVIGNILVLLALYVRRRYQRRKFTRMYFFILHLSVADLLTGLLDVLPQLAWDITFRFQGGAVL
CKLIKFGQPFGLYLSSYILTVTAFDRYYAICHPFSYCGVTSQRSKMMVYGAWALAVILCVPQIFIFSYMEIAPDIWECWATFYMPYGERAYITWYSVTQFL
IPFIVLIYTYTKICVSIWTSNKISGIVDFKKGNKANFSQRNRDPLISKAMINTIKQMIVVVTLYIITNTPFIGCELWATWDPKASSSPFFTGAAFTILSLL
NSLTSCVNPWIYFAFNKELRMALINFLCGKKDYSLTYGNHNVSL
>Athalia_rosae gi|817066760|ref|XP_012255235.1| PREDICTED: vasopressin V1a receptor-like isoform X2
MLCGSGMCATANEEALKVDDGNRDTRDEDIANWEVATLAAMFVVAVFGNSLVVTALYLRRYGKTPIRKKSSSRMYFFILHLSIADLITAFLNVLPQLFWEI
TYRFQGGPILCKLVKFGQPLGPYLSSYVLMVTALDRYQAICHPLNYCSVTSRRSKIMVYLAWGLSLALCIPQLVVFSYQEASPGVWDCWGTFYSPLGERAY
VTWFSISAFILPLLVLGYTNVSICLAIWRNTGIAGEARGTDGTLLRQGSVISRAKINSIKQMVAVISFYAASSSPFVASLLWVTWDPGAVNSPFFEGAAFA
ILTLMSSLNSCVNPWIYLTLNRELPRTLFARIYSSGRSASYARTREGRRRCVSTETPCNSSGSSTGTSVESYASRMSRYAYAILGKSDTANSKEFHCLQSS
SLRGIRNELE
>Pogonomyrmex_barbatus gi|769855843|ref|XP_011639495.1| PREDICTED: vasopressin V1a receptor-like isoform X1
MSHDWNSSLSSEWPPDDVRDEYLARWEIAVLTSIFLTTLIGNSLVLLALYTRRRYQRRKFTRMYFFILHLSIADLLTGLLDVLPQLAWDITFRFQGGAVLC
KLIKFGQPFGLYLSSYILTVTAIDRHYAICYPFSYCGITSRRSKMMVYGAWMLAVILCIPQIFIFSYTEISPGVWECWATFYLKYGERAYITWYSIMQFLL
PFIVLVYTYTRICMSIWTSSKMSGVVDLKRRNKASFSQRNRDPLISKAMINTVKQTIVVVTLYIITNIPFIGCELWATWDPKASTSPFFTGAAFTILSLLN
SLTSCVNPWIYFAFNKELRTALTNFFCRKKDYSLTYDIDARQNALDVPSSTSPFISRISRLISSKIFE
>Solenopsis_invicta gi|751213591|ref|XP_011159612.1| PREDICTED: vasopressin V1a receptor-like isoform X1
MSHDLNSSSSPPSSSEWPPDDARDEYLARWEIAVLTSIFLVTLIGNSLVLLALYARRRYQRRKFTRMYFFILHLSVADLLTGLLDVLPQLAWDITFRFQGG
AVLCKLIKFGQPFGLYLSSYILTVTAIDRYHAICHPFSYCGVTSRRSKMMVYGAWVLATMLCVPQIFIFSYMEISPGVWECWATFYLKYGERAYITWYSVT
QFLIPFIALTYTYTKICVSIWTSSKMSGVIDLKKGNKASFCQRNRDPLISKAMINTVKQMIVVVTLYIITNTPFIGCELWATWNPKASSSPFITGAAFTIL
SLLNSLTSCVNPWIYFAFNKELRAALTNFFYRKKDYSLTYDIDARQNASDVPSSTSSFISRISRLASSKIFG
>Nilaparvata_lugens gi|549137481|dbj|BAO01091.1| neuropeptide GPCR A41
MIEANYTFSTERVNESVVAVSNGGRDEDLARVEVATLAAILAITLAGNSTVLVALYMRRTWGGRKKLSRMYYFILHLCIADLVTGLLNVFPQLIWDITYRF
RGGPLLCKLVKYGQTLGPYLSSYVLMATAMDRYKAICHPLTYCSWTSHHARSMVQCAWATALLFCIPQVTIFSYMEVPGSPGDYDCWATFPRDWGERAYVT
WYSVSVFILPLIVLIYTYWCICRELWRNAGFPVTKASPHRLISRAKVNTVKQTVAVIALYVACSSPFIVAQLWAVWDPGAVHSPFFQGATFTILSLLSSLN
SCVNPWIYLVSNKELMRGLRQLLGCKYALSSDYRGNNSAAESGSSGCGAGSCSKRSEVTNTTSLLPRKPLVAEQPLPEVTPIRRWVVTIPPNVSREERECL
QLFSGLNNERILPRSQQQQHNQQRAIRTSSSPCRAARCRLDL
>Halyomorpha_halys gi|939664743|ref|XP_014279829.1| PREDICTED: oxytocin receptor-like
MFLYFRSLYTETRMVAFSSMGDASYSDYADKDNITSTVPSRVLCPNDSINCSAVHERDEDLAKIEMATLAVMFVMTVVGNGAVLAALFARRQSATRTKVPR
MNFFMLHLCVADMCTAWLNILPQLAWEVTFRFKGGEFLCKSVKFGQTLGPYLSSYVLTVTALDRYQAICHPLTYCSWSSKRARGMIMGAWALSITFCTPQL
TIFSYQEVAPGVFDCWAVFSEPYGERVYVTWYSISVFVVPLVSLIYTYTCICLTIWHNTGPLSASAPHKVVSRAKINTIKQTVAVIALYIACSLPFISAQL
WATWDPHATSSPFLSGGTFTILTLLSSLNSCVNPWIYLTSNQDLMEALWETIICRKNKDRPENVGSGGSSGRPHQSCTLAGQHTHYAKWVVALPPNLSESD
KTMLRLFRTPNRNVWSSLEAKYETTDSKLSPEL
>Acromyrmex_echinatior gi|332020179|gb|EGI60623.1| [Arg8]-vasotocin receptor
MSHDLNSSSSSELPLDDDARDEYLARWEIAVLTSIFLITIIGNVLILLALYARRRYQRRKFTRMYFFIMHLSVADLLTGLFDVLPQLAWDITFRFQGGAVL
CKLIKFGQPFGLYLSSYILTVTAMDRYHAICHPFSYCSVTSRRSKMMVYGTWTLAAMLCVPQIFIFSYMEISPGVWECWATFYLKYGERAYITWYSVMQFL
LPFIVLVYTYTRICISIWRSSKLSGLIDLKRSNKISFCQRNRDPLISKAMINTVKQMIVVVTLYIVTNTPFIGCELWATWDPKASTSPFFTGAAFTILSLL
NSLTSCVNPWIYFAFNKELRVALTCFFCRKKDYSLTYGNHNLSLFSLSLSLSLYIYIYISSSHVCICIIPSNIWITRNINVIFICHFLSDIDARQNTSDVP
SSTSSFISRISRLASSKIFG
>Tribolium_castaneum tr|A3RE84|A3RE84_TRICA Arginine vasopressin receptor OS=Tribolium castaneum GN=avpr PE=2 SV=2
MYTPKLSQMDISENSTYLFDKHEDRNNTDRDENLARVEVATLAIIFLVTVIGNSTVLLALWTRRRYAGRKKLSRMYFFILHLSIADLITAFLSVLPQLAWD
ITYRFYGGFLLCKVVKYGQTLGPYLSSYVLMATAIDRHQAICYPLTYCSWTSRRSKVMVYLAWVASLAFCIPQLTIFTYTSVGEDEYDCWATFQEPWGKRA
YVTWYSISVFMVPLVVLIFTYTSICIEIWQSSESSLRPRSSQKSAPGKRTPLISRAKINTVKQTIAVIVMYIACSTPFILAQLWATWDPQSPFIDGPVFVI
LTLLYSLNSCVNPWIYLAFNRELPRLLLRHYTASSKNYRSATGGNSASNSSGDAQSTSLRPFSRWSLCNSARSNKYPTRVPHRPYVAQYNARRWIVTTTT

(C)

Polistes dominula PdomSCFr1.2-0007, whole genome shotgun sequence
Sequence ID: gb|LMBU01000007.1|Length: 3730956
Number of Matches: 4
Range 1: 2381887 to 2382378 GenBankGraphics
Query: Polistes_canadensis gi|954548184|ref|XP_014616661.1|

Alignment statistics for match #1

Score	Expect	Method	Identities	Positives	Gaps	Frame
196 bits(499)	2e-87	Compositional matrix adjust.	102/164(62%)	107/164(65%)	51/164(31%)	+1

```
Query  93       RFQGGLVLCKLVKFFQPFGLYLSSYILTATAIDRYHAICHPLSYCSVTSRKSRIMVYCAW  152
                RFQGGLVLCKLVKFFQPFGLYLSSYILTATAIDRYHAICHPLSYCSVTSRKSRIMVYCAW
Sbjct  2381887  RFQGGLVLCKLVKFFQPFGLYLSSYILTATAIDRYHAICHPLSYCSVTSRKSRIMVYCAW  2382066

Query  153      SFALILCIP---------------------------------------------------  161
                S ALILCIP
Sbjct  2382067  SLALILCIPQVNMFKLRLKRNSSIFFLFFFLLLSTIP*QI*KRTEFIEHRIHTYNRYIKF  2382246

Query  162      QLFVFSYQEVMPRIWDCWATFDIKFGEKAYVTWYSLMVFLLPFI  205
                QLFVFSYQEVMP+IWDCWATFDIKFGE+AYVTW ++   LL F+
Sbjct  2382247  QLFVFSYQEVMPKIWDCWATFDIKFGERAYVTW*AIFRLLLSFL  2382378
```

Range 2: 2381495 to 2381782 GenBankGraphics
Alignment statistics for match #2

Score	Expect	Method	Identities	Positives	Gaps	Frame
147 bits(372)	2e-87	Compositional matrix adjust.	89/96(93%)	90/96(93%)	2/96(2%)	+2

```
Query  1        MNETTNSSFTAAN--EEEDIRDENLAKWEIVLLANILIFTIFGNslvllslylrryrAHK  58
                MNETTNSSFT  N  EEED RDE LAKWEIVLLANILIFTIFGNSLVLLSLYLRRYRAHK
Sbjct  2381495  MNETTNSSFTTVNKEEEEDTRDEYLAKWEIVLLANILIFTIFGNSLVLLSLYLRRYRAHK  2381674

Query  59       KLSRMYFFIMNLSIADLLTGLLDVLPQLAWDITFRF  94
                KLSRMYFFIMNLSIADLLTGLLDVLPQLAWDITFR+
Sbjct  2381675  KLSRMYFFIMNLSIADLLTGLLDVLPQLAWDITFRY  2381782
```

Range 3: 2382545 to 2383084GenBankGraphics

Alignment statistics for match #3

Score	Expect	Method	Identities	Positives	Gaps	Frame
236 bits(603)	1e-68	Compositional matrix adjust.	135/180(75%)	146/180(81%)	28/180(15%)	+2

```
Query  195      YSLMVFLLPFIVLVYTYVGICVGVWQSNKMSGAVDDRTyirsisnysrnrssfisKAMIN  254
                YSLMVFLLPFIVLVYTYVGICVGVW+SNKMSG +DD+ YIRSISNYSRNRS+FISKAMIN
Sbjct  2382545  YSLMVFLLPFIVLVYTYVGICVGVWRSNKMSGTMDDKAYIRSISNYSRNRSTFISKAMIN  2382724

Query  255      TVKQTIAVITLYAITSIPFIGCELWISWDPEAPKSSFAN---------------------  293
                TVKQTIAVITLYA+TSIPFIGCELW++WD EA K+SFAN
Sbjct  2382725  TVKQTIAVITLYALTSIPFIGCELWMTWDTEASKTSFANGK*NFSYTNLKKNVLSLII*R  2382904

Query  294      -------GPAFTILALLNSLTSCVNPWIYLSFNRELRQTLMNYFCNHNEHSQKYYGQRMM  346
                       GPAFTILALLNSLTSCVNPWIYLSFNRELRQTLMNYFCN NEHSQKYYG +++
Sbjct  2382905  FFVSFFSGPAFTILALLNSLTSCVNPWIYLSFNRELRQTLMNYFCNRNEHSQKYYGIKIL  2383084
```

Range 4: 2383149 to 2383253GenBankGraphics

Alignment statistics for match #4

Score	Expect	Method	Identities	Positives	Gaps	Frame
40.0 bits(92)	0.016	Compositional matrix adjust.	32/35(91%)	33/35(94%)	0/35(0%)	+3

```
Query  340      YYGQRMMrrsdsndtstrtsLMSRISRYTSSIIPR  374
                + GQRMMRRSDSNDTSTR SLMSRISRYTSSIIPR
Sbjct  2383149  FTGQRMMRRSDSNDTSTRISLMSRISRYTSSIIPR  2383253
```

Lasius niger Lnig_2.1_21704, whole genome shotgun sequence
Sequence ID: gb|LBMM01020432.1|Length: 1899
Number of Matches: 2
Range 1: 1398 to 1694GenBankGraphics
Query: Monomorium_pharaonis gi|826425402|ref|XP_012527228.1|

Alignment statistics for match #1

Score	Expect	Method	Identities	Positives	Gaps	Frame
162 bits(409)	6e-44	Compositional matrix adjust.	73/99(74%)	89/99(89%)	0/99(0%)	-2

```
Query  196   YSVTQFLIPFIVLIYTYTKICVSIWTSNKISGIVDFKKGNKANFSQRNRDPLISKAMINT  255
             YS+ QFL+PFIVL+YTYTKIC++IWTS+K+SG+VD KK NKANFSQRNR+PLISKAM+NT
Sbjct  1694  YSIMQFLLPFIVLVYTYTKICIAIWTSSKMSGVVDLKKNNKANFSQRNREPLISKAMMNT  1515

Query  256   IKQMIVVVTLYIITNTPFIGCELWATWDPKASSSPFFTG  294
             ++Q I+V+TLYI T+ PFIG  LWATWDPKA + PFFTG
Sbjct  1514  VRQTIIVITLYIATSFPFIGSMLWATWDPKAFTLPFFTG  1398
```

Range 2: 938 to 1099GenBankGraphics

Alignment statistics for match #2

Score	Expect	Method	Identities	Positives	Gaps	Frame
88.2 bits(217)	3e-18	Compositional matrix adjust.	45/55(82%)	46/55(83%)	1/55(1%)	-3

```
Query  292   FTGAAFTILSLLNSLTSCVNPWIYFAFNKELRMALINFLCGKKDYSLTYGNHNVS  346
             F GAAFTILSLLNSLTSCVNPWIYFAFNKELR AL NF   KKD SL YGNH+ S
Sbjct  1099  FAGAAFTILSLLNSLTSCVNPWIYFAFNKELRGALTNFFYRKKD-SLNYGNHDSS  938
```

Lasius niger Lnig_2.1_5428, whole genome shotgun sequence
Sequence ID: gb|LBMM01005371.1|Length: 12101
Number of Matches: 3
Range 1: 11103 to 11315GenBankGraphics
Query: Monomorium_pharaonis gi|826425402|ref|XP_012527228.1|

Alignment statistics for match #1

Score	Expect	Method	Identities	Positives	Gaps	Frame
135 bits(340)	4e-34	Compositional matrix adjust.	60/71(85%)	66/71(92%)	0/71(0%)	+3

```
Query  94     RFQGGAVLCKLIKFGQPFGLYLSSYILTVTAFDRYYAICHPFSYCGVTSQRSKMMVYGAW  153
              RFQGGAVLCKL+KFGQPFG+YLSSY+LTVTA DRYYAICHPF YC +TS+RSKMMVYGAW
Sbjct  11103  RFQGGAVLCKLVKFGQPFGVYLSSYVLTVTAMDRYYAICHPFLYCSITSRRSKMMVYGAW  11282

Query  154    ALAVILCVPQI  164
               LA ILCVPQ+
Sbjct  11283  TLAAILCVPQV  11315
```

Range 2: 10003 to 10236GenBankGraphics

Alignment statistics for match #2

Score	Expect	Method	Identities	Positives	Gaps	Frame
114 bits(285)	7e-27	Compositional matrix adjust.	69/78(88%)	73/78(93%)	0/78(0%)	+1

```
Query  17     DDARDEYLARWEIAVLTSIFLLTVIGNIlvllalyvrrryqrrKFTRMYFFILHLSVADL  76
              DD RDEYLARWEIAVLT IFL+T+IGN L+L ALY RRRYQRRKFTRMYFFILHLS+ADL
Sbjct  10003  DDTRDEYLARWEIAVLTIIFLVTLIGNTLILFALYARRRYQRRKFTRMYFFILHLSIADL  10182

Query  77     LTGLLDVLPQLAWDITFR  94
              LTGLLDVLPQLAWDITFR
Sbjct  10183  LTGLLDVLPQLAWDITFR  10236
```

Range 3: 11691 to 11780GenBankGraphics

Alignment statistics for match #3

Score	Expect	Method	Identities	Positives	Gaps	Frame
58.5 bits(140)	2e-08	Compositional matrix adjust.	23/30(77%)	27/30(90%)	0/30(0%)	+3

```
Query  166    IFSYMEIAPDIWECWATFYMPYGERAYITW  195
              IFSY EI+P +WECWATFY+ YGERAY+TW
Sbjct  11691  IFSYQEISPGVWECWATFYLKYGERAYVTW  11780
```

Cyphomyrmex costatus contig19312, whole genome shotgun sequence
Sequence ID: gb|LKEX01019312.1|Length: 44936
Number of Matches: 5
Range 1: 27566 to 27862GenBankGraphics
Query: Monomorium_pharaonis gi|826425402|ref|XP_012527228.1|

Alignment statistics for match #1

Score	Expect	Method	Identities	Positives	Gaps	Frame
171 bits(432)	5e-54	Compositional matrix adjust.	77/99(78%)	90/99(90%)	0/99(0%)	+2

```
Query  196    YSVTQFLIPFIVLIYTYTKICVSIWTSNKISGIVDFKKGNKANFSQRNRDPLISKAMINT  255
              YSV QFL+PFIVL+YTY +IC+SIW S+K+SG++D KK N  ++ QRNRDPLISKAMINT
Sbjct  27566  YSVMQFLLPFIVLVYTYMRICISIWKSSKLSGLIDHKKSNTVSYCQRNRDPLISKAMINT  27745

Query  256    IKQMIVVVTLYIITNTPFIGCELWATWDPKASSSPFFTG  294
              +KQMI+VVTLYI TNTPFIGCELWATWDPKAS+SPFFTG
Sbjct  27746  VKQMIIVVTLYIATNTPFIGCELWATWDPKASTSPFFTG  27862
```

Range 2: 27379 to 27489GenBankGraphics

Alignment statistics for match #2

Score	Expect	Method	Identities	Positives	Gaps	Frame
59.7 bits(143)	5e-54	Compositional matrix adjust.	27/37(73%)	30/37(81%)	1/37(2%)	+1

```
Query  168    SYMEIAPDIWECWATFYMPYGERAYITW-YSVTQFLI  203
              SYMEI+P +WECWATFY+ YGERAYITW YS   F I
Sbjct  27379  SYMEISPGVWECWATFYLKYGERAYITW*YSFIYFFI  27489
```

Range 3: 26537 to 26758GenBankGraphics

Alignment statistics for match #3

Score	Expect	Method	Identities	Positives	Gaps	Frame
134 bits(338)	1e-33	Compositional matrix adjust.	61/74(82%)	68/74(91%)	0/74(0%)	+2

```
Query  91     ITFRFQGGAVLCKLIKFGQPFGLYLSSYILTVTAFDRYYAICHPFSYCGVTSQRSKMMVY  150
              +  RFQGGAVLCKLIKFGQPFGLYLSSYILTVTA DRY+AIC+PFSYC +TS+RSKMMVY
Sbjct  26537  MAVRFQGGAVLCKLIKFGQPFGLYLSSYILTVTAIDRYHAICYPFSYCSITSRRSKMMVY  26716

Query  151    GAWALAVILCVPQI  164
              GAW LA +LCVPQ+
Sbjct  26717  GAWTLAAMLCVPQV  26758
```

Range 4: 23292 to 23573GenBankGraphics

Alignment statistics for match #4

Score	Expect	Method	Identities	Positives	Gaps	Frame
125 bits(315)	1e-30	Compositional matrix adjust.	80/94(85%)	85/94(90%)	0/94(0%)	+3

```
Query  1      MSRDLNSSSSPSEWPLDDARDEYLARWEIAVLTSIFLLTVIGNIlvllalyvrrryqrrK  60
              MS DLN SSS      DDARDEYLARWEIAVLTSIFL+T+IGNIL+LLALYVRR YQ+RK
Sbjct  23292  MSHDLNFSSSSERPSSDDARDEYLARWEIAVLTSIFLITIIGNILILLALYVRRCYQKRK  23471

Query  61     FTRMYFFILHLSVADLLTGLLDVLPQLAWDITFR  94
              FTRMYFFI+HLSVADLLTGLLDVLPQLAWDITFR
Sbjct  23472  FTRMYFFIMHLSVADLLTGLLDVLPQLAWDITFR  23573
```

Range 5: 28253 to 28411GenBankGraphics

Alignment statistics for match #5

Score	Expect	Method	Identities	Positives	Gaps	Frame
97.8 bits(242)	3e-21	Compositional matrix adjust.	46/53(87%)	47/53(88%)	0/53(0%)	+2

```
Query  292    FTGAAFTILSLLNSLTSCVNPWIYFAFNKELRMALINFLCGKKDYSLTYGNHN  344
              F GAAFTILSLLNSLTSC+NPWIYFAFNKELR AL  F C KKDYSLTYGNHN
Sbjct  28253  FAGAAFTILSLLNSLTSCINPWIYFAFNKELRAALTYFFCRKKDYSLTYGNHN  28411
```

Atta colombica contig24766, whole genome shotgun sequence
Sequence ID: gb|LKEW01024766.1|Length: 24004Number of Matches: 4
Related Information
Range 1: 3747 to 4241GenBankGraphics
Query: Monomorium_pharaonis gi|826425402|ref|XP_012527228.1|

Alignment statistics for match #1

Score	Expect	Method	Identities	Positives	Gaps	Frame
212 bits(539)	5e-60	Compositional matrix adjust.	102/165(62%)	117/165(70%)	38/165(23%)	-3

```
Query  169   YMEIAPDIWECWATFYMPYGERAYITW---------------------------------  195
             YMEI+P +WECWATFY+ YGERAYITW
Sbjct  4241  YMEISPGVWECWATFYLKYGERAYITW*YPFIYIFSLKNILFLYNITFNNINRFKCCEMS  4062

Query  196   -----YSVTQFLIPFIVLIYTYTKICVSIWTSNKISGIVDFKKGNKANFSQRNRDPLISK  250
                  YSV QFL+PFIVL+YTYT+IC+SIW S+K+SG++D KK NK +F QRNRDPLISK
Sbjct  4061  GFNYRYSVMQFLLPFIVLVYTYTRICISIWRSSKLSGLIDLKKSNKVSFCQRNRDPLISK  3882

Query  251   AMINTIKQMIVVVTLYIITNTPFIGCELWATWDPKASSSPFFTGA  295
             AMINT+KQMIVVV LYI+TNTPFIGCELWATWDPKAS+SPFFTG
Sbjct  3881  AMINTVKQMIVVVILYIVTNTPFIGCELWATWDPKASTSPFFTGK  3747
```

Range 2: 4891 to 5103GenBankGraphics

Alignment statistics for match #2

Score	Expect	Method	Identities	Positives	Gaps	Frame
136 bits(343)	5e-34	Compositional matrix adjust.	62/71(87%)	66/71(92%)	0/71(0%)	-2

```
Query  94    RFQGGAVLCKLIKFGQPFGLYLSSYILTVTAFDRYYAICHPFSYCGVTSQRSKMMVYGAW  153
             RFQGGAVLCKLIKFGQPFGLYLSSYILTVTA DRY+AICHPFSYC VTS+RSKMMVYG W
Sbjct  5103  RFQGGAVLCKLIKFGQPFGLYLSSYILTVTAIDRYHAICHPFSYCSVTSRRSKMMVYGTW  4924

Query  154   ALAVILCVPQI  164
              LA +LCVPQ+
Sbjct  4923  MLAAMLCVPQV  4891
```

Range 3: 6822 to 7103GenBankGraphics

Alignment statistics for match #3

Score	Expect	Method	Identities	Positives	Gaps	Frame
125 bits(313)	5e-30	Compositional matrix adjust.	85/95(89%)	89/95(93%)	2/95(2%)	-3

```
Query  1     MSRDLNSSSSPSEWPLDD-ARDEYLARWEIAVLTSIFLLTVIGNIlvllalyvrrryqrr  59
             MS DLNSSSS SE PLDD ARDEYLARWEIAVLTSIFL+T+IGN+LVLLALY RRRYQRR
Sbjct  7103  MSHDLNSSSS-SELPLDDDARDEYLARWEIAVLTSIFLITIIGNVLVLLALYARRRYQRR  6927

Query  60    KFTRMYFFILHLSVADLLTGLLDVLPQLAWDITFR  94
             KFTRMYFFI+HLSVADLLTGL DVLPQLAWDITFR
Sbjct  6926  KFTRMYFFIMHLSVADLLTGLFDVLPQLAWDITFR  6822
```

Range 4: 3235 to 3387GenBankGraphics

Alignment statistics for match #4

Score	Expect	Method	Identities	Positives	Gaps	Frame
96.7 bits(239)	2e-20	Compositional matrix adjust.	46/51(90%)	47/51(92%)	0/51(0%)	-2

```
Query  294   GAAFTILSLLNSLTSCVNPWIYFAFNKELRMALINFLCGKKDYSLTYGNHN  344
             GAAFTILSLLNSLTSCVNPWIYFAFNKELR+AL  F C KKDYSLTYGNHN
Sbjct  3387  GAAFTILSLLNSLTSCVNPWIYFAFNKELRVALTCFFCRKKDYSLTYGNHN  3235
```

Linepithema humile ctg7180000726062, whole genome shotgun sequence
Sequence ID: gb|ADOQ01003105.1|Length: 22838
Number of Matches: 5
Range 1: 12037 to 12333GenBankGraphics
Query: Monomorium_pharaonis gi|826425402|ref|XP_012527228.1|

Alignment statistics for match #1

Score	Expect	Method	Identities	Positives	Gaps	Frame
173 bits(439)	1e-54	Compositional matrix adjust.	79/99(80%)	92/99(92%)	0/99(0%)	+1

```
Query  196    YSVTQFLIPFIVLIYTYTKICVSIWTSNKISGIVDFKKGNKANFSQRNRDPLISKAMINT  255
              YSVTQFL+PFIVL+YTYT+IC+SIWTSN +SG+V FKK +KA+F+ RNRDP ISKA+INT
Sbjct  12037  YSVTQFLLPFIVLVYTYTRICISIWTSNTMSGVVGFKKSDKASFAHRNRDPFISKALINT  12216

Query  256    IKQMIVVVTLYIITNTPFIGCELWATWDPKASSSPFFTG  294
              +KQ IVVVTLYIITN PFIGCELWATWDPKAS+SPFF+G
Sbjct  12217  VKQTIVVVTLYIITNIPFIGCELWATWDPKASTSPFFSG  12333
```

Range 2: 11847 to 11957GenBankGraphics

Alignment statistics for match #2

Score	Expect	Method	Identities	Positives	Gaps	Frame
59.3 bits(142)	1e-54	Compositional matrix adjust.	26/37(70%)	31/37(83%)	2/37(5%)	+3

```
Query  161    VPQ--IFIFSYMEIAPDIWECWATFYMPYGERAYITW  195
              VP+  +FIFSY EI+P +WECWATFY+ YGE AYITW
Sbjct  11847  VPKL*VFIFSYKEISPGVWECWATFYLKYGEAAYITW  11957
```

Range 3: 11290 to 11502GenBankGraphics

Alignment statistics for match #3

Score	Expect	Method	Identities	Positives	Gaps	Frame
135 bits(339)	5e-34	Compositional matrix adjust.	61/71(86%)	65/71(91%)	0/71(0%)	+1

```
Query  94     RFQGGAVLCKLIKFGQPFGLYLSSYILTVTAFDRYYAICHPFSYCGVTSQRSKMMVYGAW  153
              RFQGGA+LCKLIKFGQPFGLYL SYILTVTA DRYYAICHPFSYC VTS+RSKMMV+ AW
Sbjct  11290  RFQGGAILCKLIKFGQPFGLYLGSYILTVTALDRYYAICHPFSYCSVTSRRSKMMVFAAW  11469

Query  154    ALAVILCVPQI  164
              A A ILCVPQ+
Sbjct  11470  AFAAILCVPQV  11502
```

Range 4: 10330 to 10563GenBankGraphics

Alignment statistics for match #4

Score	Expect	Method	Identities	Positives	Gaps	Frame
121 bits(303)	3e-29	Composition-based stats.	69/78(88%)	75/78(96%)	0/78(0%)	+1

```
Query  17     DDARDEYLARWEIAVLTSIFLLTVIGNIlvllalyvrrryqrrKFTRMYFFILHLSVADL  76
              DDARDEYLARWEIAVLTSIFL+T+IGN+++L ALY RRRYQRRKFTRMYFFILHLS+ADL
Sbjct  10330  DDARDEYLARWEIAVLTSIFLITLIGNVMILFALYARRRYQRRKFTRMYFFILHLSIADL  10509

Query  77     LTGLLDVLPQLAWDITFR  94
              LTGL DVLPQLAWDITFR
Sbjct  10510  LTGLFDVLPQLAWDITFR  10563
```

Range 5: 12585 to 12746GenBankGraphics

Alignment statistics for match #5

Score	Expect	Method	Identities	Positives	Gaps	Frame
92.4 bits(228)	2e-19	Compositional matrix adjust.	45/54(83%)	45/54(83%)	0/54(0%)	+3

```
Query  292    FTGAAFTILSLLNSLTSCVNPWIYFAFNKELRMALINFLCGKKDYSLTYGNHNV  345
              F GA FTILSLLNSLTSCVNPWIYFAFNKELR AL NF C KKDYSLT GN N
Sbjct  12585  FPGAIFTILSLLNSLTSCVNPWIYFAFNKELRAALTNFFCRKKDYSLTNGNRNT  12746
```

Camponotus floridanus CamFlo_1.0_4.contig5511, whole genome shotgun sequence
Sequence ID: gb|AEAB01029474.1|Length: 1857
Number of Matches: 2
Range 1: 520 to 816GenBankGraphics
Query: Monomorium_pharaonis gi|826425402|ref|XP_012527228.1|

Alignment statistics for match #1

Score	Expect	Method	Identities	Positives	Gaps	Frame
159 bits(403)	4e-43	Compositional matrix adjust.	69/99(70%)	88/99(88%)	0/99(0%)	-1

```
Query  196  YSVTQFLIPFIVLIYTYTKICVSIWTSNKISGIVDFKKGNKANFSQRNRDPLISKAMINT  255
            YS+ QFL+PFIVL+YTY  IC++IW SNK+SG++D KK NK NFSQ+NR+P +SKAM+NT
Sbjct  816  YSIVQFLLPFIVLVYTYASICIAIWRSNKMSGVIDSKKKNKINFSQQNRNPFMSKAMLNT  637

Query  256  IKQMIVVVTLYIITNTPFIGCELWATWTDPKASSSPFFTG  294
            ++Q I+V+TLYI+T++PFIGCELWATWTDPKA S PFFTG
Sbjct  636  VRQTIIVITLYIVTSSPFIGCELWATWTDPKAFSLPFFTG  520
```

Range 2: 88 to 240GenBankGraphics

Alignment statistics for match #2

Score	Expect	Method	Identities	Positives	Gaps	Frame
84.0 bits(206)	8e-17	Compositional matrix adjust.	39/51(76%)	44/51(86%)	0/51(0%)	-1

```
Query  294  GAAFTILSLLNSLTSCVNPWIYFAFNKELRMALINFLCGKKDYSLTYGNHN  344
            GAAFTILSLLNSLTSCVNPWIYFAFNK+LR AL  F   +K++SL YGNH+
Sbjct  240  GAAFTILSLLNSLTSCVNPWIYFAFNKDLRTALTTFFYRRKNHSLNYGNHD  88
```

Camponotus floridanus CamFlo_1.0_4.contig5512, whole genome shotgun sequence
Sequence ID: gb|AEAB01029475.1|Length: 4331
Number of Matches: 3
Range 1: 1496 to 1714GenBankGraphics
Query: Monomorium_pharaonis gi|826425402|ref|XP_012527228.1|

Alignment statistics for match #1

Score	Expect	Method	Identities	Positives	Gaps	Frame
130 bits(327)	2e-32	Compositional matrix adjust.	60/73(82%)	64/73(87%)	0/73(0%)	-2

```
Query  92    TFRFQGGAVLCKLIKFGQPFGLYLSSYILTVTAFDRYYAICHPFSYCGVTSQRSKMMVYG  151
             T RFQGGAVLCKLIKFGQPFG YLSSY+LTVTA DRY AICHPFSYC  TS+RSK+MVYG
Sbjct  1714  TIRFQGGAVLCKLIKFGQPFGPYLSSYVLTVTAIDRYQAICHPFSYCSNTSRRSKIMVYG  1535

Query  152   AWALAVILCVPQI  164
             AW  A ILCVPQ+
Sbjct  1534  AWVFAAILCVPQV  1496
```

Range 2: 2836 to 3159GenBankGraphicsNext MatchPrevious MatchFirst Match

Alignment statistics for match #2

Score	Expect	Method	Identities	Positives	Gaps	Frame
119 bits(298)	1e-28	Compositional matrix adjust.	77/108(71%)	87/108(80%)	10/108(9%)	-3

```
Query  8     SSSPSEWPLDDARDEYLARWEIAVLTSIFLLTVIGNIlvllalyvrrryqrrKFTRMYFF  67
             S SPS     D+ RDEYLARWEIA+LTSIFL+T+IGN L+L ALYVRRRYQR+KFTRMYFF
Sbjct  3159  SLSPSMQTPDNTRDEYLARWEIAILTSIFLITLIGNTLILFALYVRRRYQRQKFTRMYFF  2980

Query  68    ILHLSVADLLTGLLDVLPQLAWDITFR-----FQG-----GAVLCKLI  105
             ILHLSVADLLTGLLDVLPQLAWDITFR     FQ         ++C+L+
Sbjct  2979  ILHLSVADLLTGLLDVLPQLAWDITFR*IVLPFQSVL*LLNELICRLL  2836
```

Range 3: 1016 to 1099GenBankGraphics

Alignment statistics for match #3

Score	Expect	Method	Identities	Positives	Gaps	Frame
51.2 bits(121)	4e-06	Compositional matrix adjust.	19/28(68%)	24/28(85%)	0/28(0%)	-2

```
Query  168   SYMEIAPDIWECWATFYMPYGERAYITW  195
             SY EI+PD+W+CWATF + YGER Y+TW
Sbjct  1099  SYEEISPDVWDCWATFNLKYGERVYVTW  1016
```

Trachymyrmex septentrionalis contig23603, whole genome shotgun sequence
Sequence ID: gb|LKEZ01023603.1|Length: 6298
Number of Matches: 4
Range 1: 5221 to 5724GenBankGraphics
Query: Monomorium_pharaonis gi|826425402|ref|XP_012527228.1|

Alignment statistics for match #1

Score	Expect	Method	Identities	Positives	Gaps	Frame
222 bits(565)	2e-63	Compositional matrix adjust.	107/168(64%)	122/168(72%)	37/168(22%)	+1

```
Query  164   IFIFSYMEIAPDIWECWATFYMPYGERAYITW----------------------------  195
             IFIFSYMEI+P +WECWATFY+ YGERAYITW
Sbjct  5221  IFIFSYMEISPGVWECWATFYLKYGERAYITW*DLFIYFFIQKYLIFIT*HLITLIRFKC  5400

Query  196   ---------YSVTQFLIPFIVLIYTYTKICVSIWTSNKISGIVDFKKGNKANFSQRNRDP  246
                      YSV QFL+PFIVL+YTYT+IC+SIW S K+SG++D K+ NK +F QRNRDP
Sbjct  5401  CKMSGFNYRYSVMQFLLPFIVLVYTYTRICISIWRSGKLSGLIDLKRSNKVSFCQRNRDP  5580

Query  247   LISKAMINTIKQMIVVVTLYIITNTPFIGCELWATWDPKASSSPFFTG  294
             LISKAMINT+KQMIVVVTLYI+TNTPFIGCELWATWDPKAS+SPFFTG
Sbjct  5581  LISKAMINTVKQMIVVVTLYIVTNTPFIGCELWATWDPKASTSPFFTG  5724
```

Range 2: 4405 to 4617GenBankGraphics

Alignment statistics for match #2

Score	Expect	Method	Identities	Positives	Gaps	Frame
137 bits(345)	4e-34	Compositional matrix adjust.	62/71(87%)	66/71(92%)	0/71(0%)	+1

```
Query  94    RFQGGAVLCKLIKFGQPFGLYLSSYILTVTAFDRYYAICHPFSYCGVTSQRSKMMVYGAW  153
             RFQGGAVLCKLIKFGQPFGLYLSSYILTVTA DRY+AICHPFSYC VTS+RSKMMVYG W
Sbjct  4405  RFQGGAVLCKLIKFGQPFGLYLSSYILTVTAIDRYHAICHPFSYCSVTSRRSKMMVYGTW  4584

Query  154   ALAVILCVPQI  164
              LA +LCVPQ+
Sbjct  4585  TLAAMLCVPQV  4617
```

Range 3: 2497 to 2769GenBankGraphics

Alignment statistics for match #3

Score	Expect	Method	Identities	Positives	Gaps	Frame
131 bits(330)	4e-32	Compositional matrix adjust.	82/92(89%)	87/92(94%)	2/92(2%)	+1

```
Query  4     DLNSSSSPSEWPLDD-ARDEYLARWEIAVLTSIFLLTVIGNIlvllalyvrrryqrrKFT  62
             DLNSSSS SE PLDD ARDEYLARWEIAVLTSIFL+T++GN+LVLLALY RRRYQRRKFT
Sbjct  2497  DLNSSSS-SELPLDDDARDEYLARWEIAVLTSIFLITIVGNVLVLLALYARRRYQRRKFT  2673

Query  63    RMYFFILHLSVADLLTGLLDVLPQLAWDITFR  94
             RMYFFI+HLSVADLLTGL DVLPQLAWDITFR
Sbjct  2674  RMYFFIMHLSVADLLTGLFDVLPQLAWDITFR  2769
```

Range 4: 6089 to 6241GenBankGraphics

Alignment statistics for match #4

Score	Expect	Method	Identities	Positives	Gaps	Frame
96.7 bits(239)	3e-20	Compositional matrix adjust.	46/51(90%)	47/51(92%)	0/51(0%)	+2

```
Query  294   GAAFTILSLLNSLTSCVNPWIYFAFNKELRMALINFLCGKKDYSLTYGNHN  344
             GAAFTILSLLNSLTSCVNPWIYFAFNKELR+AL  F C KKDYSLTYGNHN
Sbjct  6089  GAAFTILSLLNSLTSCVNPWIYFAFNKELRVALTCFFCRKKDYSLTYGNHN  6241
```

Trachymyrmex zeteki contig15596, whole genome shotgun sequence
Sequence ID: gb|LKFA01015596.1|Length: 111526
Number of Matches: 5
Range 1: 16865 to 17161GenBankGraphics
Query: Monomorium_pharaonis gi|826425402|ref|XP_012527228.1|

Alignment statistics for match #1

Score	Expect	Method	Identities	Positives	Gaps	Frame
175 bits(443)	3e-54	Compositional matrix adjust.	78/99(79%)	91/99(91%)	0/99(0%)	-1

```
Query  196    YSVTQFLIPFIVLIYTYTKICVSIWTSNKISGIVDFKKGNKANFSQRNRDPLISKAMINT  255
              YSV QFL+PFIVL+YTYT+IC+SIW S+K+SG++D K+ NK +F QRNRDPLISKAMINT
Sbjct  17161  YSVMQFLLPFIVLVYTYTRICISIWRSSKLSGLIDLKRSNKVSFCQRNRDPLISKAMINT  16982

Query  256    IKQMIVVVTLYIITNTPFIGCELWATWDPKASSSPFFTG  294
              +KQMIVVVTLYI+TN PFIGCELWATWDPKAS+SPFF G
Sbjct  16981  VKQMIVVVTLYIVTNIPFIGCELWATWDPKASNSPFFAG  16865
```

Range 2: 17257 to 17337GenBankGraphics

Alignment statistics for match #2

Score	Expect	Method	Identities	Positives	Gaps	Frame
57.8 bits(138)	3e-54	Compositional matrix adjust.	22/27(81%)	25/27(92%)	0/27(0%)	-2

```
Query  169    YMEIAPDIWECWATFYMPYGERAYITW  195
              YMEI+P +WECWATFY+ YGERAYITW
Sbjct  17337  YMEISPGVWECWATFYLKYGERAYITW  17257
```

Range 3: 17954 to 18166GenBankGraphics

Alignment statistics for match #3

Score	Expect	Method	Identities	Positives	Gaps	Frame
138 bits(348)	2e-34	Compositional matrix adjust.	63/71(89%)	67/71(94%)	0/71(0%)	-1

```
Query  94     RFQGGAVLCKLIKFGQPFGLYLSSYILTVTAFDRYYAICHPFSYCGVTSQRSKMMVYGAW  153
              RFQGGAVLCKLIKFGQPFGLYLSSYILTVTA DRY+AICHPFSYC VTS+RSKMMVYGAW
Sbjct  18166  RFQGGAVLCKLIKFGQPFGLYLSSYILTVTAIDRYHAICHPFSYCSVTSRRSKMMVYGAW  17987

Query  154    ALAVILCVPQI  164
               LA +LCVPQ+
Sbjct  17986  TLAAMLCVPQV  17954
```

Range 4: 19839 to 20126GenBankGraphics

Alignment statistics for match #4

Score	Expect	Method	Identities	Positives	Gaps	Frame
123 bits(308)	3e-29	Compositional matrix adjust.	83/96(86%)	88/96(91%)	2/96(2%)	-3

```
Query  1      MSRDLNSSSSPS-EWPLDD-ARDEYLARWEIAVLTSIFLLTVIGNIlvllalyvrrryqr  58
              MS DLNSSSS   E PLDD ARDE+LARWEIAVLTSIFL+T+IGN+LVLLALY RRRYQR
Sbjct  20126  MSHDLNSSSSSFSERPLDDDARDEHLARWEIAVLTSIFLITIIGNVLVLLALYARRRYQR  19947

Query  59     rKFTRMYFFILHLSVADLLTGLLDVLPQLAWDITFR  94
              RKFTRMYFFI+HLSVADLLTGL DVLPQLAWDITFR
Sbjct  19946  RKFTRMYFFIMHLSVADLLTGLFDVLPQLAWDITFR  19839
```

Range 5: 16354 to 16515GenBankGraphics

Alignment statistics for match #5

Score	Expect	Method	Identities	Positives	Gaps	Frame
100 bits(249)	2e-21	Compositional matrix adjust.	48/54(89%)	49/54(90%)	0/54(0%)	-2

```
Query  294    GAAFTILSLLNSLTSCVNPWIYFAFNKELRMALINFLCGKKDYSLTYGNHNVSL  347
              GAAFTILSLLNSLTSCVNPWIYFAFNKELR AL +F C KKDYSLTYGNHN SL
Sbjct  16515  GAAFTILSLLNSLTSCVNPWIYFAFNKELRAALTHFFCRKKDYSLTYGNHNFSL  16354
```

Trachymyrmex cornetzi contig27754, whole genome shotgun sequence
Sequence ID: gb|LKEY01027754.1|Length: 9614
Number of Matches: 5
Range 1: 8717 to 9013GenBankGraphics
Query: Monomorium_pharaonis gi|826425402|ref|XP_012527228.1|

Alignment statistics for match #1

Score	Expect	Method	Identities	Positives	Gaps	Frame
180 bits(456)	9e-49	Compositional matrix adjust.	81/99(82%)	93/99(93%)	0/99(0%)	+2

```
Query  196   YSVTQFLIPFIVLIYTYTKICVSIWTSNKISGIVDFKKGNKANFSQRNRDPLISKAMINT  255
             YSV QFL+PFIVL+YTYT+IC+SIW SNK+SG++D K+ NK +F QRNRDPLISKAMINT
Sbjct  8717  YSVMQFLLPFIVLVYTYTRICISIWRSNKLSGLIDLKRNNKVSFCQRNRDPLISKAMINT  8896

Query  256   IKQMIVVVTLYIITNTPFIGCELWATWDPKASSSPFFTG  294
             +KQMIVVVTLYI+TNTPFIGCELWATWDPKAS+SPFFTG
Sbjct  8897  VKQMIVVVTLYIVTNTPFIGCELWATWDPKASTSPFFTG  9013
```

Range 2: 7686 to 7898GenBankGraphics

Alignment statistics for match #2

Score	Expect	Method	Identities	Positives	Gaps	Frame
138 bits(348)	2e-34	Compositional matrix adjust.	63/71(89%)	67/71(94%)	0/71(0%)	+3

```
Query  94    RFQGGAVLCKLIKFGQPFGLYLSSYILTVTAFDRYYAICHPFSYCGVTSQRSKMMVYGAW  153
             RFQGGAVLCKLIKFGQPFGLYLSSYILTVTA DRY+AICHPFSYC VTS+RSKMMVYGAW
Sbjct  7686  RFQGGAVLCKLIKFGQPFGLYLSSYILTVTAIDRYHAICHPFSYCSVTSRRSKMMVYGAW  7865

Query  154   ALAVILCVPQI  164
              LA +LCVPQ+
Sbjct  7866  TLAAMLCVPQV  7898
```

Range 3: 5839 to 6120GenBankGraphics

Alignment statistics for match #3

Score	Expect	Method	Identities	Positives	Gaps	Frame
126 bits(316)	3e-30	Compositional matrix adjust.	85/95(89%)	89/95(93%)	2/95(2%)	+1

```
Query  1     MSRDLNSSSSPSEWPLDD-ARDEYLARWEIAVLTSIFLLTVIGNIlvllalyvrrryqrr  59
             MS DLNSSSS SE PLDD ARDEYLARWEIAVLTSIFL+T+IGN+LVLLALY RRRYQRR
Sbjct  5839  MSHDLNSSSS-SELPLDDDARDEYLARWEIAVLTSIFLITIIGNVLVLLALYARRRYQRR  6015

Query  60    KFTRMYFFILHLSVADLLTGLLDVLPQLAWDITFR  94
             KFTRMYFFI+HLSVADLLTGL DVLPQLAWDITFR
Sbjct  6016  KFTRMYFFIMHLSVADLLTGLFDVLPQLAWDITFR  6120
```

Range 4: 9426 to 9578GenBankGraphics

Alignment statistics for match #4

Score	Expect	Method	Identities	Positives	Gaps	Frame
94.0 bits(232)	2e-19	Compositional matrix adjust.	45/51(88%)	47/51(92%)	0/51(0%)	+3

```
Query  294   GAAFTILSLLNSLTSCVNPWIYFAFNKELRMALINFLCGKKDYSLTYGNHN  344
             GAAFTILSLLNSLTSCVNPWIYFAFNKELR+AL  F C KKDYSLTYGN+N
Sbjct  9426  GAAFTILSLLNSLTSCVNPWIYFAFNKELRVALTCFFCRKKDYSLTYGNYN  9578
```

Range 5: 8524 to 8658GenBankGraphicsNext MatchPrevious MatchFirst Match

Alignment statistics for match #5

Score	Expect	Method	Identities	Positives	Gaps	Frame
55.8 bits(133)	5e-07	Compositional matrix adjust.	27/45(60%)	32/45(71%)	1/45(2%)	+1

```
Query  169   YMEIAPDIWECWATFYMPYGERAYITW-YSVTQFLIPFIVLIYTY  212
             YMEI+P +WECWATFY+ YGERAYITW Y    F I   ++  TY
Sbjct  8524  YMEISPGVWECWATFYLKYGERAYITW*YPFIYFFIKKYLIFITY  8658
```

Nasonia longicornis strain IV7(U) Contig169224, whole genome shotgun sequence
Sequence ID: gb|ADAP01169223.1|Length: 6826
Number of Matches: 2
Range 1: 5931 to 6248GenBankGraphics
Query: Monomorium_pharaonis gi|826425402|ref|XP_012527228.1|

Alignment statistics for match #1

Score	Expect	Method	Identities	Positives	Gaps	Frame
113 bits(282)	4e-26	Compositional matrix adjust.	53/106(50%)	72/106(67%)	0/106(0%)	-3

```
Query  189   ERAYITWYSVTQFLIPFIVLIYTYTKICVSIWTSNKISGIVDFKKGNKANFSQRNRDPLI  248
             + AY+  YSVT FL+PF VL +TY +IC SIW + ++  +   ++         R++  LI
Sbjct  6248  DNAYLYRYSVTVFLLPFCVLTFTYAEICCSIWRNREVMVLASHERQQALTKEGRSQTTLI  6069

Query  249   SKAMINTIKQMIVVVTLYIITNTPFIGCELWATWDPKASSSPFFTG  294
             SKA INT+KQ + VVTLY  ++ PF+GC+LWATWDP ASSS FF G
Sbjct  6068  SKAKINTVKQTLAVVTLYAASSIPFVGCQLWATWDPFASSSAFFDG  5931
```

Range 2: 5200 to 5367GenBankGraphics

Alignment statistics for match #2

Score	Expect	Method	Identities	Positives	Gaps	Frame
44.3 bits(103)	0.002	Compositional matrix adjust.	31/56(55%)	37/56(66%)	2/56(3%)	-2

```
Query  291   FFTGAAFTILSLLNSLTSCVNPWIYFAFNKELRMALINFLCG--KKDYSLTYGNHN  344
             F  G  FTILSLL+SLTSCVNPWIY  F+ ELR AL  FL    K+D +  +G +
Sbjct  5367  FLLGPIFTILSLLSSLTSCVNPWIYLTFSYELRAALTKFLRSLIKRDRTSRFGKYQ  5200
```

Nasonia longicornis strain IV7(U) Contig169231, whole genome shotgun sequence
Sequence ID: gb|ADAP01169230.1|Length: 1848
Number of Matches: 1
Range 1: 602 to 919GenBankGraphics
Query: Monomorium_pharaonis gi|826425402|ref|XP_012527228.1|

Alignment statistics for match #1

Score	Expect	Method	Identities	Positives	Gaps	Frame
89.7 bits(221)	2e-18	Compositional matrix adjust.	57/107(53%)	80/107(74%)	2/107(1%)	-3

```
Query  6    NSSSSPSEWPLDDARDEYLARWEIAVLTSIFLLTVIGNIlvllalyvrr-ryqrrKFTRM  64
             S++S    P +D RDE LA WE+ VL  I   T++GN+LVL A+Y++R R +R++ TRM
Sbjct  919  ESATSMVAAPAEDWRDESLAVWEVIVLVLILTTTLMGNVLVLFAIYLKRCRGRRQRLTRM  740

Query  65   YFFILHLSVADLLTGLLDVLPQLAWDITFRFQGGAVLCKLIKFGQPF  111
            +FF++HLSVADL+TGLL+VLPQLAWD+TFR+     L ++IKF + F
Sbjct  739  HFFVMHLSVADLITGLLNVLPQLAWDVTFRYVYAHDL-QIIKFKKLF  602
```

Neodiprion lecontei scaffold_555, whole genome shotgun sequence
Sequence ID: gb|LGIB01000555.1|Length: 122789
Number of Matches: 4
Range 1: 54570 to 55106GenBankGraphics
Query: Polistes_canadensis gi|954548184|ref|XP_014616661.1|

Alignment statistics for match #1

Score	Expect	Method	Identities	Positives	Gaps	Frame
108 bits(269)	2e-24	Compositional matrix adjust.	68/185(37%)	93/185(50%)	46/185(24%)	+3

```
Query  195    YSLMVFLLPFIVLVYTYVGICVGVWQSNKMSGAVDDRAyirsisnysrnrssfisKAMIN  254
              YS+ +F+LP +VLVYTY  IC  VW +  + G   D       +     + + IS+A IN
Sbjct  54570  YSISIFILPLLVLVYTYASICFAVWNNTGIIGEARD------TNGGPLRQRAIISRAKIN  54731

Query  255    TVKQTIAVITLYAITSIPFIGCELWVSWDTKAFES------------------------  289
              ++KQ IAVI+ YA++S PFI   LWV+WD  A  S
Sbjct  54732  SIKQMIAVISFYAVSSSPFIASLLWVTWDRNAPTSPYFDGTLYSKL*FHWFNKLYL*YQK  54911

Query  290    -----------SFANGPAFTILALLNSLTSCVNPWIYLSFNRELRQTLMNYFC----NRN  334
                         +F  G AF IL L++SL SCVNPWIYL+ NREL + L++  C    NR+
Sbjct  54912  VLRIRASV*STNFIAGAAFAILTLMSSLNSCVNPWIYLALNRELWRALIDRVCLSRSNRD  55091

Query  335    EHSQK  339
              E  +
Sbjct  55092  ETRKN  55106
```

Range 2: 53667 to 53882GenBankGraphics

Alignment statistics for match #2

Score	Expect	Method	Identities	Positives	Gaps	Frame
106 bits(265)	7e-24	Compositional matrix adjust.	49/72(68%)	58/72(80%)	0/72(0%)	+3

```
Query  92     FRFQGGLVLCKLVKFFQPFGLYLSSYILTATAIDRYHAICHPLSYCSVTSRQSRIMVYCA  151
              +RFQGG +LCKLVKF QP G Y SSYILTATA+DRY A+CHPL+Y S TSR+S+I V  A
Sbjct  53667  YRFQGGPILCKLVKFGQPLGPYSSSYILTATALDRYKAVCHPLAYSSFTSRRSKITVCLA  53846

Query  152    WSFALILCIPQL  163
              WS +L  CIPQ+
Sbjct  53847  WSLSLAFCIPQV  53882
```

Range 3: 53329 to 53697GenBankGraphics

Alignment statistics for match #3

Score	Expect	Method	Identities	Positives	Gaps	Frame
102 bits(253)	2e-22	Compositional matrix adjust.	59/130(45%)	84/130(64%)	8/130(6%)	+1

```
Query  1      MNETTNSSFTVANEEKDTRDENLAKWEIVLLANILIFTIFGNSLVLLSLYLRKYRAHKK-  59
              +N TT ++    N++ D RDE LA WEI  LA + +  + GNSLVLL++YLR Y A KK
Sbjct  53329  LNGTTENT----NKKDDPRDEYLASWEIATLAAMFLAALAGNSLVLLAIYLRGYDAPKKN  53496

Query  60     LSRMYFFIMNLSIADLLTGLLDVLPQLAWDITFRFQGGLVLCKLVKFFQPFGLYLSSYIL  119
              L+RM+ FI++LS+ADLLT +L+V PQLAW+ITFR+     +          G YL+ +I+
Sbjct  53497  LTRMHLFILHLSVADLLTAILNVFPQLAWEITFRYNN---INYTASIIHTTGFYLTRWIV  53667

Query  120    TATAIDRYHA  129
              T   + R+ A
Sbjct  53668  TGFKVGRFFA  53697
```

Range 4: 54182 to 54316GenBankGraphics

Alignment statistics for match #4

Score	Expect	Method	Identities	Positives	Gaps	Frame
65.9 bits(159)	1e-10	Compositional matrix adjust.	30/45(67%)	33/45(73%)	0/45(0%)	+2

```
Query  150    CAWSFALILCIPQLFVFSYQEVMPRIWDCWATFDIQFGEKAYVTW  194
              C  +F LI  I QL VFSYQEV P IWDCWATF+   GE+AYVTW
Sbjct  54182  CRETFPLITIIRQLIVFSYQEVSPDIWDCWATFNPPSGERAYVTW  54316
```

Diachasma alloeum Dall_scaffold_00798_contig13, whole genome shotgun sequence
Sequence ID: gb|LDKA01015940.1|Length: 53672Number of Matches: 2
Related Information
Range 1: 50694 to 50906GenBankGraphics
Query: Polistes_canadensis gi|954548184|ref|XP_014616661.1|

Alignment statistics for match #1

Score	Expect	Method	Identities	Positives	Gaps	Frame
106 bits(265)	9e-24	Compositional matrix adjust.	45/71(63%)	56/71(78%)	0/71(0%)	+3

```
Query  93     RFQGGLVLCKLVKFFQPFGLYLSSYILTATAIDRYHAICHPLSYCSVTSRQSRIMVYCAW  152
              RFQGG VLCK+VKF QP G Y S+Y+L A AIDRYHAIC+P +YC  T +Q+++MVY AW
Sbjct  50694  RFQGGAVLCKMVKFGQPLGHYASTYVLIAGAIDRYHAICYPFNYCRTTHKQAKVMVYIAW  50873

Query  153    SFALILCIPQL  163
                +L LCIPQ+
Sbjct  50874  GISLALCIPQV  50906
```

Range 2: 48177 to 48419GenBankGraphics

Alignment statistics for match #2

Score	Expect	Method	Identities	Positives	Gaps	Frame
87.0 bits(214)	3e-17	Compositional matrix adjust.	51/81(63%)	65/81(80%)	1/81(1%)	+3

```
Query  12     ANEEKDTRDENLAKWEIVLLANILIFTIFGNSLVLLSLYLRKY-RAHKKLSRMYFFIMNL  70
              +N  +D RDE LA WEIV+L  IL+ T+ GN LVL +LYLR+Y    +KL+RMYFF+M+L
Sbjct  48177  SNMTEDPRDEFLAVWEIVILLVILVITVVGNFLVLFALYLRRYCGRRRKLTRMYFFMMHL  48356

Query  71     SIADLLTGLLDVLPQLAWDIT  91
              SIADL+TG+L+VLPQL WDIT
Sbjct  48357  SIADLMTGVLNVLPQLVWDIT  48419
```

Diachasma alloeum Dall_scaffold_00798_contig14, whole genome shotgun sequence
Sequence ID: gb|LDKA01015941.1|Length: 26306
Number of Matches: 3
Range 1: 813 to 1109GenBankGraphics
Query: Polistes_canadensis gi|954548184|ref|XP_014616661.1|

Alignment statistics for match #1

Score	Expect	Method	Identities	Positives	Gaps	Frame
83.6 bits(205)	5e-27	Compositional matrix adjust.	44/100(44%)	66/100(66%)	1/100(1%)	+3

```
Query  195  YSLMVFLLPFIVLVYTYVGICVGVWQSNKMSGAVDDRAyirsisnysrnrssfisKAMIN  254
            YS+ V LLPF VLVYTYV +C  +W++ +++      R  ++ + +   NR   IS+A I
Sbjct  813  YSITVLLLPFSVLVYTYVSVCAEIWKNTEIT-VFGHRGEVKRMDSRENNREPVISRARIK  989

Query  255  TVKQTIAVITLYAITSIPFIGCELWVSWDTKAFESSFANG  294
            TVKQ I V++L+ ITS PF+GC+LW ++D  A ++SF  G
Sbjct  990  TVKQMITVVSLHVITSAPFVGCQLWAAFDPDAVKTSFYEG  1109
```

Range 2: 625 to 723GenBankGraphics

Alignment statistics for match #2

Score	Expect	Method	Identities	Positives	Gaps	Frame
57.0 bits(136)	5e-27	Compositional matrix adjust.	22/33(67%)	27/33(81%)	0/33(0%)	+1

```
Query  162  QLFVFSYQEVMPRIWDCWATFDIQFGEKAYVTW  194
            Q+F+FSYQE+ PRIWDCWA F    G++AYVTW
Sbjct  625  QIFIFSYQEISPRIWDCWADFTPPHGQRAYVTW  723
```

Range 3: 2519 to 2698GenBankGraphics

Alignment statistics for match #3

Score	Expect	Method	Identities	Positives	Gaps	Frame
51.2 bits(121)	8e-06	Compositional matrix adjust.	32/60(53%)	38/60(63%)	0/60(0%)	+2

```
Query  294   GPAFTILALLNSLTSCVNPWIYLSFNRELRQTLMNYFCNRNEHSQKYYGQRMMRRSDSND  353
             GPAF IL+LL+SLTSCVNPWIY+ FNRELR TL  Y           +G+   + SD  D
Sbjct  2519  GPAFAILSLLSSLTSCVNPWIYMGFNRELRSTLKKYIKKLILGRSMTFGESKHQNSDITD  2698
```

Cephus cinctus Ccin1_scaffold0013_contig28, whole genome shotgun sequence
Sequence ID: gb|AMWH01000205.1|Length: 159119
Number of Matches: 4
Range 1: 118317 to 118703GenBankGraphics
Query: Polistes_canadensis gi|954548184|ref|XP_014616661.1|

Alignment statistics for match #1

Score	Expect	Method	Identities	Positives	Gaps	Frame
158 bits(400)	6e-88	Compositional matrix adjust.	76/129(59%)	89/129(68%)	25/129(19%)	-1

```
Query  91      TFRFQGGLVLCKLVKFFQPFGLYLSSYILTATAIDRYHAICHPLSYCSVTSRQSRIMVYC  150
               + RFQGG +LCKLVK+ QP G YLSSY+LT TA+DRYHAIC+PLSYC  TSRQSR+MVYC
Sbjct  118703  SIRFQGGALLCKLVKYGQPLGPYLSSYVLTVTALDRYHAICYPLSYCGTTSRQSRVMVYC  118524

Query  151     AWSFALILCIP-------------------------QLFVFSYQEVMPRIWDCWATFDIQ  185
               AW  AL  C P                         QLFVFSYQEVMP ++DCWATF++
Sbjct  118523  AWVIALTFCTPQVCQSF*VAF*FSIDISLELCPLCGQLFVFSYQEVMPGVFDCWATFELS  118344

Query  186     FGEKAYVTW  194
               +GE+AYVTW
Sbjct  118343  YGEQAYVTW  118317
```

Range 2: 118784 to 119044GenBankGraphics

Alignment statistics for match #2

Score	Expect	Method	Identities	Positives	Gaps	Frame
112 bits(279)	6e-88	Compositional matrix adjust.	58/89(65%)	69/89(77%)	3/89(3%)	-2

```
Query  6       NSSFTVANEEKDTRDENLAKWEIVLLANILIFTIFGNSLVLLSLYLRKYR-AHKKLSRMY  64
               N +F   NE  DTRDE LA+WEI  L++IL  T+ GN  VL +LYLR+Y  + KKL+RMY
Sbjct  119044  NKTFEEVNE--DTRDEYLAQWEITTLSSILALTLVGNIFVLFALYLRRYSISKKKLTRMY  118871

Query  65      FFIMNLSIADLLTGLLDVLPQLAWDITFR  93
               FFI++LSIADLLTGL  VLPQLAWDITFR
Sbjct  118870  FFIIHLSIADLLTGLFSVLPQLAWDITFR  118784
```

Range 3: 117938 to 118246GenBankGraphics

Alignment statistics for match #3

Score	Expect	Method	Identities	Positives	Gaps	Frame
94.4 bits(233)	6e-88	Compositional matrix adjust.	53/103(51%)	72/103(69%)	3/103(2%)	-2

```
Query  195     YSLMVFLLPFIVLVYTYVGICVGVWQSNKMSGAVD---DRAyirsisnysrnrssfisKA  251
               YS  VFLLP  VL+YTY GIC+G+W+++ + G  +   + + +    N+ RNR+  IS+A
Sbjct  118246  YSFSVFLLPLCVLLYTYTGICMGIWRNSNVDGLTEIGANGSLLEKNQNHFRNRTLLISRA  118067

Query  252     MINTVKQTIAVITLYAITSIPFIGCELWVSWDTKAFESSFANG  294
                INT+KQTIAVI+LY I S PFIGC+LW SWD +A  S F +G
Sbjct  118066  RINTLKQTIAVISLYIICSSPFIGCQLWASWDPQASSSPFYSG  117938
```

Range 4: 117681 to 117782GenBankGraphics

Alignment statistics for match #4

Score	Expect	Method	Identities	Positives	Gaps	Frame
56.6 bits(135)	7e-08	Compositional matrix adjust.	26/34(76%)	30/34(88%)	0/34(0%)	-1

```
Query  294     GPAFTILALLNSLTSCVNPWIYLSFNRELRQTLM  327
               G  FTILALL+SL SCVNPWIYL+FN ELR+TL+
Sbjct  117782  GTTFTILALLSSLNSCVNPWIYLAFNNELRRTLI  117681
```

Trichogramma pretiosum Contig3858, whole genome shotgun sequence
Sequence ID: gb|JARR01003858.1|Length: 67335
Number of Matches: 4
Range 1: 28185 to 28532GenBankGraphics
Query: Polistes_canadensis gi|954548184|ref|XP_014616661.1|

Alignment statistics for match #1

Score	Expect	Method	Identities	Positives	Gaps	Frame
163 bits(413)	8e-62	Compositional matrix adjust.	76/119(64%)	92/119(77%)	3/119(2%)	+3

```
Query  93     RFQGGLVLCKLVKFFQPFGLYLSSYILTATAIDRYHAICHPLSYCSVTSRQSRIMVYCAW  152
              RF GG +LCKLVKF QP G YLSSY+L ATA DRYHAIC+PLSYC  TSR+SRI VY AW
Sbjct  28185  RFHGGEILCKLVKFGQPLGSYLSSYVLMATAADRYHAICYPLSYCRTTSRRSRITVYTAW  28364

Query  153    SFALILCIPQLFVFSYQEVMPRIWDCWATFDIQFGEKAYVTWYSLMVFLLPFIVLVYTY  211
                +L+LC+PQ+F+FS QE+ P IWDCWATFD+ + +KAYVTW   +VF   F++   TY
Sbjct  28365  ILSLLLCLPQIFIFSIQEIAPGIWDCWATFDLPYAKKAYVTW---LVFKKIFVIACNTY  28532
```

Range 2: 27862 to 28101GenBankGraphics

Alignment statistics for match #2

Score	Expect	Method	Identities	Positives	Gaps	Frame
92.8 bits(229)	8e-62	Compositional matrix adjust.	48/80(60%)	67/80(83%)	1/80(1%)	+1

```
Query  15     EKDTRDENLAKWEIVLLANILIFTIFGNSLVLLSLYLRKYRAHKK-LSRMYFFIMNLSIA  73
              E+D RD +L K E+++L+ I + T+ GNSLVL ++YLR+ R  ++ L+RM+FF+M+LSIA
Sbjct  27862  EEDERDNDLVKVELLVLSLIFLVTLMGNSLVLFAIYLRRCRGRRQRLTRMHFFVMHLSIA  28041

Query  74     DLLTGLLDVLPQLAWDITFR  93
              DL+TGLL+VLPQLAWDITFR
Sbjct  28042  DLITGLLNVLPQLAWDITFR  28101
```

Range 3: 29159 to 29452GenBankGraphics

Alignment statistics for match #3

Score	Expect	Method	Identities	Positives	Gaps	Frame
88.6 bits(218)	4e-18	Compositional matrix adjust.	48/100(48%)	69/100(69%)	2/100(2%)	+2

```
Query  195    YSLMVFLLPFIVLVYTYVGICVGVWQSNKMSGAVDDRAyirsisnysrnrssfisKAMIN  254
              +S+M+FLLPF VL +TYV IC+ +W + ++  A+ +          +RN+ S ISKA IN
Sbjct  29159  FSIMIFLLPFSVLTFTYVEICLSIWHNREV--AMLESQQALQPQIRARNQGSLISKAKIN  29332

Query  255    TVKQTIAVITLYAITSIPFIGCELWVSWDTKAFESSFANG  294
              T+KQT+AV+ LYA +S+PFIG +LW +WD  A ES+F  G
Sbjct  29333  TIKQTLAVVILYAASSMPFIGGQLWSAWDPYAAESAFFKG  29452
```

Range 4: 29709 to 29822GenBankGraphics

Alignment statistics for match #4

Score	Expect	Method	Identities	Positives	Gaps	Frame
43.1 bits(100)	0.001	Compositional matrix adjust.	26/38(68%)	32/38(84%)	0/38(0%)	+3

```
Query  292    ANGPAFTILALLNSLTSCVNPWIYLSFNRELRQTLMNY  329
               +GP FTIL+LL+SLTSCVNPWIYL+F+ ELR  LM +
Sbjct  29709  CSGPTFTILSLLSSLTSCVNPWIYLTFSYELRIVLMKF  29822
```

Copidosoma floridanum Contig51734, whole genome shotgun sequence

Sequence ID: gb|JBOX01051729.1|Length: 2154

Number of Matches: 4

Range 1: 1652 to 1864GenBankGraphics

Query: Polistes_canadensis gi|954548184|ref|XP_014616661.1|

Alignment statistics for match #1

Score	Expect	Method	Identities	Positives	Gaps	Frame
116 bits(291)	3e-27	Compositional matrix adjust.	53/71(75%)	60/71(84%)	0/71(0%)	-3

```
Query  93    RFQGGLVLCKLVKFFQPFGLYLSSYILTATAIDRYHAICHPLSYCSVTSRQSRIMVYCAW  152
             RF+GG VLCK+VKF QP G YLSSYIL ATA+DRYHAIC PLSYC  TSR+SR+MVY AW
Sbjct  1864  RFKGGDVLCKMVKFGQPLGSYLSSYILIATAVDRYHAICFPLSYCRTTSRRSRVMVYSAW  1685

Query  153   SFALILCIPQL  163
               ALILC+PQ+
Sbjct  1684  VLALILCVPQV  1652
```

Range 2: 980 to 1282GenBankGraphics

Alignment statistics for match #2

Score	Expect	Method	Identities	Positives	Gaps	Frame
79.7 bits(195)	3e-22	Compositional matrix adjust.	47/101(47%)	70/101(69%)	1/101(0%)	-3

```
Query  195   YSLMVFLLPFIVLVYTYVGICVGVWQSNKMSGAVD-DRAyirsisnysrnrssfisKAMI  253
             YSL VFL+PF VL +TY  IC  +W++  +    + ++   +++SN +RN+ + ISKA +
Sbjct  1282  YSLTVFLVPFSVLSFTYTRICCSIWRNRDVLVLGNLEKQQQQALSNQARNQHALISKAKM  1103

Query  254   NTVKQTIAVITLYAITSIPFIGCELWVSWDTKAFESSFANG  294
             +TVKQT+AV+ LYA +S+PFIGC+LW +W   A ES F  G
Sbjct  1102  STVKQTLAVVILYAASSLPFIGCQLWATWVPGAAESPFYTG  980
```

Range 3: 1404 to 1514GenBankGraphics

Alignment statistics for match #3

Score	Expect	Method	Identities	Positives	Gaps	Frame
45.1 bits(105)	3e-22	Compositional matrix adjust.	20/38(53%)	27/38(71%)	1/38(2%)	-2

```
Query  157   ILCIPQLFVFSYQEVMPRIWDCWATFDIQFGEKAYVTW  194
             I+C+ Q+F+FS + V   +WDCWATF    G+K YVTW
Sbjct  1514  IICVFQVFIFSLRIVSAGVWDCWATFT-GHGQKIYVTW  1404
```

Range 4: 757 to 978GenBankGraphics

Alignment statistics for match #4

Score	Expect	Method	Identities	Positives	Gaps	Frame
50.4 bits(119)	1e-05	Compositional matrix adjust.	36/74(49%)	48/74(64%)	9/74(12%)	-1

```
Query  276   CELWVSWDTKAFESSFAN----GPAFTILALLNSLTSCVNPWIYLSFNRELRQTLMNYFC  331
             C ++    ++ F  S+A     GPAFTIL+LL+SLTSCVNPWIYL F+RELR +L+ +
Sbjct  978   CCVFSYLQSRIFSISYARVLCAGPAFTILSLLSSLTSCVNPWIYLVFSRELRVSLIKFLH  799

Query  332   -----NRNEHSQKY  340
                  NR+  S KY
Sbjct  798   HLVKRNRSSSSGKY  757
```

Copidosoma floridanum Contig51735, whole genome shotgun sequence

Sequence ID: gb|JBOX01051730.1|Length: 2519

Number of Matches: 1

Range 1: 182 to 430GenBankGraphics

Query: Polistes_canadensis gi|954548184|ref|XP_014616661.1|

Alignment statistics for match #1

Score	Expect	Method	Identities	Positives	Gaps	Frame
82.0 bits(201)	1e-15	Compositional matrix adjust.	47/83(57%)	65/83(78%)	1/83(1%)	-2

```
Query  13    NEEKDTRDENLAKWEIVLLANILIFTIFGNSLVLLSLYLRKYRAHKKL-SRMYFFIMNLS  71
             ++ +D RDE+LA WE+ LL  IL  ++  N LVL ++YLR+YR  +   +RM+FF+M+LS
Sbjct  430   SKGEDWRDEDLAFWEVALLGFILAVSVVSNGLVLFAIYLRRYRGRRHRLTRMHFFVMHLS  251

Query  72    IADLLTGLLDVLPQLAWDITFRF  94
             IADL TGLL+VLPQLAWDIT+R+
Sbjct  250   IADLTTGLLNVLPQLAWDITYRW  182
```

Fopius arisanus strain USDA-PBARC FA_bdor contig003174, whole genome shotgun sequence
Sequence ID: gb|JRKH01003174.1|Length: 107392
Number of Matches: 4
Range 1: 6563 to 7036GenBankGraphics
Query: Polistes_canadensis gi|954548184|ref|XP_014616661.1|

Alignment statistics for match #1

Score	Expect	Method	Identities	Positives	Gaps	Frame
117 bits(294)	1e-58	Compositional matrix adjust.	73/159(46%)	97/159(61%)	25/159(15%)	+2

```
Query  195    YSLMVFLLPFIVLVYTYVGICVGVWQSNKMSGAVDDRAyirsisnysrnrssfisKAMIN  254
              YS+ V +LPF VLVYTYV IC  +W++ +++     R  ++ + +   NR   IS+A I
Sbjct  6563   YSITVLMLPFSVLVYTYVSICREIWKNTEIT-VFGHRGEVKRMDSRENNREPVISRARIK  6739

Query  255    TVKQTIAVITLYAITSIPFIGCELWVSWDTKAFESSFANGP-------------------  295
              TVKQ I V++L+ ITS PF+GC+LW +WD  A ++ F  GP
Sbjct  6740   TVKQMITVVSLHVITSAPFVGCQLWAAWDPDALKTPFYQGPYI*KQQPNH*YYCI*YSYC  6919

Query  296    -----AFTILALLNSLTSCVNPWIYLSFNRELRQTLMNY  329
                   AFTIL+LL+SLTSCVNPWIY+ FNRELR TL NY
Sbjct  6920   SPVGPAFTILSLLSSLTSCVNPWIYMGFNRELRSTLKNY  7036
```

Range 2: 6057 to 6275GenBankGraphics

Alignment statistics for match #2

Score	Expect	Method	Identities	Positives	Gaps	Frame
105 bits(261)	1e-58	Compositional matrix adjust.	45/73(62%)	56/73(76%)	0/73(0%)	+3

```
Query  93     RFQGGLVLCKLVKFFQPFGLYLSSYILTATAIDRYHAICHPLSYCSVTSRQSRIMVYCAW  152
              RFQGG +LCKLVKF QP G Y S+Y+L A AIDRYHAIC+P +YC  T RQ+R+MVY AW
Sbjct  6057   RFQGGAMLCKLVKFGQPLGHYASTYVLIAGAIDRYHAICYPFNYCRTTHRQARLMVYIAW  6236

Query  153    SFALILCIPQLFV  165
                +  LC+PQ+ +
Sbjct  6237   GISSALCLPQVMI  6275
```

Range 3: 6385 to 6468GenBankGraphics

Alignment statistics for match #3

Score	Expect	Method	Identities	Positives	Gaps	Frame
43.1 bits(100)	1e-58	Compositional matrix adjust.	17/28(61%)	20/28(71%)	0/28(0%)	+1

```
Query  167    SYQEVMPRIWDCWATFDIQFGEKAYVTW  194
              SYQE+ P IWDCWA F    G++ YVTW
Sbjct  6385   SYQEISPGIWDCWADFIPPHGQRTYVTW  6468
```

Range 4: 5664 to 5894GenBankGraphics

Alignment statistics for match #4

Score	Expect	Method	Identities	Positives	Gaps	Frame
99.0 bits(245)	1e-21	Compositional matrix adjust.	49/77(64%)	63/77(81%)	1/77(1%)	+3

```
Query  16     KDTRDENLAKWEIVLLANILIFTIFGNSLVLLSLYLRKY-RAHKKLSRMYFFIMNLSIAD  74
              +D RDE LA WE+V+L  IL+ T+ GN LVL +LYLR+Y    +KL+RMYFF+M+LSIAD
Sbjct  5664   EDPRDEYLAIWEMVVLLVILVITVIGNFLVLFALYLRRYCGRRRKLTRMYFFMMHLSIAD  5843

Query  75     LLTGLLDVLPQLAWDIT  91
              L+TG+L+VLPQL WDIT
Sbjct  5844   LMTGVLNVLPQLMWDIT  5894
```

Hypochthonius rufulus scaffold216102_cov125, whole genome shotgun sequence
Sequence ID: gb|LBFL01050327.1|Length: 5802
Number of Matches: 1
Range 1: 339 to 1244GenBankGraphics
Query: Daphnia_pulex tr|K9JBV2|K9JBV2_DAPPU

Alignment statistics for match #1

Score	Expect	Method	Identities	Positives	Gaps	Frame
240 bits(612)	2e-68	Compositional matrix adjust.	143/360(40%)	196/360(54%)	62/360(17%)	-2

```
Query  17     RDEKLASVEISTLSVILVLAVTSNLTMLIAIWRQRRNRPLSRMYFFMMHLSLADLLVALF  76
              RDE LA  EI    +IL+LA+  N  +L  + +QR+ RP SR+Y FM HLSLADLLVA+F
Sbjct  1244   RDETLALFEIIIQILILILALIGNSFVLYVLLKQRQLRPWSRIYLFMAHLSLADLLVAIF  1065

Query  77     NILPQLAWDITYRFQGGDVLCRFVKYAQVMTLYLSTYILMFMAVDRYRAV-CCRNLHWNS  135
              NILPQL WDIT+RF+ GD +CR VKY QV  LYLSTYI++ M++DRY  +   +  HW +
Sbjct  1064   NILPQLIWDITFRFKAGDFMCRSVKYFQVFVLYLSTYIIVAMSIDRYLTIQSIKAGHWRA  885

Query  136    LKVAKCFVAASWVMAILFSIPQAVIFHEEEISV--GVTDCWVQFVEPWGAKAYVTWFVVS  193
               K  K  +  +W+++ LFSIPQA+IF  +E+ +  GV DCW  F   W  K YV+WFV S
Sbjct  884    SKTFKIMIIMAWILSTLFSIPQAIIFSVKEVKLNSGVFDCWATFTY-WNLKFYVSWFVTS  708

Query  194    IFGAPLLVIGVCYGVICRQIWIYsqsalpssqqpptssaypaLTSETGSTLSVMRRWILR  253
              +F  PL++I  CYG IC ++W Y+                    S T  +L V
Sbjct  707    VFIIPLIIIAFCYGTICFKLWKYN-------------------PSRTALSLHV-------  606

Query  254    ASLRWQKSRSSSNNGTKNHSAIANTSQLSDTI-PMRSLATQPASNPLSAEAKVIRslppp  312
                      R ++N+       + N  +    I   R +    +   L+ E +
Sbjct  605    --------RYAANDEDDTDREMMNHDKSDARISAKRKVQFIDSEKDLAIEER--------  474

Query  313    lspqccqqmplRRSNSNQNRITKAKMKTIKLTLAVVLCFVACWAPFCITQLIMVYCPPTS  372
                             +  N I++AK+KTIKLT+ VV+CF  CWAPFCITQL + + PP S
Sbjct  473    ---------------NRYNLISRAKVKTIKLTITVVICFFCCWAPFCITQLCLAFNPPNS  339
```

Steganacarus magnus, whole genome shotgun sequence
Sequence ID: gb|LBFN01047006.1|Length: 3438
Number of Matches: 1
Range 1: 853 to 1449GenBankGraphics

Query: Daphnia_pulex tr|K9JBV2|K9JBV2_DAPPU

Alignment statistics for match #1

Score	Expect	Method	Identities	Positives	Gaps	Frame
187 bits(476)	2e-50	Compositional matrix adjust.	94/202(47%)	136/202(67%)	7/202(3%)	+1

```
Query  17    RDEKLASVEISTLSVILVLAVTSNLTMLIAIWRQRRNRPLSRMYFFMMHLSLADLLVALF  76
             RDE LA +EI  L +I +L +  N  +LIA+  +R    ++RMY+F++HL ++DL+
Sbjct  853   RDEDLAKMEIIVLILIFILIIIGNSCVLIALAAKRFK--MTRMYYFLLHLCISDLITGCC  1026

Query  77    NILPQLAWDITYRFQGGDVLCRFVKYAQVMTLYLSTYILMFMAVDRYRAVC--CRNLHWN  134
              +LPQ+AWDITYRF+GG++LC+FVK  Q++  YLS+Y+L+  A+DRY+A+C    N  W
Sbjct  1027  TVLPQIAWDITYRFKGGNILCKFVKTIQLLGPYLSSYVLVVNAIDRYQAICFPLTNSQWT  1206

Query  135   SLKVAKCFVAASWVMAILFSIPQAVIFHEEEI--SVGVTDCWVQFVEPWGAKAYVTWFVV  192
             S + +K  +  +W +AI+FS PQA  F  +EI  S G +DCW  F EPWG K YVTW+ +
Sbjct  1207  S-RQSKLMITIAWCIAIVFSSPQAFFFSYQEIPNSSGESDCWGTFPEPWGEKLYVTWYAI  1383

Query  193   SIFGAPLLVIGVCYGVICRQIW  214
             SIF  PL +I   Y  ICR++W
Sbjct  1384  SIFFIPLFIITYTYVYICREVW  1449
```

Dermatophagoides farinae contig5815, whole genome shotgun sequence
Sequence ID: gb|ASGP01005815.1|Length: 7082
Number of Matches: 1
Range 1: 3977 to 4369GenBankGraphics
Query: Daphnia_pulex tr|K9JBV2|K9JBV2_DAPPU

Alignment statistics for match #1

Score	Expect	Method	Identities	Positives	Gaps	Frame
122 bits(307)	2e-28	Compositional matrix adjust.	60/133(45%)	90/133(67%)	9/133(6%)	+2

```
Query  52    RNRPL--SRMYFFMMHLSLADLLVALFNILPQLAWDITYRFQGGDVLCRFVKYAQVMTLY  109
             +NR L  +RMYFF+ HLS+ADL+   FN+LPQL W+I  RF GG+VLC+ +K+ Q++  Y
Sbjct  3977  KNRKLRKNRMYFFLAHLSIADLVTGFFNVLPQLGWEIASRFYGGNVLCKMIKFLQILGPY  4156

Query  110   LSTYILMFMAVDRYRAVCCRNLHWNSL---KVAKCFVAASWVMAILFSIPQAVIFHEEEI  166
             LS+Y+L+  ++DRY+A+C  +   NSL   + ++  VA +W+M++LF  PQ  IF  ++I
Sbjct  4157  LSSYVLVMTSIDRYQAIC--HPLSNSLAHTRRSRWMVAVAWIMSLLFCTPQTFIFSYQKI  4330

Query  167   SVGVTD--CWVQF  177
             S    D  CW  F
Sbjct  4331  SPTSDDYECWATF  4369
```

Sarcoptes scabiei Contig17956, whole genome shotgun sequence
Sequence ID: gb|JXLN01017957.1|Length: 20533
Number of Matches: 3
Range 1: 20086 to 20493GenBankGraphics
Query: Daphnia_pulex tr|K9JBV2|K9JBV2_DAPPU

Alignment statistics for match #1

Score	Expect	Method	Identities	Positives	Gaps	Frame
111 bits(277)	1e-24	Compositional matrix adjust.	53/136(39%)	84/136(61%)	7/136(5%)	-2

```
Query  53     NRPL--SRMYFFMMHLSLADLLVALFNILPQLAWDITYRFQGGDVLCRFVKYAQVMTLYL  110
              NR L  +RMYFF+ HLS+ADL+   FN+LPQLAW+    RF GG+ LC+ +K+ Q++  YL
Sbjct  20493  NRKLRKNRMYFFLAHLSIADLVTGFFNVLPQLAWEAAKRFYGGNFLCKTIKFLQILGPYL  20314

Query  111    STYILMFMAVDRYRAVC---CRNLHWNSLKVAKCFVAASWVMAILFSIPQAVIFHEEEIS  167
              S+Y+L+  A+DRY+A+C     +    + + ++  +A +W +++ +  PQ  IF  + +S
Sbjct  20313  SSYVLVMTAIDRYQAICYPLSNSFATKNTRRSRWMIAIAWFISLSYCTPQCFIFSYQRVS  20134

Query  168    VGVTD--CWVQFVEPW  181
                  D  CW  F   W
Sbjct  20133  YETDDYECWGTFQVSW  20086
```

Range 2: 14702 to 14812GenBankGraphics

Alignment statistics for match #2

Score	Expect	Method	Identities	Positives	Gaps	Frame
48.1 bits(113)	2e-04	Compositional matrix adjust.	17/37(46%)	26/37(70%)	0/37(0%)	-1

```
Query  179    EPWGAKAYVTWFVVSIFGAPLLVIGVCYGVICRQIWI  215
              +PW +K YVTW+ +S+F  P L++   +  ICR+IWI
Sbjct  14812  QPWMSKVYVTWYAISVFIVPFLILLWTHYFICREIWI  14702
```

Range 3: 5948 to 6088GenBankGraphics

Alignment statistics for match #3

Score	Expect	Method	Identities	Positives	Gaps	Frame
41.6 bits(96)	0.020	Compositional matrix adjust.	21/49(43%)	31/49(63%)	2/49(4%)	-1

```
Query  372   SQADVSPVAVIILLLASLNSCSNPWIYLAFSGSLLNQMRVCSILLLRST  420
             S   + PV  I++LL SLNSC NPWIY+ F+ +L+    +C    L+S+
Sbjct  6088  SSISLGPVIAILMLLPSLNSCVNPWIYIYFNPNLI--ALLCQFFKLKSS  5948
```

Centruroides exilicauda breed non domesticated species of scorpion Contig23310, whole genome shotgun sequence
Sequence ID: gb|AXZI01023310.1|Length: 1410
Number of Matches: 2

Range 1: 522 to 1058GenBankGraphics
Query: Caligus_rogercresseyi

Alignment statistics for match #1

Score	Expect	Method	Identities	Positives	Gaps	Frame
179 bits(453)	2e-50	Compositional matrix adjust.	88/180(49%)	125/180(69%)	6/180(3%)	-2

```
Query  24    NSIVILAIATRHIKMTRMYFFILHLSIADVLTAFLTLLPELIWTYTSPNFYGGGALCKIV  83
             N +V+LA+  R I+MTRMY+F+ HL ++D++TAFL +LP++ W  T   F GG  LCK+V
Sbjct  1058  NCLVLLALMGRRIQMTRMYYFLFHLCLSDLVTAFLHVLPQIAWDITY-RFRGGYLLCKLV  882

Query  84    KFCQMIGPYLSSYVLIMTAIDRYHAICNPLSKCTWTPKRSNIMIGLAWLVSFALCIPQAI  143
             K+ Q++GPYLSSY+L++TAIDRY AIC PL+  +WTP R  IM+ +AW++S   CIPQ
Sbjct  881   KYVQILGPYLSSYILVITAIDRYQAICFPLTSFSWTPIRGKIMVLVAWIISLLCCIPQLF  702

Query  144   IFGSSEAK-----YSCSASFVVDWGQKAYVTWFAVSNFFFPLLILIYCYGRICKTIWDNF  198
             IF   + +       C  +F+  WG+KAYVTW+ +S FF P +I+ + Y RIC  +W NF
Sbjct  701   IFSYQDVQGLSGVKDCWGTFIQPWGEKAYVTWYTISVFFIPFIIITFTYTRICLAVWKNF  522
```

Range 2: 294 to 428GenBankGraphics

Alignment statistics for match #2

Score	Expect	Method	Identities	Positives	Gaps	Frame
40.4 bits(93)	0.056	Compositional matrix adjust.	26/45(58%)	38/45(84%)	0/45(0%)	-2

```
Query  303  KRKSLKPRSHSIQGISRAkiktikltvvvivGYIACSAPFICVQL  347
            +RK+   R H+++G+S+AK+KT+K+T+VVI+ YI CS PFICVQL
Sbjct  428  RRKTPLSRRHNMKGLSKAKVKTVKITIVVIICYIVCSCPFICVQL  294
```

Mesobuthus martensii Contig339050, whole genome shotgun sequence
Sequence ID: gb|AYEL01079865.1|Length: 43520
Number of Matches: 2
Range 1: 24252 to 24788GenBankGraphics
Query: Caligus_rogercresseyi

Alignment statistics for match #1

Score	Expect	Method	Identities	Positives	Gaps	Frame
178 bits(451)	6e-48	Compositional matrix adjust.	89/180(49%)	123/180(68%)	6/180(3%)	+3

```
Query  24     NSIVILAIATRHIKMTRMYFFILHLSIADVLTAFLTLLPELIWTYTSPNFYGGGALCKIV  83
              N +V+LA+  R I+MTRMY+F+ HL I+D++TAFL +LP++ W  T   F GG  LCK V
Sbjct  24252  NCLVLLALMGRRIQMTRMYYFLFHLCISDLVTAFLHVLPQIAWDVTY-RFRGGYFLCKFV  24428

Query  84     KFCQMIGPYLSSYVLIMTAIDRYHAICNPLSKCTWTPKRSNIMIGLAWLVSFALCIPQAI  143
              K+ Q++GPYLSSY+L++TAIDRY AIC PL+  +WTP +  IM+ +AW++S   CIPQ
Sbjct  24429  KYVQLLGPYLSSYILVITAIDRYQAICFPLASFSWTPIKGKIMVMVAWIISLLCCIPQLF  24608

Query  144    IFGSSEAK-----YSCSASFVVDWGQKAYVTWFAVSNFFFPLLILIYCYGRICKTIWDNF  198
              IF   E         C  +F+  WG+KAYVTW+ +S FF P +I+ + Y RIC  +W NF
Sbjct  24609  IFSYQEIDDFSGVKDCWGTFIQPWGEKAYVTWYTISVFFIPFIIITFTYTRICIVVWKNF  24788
```

Range 2: 24882 to 25016GenBankGraphics

Alignment statistics for match #2

Score	Expect	Method	Identities	Positives	Gaps	Frame
39.3 bits(90)	0.17	Compositional matrix adjust.	24/45(53%)	38/45(84%)	0/45(0%)	+3

```
Query  303    KRKSLKPRSHSIQGISRAkiktikltvvvivGYIACSAPFICVQL  347
              +RK+   + H+++G+S+AK+KT+K+T+VV++ YI CS PFICVQL
Sbjct  24882  RRKTFLSQRHNLKGLSKAKVKTVKVTIVVVLCYIVCSCPFICVQL  25016
```

Oryctes borbonicus contig25461, whole genome shotgun sequence
Sequence ID: gb|LJIG01025627.1|Length: 62898
Number of Matches: 1
Range 1: 48816 to 49289GenBankGraphics
Query: Tribolium_castaneum tr|A3RE84|A3RE84_TRICA

Alignment statistics for match #1

Score	Expect	Method	Identities	Positives	Gaps	Frame
280 bits(716)	5e-82	Compositional matrix adjust.	130/158(82%)	148/158(93%)	0/158(0%)	+3

```
Query  24     DRNNTDRDENLARVEVATLAIIFLVTVIGNSTVLLALWTRRRYAGRKKLSRMYFFILHLS  83
              D N  DRDENLA++E+ATLA+IFLVT+IGN  VLLALWTR+RYAGRKKLSRMYFFILHLS
Sbjct  48816  DENVLDRDENLAKIEIATLAVIFLVTIIGNCVVLLALWTRKRYAGRKKLSRMYFFILHLS  48995

Query  84     IADLITAFLSVLPQLAWDITYRFYGGFLLCKVVKYGQTLGPYLSSYVLMATAIDRHQAIC  143
              IADLITAFLSVLPQLAW++T+RF GGF+LCKVVKYGQTLGPYLSSYVL+ATAIDRHQAIC
Sbjct  48996  IADLITAFLSVLPQLAWEVTFRFQGGFVLCKVVKYGQTLGPYLSSYVLVATAIDRHQAIC  49175

Query  144    YPLTYCSWTSRRSKVMVYLAWVASLAFCIPQLTIFTYT  181
              YPLTYCSWTSRRSKVMV++AW  SL+FCIPQ++ F ++
Sbjct  49176  YPLTYCSWTSRRSKVMVWIAWGVSLSFCIPQVSNFCFS  49289
```

Onthophagus taurus breed undomesticated insect ContigNC25934, whole genome shotgun sequence
Sequence ID: gb|JHOM01025934.1|Length: 3568
Number of Matches: 1
Range 1: 1238 to 1732GenBankGraphics
Query: Tribolium_castaneum tr|A3RE84|A3RE84_TRICA

Alignment statistics for match #1

Score	Expect	Method	Identities	Positives	Gaps	Frame
271 bits(694)	9e-80	Compositional matrix adjust.	128/165(78%)	150/165(90%)	2/165(1%)	-1

```
Query  14    NSTYLFDKHEDR--NNTDRDENLARVEVATLAIIFLVTVIGNSTVLLALWTRRRYAGRKK  71
             N+T L D  E+   +  +RDENLA++E+ATLAIIFLVT+IGN TVLLALWTRRRYAGRKK
Sbjct  1732  NATPLNDTTEENVIDIVERDENLAKIEIATLAIIFLVTIIGNGTVLLALWTRRRYAGRKK  1553

Query  72    LSRMYFFILHLSIADLITAFLSVLPQLAWDITYRFYGGFLLCKVVKYGQTLGPYLSSYVL  131
             LSRMYFFILHLS+ADLITAFLSVLPQL W++T+RF GGF+LCK+VK+GQTLGPYLSSY+L
Sbjct  1552  LSRMYFFILHLSVADLITAFLSVLPQLIWEVTFRFRGGFILCKLVKFGQTLGPYLSSYIL  1373
```

```
Query  132   MATAIDRHQAICYPLTYCSWTSRRSKVMVYLAWVASLAFCIPQLT  176
             +ATAIDRHQAICYPLTYCSWTSRRSK MV++AW  SL+FCIPQ++
Sbjct  1372  VATAIDRHQAICYPLTYCSWTSRRSKAMVWIAWGVSLSFCIPQVS  1238
```

Agrilus planipennis Contig56141, whole genome shotgun sequence
Sequence ID: gb|JENH01056123.1|Length: 4939
Number of Matches: 1
Range 1: 4171 to 4683GenBankGraphics
Query: Tribolium_castaneum tr|A3RE84|A3RE84_TRICA

Alignment statistics for match #1

Score	Expect	Method	Identities	Positives	Gaps	Frame
261 bits(668)	9e-76	Compositional matrix adjust.	124/171(73%)	148/171(86%)	2/171(1%)	+1

```
Query  8     QMDISENSTYLFDKHEDRNN--TDRDENLARVEVATLAIIFLVTVIGNSTVLLALWTRRR  65
             +M+ S N+  L+    D  N  ++RDENLA++E+A L +IF+VTV+GNS VLLALWTRR
Sbjct  4171  RMNFSVNNQSLYQPQFDVENVLSERDENLAKIEIAILIVIFIVTVLGNSIVLLALWTRRI  4350

Query  66    YAGRKKLSRMYFFILHLSIADLITAFLSVLPQLAWDITYRFYGGFLLCKVVKYGQTLGPY  125
             YAGRKKLSRMYFFILHLSIADL+TA LS LPQLAWDIT+RF GG+LLCK+VK+GQ LGPY
Sbjct  4351  YAGRKKLSRMYFFILHLSIADLVTALLSDLPQLAWDITFRFKGGWLLCKIVKFGQLLGPY  4530

Query  126   LSSYVLMATAIDRHQAICYPLTYCSWTSRRSKVMVYLAWVASLAFCIPQLT  176
             LSSY+LMATA+DR+QAICYPLTYCSWTSRRSK+MV+ AW+ SL FCIPQ+T
Sbjct  4531  LSSYILMATAVDRYQAICYPLTYCSWTSRRSKLMVWTAWITSLIFCIPQVT  4683
```

Anoplophora glabripennis Contig68241, whole genome shotgun sequence
Sequence ID: gb|AQHT01068175.1|Length: 5957
Number of Matches: 1
Range 1: 583 to 921GenBankGraphics
Query: Tribolium_castaneum tr|A3RE84|A3RE84_TRICA

Alignment statistics for match #1

Score	Expect	Method	Identities	Positives	Gaps	Frame
214 bits(546)	2e-59	Compositional matrix adjust.	99/113(88%)	109/113(96%)	0/113(0%)	+1

```
Query  65   RYAGRKKLSRMYFFILHLSIADLITAFLSVLPQLAWDITYRFYGGFLLCKVVKYGQTLGP  124
            RYAGRKKLSRMYFFILHLSIADLITAFL+ LPQLAWDIT+RF GGF +CKVVKYGQTLGP
Sbjct  583  RYAGRKKLSRMYFFILHLSIADLITAFLTDLPQLAWDITFRFQGGFFMCKVVKYGQTLGP  762

Query  125  YLSSYVLMATAIDRHQAICYPLTYCSWTSRRSKVMVYLAWVASLAFCIPQLTI  177
            YLSSY+LMATA+DRHQAICYPLTYCSWTSRRSKVMVYLAW+A+L FCIPQ+++
Sbjct  763  YLSSYILMATAVDRHQAICYPLTYCSWTSRRSKVMVYLAWMAALLFCIPQVSV  921
```

Leptinotarsa decemlineata Contig191282, whole genome shotgun sequence
Sequence ID: gb|AYNB01191279.1|Length: 2693
Number of Matches: 1
Range 1: 387 to 725GenBankGraphics
Query: Tribolium_castaneum tr|A3RE84|A3RE84_TRICA

Alignment statistics for match #1

Score	Expect	Method	Identities	Positives	Gaps	Frame
204 bits(520)	1e-56	Compositional matrix adjust.	97/113(86%)	103/113(91%)	0/113(0%)	-1

```
Query  65   RYAGRKKLSRMYFFILHLSIADLITAFLSVLPQLAWDITYRFYGGFLLCKVVKYGQTLGP  124
            RY GRKKLSRMYFFILHLSIADLITAFLSVL QL WDITYRF GG  LCK VKYGQTLGP
Sbjct  725  RYTGRKKLSRMYFFILHLSIADLITAFLSVLTQLGWDITYRFKGGNFLCKSVKYGQTLGP  546

Query  125  YLSSYVLMATAIDRHQAICYPLTYCSWTSRRSKVMVYLAWVASLAFCIPQLTI  177
            YLSSY+L+ATA+DRHQAICYPLTYCSWTSRRSKVMVY+AW  SL FCIPQ++I
Sbjct  545  YLSSYILVATALDRHQAICYPLTYCSWTSRRSKVMVYIAWTVSLIFCIPQVSI  387
```

Hypothenemus hampei scaffold1221, whole genome shotgun sequence
Sequence ID: gb|LBGY01001191.1|Length: 69032
Number of Matches: 5
Range 1: 61624 to 61809GenBankGraphics
Query: Tribolium_castaneum tr|A3RE84|A3RE84_TRICA

Alignment statistics for match #1

Score	Expect	Method	Identities	Positives	Gaps	Frame
114 bits(286)	3e-25	Compositional matrix adjust.	52/62(84%)	57/62(91%)	0/62(0%)	-3

```
Query  117    KYGQTLGPYLSSYVLMATAIDRHQAICYPLTYCSWTSRRSKVMVYLAWVASLAFCIPQLT  176
              +YGQTLGPYLSSYVLMATAIDRHQAICYPLTYCSWTSRRSKVM+Y+AW+  L  CIPQ+
Sbjct  61809  QYGQTLGPYLSSYVLMATAIDRHQAICYPLTYCSWTSRRSKVMMYVAWIVGLLCCIPQVN  61630

Query  177    IF  178
              IF
Sbjct  61629  IF  61624
```

Range 2: 59610 to 59918GenBankGraphics

Alignment statistics for match #2

Score	Expect	Method	Identities	Positives	Gaps	Frame
112 bits(279)	2e-24	Compositional matrix adjust.	65/103(63%)	83/103(80%)	2/103(1%)	-1

```
Query  207    YSISVFMVPLVVLIFTYTSICIEIWqssesslrprssqksAPGKR--TPLISRAKINTVK  264
              YS+++F++PL+VL++TY+ IC EIW+ S+SSLRPR+  K    KR   PLISRAKINTVK
Sbjct  59918  YSLTIFIIPLLVLVYTYSCICREIWRCSDSSLRPRNMSKQIATKRDRVPLISRAKINTVK  59739

Query  265    QTIAVIVMYIACSTPFILAQLWATWDPQSPFIDGPVFVILTLL  307
              QT+AVIVMYI CSTPFI++QLWAT DP S F++G  F + TLL
Sbjct  59738  QTVAVIVMYIVCSTPFIVSQLWATIDPTSSFLEGKNFQVFTLL  59610
```

Range 3: 62170 to 62328GenBankGraphics

Alignment statistics for match #3

Score	Expect	Method	Identities	Positives	Gaps	Frame
96.7 bits(239)	2e-19	Compositional matrix adjust.	44/53(83%)	48/53(90%)	0/53(0%)	-3

```
Query  65     RYAGRKKLSRMYFFILHLSIADLITAFLSVLPQLAWDITYRFYGGFLLCKVVK  117
              RY GRKKLSRMYFFILHLSIADLI AF SVLPQLAW++T+RF GGF+LCK VK
Sbjct  62328  RYCGRKKLSRMYFFILHLSIADLIVAFFSVLPQLAWEVTFRFQGGFILCKFVK  62170
```

Range 4: 54000 to 54143GenBankGraphics

Alignment statistics for match #4

Score	Expect	Method	Identities	Positives	Gaps	Frame
77.4 bits(189)	5e-13	Compositional matrix adjust.	35/48(73%)	41/48(85%)	0/48(0%)	-1

```
Query  295    FIDGPVFVILTLLYSLNSCVNPWIYLAFNRELPRLLLRHYTASSKNYR  342
               + G  F ILTLLYSLNSCVNPWIYL FNRELP+LL+RH+ AS+K+YR
Sbjct  54143  LLAGTPFTILTLLYSLNSCVNPWIYLIFNRELPQLLIRHFVASNKSYR  54000
```

Range 5: 61006 to 61104GenBankGraphics

Alignment statistics for match #5

Score	Expect	Method	Identities	Positives	Gaps	Frame
45.1 bits(105)	0.007	Compositional matrix adjust.	17/33(52%)	23/33(69%)	0/33(0%)	-3

```
Query  175    LTIFTYTSVGEDEYDCWATFQEPWGKRAYVTWY  207
              + IF+Y  V    YDCWATF + WG++AYV W+
Sbjct  61104  VIIFSYQEVAHGTYDCWATFNKEWGEQAYVIWF  61006
```

Supplementary Table S1. List of analysed arthropod species

Subphylum or Order[a]	#	#	Species	Oxytocin-like peptide neurophysin domain (+)	Oxytocin-like precursor ID	Oxytocin-like receptor (+)[b]	Oxytocin-like receptor ID	Systematics (from NCBI)	Commom name
**Chelicerata**	1	1	*Limulus polyphemus*	CFITNCPPG	926609726	+++	926615389; 926636334; 926627326	Merostomata; Xiphosura; Limulidae	horseshoe crabs
	2	2	*Ixodes scapularis*	CFITNCPPG	AGM20426.1	+++	215501697; 215501196; 215493320	Arachnida;Acari; Parasitiforme ; Ixodida; Ixodoidea; Ixodidae; Ixodinae	mites, ticks
	3	3	*Ixodes ricinus*	CFITNCPPG	807918304	+	1016843980	Arachnida;Acari; Parasitiformes; Ixodida; Ixodoidea; Ixodidae; Ixodinae	
	4	4	*Rhipicephalus microplus*	CFITNCPPG	ADMZ02000298.1			Arachnida;Acari; Parasitiformes; Ixodida; Ixodoidea; Ixodidae; Rhipicephalinae	
	5	5	*Ornithodoros turicata*			+	GDIE01074084.1	Arachnida; Acari; Parasitiformes; Ixodida; Ixodoidea; Argasidae	
	6	6	*Metaseiulus occidentalis*	CFITNCPIG	391348425	++	391328891; 391341506	Arachnida;Acari; Parasitiformes; Mesostigmata; Gamasina; Phytoseioidea; Phytoseiidae; Typhlodrominae	
	7	7	*Varroa destructor*	CFITNCPIG	ADDG01001370.1	++	ADDG01054890.1; ADDG01034892.1	Arachnida; Acari; Parasitiformes; Mesostigmata; Gamasina; Dermanyssoidea; Varroidae	
	8	8	*Panonychus ulmi*			+	GCAC01000419.1	Arachnida;Acari;Acariformes; Trombidiformes; Prostigmata; Eleutherengona; Raphignathae;Tetranychoidea; Tetranychidae	
	9	9	*Tetranychus urticae*	CFITNCPPG	1005954275	+	1005961572	Arachnida; Acari; Acariformes; Trombidiformes; Prostigmata; Eleutherengona;Raphignathae; Tetranychoidea; Tetranychidae	
	10	10	*Dermatophagoides farinae*	CFITNCPRA	ASGP01007982.1	+	ASGP01005815.1	Arachnida;Acari; Acariformes; Sarcoptiformes; Astigmata; Psoroptidia; Analgoidea; Pyroglyphidae; Dermatophagoidinae	
	11	11	*Sarcoptes scabiei*	CFITNCPPA	KPL97556.1	+	gb|JXLN01017957.1	Arachnida;Acari; Acariformes; Sarcoptiformes; Astigmata; Psoroptidia; Sarcoptoidea; Sarcoptidae; Sarcoptinae	
	12	12	*Hypochthonius rufulus*	CFITNCPPG	LBFL01028909.1	+	LBFL01050327.1	Arachnida;Acari; Acariformes; Sarcoptiformes; Oribatida; Enarthronota; Hypochthonoidea; Hypochthoniidae	
	13	13	*Achipteria coleoptrata*	CFITNCPPG	LBFM01000221.1	++	LBFM01000129.1|: 5981-6625; LBFM01004763.1	Arachnida;Acari; Acariformes; Sarcoptiformes; Oribatida; Brachypylina; Achipterioidea; Achipteriidae	
	14	14	*Platynothrus peltifer*	CFITNCPPG	LBFO01089471.1	+++++	LBFO01084717.1; LBFO01069293.1; LBFO01087167.1; LBFO01104342.1, LBFO01089844.1	Arachnida;Acari; Acariformes; Sarcoptiformes; Oribatida; Desmonomata; Crotonioidea; Camisiidae	
	15	15	*Steganacarus magnus*	CFITNCPPG	LBFN01029022.1	+	LBFN01047006.1	Arachnida;Acari; Acariformes; Sarcoptiformes; Oribatida; Mixonomata; Phthiracaroidea; Steganacaridae	
	16	16	*Centruroides exilicauda*	CFITNCPPG	AXZI01013139.1	+	AXZI01023310.1	Arachnida;Scorpiones; Buthida; Buthoidea; Buthidae	scorpions
	17	17	*Mesobuthus martensii*	CFITNCPPG	AYEL01091269.1	+	AYEL01079865.1	Arachnida;Scorpiones; Buthida; Buthoidea; Buthidae	
	18	18	*Loxosceles reclusa*					Arachnida;Araneae; Araneomorphae; Haplogynae; Sicariidae	spiders
	19	19	*Latrodectus hesperus*					Arachnida;Araneae; Araneomorphae; Entelegynae; Araneoidea; Theridiidae	
	20	20	*Parasteatoda tepidariorum*					Arachnida;Araneae; Araneomorphae; Entelegynae; Araneoidea; Theridiidae	
	21	21	*Stegodyphus mimosarum*					Arachnida;Araneae; Araneomorphae; Entelegynae; Eresoidea; Eresidae	
**Myriapoda**	22	1	*Symphylella vulgaris*	CFITNCPIG	GAKX01066982.1			Symphyla; Scolopendrellidae	milipedes centipedes
	23	2	*Strigamia maritima*	CYITNCPPG	AFFK01014417.1:157792-158367	++	AFFK01021536.1|: 6079-6519;6079-	Chilopoda; Pleurostigmophora; Epimorpha; Geophilomorpha;	

							6519; AFFK01021536:22344-23360;25188-25421	Linotaeniidae	
	24	3	*Scolopendra subspinipes*	CFITNCPPG CYIINCIDND	GBIM01005348.1; GBIM01002605.1			Chilopoda; Pleurostigmophora; Epimorpha; Scolopendromorpha; Scolopendridae	
	25	4	*Lithobius forficatus*	CFITNCPPG	679426463			Chilopoda; Pleurostigmophora; Lithobiomorpha; Lithobiidae; Lithobius	
	26	5	*Glomeris pustulata*			+	GAKW01027288.1	Diplopoda; Pentazonia; Glomerida; Glomeridae	
**Crustaceans**	27	1	*Cypridina sp.*					Ostracoda;Myodocopa; Myodocopida; Cypridinoidea; Cypridinidae	mussels, shrimps crabs, lobsters, crayfish, shrimps, krill, woodlice
	28	2	*Sarsinebalia urgorrii*					Malacostraca; Phyllocarida; Leptostraca; Nebaliidae	
	29	3	*Hyalella azteca*			+	JQDR01035809.1	Malacostraca; Eumalacostraca; Peracarida; Amphipoda; Senticaudata; TalitridaTalitroidea; Hyalellidae	
	30	4	*Parhyale hawaiensis*			+	LQNS01011624.1	Malacostraca; Eumalacostraca; Peracarida; Amphipoda; Senticaudata; TalitridaTalitroidea; Hyalidae	
	31	5	*Litopenaeus vannamei*	CFITNCPPG	JP362883.1			Malacostraca; Eumalacostraca; Eucarida; Decapoda; Dendrobranchiata; PenaeoideaPenaeidae	
	32	6	*Celuca pugilator*					Malacostraca;Eumalacostraca;Eucarida;Decapoda; Pleocyemata;Brachyura; Eubrachyura;Thoracotremata; Ocypodoidea	
	33	7	*Scylla paramamosain*	+	ALQ28600.1			Malacostraca; Eumalacostraca; Eucarida; Decapoda; Pleocyemata; Brachyura;Eubrachyura; Heterotremata; Portunoidea; Portunidae	
	34	8	*Homarus americanus*	CFITNCPPG	GEBG01052869.1; GEBG01052869.1			Malacostraca; Eumalacostraca; Eucarida; Decapoda; Pleocyemata; Astacidea; Nephropoidea; Nephropidae	
	35	9	*Triops cancriformis*	CFITNCPPG	787010878:2475-2558	+	787019224:5635-6762	Branchiopoda; Phyllopoda; Notostraca; Triopsidae	shrimps
	36	10	*Daphnia magna*	CFITNCPPG	JAK93241.1	+	JAK30878.1, JAN78479.1, JAJ41077.1, JAM92944.1	Branchiopoda; Phyllopoda; Diplostraca; Cladocera; Anomopoda; Daphniidae	
	37	11	*Daphnia pulex*	CFITNCPPG	EFX71881.1	+	K9JBV2; ADK27313.1	Branchiopoda; Phyllopoda; Diplostraca; Cladocera; Anomopoda; Daphniidae	
	38	12	*Tigriopus californicus*	CFITNCPSG	GBSZ01008472.1	+	GBTC01003018.1	Maxillopoda; Copepoda; Neocopepoda; Podoplea; Harpacticoida; Harpacticidae	barnacles, copepods
	39	13	*Caligus rogercresseyi*	CFITNCPVGS	GAZX01009828.1|	+	LBBU01122538.1	Maxillopoda; Copepoda; Neocopepoda;Sodoplea; Siphonostomatoida; Caligidae	
	40	14	*Lepeophtheirus salmonis*	CFITNCPVGG	JAJ41077.1; JAN78479.1; JAK30878.1; JAM92944.1	+	ADND02009520.1	Maxillopoda;Copepoda; Neocopepoda; Podoplea; Siphonostomatoida; Caligidae	
	41	13	*Calanus finmarchicus*	CFISNCPVS	GAXK01089312.1; GAXK01048248.1; GBFB01189857.1	+	LBBU01122538.1	Maxillopoda; Copepoda; Neocopepoda; Gymnople; Calanoida; Calanidae	
	42	14	*Eurytemora affinis*					Maxillopoda; Copepoda; Neocopepoda; Gymnople; Calanoida; Temoridae	
	43	15	*Speleonectes cf. tulumensis*	CFILDCPLM	JL155260.1			Remipedia; Nectiopoda; Speleonectidae	
**Protura**	44	1	*Acerentomon sp.*			+	GAXE01139219.1	Acerentomata; Acerentomidae	
**Collembola**	45	1	*Sminthurus viridis*	CFITNCPPG	GATZ01101765.1			Symphypleona; Sminthuridae	
	46	2	*Tetrodontophora bielanensis*	+	GAXI01072001.1			Poduromorpha; Poduroidea; Onychiuridae; Tetrodontophorinae	
	47	3	*Anurida maritima*	CFITNCPPG	GAUE01051410.1	+	GAUE01010701.1	Poduromorpha; Poduroidea; Neanuridae; Pseudachorutinae	
	48	4	*Pogonognathellus sp.*	CFITNCPPG	GATD01083368.1	+	GATD01094158.1	Entomobryomorpha; Tomoceroidea; Tomoceridae	
	49	5	*Folsomia candida*	CFITNCPPG	GASX01087107.1	+	GASX01061907.1	Entomobryomorpha; Isotomoidea; Isotomidae; Proisotominae	
**Diplura**	50	1	*Campodea augens*	+	GAYN01126761.1			Rhabdura;Campodeoidea; Campodeidae	

	51	2	*Catajapyx aquilonaris*	CFITNCPPG	JYFJ01031256.1	+	JYFJ01023801.1	Dicellurata; Japygoidea; Japygidae; Japyginae
	52	3	*Occasjapyx japonicus*	CFITNCPPG	GAXJ01105745.1	+	GAXJ01102351.1	Dicellurata; Japygoidea; Japygidae; Japyginae
**Archaeognatha**	53	1	*Meinertellus cundinamarcensis*	CFITNCPPG	GAUG01237411.1	+	GAUG01249614.1	Meinertellidae
	54	2	*Machilis hrabei*	CFITNCPPG	GAUM01180328.1	+	GAUM01006385.1	Machilidae
**Zygentoma**	55	1	*Tricholepidion gertschi*	CFITNCPPG	GASO01245888.1	+	GASO01213666.1	Lepidotrichidae
	56	2	*Thermobia domestica*					Lepismatidae
	57	3	*Atelura formicaria*	CFITNCPPG	GAYJ01010327.1; GAYJ01211146.1			Nicoletiidae
**Odonata**	58	1	*Calopteryx splendens*					Zygoptera; Calopterygoidea; Calopterygidae;Calopteryx
	59	2	*Cordulegaster boltonii*			+	GAYO01131401.1	Anisoptera; Cavilabiata; Cordulegastridae
	60	3	*Ladona fulva*	CFITNCPPG	APVN01010136.1	+	APVN01071783.1; APVN01071781.1	Anisoptera; Libellulidae
	61	4	*Epiophlebia superstes*			+	GAVW01126771.1	Anisozygoptera; Epiophlebiidae
**Ephemeroptera**	62	1	*Baetis sp.*	+	GATU01026804.1	+	GATU01012660.1	Pisciforma
	63	2	*Isonychia bicolor*	+	GAXA01082263.1	+	GAXA01009774.1	Setisura; Isonychiidae
	64	3	*Eurylophella sp.*	CFITNCPPG	GAZG01089132.1	+	GAZG01097604.1	Furcatergalia; Pannota; Ephemerelloidea; Ephemerellidae
	65	4	*Ephemera danica*	CFITNCPPG	AYNC01016661.1	+	AYNC01080313.1	Scapphodonta; Ephemeridae
**Zoraptera**	66	1	*Zorotypus caudelli*			+	GAYA01146842.1	Zorotypidae
**Dermaptera**	67	1	*Forficula auricularia*					Forficulina; Forficuloidea; Forficulidae
	68	2	*Apachyus charteceus*	CLITNCPKG	GAUW01104117.1	+		Forficulina; Apachyoidea;Apachyidae
**Plecoptera**	69	1	*Leuctra sp.*			+	GAUF01061620.1	Nemouroidea; Leuctridae
	70	2	*Perla marginata*			+	GATV01107607.1	Perloidea; Perlidae
	71	3	*Cosmioperla kuna*					Eusthenioidea; Eustheniidae
**Orthoptera**	72	1	*Gryllotalpa sp.*	CMIINCPRG	GAWZ01146014.1			Ensifera; Grylloidea;Gryllotalpidae
	73	2	*Teleogryllus commodus*	CMIINCPRG	GBHB01042598.1			Ensifera; Grylloidea; Gryllidae
	74	3	*Ceuthophilus sp.*	+	GAUX01264151.1			Ensifera; Rhaphidophoroidea; Rhaphidophoridae
	75	4	*Tetrix subulata*					Caelifera; Tetrigoidea; Tetrigidae
	76	5	*Prosarthria teretrirostris*					Caelifera; Acridomorpha; Eumastacoidea; Proscopiidae
	77	6	*Locusta migratoria manilensis*	CLITNCPRG	GCGJ01016197.1			Caelifera; Acridomorpha; Acridoidea; Acrididae
	78	7	*Stenobothrus lineatus*			+	GAUZ01276196.1	Caelifera; Acridomorpha; Acridoidea; Acrididae
**Mantophasma-todea**	79	1	*Tanzaniophasma sp.*					Tanzaniophasmatidae
**Grylloblattodea**	80	1	*Galloisiana yuasai*	CLITNCPKG	GAWN01176617.1	+	GAWN01106889.1	Grylloblattodea; Grylloblattidae
	81	2	*Grylloblatta bifratrilecta*					Grylloblattodea; Grylloblattidae
**Embioptera**	82	1	*Haploembia palaui*	CLITNCPIG	GAZA01216527.1	+	GAZA01236978.1	Oligotomidae
	83	2	*Aposthonia japonica*	CLITNCPKG	GAWU01233766.1	+	GAWU01020812.1	Oligotomidae
**Phasmatodea**	84	1	*Timema cristinae*					Timematodea; Timematoidea; Timematidae
	85	2	*Peruphasma schultei*					Verophasmatodea; Areolatae; Pseudophasmatoidea; Pseudophasmatidae
	86	3	*Aretaon asperrimus*			+	GAWC01043798.1	Verophasmatodea; Areolatae; Bacilloidea;Heteropterygidae
	87	5	*Sipyloidea sipylus*			+	GAWF01069276.1	Verophasmatodea; Anareolatae; Diapheromeridae
	88	4	*Ramulus artemis*			+	GAWE01092086.1	Verophasmatodea; Anareolatae; Phasmatidae
	89	6	*Extatosoma tiaratum*			+	GAWG01068689.1	Verophasmatodea; Anareolatae; Phasmatidae
	90	7	*Medauroidea extradentata*	+	GAWD01076003.1	+	GAWD01027027.1	Verophasmatodea; Anareolatae; Phasmatidae
**Mantodea**	91	1	*Metallyticus splendidus*					Metallyticidae
	92	2	*Empusa pennata*					Empusidae

	93	3	*Mantis religiosa*			+	GASW01107792.1	Mantidae	
**Blattodea**	94	1	*Blaberus atropos*	+	GAYD01152108.1	+	GAYD01167921.1	Blaberoidea; Blaberidae	
	95	3	*Blattella germanica*	CLITNCPKG	JPZV01163469.1; JPZV01163471.1	+	JPZV01131362.1; JPZV01131361.1	Blaberoidea; Ectobiidae	
	96	2	*Periplaneta americana*	+	GAWS01144903.1			Blattoidea; Blattidae	
	97	4	*Cryptocercus wrighti*	CLITNCPKG	GAZN01121910.1			Blattoidea; Cryptocercidae	
**Isoptera**	98	1	*Mastotermes darwiniensis*					Mastotermitidae	
	99	2	*Prorhinotermes simplex*	+	GASE02010515.1			Rhinotermitidae	
	100	3	*Nasutitermes exitiosus*					Termitidae	
	101	4	*Zootermopsis nevadensis*	CLITNCPKG	GCA_000696155.1 :scaffold412:22921: 25055:1 gene:L798_08907	+	KDQ65295:16-501	Termopsidae	
**Thysanoptera**	102	1	*Gynaikothrips ficorum*	CLITNCPRG	GAXG01236909.1			Tubulifera; Phlaeothripoidea	
	103	2	*Frankliniella occidentalis*	+	JMDY01016652.1|: 503-766	+	JMDY01025147.1|	Terebrantia; Thripoidea; Thripidae	
	104	3	*Frankliniella cephalica*			+	GAYE01097758.1	Terebrantia; Thripoidea; Thripidae	
	105	4	*Thrips palmi*					Terebrantia; Thripoidea;Thripidae	
**Hemiptera**	106	1	*Acanthocasuarina muellerianae*			+	GAYY01128821.1	Sternorrhyncha; Psylliformes; Psylloidea; Psyllidae	jumping plant lice
	107	2	*Diaphorina citri*	CLINNCPTG	AWGM01069842.1	+	XM_008483692.1	Sternorrhyncha; Psylliformes; Psylloidea; Psyllidae	
	108	3	*Pachypsylla venusta*	CLISNCPKG	AZLD01215249.1	+	AZLD01271666.1; AZLD01292216.1; gb|AZLD01271660 .1	Sternorrhyncha; Psylliformes; Psylloidea; Psyllidae	
	109	4	*Trialeurodes vaporariorum*					Sternorrhyncha; Aleyrodiformes; Aleyrodoidea; Aleyrodidae; Aleyrodinae	white flies
	110	5	*Bemisia tabaci*					Sternorrhyncha; Aleyrodiformes; Aleyrodoidea; Aleyrodidae; Aleyrodinae	
	111	6	*Pseudococcus longispinus*					Sternorrhyncha; Aphidiformes; Coccoidea; Pseudococcidae	scale insects
	112	7	*Maconellicoccus hirsutus*					Sternorrhyncha; Aphidiformes; Coccoidea; Pseudococcidae	
	113	8	*Ferrisia virgata*					Sternorrhyncha; Aphidiformes; Coccoidea; Pseudococcidae	
	114	9	*Trionymus perrisii*					Sternorrhyncha; Aphidiformes; Coccoidea; Pseudococcidae	
	115	10	*Paracoccus marginatus*					Sternorrhyncha; Aphidiformes; Coccoidea; Pseudococcidae	
	116	11	*Planococcus citri*					Sternorrhyncha; Aphidiformes; Coccoidea; Pseudococcidae	
	117	12	*Dactylopius coccus*					Sternorrhyncha; Aphidiformes; Coccoidea; Dactylopiidae	
	118	13	*Essigella californica*					Sternorrhyncha; Aphidiformes; Aphidoidea; Lachnidae	aphids
	119	14	*Diuraphis noxia*					Sternorrhyncha; Aphidiformes; Aphidoidea; Aphididae; Macrosiphini	
	120	15	*Acyrthosiphon pisum*					Sternorrhyncha; Aphidiformes; Aphidoidea; Aphididae; Macrosiphini	
	121	16	*Aphis gossypii*					Sternorrhyncha; Aphidiformes; Aphidoidea; Aphididae; Aphidini	
	122	17	*Velia caprai*					Heteroptera; Gerromorpha; Gerroidea; Veliidae; Veliinae	bugs
	123	18	*Gerris buenoi*					Heteroptera; Gerromorpha; Gerroidea; Gerridae; Gerrinae	
	124	19	*Oncopeltus fasciatus*	+	JHQO01232535.1			Heteroptera; Panheteroptera; Pentatomomorpha; Lygaeoidea; Lygaeinae	
	125	20	*Piezodorus guildinii*					Heteroptera;Panheteroptera; Pentatomomorpha; Pentatomoidea; Pentatomidae; Pentatominae	
	126	21	*Halyomorpha halys*	CLITNCPRG	JMPT01086066.1|: 13297-13677	+	XP_014279829.1	Heteroptera; Panheteroptera; Pentatomomorpha; Pentatomoidea; Pentatomidae; Pentatominae	
	127	22	*Acanthosoma haemorrhoidale*					Heteroptera; Panheteroptera; Pentatomomorpha; Pentatomoidea; Acanthosomatidae	
	128	23	*Cimex lectularius*					Heteroptera;Panheteroptera; Cimicomorpha; Cimicidae	
	129	24	*Rhodnius prolixus*					Heteroptera; Panheteroptera; Cimicomorpha; Reduviidae;	

								Triatominae	
	130	25	*Notostira elongata*					Heteroptera; Panheteroptera; Cimicomorpha; Miridae; Stenodemini	
	131	26	*Ranatra linearis*					Heteroptera; Panheteroptera; Nepomorpha; Nepidae; Ranatrinae	
	132	27	*Xenophysella greensladeae*					Coleorrhyncha; Peloridiomorpha; Peloridiidae	moss bugs
	133	28	*Nilaparvata lugens*	CLITNCPRG	AFW19795.1	+	BAO01091.1	Auchenorrhyncha; Fulgoroidea; Delphacidae; Delphacinae	planthopper
	134	29	*Homalodisca vitripennis*	CLITNCPKG	JJNS01285955.1; JJNS01158934.1	+	JJNS01062030.1; JJNS01434099.1	Auchenorrhyncha; Membracoidea; Cicadellidae; Cicadellinae; unclassified Cicadellinae	leafhoppers
	135	30	*Okanagana villosa*					Auchenorrhyncha; Cicadoidea; Cicadidae; Tibicininae; Tibicinini	cicadas
	136	31	*Cercopis vulnerata*			+	GAUN01111305.1	Auchenorrhyncha; Cercopoidea; Cercopidae;	froghoppers
**Psocodea**	137	1	*Ectopsocus briggsi*					Psocomorpha; Homilopsocidea; Lachesilloidea; Ectopsocidae	
	138	2	*Liposcelis bostrychophila*	CLITNCPRG	GAYV01106698.1	+	GAYV01018252.1	Troctomorpha; Nanopsocetae; Liposcelidae	
	139	3	*Menopon gallinae*	CLITNCPRG	GAWR01010043.1			Phthiraptera; Amblycera; Menoponidae	
	140	4	*Pediculus humanus corporis*					Phthiraptera; Anoplura; Pediculidae	
**Hymenoptera**	141	1	*Tenthredo koehleri*	+	GAWW01055882.1	+	GAWW01089352.1	Tenthredinoidea; Tenthredinidae; Tenthrediniinae	sawflies
	142	2	*Athalia rosae*	CLIINCPRG	XP_012261240.1	+	XP_012255235.1	Tenthredinoidea; Tenthredinidae; Allantinae	
	143	3	*Neodiprion lecontei*	CLIINCPRG	XP_015516596.1	+	LGIB01000555.1	Tenthredinoidea; Diprionidae; Diprioninae	
	144	4	*Orussus abietinus*	CLITNCPRG	XP_012280828.1	+	XP_012272252.1	Endopterygota; Hymenoptera; Orussoidea	
	145	5	*Ganaspis sp*			+	GAIW01017396.1	Symphyta; Cynipoidea; Figitidae; Eucoilinae	wasps
	146	6	*Leptopilina boulardi*			+	GAJA01004803.1	Symphyta; Cynipoidea; Figitidae; Eucoilinae	
	147	7	*Leptopilina clavipes*			+	GAXY01060534.1	Symphyta; Cynipoidea; Figitidae; Eucoilinae	
	148	8	*Nasonia giraulti*	CLITNCPRG	GBEC01021014.1	+	GBEC01022231.1	Apocrita; Chalcidoidea; Pteromalidae; Pteromalinae	
	149	9	*Nasonia vitripennis*	CLITNCPRG	XP_001606547.1	+	NM_001172274.1	Apocrita; Chalcidoidea; Pteromalidae; Pteromalinae	
	150	10	*Nasonia longicornis*	CLITNCPRG	ADAP01018019.1	+	ADAP01169230.1; ADAP01169223.1	Apocrita; Chalcidoidea; Pteromalidae; Pteromalinae	
	151	11	*Trichogramma pretiosum*	CLITNCPRG	XP_014226062.1	+	JARR01003858.1	Apocrita; Chalcidoidea; Trichogrammatidae	
	152	12	*Copidosoma floridanum*	CLITNCPRG	XP_014216967.1	+	JBOX01051729.1; JBOX01051730.1	Apocrita; Chalcidoidea; Encyrtidae; Encyrtinae	
	153	13	*Ceratosolen solmsi marchali*	+	XP_011505828.1			Apocrita; Chalcidoidea; Agaonidae; Agaoninae	
	154	14	*Cephus cinctus*	CLITNCPRG	XP_015604200.1	+	AMWH01000205.1	Apocrita; Cephoidea; Cephidae	
	155	15	*Diachasma alloeum*	CLITNCPRG	XP_015116397.1	+	LDKA01015940.1; : LDKA01015941.1	Apocrita; Ichneumonoidea; Braconidae; Opiinae	
	156	16	*Fopius arisanus*	CLITNCPRG	XP_011303419.1	+	JRKH01003174.1	Apocrita; Ichneumonoidea; Braconidae; Opiinae	
	157	17	*Microplitis demolitor*	CLITNCPRG	XP_008557637.1	+	GAXO01007279.1	Apocrita; Ichneumonoidea; Braconidae; Microgastrinae	
	158	18	*Cotesia vestalis*	CLITNCPRG	GAUP01067026.1	+	GAUP01058790.1	Apocrita; Ichneumonoidea; Braconidae; Microgastrinae	
	159	19	*Telenomus podisi*	CLITNCPRG	GBEU01047114.1	+	GBEU01035833.1	Apocrita; Platygastroidea; Scelionidae; Telenominae	
	160	20	*Chrysis viridula*	CLITNCPRG	GATY01003634.1	+	GATY01011771.1	Apocrita; Aculeata; Chrysidoidea; Chrysididae; Chrysidinae	
	161	21	*Argochrysis armilla*			+	GAXO01007279.1	Apocrita; Aculeata; Chrysidoidea; Chrysididae; Chrysidinae	
	162	22	*Sphaeropthalma orestes*			+	GAXP01010798.1	Apocrita; Aculeata; Pompiloidea; Mutillidae; Sphaeropthalminae	
	163	23	*Polistes dominula*	CLITNCPRG	XP_015176922.1	+	LMBU01000007.1	Apocrita; Aculeata; Vespoidea; Vespidae; Polistinae	
	164	24	*Polistes canadensis*	CLITNCPRG	XP_014601007.1	+	XP_014616661.1	Apocrita; Aculeata; Vespoidea; Vespidae; Polistinae	
	165	25	*Polistes metricus*	CLITNCPRG	GBGV01014087.1|: 870-1319	+	GBGV01009051.1	Apocrita; Aculeata; Vespoidea; Vespidae; Polistinae	
	166	26	*Mischocyttarus flavitarsis*	CLITNCPRG	GAXM01001949.1| :391-804	+	GAXM01016032.1	Apocrita; Aculeata; Vespoidea; Vespidae; Polistinae	
	167	27	*Pseudomasaris vespoides*	CLITNCPRG	GAXQ01038846.1			Apocrita; Aculeata; Vespoidea; Vespidae; Masarinae	
	168	28	*Chyphotes mellipes*	CLITNCPRG	GAXL01043733.1	+	GAXL01018116.1	Apocrita; Aculeata; Vespoidea; Bradynobaenidae; Chyphotinae	
	169	29	*Brachycistis timberlakei*	CLITNCPRG	GAZU01023639.1|: 2211-2657	+	GAZU01029175.1	Apocrita;Aculeata; Vespoidea; Tiphiidae; Brachycistidinae	

	170	30	*Crioscolia alcione*	CLITNCPRG	GAXT01010223.1|: 1-207	+	GAXT01013548.1	Apocrita; Aculeata; Vespoidea; Scoliidae	
	171	31	*Tetramorium bicarinatum*	+	GASM01006298.1	+	GASM01007371.1	Apocrita; Aculeata; Vespoidea; Formicidae; Myrmicinae	ants
	172	32	*Wasmannia auropunctata*	CLITNCPRG	XP_011702880.1	+	XM_011691615.1	Apocrita; Aculeata; Vespoidea; Formicidae; Myrmicinae	
	173	33	*Pogonomyrmex barbatus*	CLITNCPRG	XP_011645557.1	+	XP_011639495.1	Apocrita; Aculeata; Vespoidea; Formicidae; Myrmicinae	
	174	34	*Vollenhovia emeryi*	CLITNCPRG	XP_011871987.1	+	XP_011350516.1	Apocrita; Aculeata; Vespoidea; Formicidae; Myrmicinae	
	175	35	*Acromyrmex echinatior*	CLITNCPRG	XP_011065328.1	+	EGI60623.1	Apocrita; Aculeata; Vespoidea; Formicidae; Myrmicinae	
	176	36	*Atta colombica*	CLITNCPRG	LKEW01016192.1	+	LKEW01024766.1	Apocrita; Aculeata; Vespoidea; Formicidae; Myrmicinae	
	177	37	*Atta cephalotes*	CLITNCPRG	XP_012061245.1	+	gb|LKEW01024766 .1	Apocrita; Aculeata; Vespoidea; Formicidae; Myrmicinae	
	178	38	*Monomorium pharaonis*	CLITNCPRG	XP_012527594.1	+	XP_012527228.1	Apocrita; Aculeata; Vespoidea; Formicidae; Myrmicinae	
	179	39	*Trachymyrmex cornetzi*	CLITNCPRG	KYN19822.1	+	LKEY01027754.1	Apocrita; Aculeata; Vespoidea; Formicidae; Myrmicinae	
	180	40	*Trachymyrmex zeteki*	CLITNCPRG	KYQ46204.1	+	LKFA01015596.1	Apocrita; Aculeata; Vespoidea; Formicidae; Myrmicinae	
	181	41	*Trachymyrmex septentrionalis*	CLITNCPRG	KYN39529.1	+	LKEZ01023603.1	Apocrita; Aculeata; Vespoidea; Formicidae; Myrmicinae	
	182	42	*Solenopsis invicta*	CLITNCPRG	LJ563171.1	+	XP_011159612.1	Apocrita; Aculeata; Vespoidea; Formicidae; Myrmicinae	
	183	43	*Cyphomyrmex costatus*	CLITNCPRG	LKEX01018246.1; LKEX01015288.1; LKEX01011223.1	+	gb|LKEX01019312 .1|	Apocrita; Aculeata; Vespoidea; Formicidae; Myrmicinae	
	184	44	*Lasius niger*	CLITNCPRG	LBMM01006435.1	+	LBMM01005371.1; LBMM01020432.1	Apocrita; Aculeata; Vespoidea; Formicidae; Formicinae	
	185	45	*Camponotus floridanus*	CLIVNCPRG	gnl|Cflo_3.3|CFLO 12125-RA; gnl|Cflo_3.3|CFLO 19205-RA	+	AEAB01029474.1; AEAB01029475.1	Apocrita; Aculeata; Vespoidea; Formicidae; Formicinae	
	186	46	*Harpegnathos saltator*	CLITNCPRG	XP_011147500.1	+	XP_011151734.1	Apocrita; Aculeata; Vespoidea; Formicidae; Ponerinae	
	187	47	*Dinoponera quadriceps*	CLITNCPHG	XP_014485987.1	+	XP_014470481.1	Apocrita; Aculeata; Vespoidea; Formicidae; Ponerinae	
	188	48	*Linepithema humile*	CLITNCPRG	XP_012222809.1	+	ADOQ01003105.1	Apocrita; Aculeata; Vespoidea; Formicidae; Dolichoderinae	
	189	49	*Cerapachys biroi*	CLITNCPRG	XP_011329433.1	+	XP_011350516.1	Apocrita; Aculeata; Vespoidea; Formicidae; Cerapachyinae	
	190	50	*Stigmatomma oregonense*			+	GAXR01031290.1	Apocrita; Aculeata; Vespoidea; Formicidae; Amblyoponinae	
	191	51	*Exoneura robusta*					Apocrita; Aculeata; Apoidea; Apidae; Xylocopinae	bees
	192	52	*Habropoda laboriosa*					Apocrita; Aculeata; Apoidea; Apidae; Anthophorinae	
	193	53	*Melipona quadrifasciata*					Apocrita; Aculeata; Apoidea; Apidae; Meliponinae	
	194	54	*Apis mellifera*					Apocrita; Aculeata; Apoidea; Apidae; Apinae	
	195	55	*Apis dorsata*					Apocrita; Aculeata; Apoidea; Apidae; Apinae	
	196	56	*Apis florea*					Apocrita; Aculeata; Apoidea; Apidae; Apinae	
	197	57	*Apis cerana*					Apocrita; Aculeata; Apoidea; Apidae; Apinae	
	198	58	*Bombus impatiens*					Apocrita; Aculeata; Apoidea; Apidae; Bombinae	
	199	59	*Bombus terrestris*					Apocrita; Aculeata; Apoidea; Apidae; Bombinae	
	200	60	*Dufourea novaeangliae*					Apocrita; Aculeata; Apoidea; Halictidae; Rophitinae	
	201	61	*Lasioglossum albipes*					Apocrita; Aculeata; Apoidea; Halictidae; Halictinae	
	202	62	*Megachile rotundata*					Apocrita; Aculeata; Apoidea; Megachilidae; Megachilinae	
**Raphidioptera**	203	1	*Inocellia crassicornis*	+	GAZH01017073.1	+	GAZH01092118.1	Inocelliidae	
	204	2	*Xanthostigma xanthostigma*					Raphidiidae	
**Megaloptera**	205	1	*Corydalus cornutus*	CLITNCPRG	GATG01039646.1	+	GATG01035419.1	Corydalidae	
	206	2	*Sialis lutaria*					Sialidae	
**Neuroptera**	207	1	*Conwentzia psociformis*			+	GAYH01081470.1	Coniopterygidae	
	208	2	*Osmylus fulvicephalus*	+	GAYC01082079.1	+	GAYC01075986.1	Osmylidae	
	209	3	*Pseudomallada prasinus*	CLITNCPRG	GAVV02007136.1			Chrysopidae	
	210	4	*Chrysopa pallens*			+	GAGF01046297.1	Chrysopidae	

	211	5	*Euroleon nostras*					Myrmeleontidae
**Strepsiptera**	212	1	*Mengenilla moldrzyki*					Mengenillidia; Mengenilloidea; Mengenillidae
	213	2	*Stylops melittae*					Stylopidia; Stylopidae
**Coleoptera**	214	1	*Aleochara curtula*	CLITNCPRG	GATW01005644.1	+	GATW01023067.1	Polyphaga; Staphyliniformia; Staphylinoidea; Staphylinidae; Tachyporinae group; Aleocharinae
	215	2	*Nicrophorus vespilloides*	CLITNCPRG	LJCH01004160.1	+	GDKQ01006510.1	Polyphaga; Staphyliniformia; Staphylinoidea; Silphidae; Nicrophorinae
	216	3	*Dendroctonus frontalis*			+	GAFI01014329.1	Polyphaga; Cucujiformia; Curculionoidea; Curculionidae; Scolytinae
	217	4	*Dendroctonus ponderosae*	CLITNCPRG	APGL01028326.1; GAFX01014170.1			Polyphaga; Cucujiformia; Curculionoidea; Curculionidae; Scolytinae
	218	5	*Hypothenemus hampei*	CLITNCPRG	LBGY01005928.1	+	LBGY01001191.1	Polyphaga; Cucujiformia; Curculionoidea; Curculionidae; Scolytinae
	219	6	*Dastarcus helophoroides*	+	GBCX01024478.1|: 5-211			Polyphaga; Cucujiformia; Cucujoidea; Bothrideridae
	220	7	*Aethina tumida*	+	GCKB01019799.1|: 156-437	+	GCKB01003102.1	Polyphaga; Cucujiformia; Cucujoidea; Nitidulidae; Nitidulinae
	221	8	*Meloe violaceus*	CLITNCPRG	GATA01002325.1	+	GATA01005266.1	Polyphaga; Cucujiformia; Tenebrionoidea; Meloidae
	222	9	*Tribolium castaneum*	CLITNCPRG	NP_001078831.1	+	A3RE84	Polyphaga; Cucujiformia; Tenebrionoidea; Tenebrionidae
	223	10	*Anoplophora glabripennis*	CLITNCPRG	AQHT01068515.1	+	AQHT01068175.1	Polyphaga; Cucujiformia; Chrysomeloidea; Cerambycidae; Lamiinae
	224	11	*Diabrotica virgifera virgifera*	+	EW761630:18-293			Polyphaga; Cucujiformia; Chrysomeloidea; Chrysomelidae; Galerucinae
	225	12	*Leptinotarsa decemlineata*	CLITNCPKG CLITNCPIG	AYNB01151526.1	+	AYNB01191279.1	Polyphaga; Cucujiformia; Chrysomeloidea; Chrysomelidae; Chrysomelinae
	226	13	*Onthophagus taurus*	CLITNCPRG	JHOM01000375.1		JHOM01025934.1	Polyphaga; Scarabaeiformia; Scarabaeoidea; Scarabaeidae; Scarabaeinae; Scarabaeinae
	227	14	*Oryctes borbonicus*	CLITNCPRG	LJIG01009187.1	+	LJIG01025627.1	Polyphaga; Scarabaeiformia; Scarabaeoidea; Scarabaeidae; Dynastinae
	228	15	*Agrilus planipennis*	CLITNCPRG	JENH01072924.1|	+	JENH01056123.1	Polyphaga; Elateriformia; Buprestoidea; Buprestidae; Agrilinae
	229	16	*Lepicerus sp.*	+	GAZB01101514.1; GAZB01008737.1; GAZB01008738.1			Myxophaga; Lepiceridae
	230	17	*Priacma serrata*	CLITNCPRG	GACO01007629.1			Archostemata; Cupedidae
	231	18	*Gyrinus marinus*	CLITNCPRG	GAUY01035880.1	+	GAUY01010995.1	Adephaga; Gyrinoidea; Gyrinidae
	232	19	*Pogonus chalceus*	+	JU438286.1|:2-208	+	JU436954.1	Adephaga; Caraboidea; Carabidae; Trechinae
	233	20	*Carabus granulatus*			+	GACW01037691.1	Adephaga; Caraboidea; Carabidae; Carabinae
**Trichoptera**	234	1	*Rhyacophila fasciata*					
	235	2	*Platycentropus radiatus*					
	236	3	*Hydroptila spp.*					
	237	4	*Philopotamus ludificatus*					
	238	5	*Annulipalpia sp.*					
**Lepidoptera**[c]	239	1	*Micropterix calthella*					
	240	2	*Dyseriocrania subpurpurella*					
	241	3	*Triodia sylvina*					
	242	4	*Nemophora degeerella*					
	243	5	*Yponomeuta evonymella*					
	244	6	*Zygaena fausta*					
	245	7	*Polyommatus icarus*					
	246	8	*Parides eurimedes*					
	247	9	*Bombyx mori*					

	248	10	*Manduca sexta*				
**Siphonaptera**	249	1	*Ceratophyllus gallinae*				
	250	2	*Archaeopsylla erinacei*				
	251	3	*Ctenocephalides felis*				
**Mecoptera**	252	1	*Boreus hyemalis*				
	253	2	*Nannochorista philpotti*				
	254	3	*Bittacus pilicornis*				
	255	4	*Panorpa vulgaris*				
**Diptera**[c]	256	1	*Anopheles gambiae*				
	257	2	*Aedes aegypti*				
	258	3	*Phlebotomus papatasi*				
	259	4	*Trichocera saltator*				
	260	5	*Tipula maxima*				
	261	6	*Bibio marci*				
	262	7	*Bombylius major*				
	263	8	*Drosophila melanogaster*				
	264	9	*Lipara lucens*				
	265	10	*Rhagoletis pomonella*				
	266	11	*Glossina morsitans morsitans*				
	267	12	*Sarcophaga crassipalppis*				
	268	13	*Arachnocampa luminosa*	CYITNCPWG	GDQV01021566.1	+	GDQV01043269.1
	269	14	*Triarthria setipennis*				

Colour codes:	only oxytocin-like precursor or receptor	both oxytocin-like precursor and receptor	genomes available	more than 1 receptor/precursor sequence	not annotated

[a]Three first groups of Arthropoda (Chelicerata, Myriapoda and Crustaceans, underlined) represent subphylum; all others are orders of the subphylum Hexapoda; the class Insecta comprises the orders from Archaeognatha to Diptera.
[b]Number of '+' shows the number of different found receptor copies.
[c]Not all negative hits were included although 99 Diptera and 25 Lepidoptera species found in the NCBI genome database (13th of April 2016) were included in the analysis.

Supplementary Table S2. List of species and accession numbers used to prepare dataset

Organism[a]	BioProject ID	BioSample Accession	Transcriptome Accession	Download source
Acanthocasuarina muellerianae	219519	SAMN02047100	GAYY00000000	NCBI, TSA
Acanthosoma haemorrhoidale	219520	SAMN02047154	GAUV00000000	NCBI, TSA
Acerentomon sp. AD-2013	219521	SAMN02047102	GAXE00000000	NCBI, TSA
Aleochara curtula	219522	SAMN02047128	GATW00000000	NCBI, TSA
Annulipalpia sp. AD-2013	219539	SAMN02047195	GATX00000000	NCBI, TSA
Anurida maritima	219523	SAMN02047180	GAUE00000000	NCBI, TSA
Apachyus charteceus	219524	SAMN02047175	GAUW00000000	NCBI, TSA
Aposthonia japonica	219525	SAMN02047170	GAWU00000000	NCBI, TSA
Aretaon asperrimus	219526	SAMN02047129	GAZQ00000000	NCBI, TSA
Atelura formicaria	219527	SAMN02047107	GAYJ00000000	NCBI, TSA
Baetis sp. AD-2013	219528	SAMN02047149	GATU00000000	NCBI, TSA
Bibio marci	219529	SAMN02047144	GATJ00000000	NCBI, TSA
Bittacus pilicornis	219530	SAMN02047197	GATH00000000	NCBI, TSA
Blaberus atropos	219531	SAMN02047121	GAYD00000000	NCBI, TSA
Bombylius major	219532	SAMN02047145	GATI00000000	NCBI, TSA
Boreus hyemalis	219533	SAMN02047164	GAYK00000000	NCBI, TSA
Calopteryx splendens	219534	SAMN02047184	GAYM00000000	NCBI, TSA
Campodea augens	219535	SAMN02047108	GAYN00000000	NCBI, TSA
Ceratophyllus gallinae	219536	SAMN02047116	GAWK00000000	NCBI, TSA
Cercopis vulnerata	219537	SAMN02047155	GAUN00000000	NCBI, TSA
Ceuthophilus sp. AD-2013	219538	SAMN02047189	GAUX00000000	NCBI, TSA
Chrysis viridula	219540	SAMN02047158	GATY00000000	NCBI, TSA
Conwentzia psociformis	219541	SAMN02047147	GAYH00000000	NCBI, TSA
Cordulegaster boltonii	219542	SAMN02047156	GAYO00000000	NCBI, TSA
Corydalus cornutus	219543	SAMN02047201	GATG00000000	NCBI, TSA
Cosmioperla kuna	219544	SAMN02047101	GAYL00000000	NCBI, TSA
Cotesia vestalis	219545	SAMN02047178	GAUP00000000	NCBI, TSA
Cryptocercus wrighti	219546	SAMN02047199	GAZN00000000	NCBI, TSA
Ctenocephalides felis	219547	SAMN02047194	GAYP00000000	NCBI, TSA
Dyseriocrania subpurpurella	219549	SAMN02047140	GASY00000000	NCBI, TSA
Ectopsocus briggsi	219550	SAMN01801569	GAPT00000000	NCBI, TSA
Empusa pennata	219551	SAMN02047168	GAWT00000000	NCBI, TSA
Ephemera danica	219552	SAMN02047152	GAUK00000000	NCBI, TSA
Epiophlebia superstes	219553	SAMN02047171	GAVW00000000	NCBI, TSA
Essigella californica	219554	SAMN02047099	GAZF00000000	NCBI, TSA
Euroleon nostras	219555	SAMN02047165	GAXW00000000	NCBI, TSA
Eurylophella sp. AD-2013	219556	SAMN02047200	GAZG00000000	NCBI, TSA
Folsomia candida	219557	SAMN02047120	GASX00000000	NCBI, TSA
Forficula auricularia	219558	SAMN02047143	GAYQ00000000	NCBI, TSA
Frankliniella cephalica	219559	SAMN02047110	GAYE00000000	NCBI, TSA
Galloisiana yuasai	219560	SAMN02047172	GAWN00000000	NCBI, TSA
Grylloblatta	219561	SAMN02047192	GAWP00000000	NCBI, TSA

bifratrilecta				
Gryllotalpa sp. AD-2013	219562	SAMN02047167	GAWZ00000000	NCBI, TSA
Gynaikothrips ficorum	219563	SAMN02047111	GAXG00000000	NCBI, TSA
Gyrinus marinus	219564	SAMN02047132	GAUY00000000	NCBI, TSA
Haploembia palaui	219565	SAMN02047183	GAZA00000000	NCBI, TSA
Hydroptila sp. AD-2013	219566	SAMN02047190	GAVM00000000	NCBI, TSA
Inocellia crassicornis	219567	SAMN02047103	GAZH00000000	NCBI, TSA
Isonychia bicolor	219568	SAMN02047198	GAXA00000000	NCBI, TSA
Lepicerus sp. AD-2013	219569	SAMN02047177	GAZB00000000	NCBI, TSA
Leptopilina clavipes	219570	SAMN02047179	GAXY00000000	NCBI, TSA
Leuctra sp. AD-2013	219571	SAMN02047153	GAUF00000000	NCBI, TSA
Lipara lucens	219572	SAMN02047130	GAZD00000000	NCBI, TSA
Liposcelis bostrychophila	219573	SAMN02047187	GAYV00000000	NCBI, TSA
Machilis hrabei	219574	SAMN02047109	GAUM00000000	NCBI, TSA
Mantis religiosa	219575	SAMN02047157	GASW00000000	NCBI, TSA
Mastotermes darwiniensis	219576	SAMN02047123	GAZE00000000	NCBI, TSA
Meinertellus cundinamarcensis	219577	SAMN02047182	GAUG00000000	NCBI, TSA
Meloe violaceus	219578	SAMN02047163	GATA00000000	NCBI, TSA
Menopon gallinae	219579	SAMN02047141	GAWR00000000	NCBI, TSA
Metallyticus splendidus	219580	SAMN02047174	GATB00000000	NCBI, TSA
Nemophora degeerella	219581	SAMN02047104	GATC00000000	NCBI, TSA
Nilaparvata lugens	219582	SAMN02047185	GAYF00000000	NCBI, TSA
Notostira elongata	219583	SAMN02047151	GASV00000000	NCBI, TSA
Occasjapyx japonicus	219584	SAMN02047169	GAXJ00000000	NCBI, TSA
Okanagana villosa	219585	SAMN02047193	GAWQ00000000	NCBI, TSA
Orussus abietinus	219586	SAMN02047118	GAUJ00000000	NCBI, TSA
Osmylus fulvicephalus	219587	SAMN02047166	GAYC00000000	NCBI, TSA
Panorpa vulgaris	219588	SAMN02047142	GAUH00000000	NCBI, TSA
Parides eurimedes	219589	SAMN02047186	GAXH00000000	NCBI, TSA
Periplaneta americana	219590	SAMN02047124	GAWS00000000	NCBI, TSA
Perla marginata	219591	SAMN02047115	GATV00000000	NCBI, TSA
Peruphasma schultei	219592	SAMN02047114	GAWJ00000000	NCBI, TSA
Planococcus citri	219593	SAMN02047127	GAXF00000000	NCBI, TSA
Platycentropus radiatus	219594	SAMN02047196	GASS00000000	NCBI, TSA
Pogonognathellus sp. AD-2013	219595	SAMN02047134	GATD00000000	NCBI, TSA
Polyommatus icarus	219596	SAMN02047136	GAST00000000	NCBI, TSA
Prorhinotermes simplex	219597	SAMN02047122	GASE00000000	NCBI, TSA
Prosarthria teretrirostris	219598	SAMN02047125	GAZT00000000	NCBI, TSA
Pseudomallada prasinus	219548	SAMN02047105	GAVV00000000	NCBI, TSA
Ranatra linearis	219599	SAMN02047138	GAYZ00000000	NCBI, TSA
Rhyacophila fasciata	219600	SAMN02047161	GAXX00000000	NCBI, TSA
Sminthurus viridis	219601	SAMN02047148	GATZ00000000	NCBI, TSA
Stenobothrus lineatus	219602	SAMN02047137	GAUZ00000000	NCBI, TSA

Stylops melittae	219603	SAMN02047139	GAZM00000000	NCBI, TSA
Tanzaniophasma sp. AD-2013	219604	SAMN02047176	GAXB00000000	NCBI, TSA
Tenthredo koehleri	219605	SAMN02047159	GAWW00000000	NCBI, TSA
Tetrix subulata	219606	SAMN02047150	GASQ00000000	NCBI, TSA
Tetrodontophora bielanensis	219607	SAMN02047160	GAXI00000000	NCBI, TSA
Thermobia domestica	219608	SAMN02047119	GASN00000000	NCBI, TSA
Thrips palmi	219609	SAMN02047112	GAXC00000000	NCBI, TSA
Timema cristinae	219610	SAMN02047191	GAVX00000000	NCBI, TSA
Trialeurodes vaporariorum	219611	SAMN02047126	GAWX00000000	NCBI, TSA
Triarthria setipennis	219612	SAMN02047117	GAVA00000000	NCBI, TSA
Trichocera saltator	219613	SAMN02047162	GAXZ00000000	NCBI, TSA
Tricholepidion gertschi	219614	SAMN02047188	GASO00000000	NCBI, TSA
Triodia sylvina	219615	SAMN02047133	GAVB00000000	NCBI, TSA
Velia caprai	219616	SAMN02047131	GAUO00000000	NCBI, TSA
Xanthostigma xanthostigma	219617	SAMN02047106	GAUI00000000	NCBI, TSA
Xenophysella greensladeae	219618	SAMN02047181	GAYI00000000	NCBI, TSA
Yponomeuta evonymellus	219619	SAMN02047146	GASG00000000	NCBI, TSA
Zorotypus caudelli	219620	SAMN02047173	GAYA00000000	NCBI, TSA
Zygaena fausta	219621	SAMN02047113	GAYB00000000	NCBI, TSA

**Organism**[b]	**Download source**	**Accession / Version**
Acromyrmex echinatior	Hymenoptera genome database	Aech_2.0
Acyrthosiphon pisum	AphidBase	ACYPI v2.0
Aedes aegypti	VectorBase	AaegL3.3
Anopheles gambiae	VectorBase	AgamP4.3
Aphis gossypii	NCBI, TSA	GW572841.1
Apis mellifera	Hymenoptera genome database	Amel_4.5
Archaeopsylla erinacei	NCBI, TSA	GABB01000001.1
Bemisia tabaci	NCBI, TSA	GAPP01000001.1
Bombus terrestris	NCBI, TSA	JL856152.1
Bombyx mori	NCBI, TSA	EL645743.1
Carabus granulatus	NCBI, TSA	GACW01000001.1
Celuca pugilator	NCBI, TSA	JO494914.1
Cypridininae sp.	NCBI, TSA	JL207200.1
Daphnia pulex	NCBI, TSA	FE425512.1
Dendroctonus ponderosae	NCBI, TSA	GAFW01000001.1
Drosophila melanogaster	FlyBase	dmel_r6.03
Exoneura robusta	NCBI, TSA	HP931235.1
Glomeris pustulata	NCBI, TSA	GAKW01000001.1
Glossina morsitans morsitans	NCBI, TSA	DV619302.1
Harpegnathos saltator	Hymenoptera genome database	Hsal_3.3
Ixodes scapularis	VectorBase	IscaW1.4
Lepeophtheirus	NCBI, TSA	JP350109.1

salmonis		
Litopenaeus vannamei	NCBI, TSA	JP403722.1
Manduca sexta	NCBI, TSA	JO840459.1
Mengenilla moldrzyki	NCBI, TSA	GACY01000001.1
Micropterix calthella	NCBI, TSA	GACP01000001.1
Nannochorista philpotti	NCBI, TSA	GADB01000001.1
Nasonia vitripennis	Hymenoptera genome database	Nvit_OGSv1.2
Pediculus humanus	VectorBase	PhumU2.1
Philopotamus ludificatus	NCBI, TSA	GACV01000001.1
Phlebotomus papatasi	NCBI, TSA	JP555361.1
Priacma serrata	NCBI, TSA	GACO01000001.1
Rhagoletis pomonella	NCBI, TSA	EZ140167.2
Sarcophaga crassipalpis	NCBI, TSA	EZ617705.1
Sarsinebalia urgorrii	NCBI, TSA	JL099665.1
Sialis lutaria	NCBI, TSA	GABK01000001.1
Speleonectes cf. tulumensis	NCBI, TSA	JL207199.1
Symphylella vulgaris	NCBI, TSA	GAKX01000001.1
Tipula maxima	NCBI, TSA	GACZ01000001.1
Tribolium castaneum	BeetleBase	Tcas_4.0
Zootermopsis nevadensis	Ensemblgenomes	GCA_000696155.1

Supplementary References

1 Pitti, T. & Manoj, N. Molecular evolution of the neuropeptide S receptor. *PLoS One* **7**, e34046, (2012).

2 Meyer-Rochow, V. B. Glowworms: a review of Arachnocampa spp. and kin. *Luminescence* **22**, 251-265, (2007).

3 Pugsley, C. W. Literature-Review of the New-Zealand Glowworm Arachnocampa-Luminosa (Diptera, Keroplatidae) and Related Cave-Dwelling Diptera. *N. Z. Entomol.* **7**, 419-424, (1983).

4 Lee, B. Y. *et al.* Whole transcriptome analysis of the monogonont rotifer Brachionus koreanus provides molecular resources for developing biomarkers of carbohydrate metabolism. *Comp. Biochem. Physiol., D: Genomics & Proteomics* **14**, 33-41, (2015).

5 Crisp, A., Boschetti, C., Perry, M., Tunnacliffe, A. & Micklem, G. Expression of multiple horizontally acquired genes is a hallmark of both vertebrate and invertebrate genomes. *Genome Biol.* **16**, 50, (2015).

6 Dunning Hotopp, J. C. *et al.* Widespread lateral gene transfer from intracellular bacteria to multicellular eukaryotes. *Science* **317**, 1753-1756, (2007).

7 Gasmi, L. *et al.* Recurrent Domestication by Lepidoptera of Genes from Their Parasites Mediated by Bracoviruses. *PLoS Genet.* **11**, e1005470, (2015).

8 Kirsch, R., Heckel, D. G. & Pauchet, Y. How the rice weevil breaks down the pectin network: Enzymatic synergism and sub-functionalization. *Insect Biochem. Mol. Biol.* **71**, 72-82, (2016).

9 Moran, N. A. & Jarvik, T. Lateral transfer of genes from fungi underlies carotenoid production in aphids. *Science* **328**, 624-627, (2010).

10 Zolfaghari Emameh, R., Barker, H. R., Tolvanen, M. E., Parkkila, S. & Hytonen, V. P. Horizontal transfer of beta-carbonic anhydrase genes from prokaryotes to protozoans, insects, and nematodes. *Parasit. Vectors* **9**, 152, (2016).
